# Supplementary figures and images for: DePARylation is critical for S phase progression and cell survival (part 1 of 2)
Source: eLife. 2024 Apr 5;12:RP89303. doi: 10.7554/eLife.89303 (PMC10997334; doi:10.7554/eLife.89303)

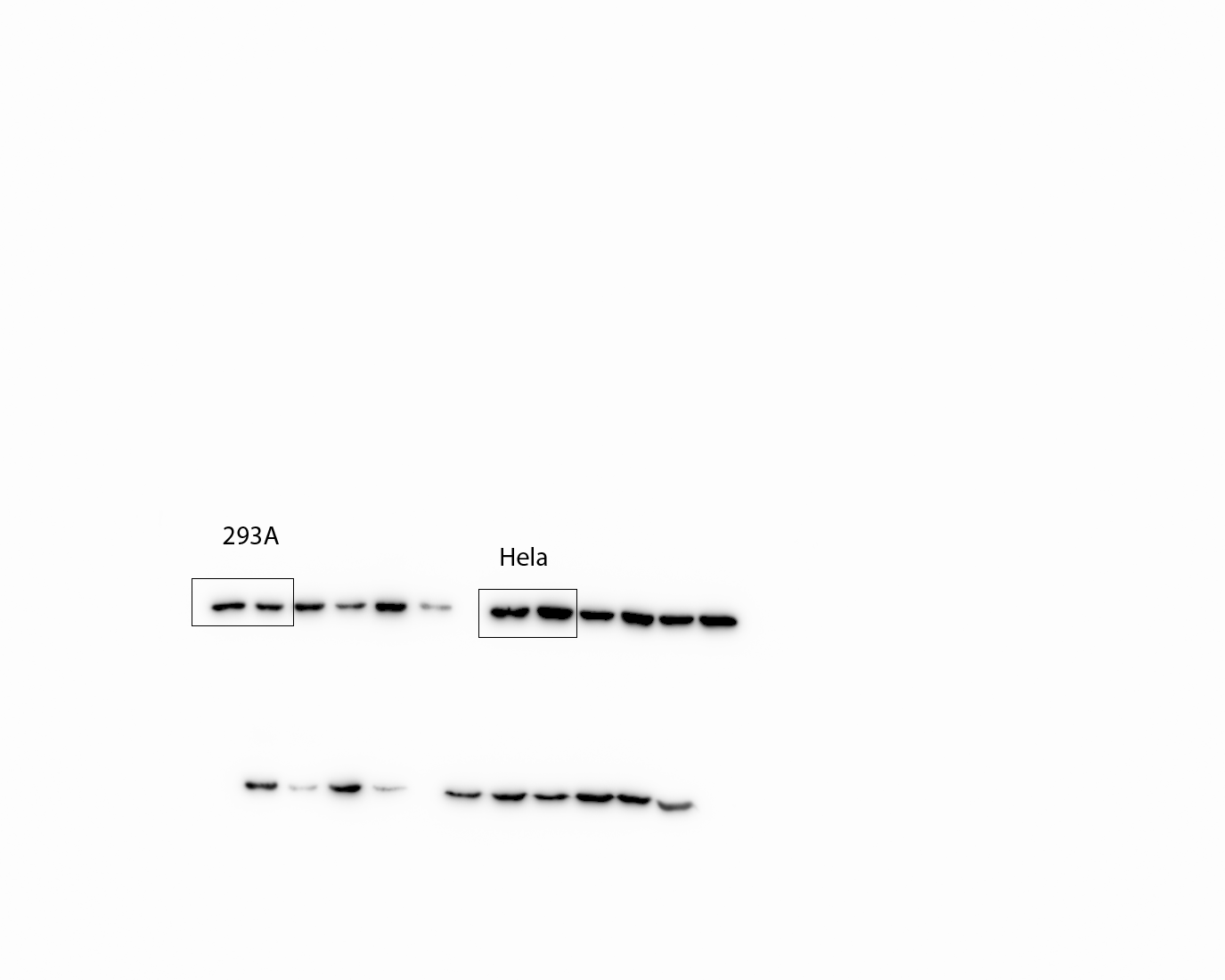

Supplement: Figure 1—source data 2. [file elife-89303-fig1-data2.zip › Figure 1-Source data 2/Actin_293A and Hela.tif]

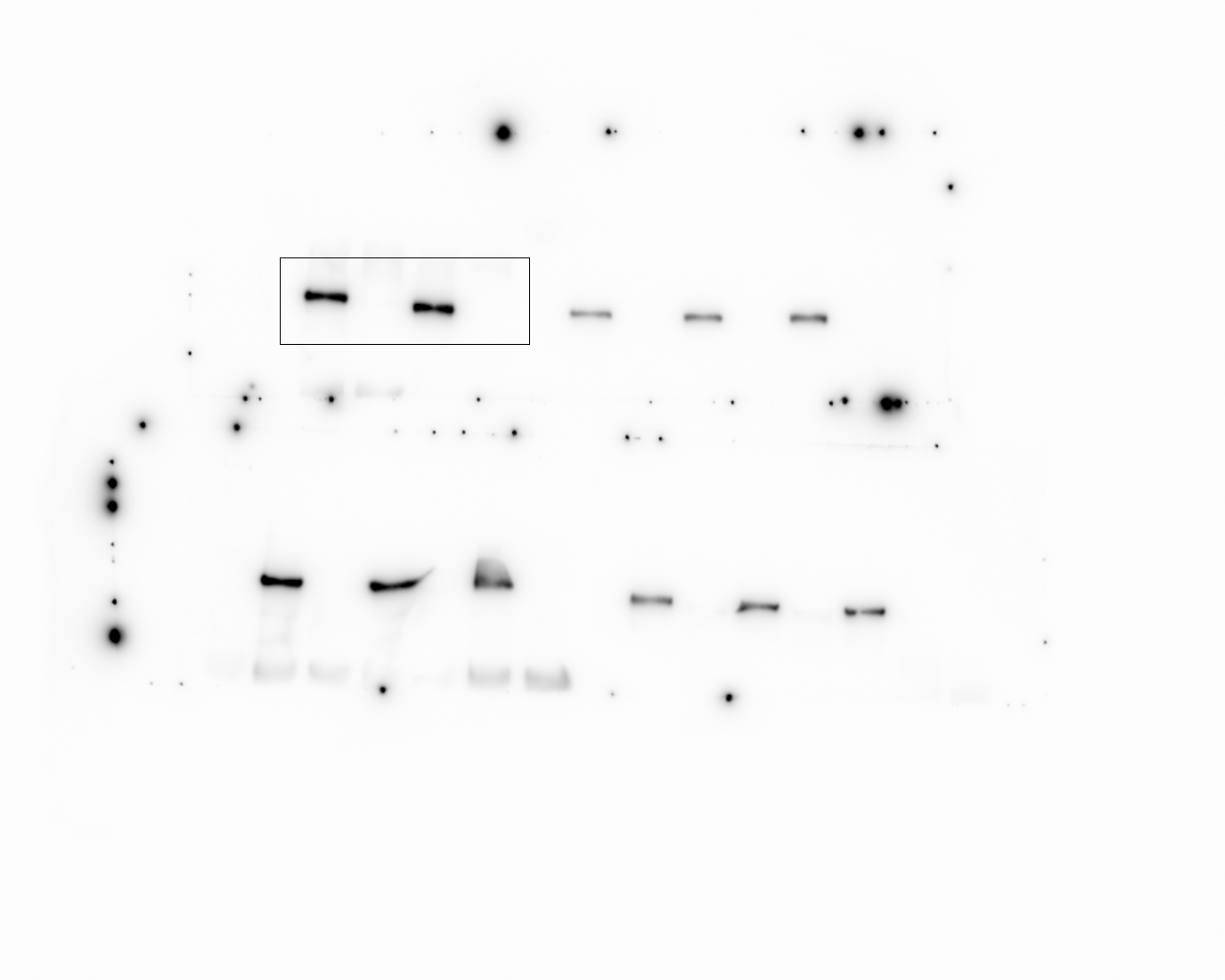

Supplement: Figure 1—source data 2. [file elife-89303-fig1-data2.zip › Figure 1-Source data 2/PARG_293A and Hela.tif]

B

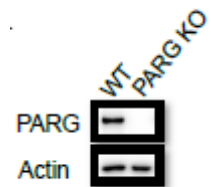

D

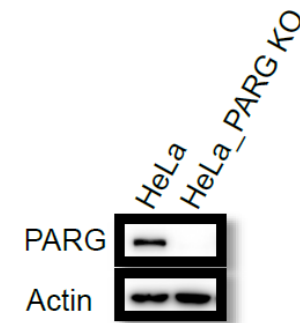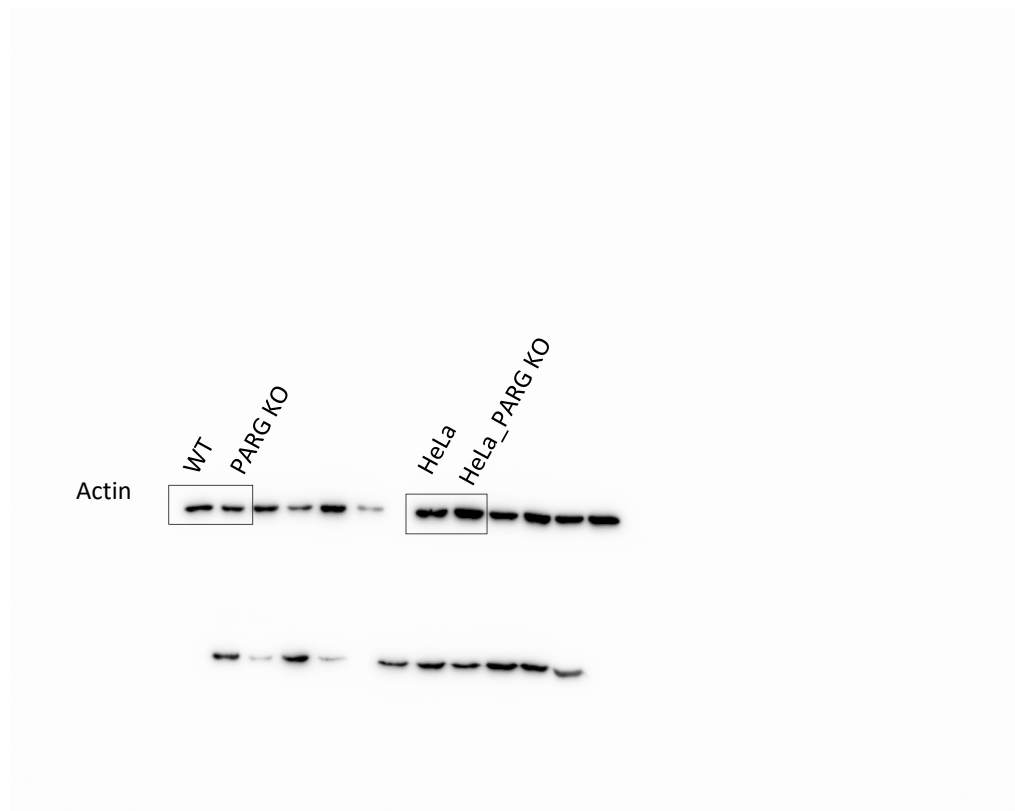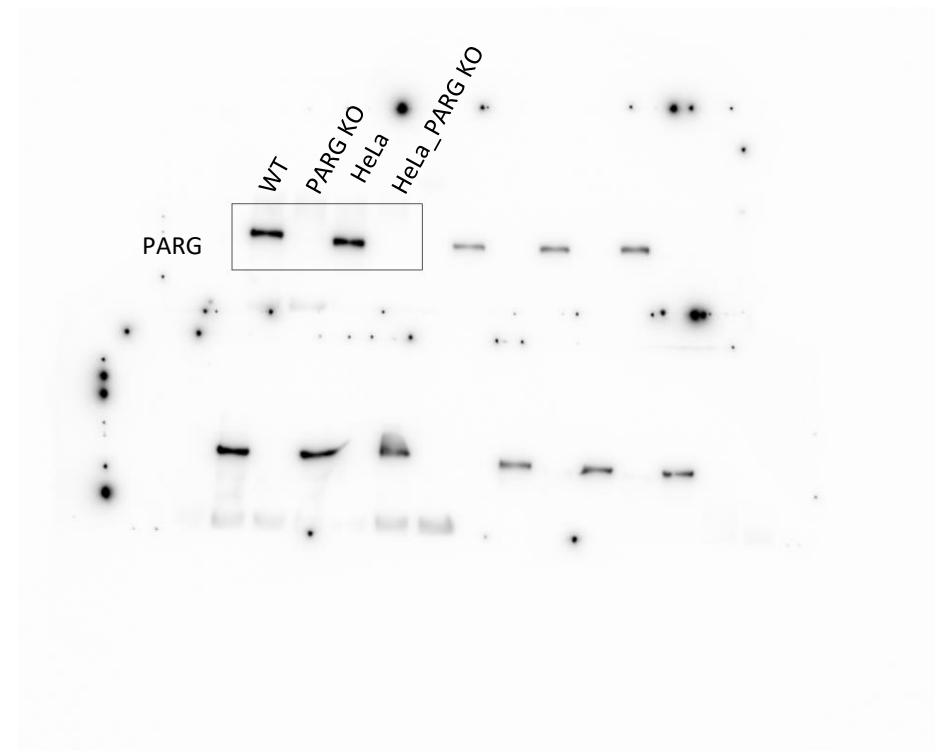

Figure 1 & Figure 1-figure supplement 1

Supplement: Figure 1—source data 3. [file elife-89303-fig1-data3.zip › Figure 1-Source data 3/Figure1-Source data 3.pdf]

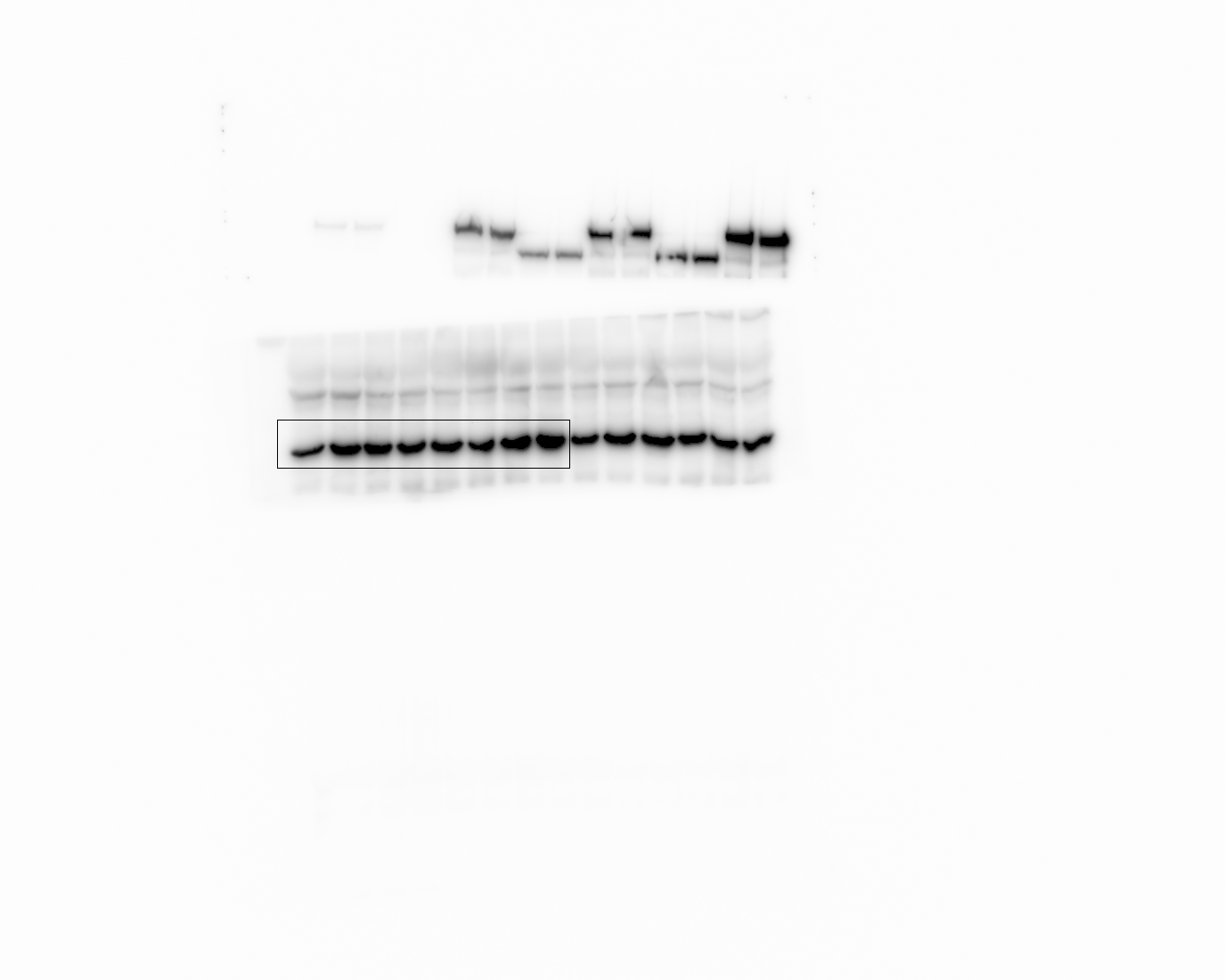

Supplement: Figure 1—figure supplement 1—source data 1. [file elife-89303-fig1-figsupp1-data1.zip › Figure 1-Figure Supplement 1-Source data 1/actin.tif]

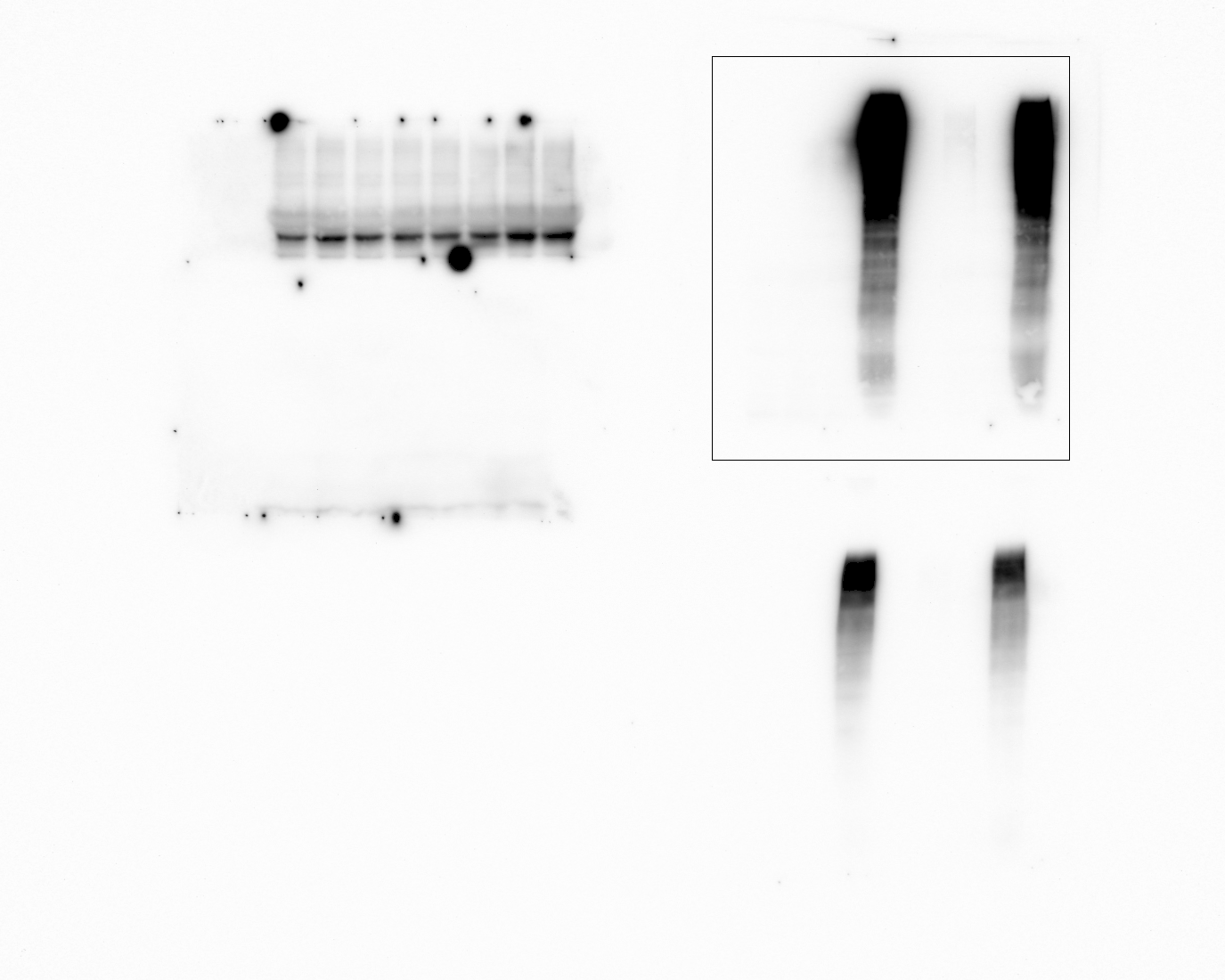

Supplement: Figure 1—figure supplement 1—source data 1. [file elife-89303-fig1-figsupp1-data1.zip › Figure 1-Figure Supplement 1-Source data 1/pADPr.tif]

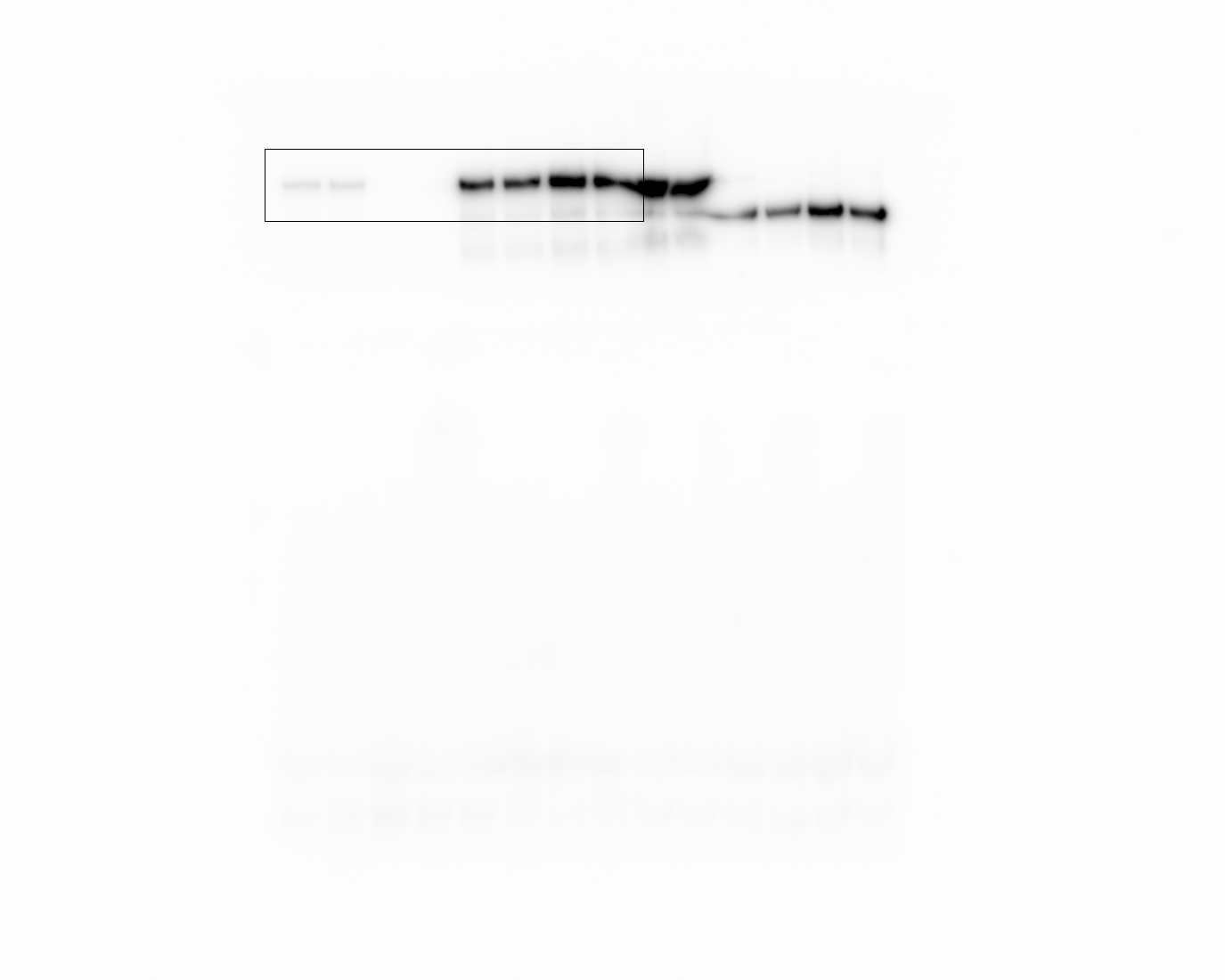

Supplement: Figure 1—figure supplement 1—source data 1. [file elife-89303-fig1-figsupp1-data1.zip › Figure 1-Figure Supplement 1-Source data 1/PARG.tif]

E

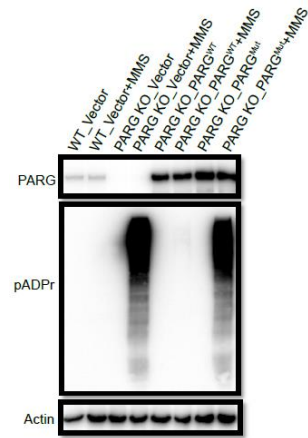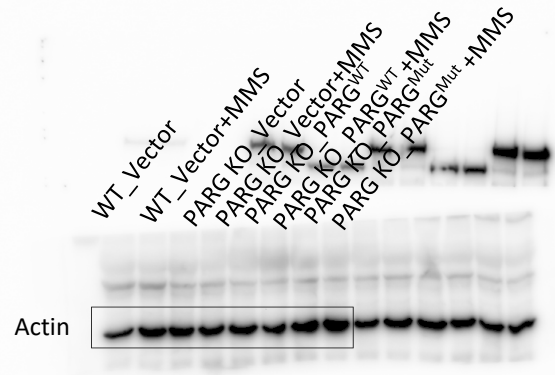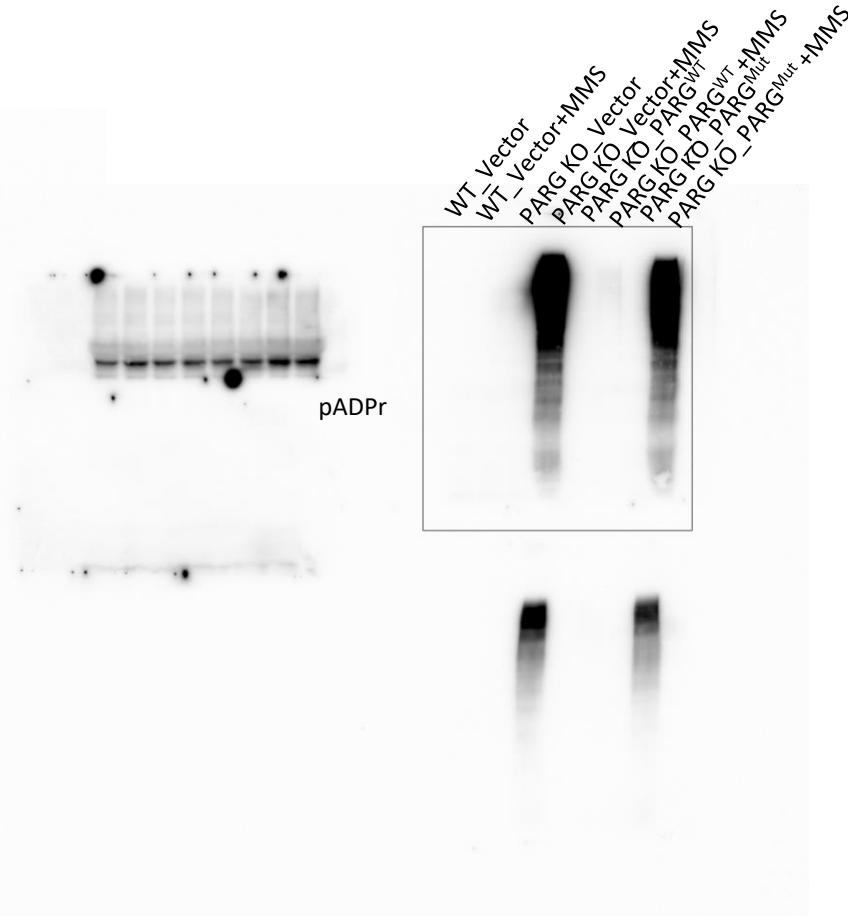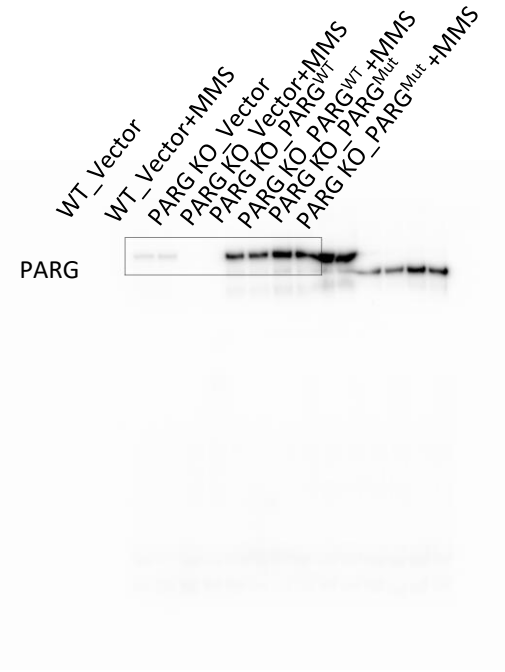

Figure 1-figure supplement 1

Supplement: Figure 1—figure supplement 1—source data 2. [file elife-89303-fig1-figsupp1-data2.zip › Figure 1-Figure Supplement 1-Source data 2/Figure1-Figure Supplement 1-Source data 2.pdf]

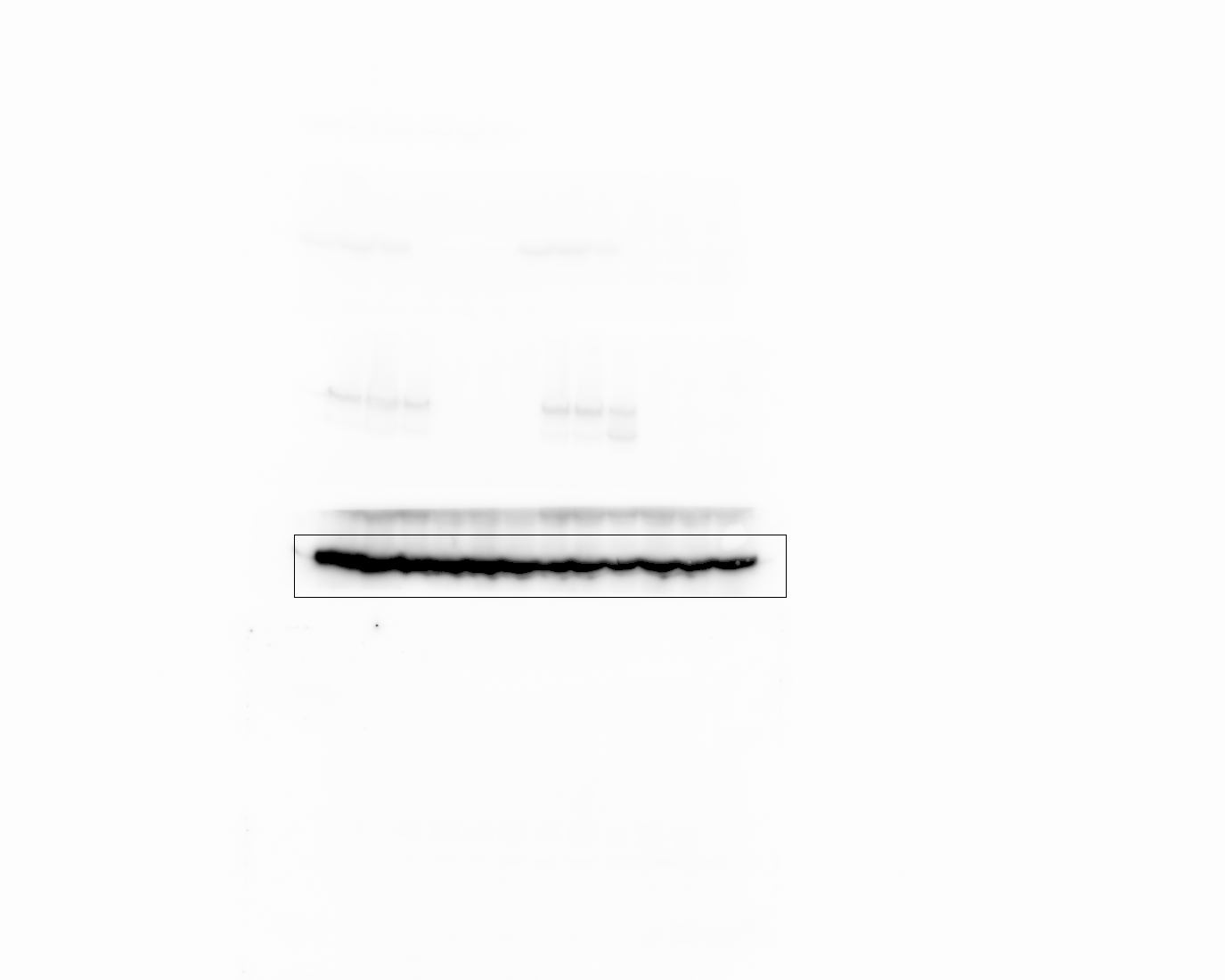

Supplement: Figure 2—source data 1. [file elife-89303-fig2-data1.zip › Figure 2-Source data 1/Figure 2A-actin.tif]

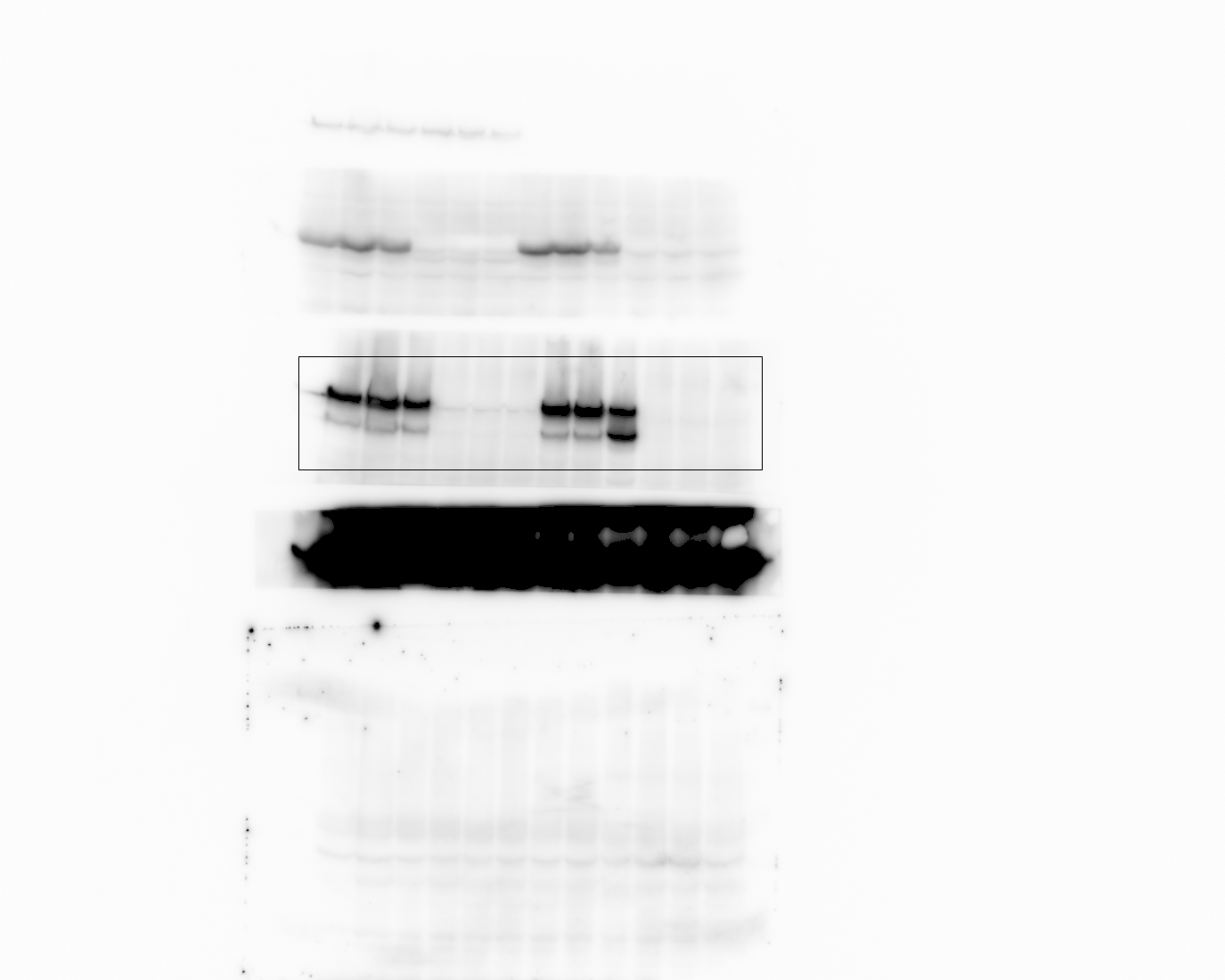

Supplement: Figure 2—source data 1. [file elife-89303-fig2-data1.zip › Figure 2-Source data 1/Figure 2A-PARP1.tif]

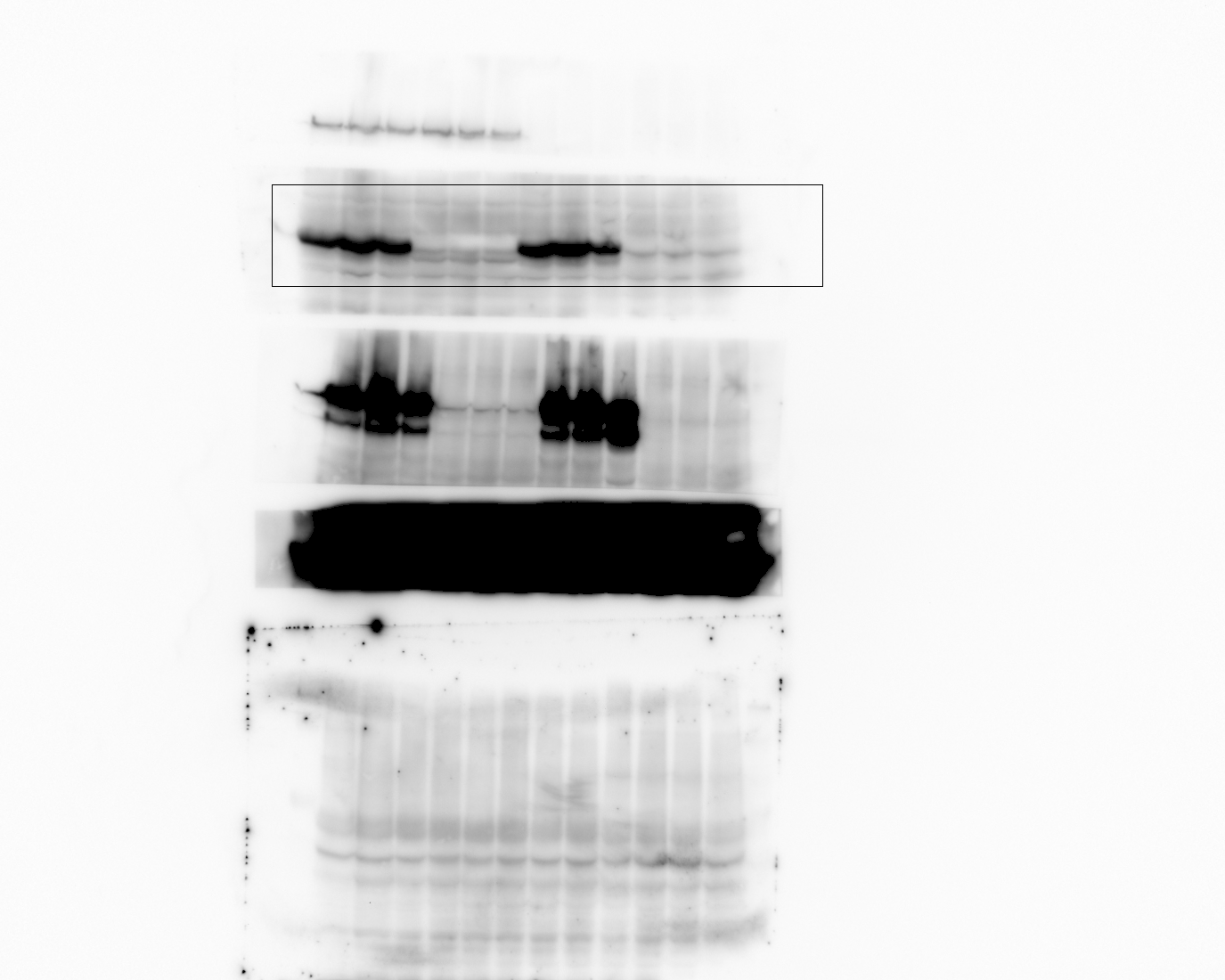

Supplement: Figure 2—source data 1. [file elife-89303-fig2-data1.zip › Figure 2-Source data 1/Figure 2A-PARP2.tif]

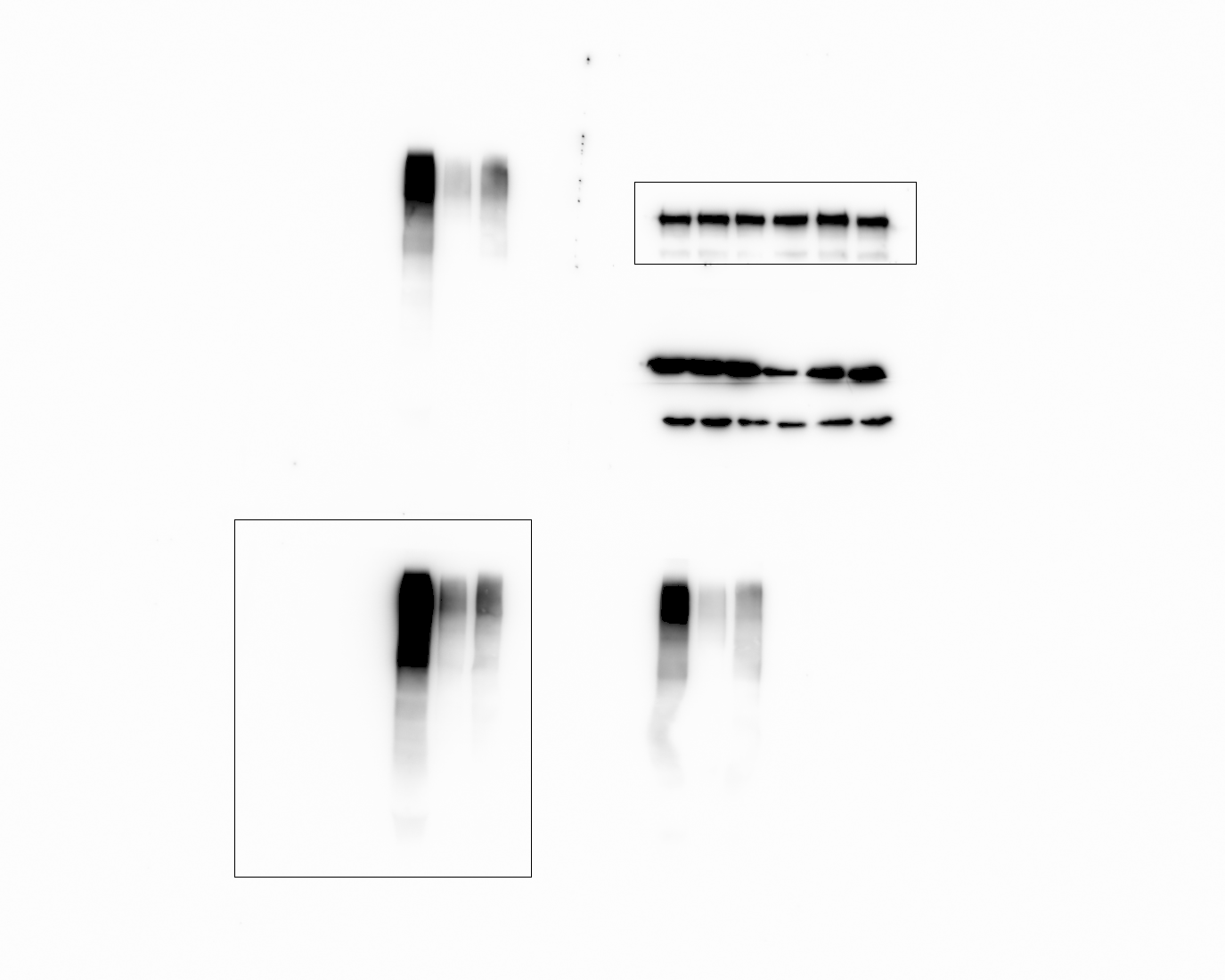

Supplement: Figure 2—source data 1. [file elife-89303-fig2-data1.zip › Figure 2-Source data 1/Figure 2E-PARP1-pADPr.tif]

A

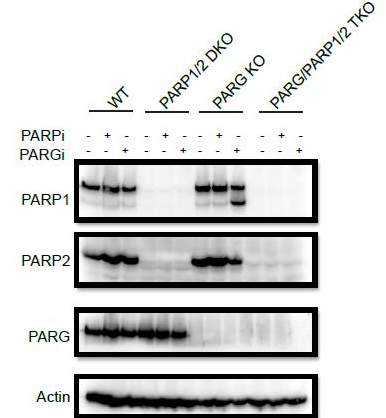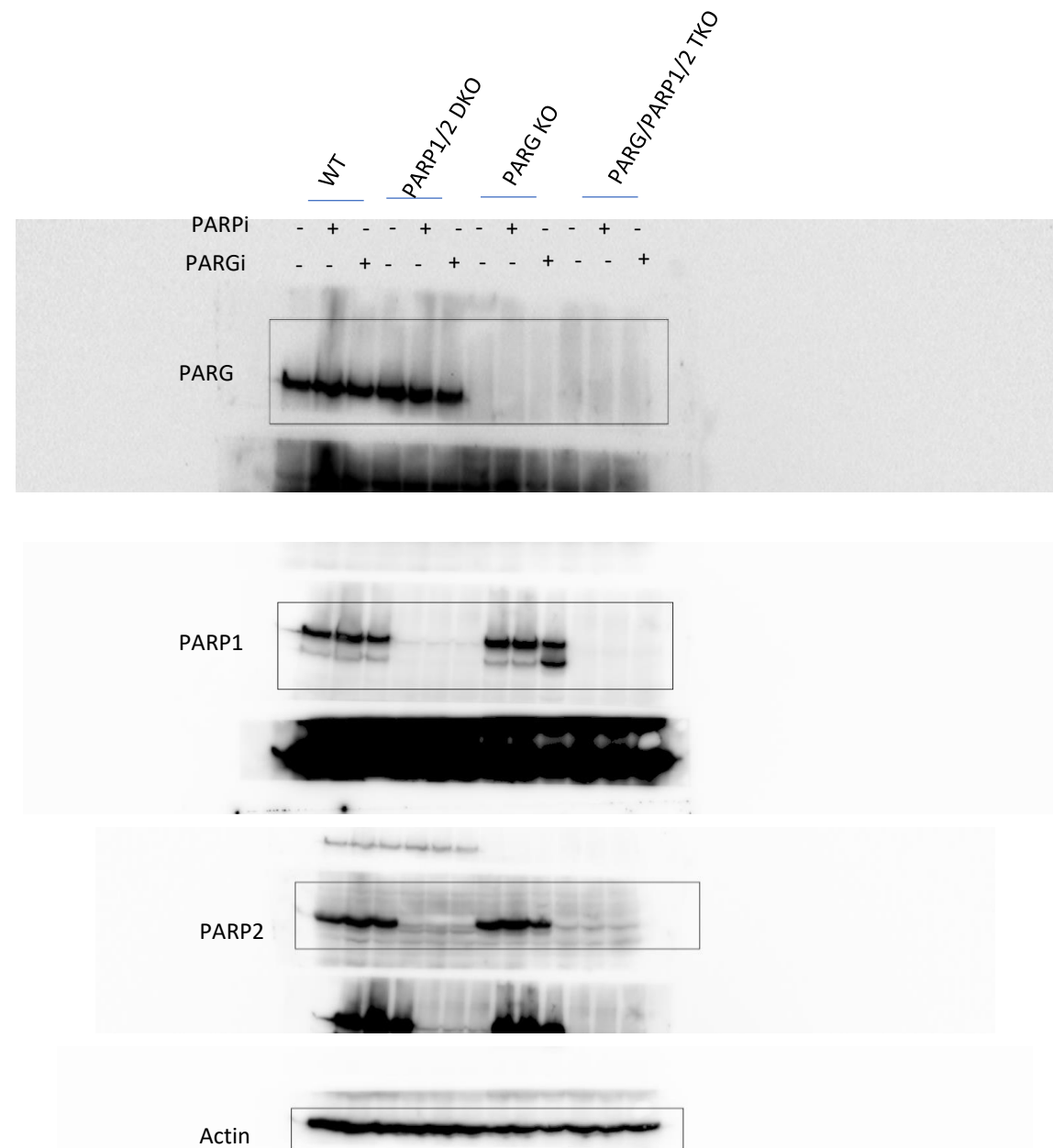

Figure 2

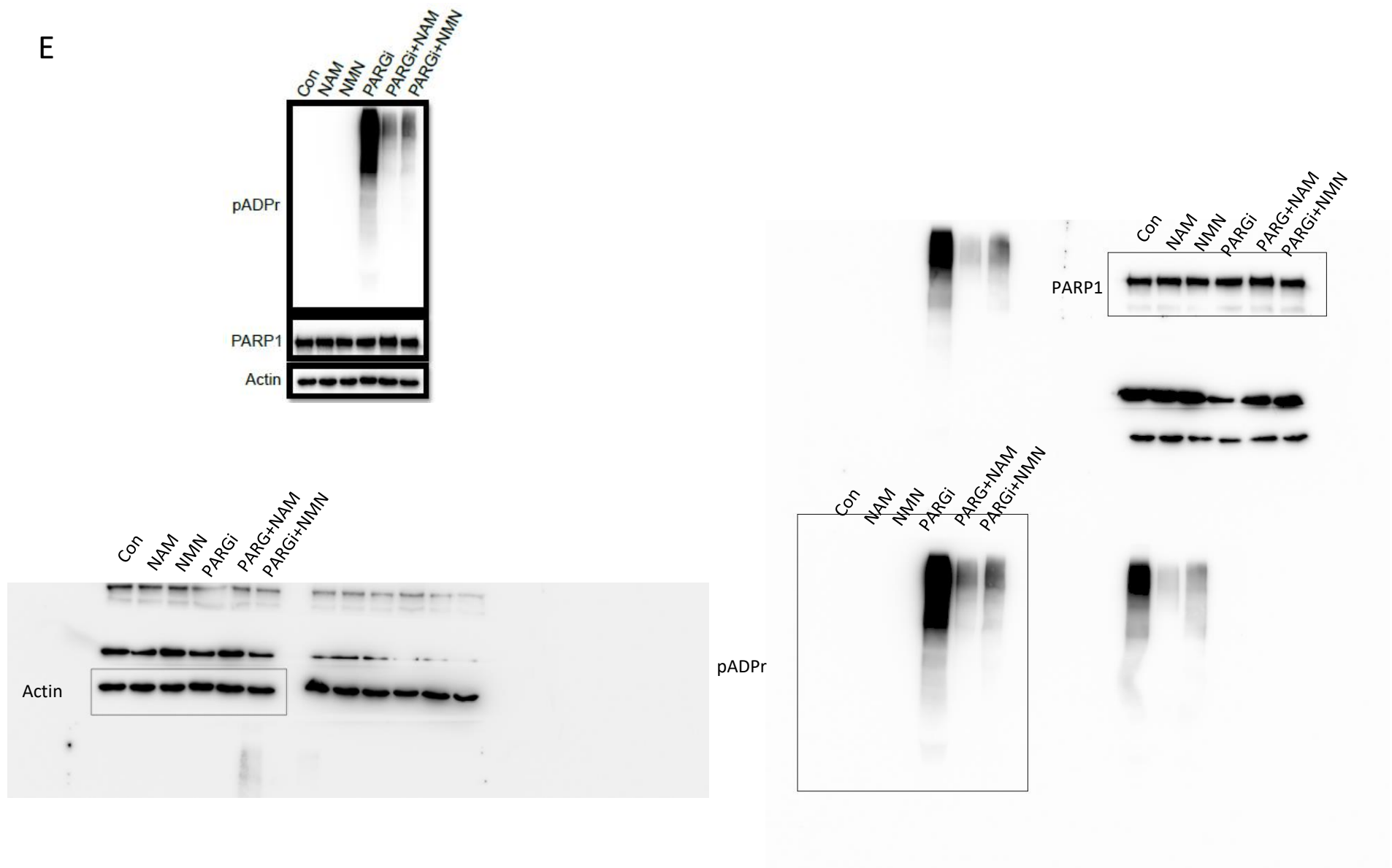

Figure 2

F

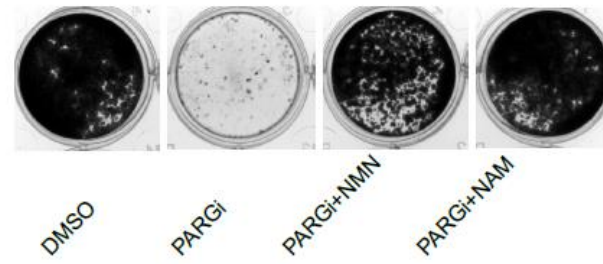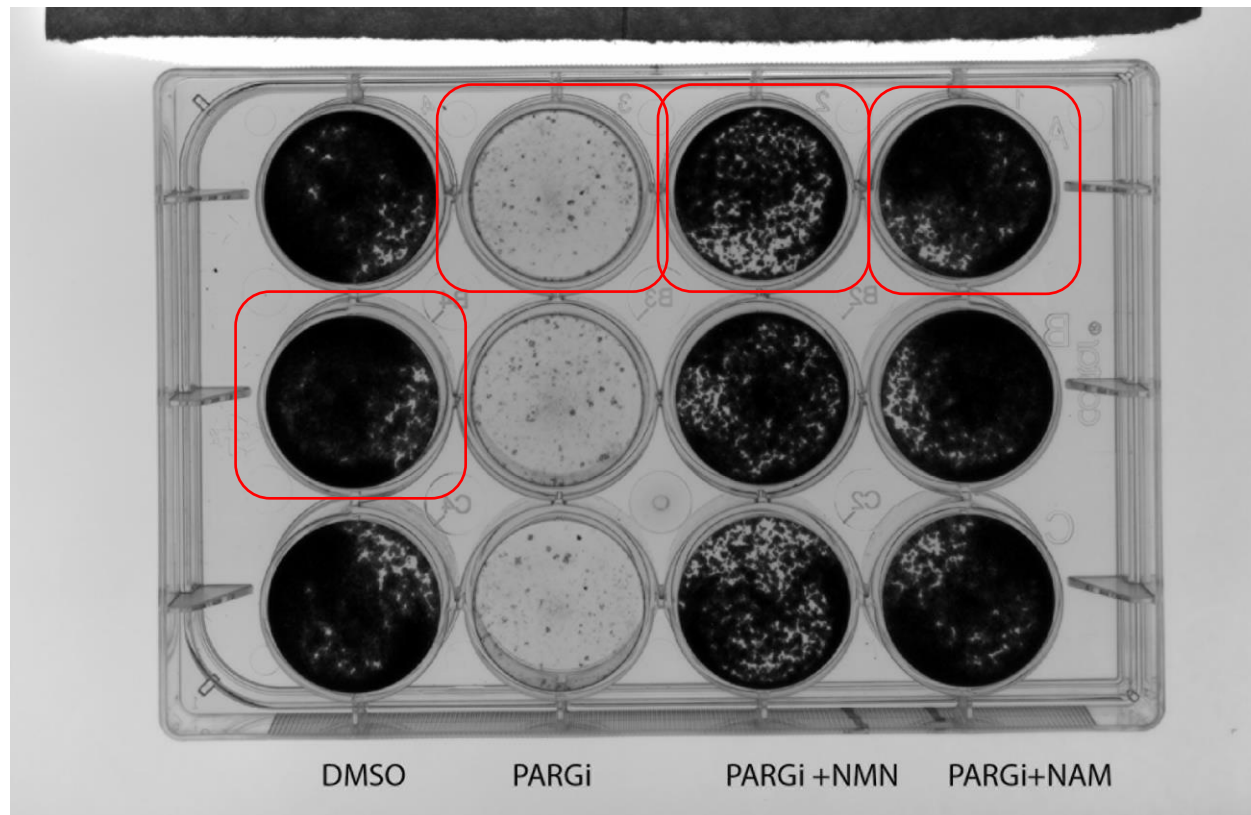

Figure 2

**B**

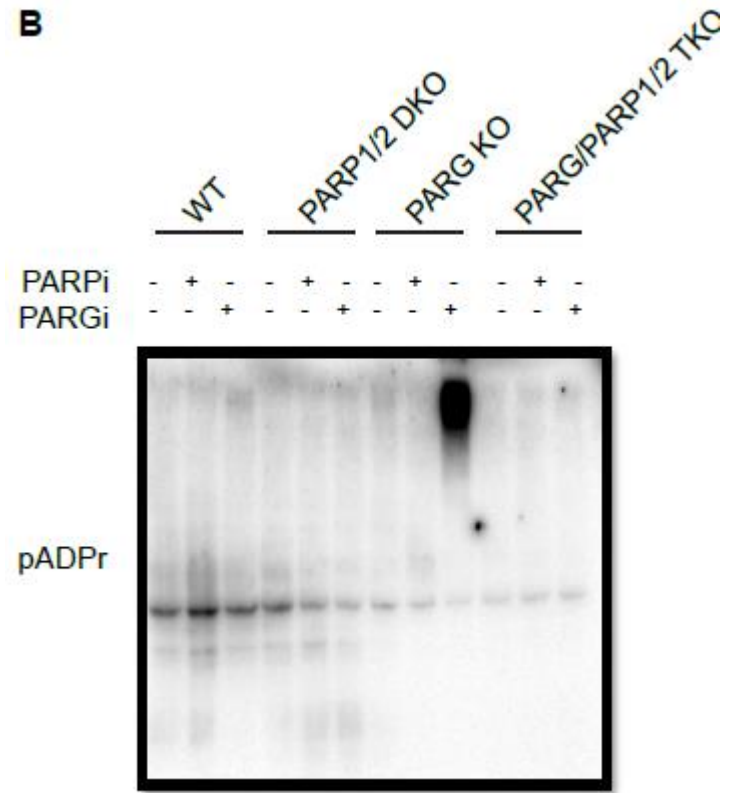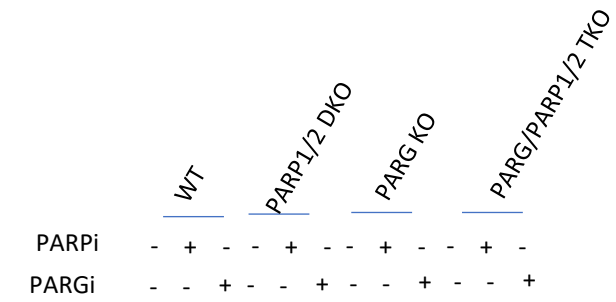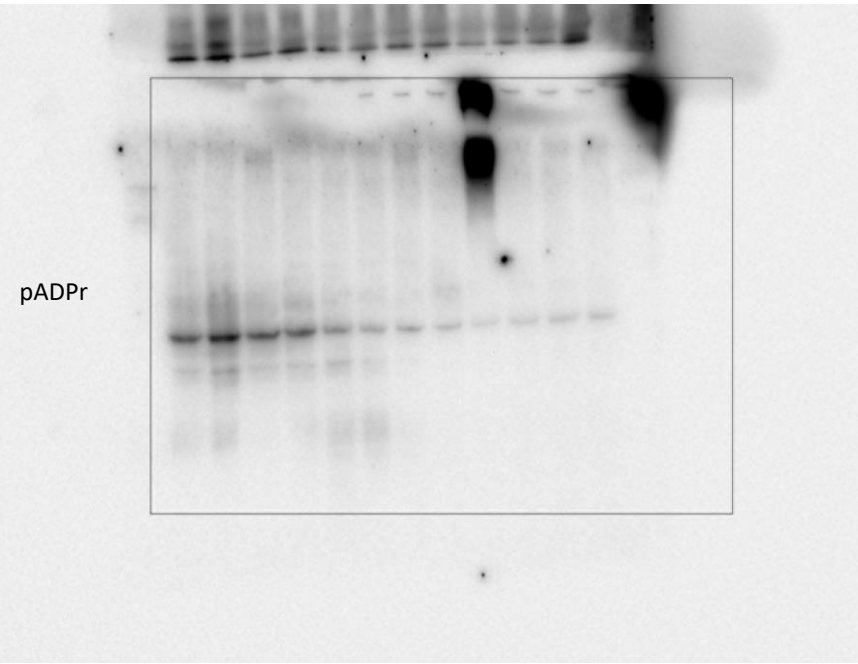

Figure 2-figure supplement 1

Supplement: Figure 2—source data 2. [file elife-89303-fig2-data2.zip › Figure 2-Source data 2/Figure 2-Source data 2.pdf]

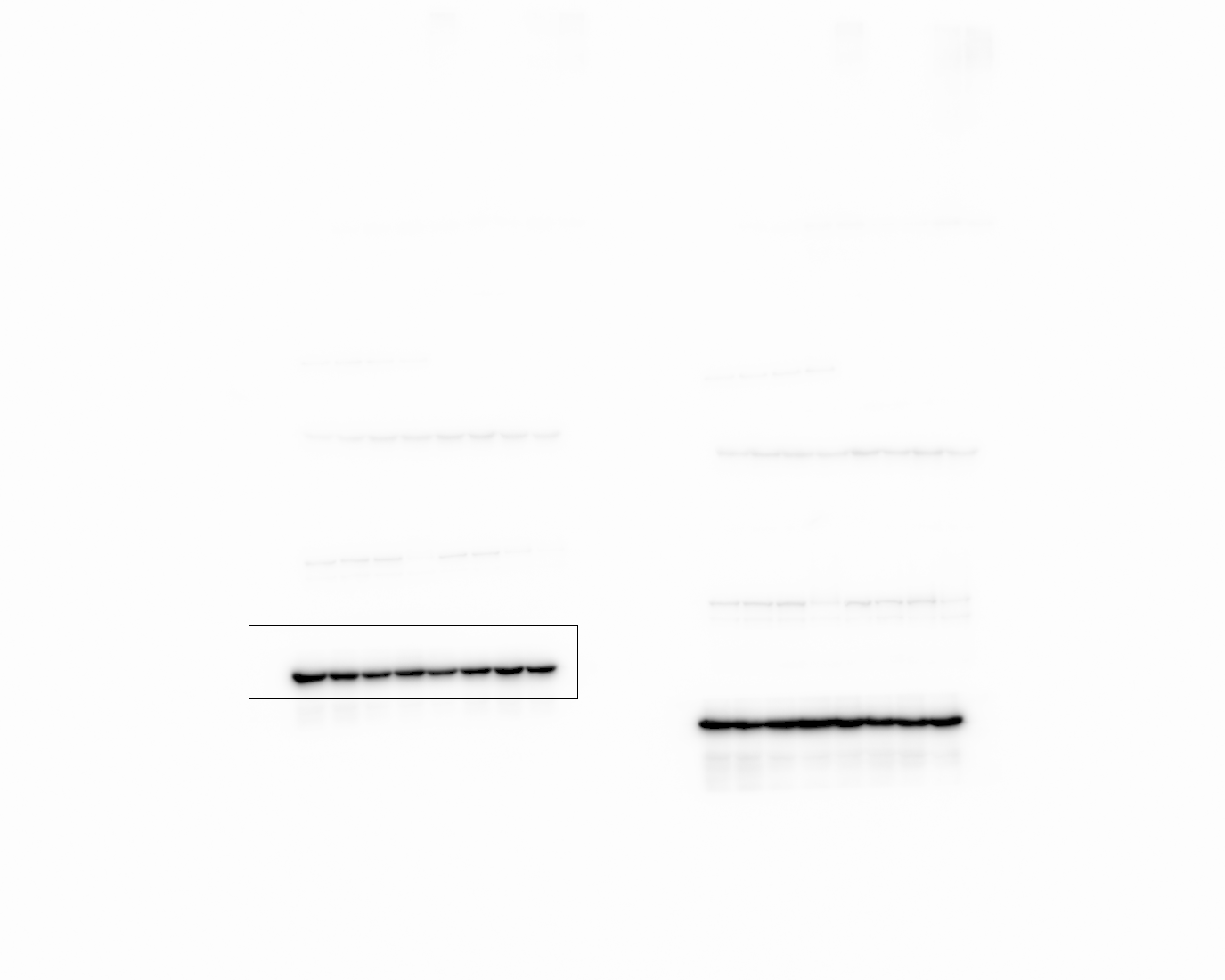

Supplement: Figure 2—figure supplement 1—source data 1. [file elife-89303-fig2-figsupp1-data1.zip › Figure 2-Figure Supplement 1-Source data 1/actin.tif]

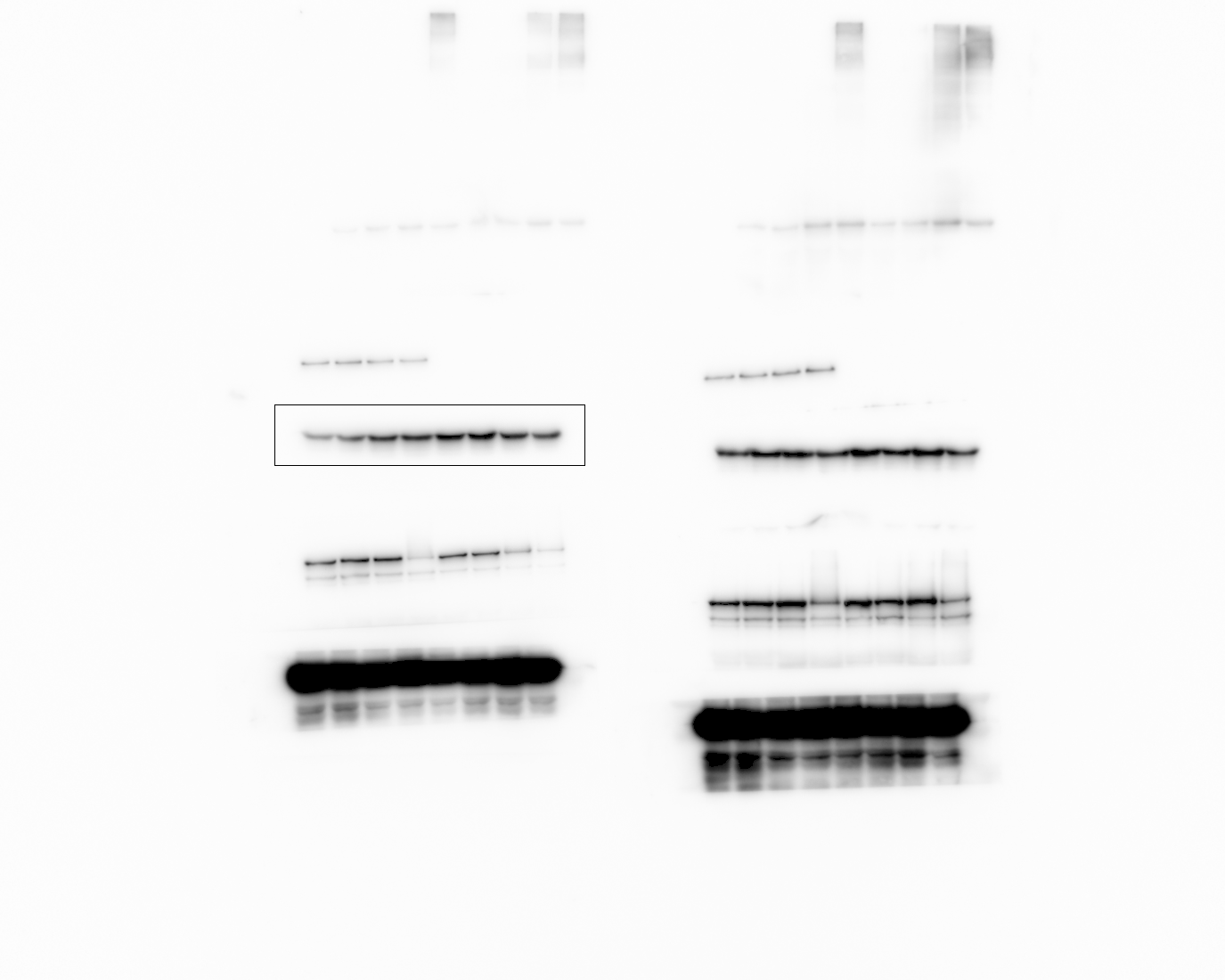

Supplement: Figure 2—figure supplement 1—source data 1. [file elife-89303-fig2-figsupp1-data1.zip › Figure 2-Figure Supplement 1-Source data 1/PARP2.tif]

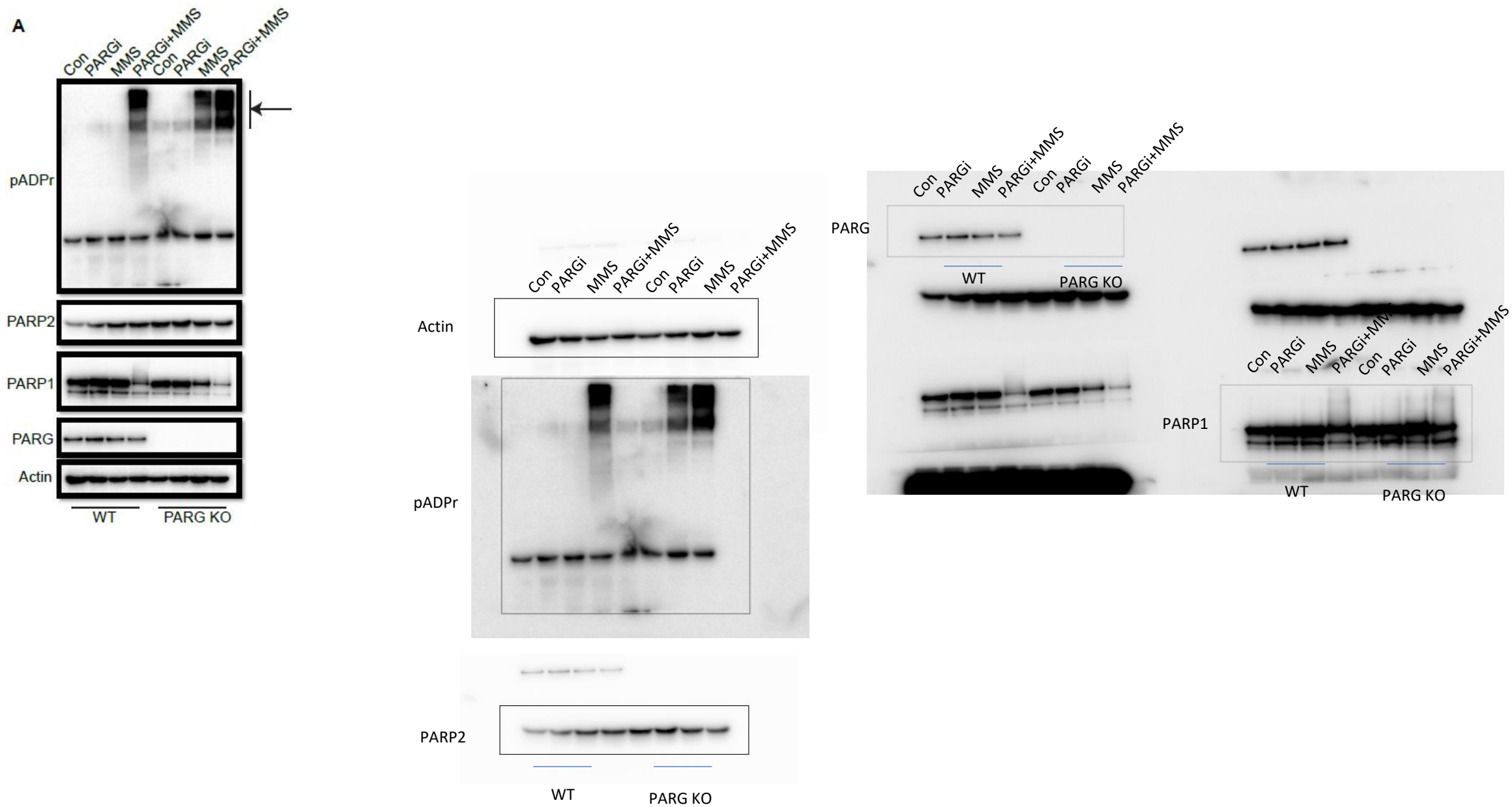

Figure 2-figure supplement 1

Supplement: Figure 2—figure supplement 1—source data 2. [file elife-89303-fig2-figsupp1-data2.zip › Figure 2-Figure Supplement 1-Source data 2/Figure 2-Figure Supplement 1-Source data 2.pdf]

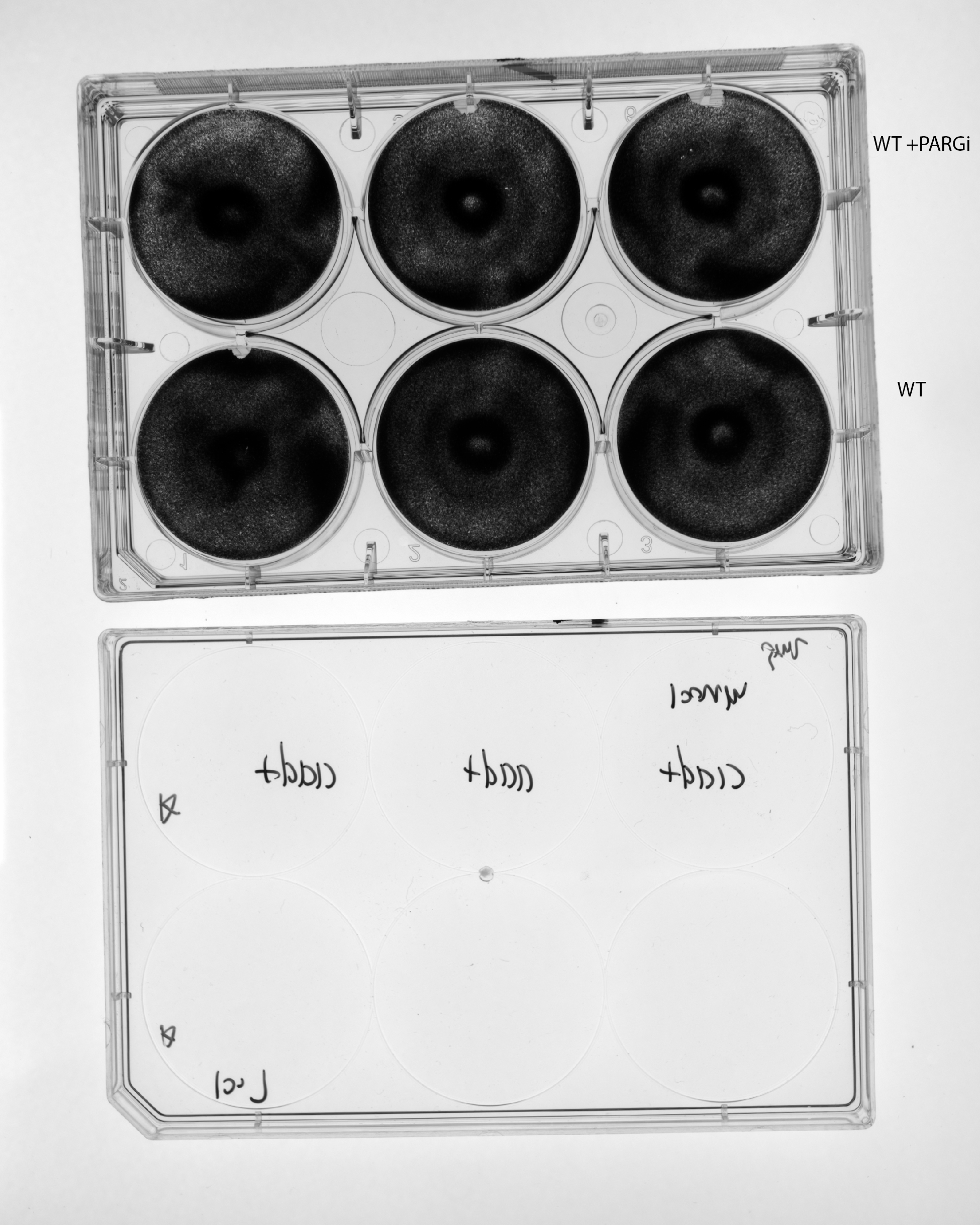

Supplement: Figure 2—figure supplement 1—source data 3. [file elife-89303-fig2-figsupp1-data3.zip › Figure 2-Figure Supplement 1-Source data 3/2E/WT.tif]

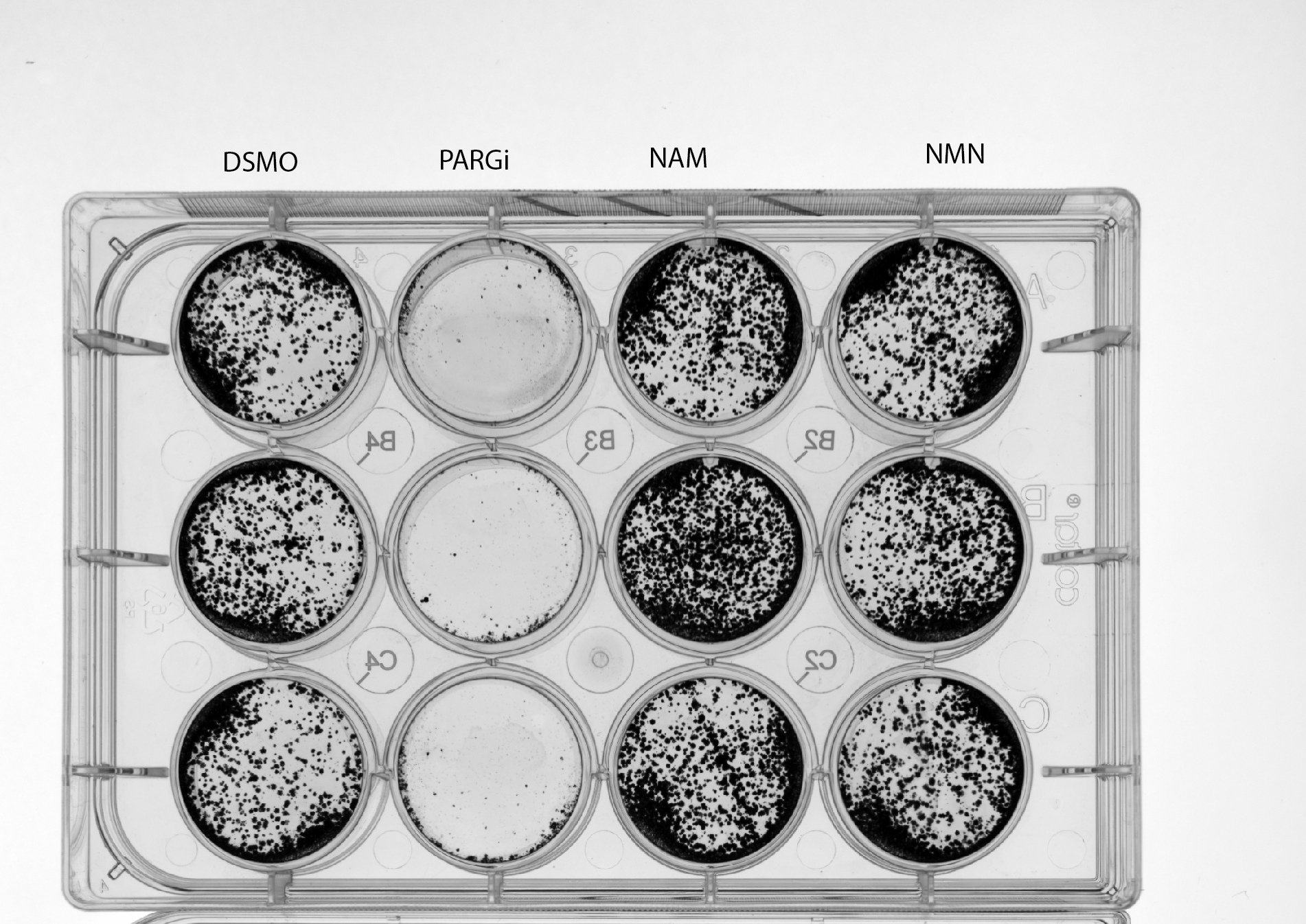

Supplement: Figure 2—figure supplement 1—source data 3. [file elife-89303-fig2-figsupp1-data3.zip › Figure 2-Figure Supplement 1-Source data 3/2F/Hela PARG KO-1.tif]

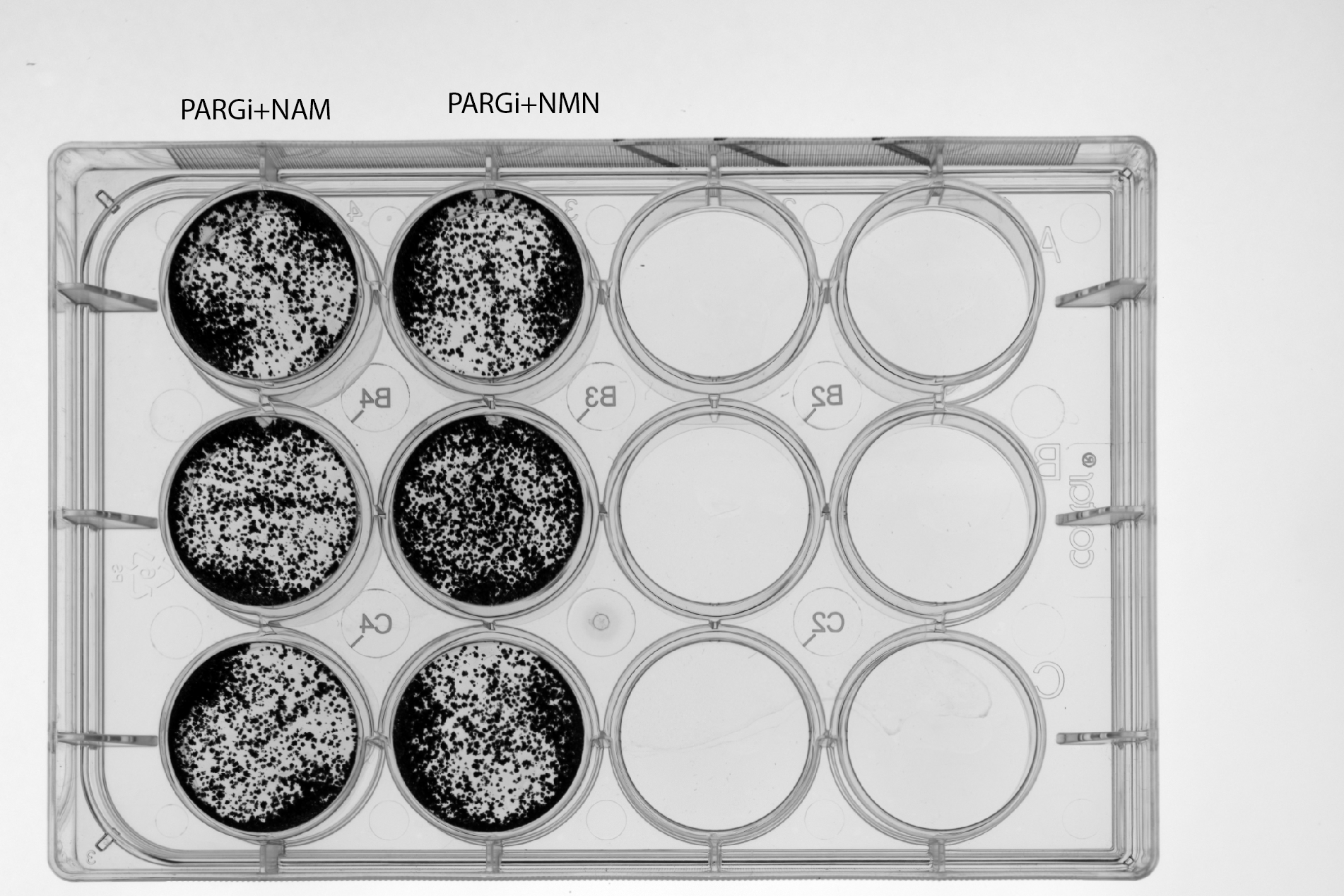

Supplement: Figure 2—figure supplement 1—source data 3. [file elife-89303-fig2-figsupp1-data3.zip › Figure 2-Figure Supplement 1-Source data 3/2F/Hela PARG Ko-2.tif]

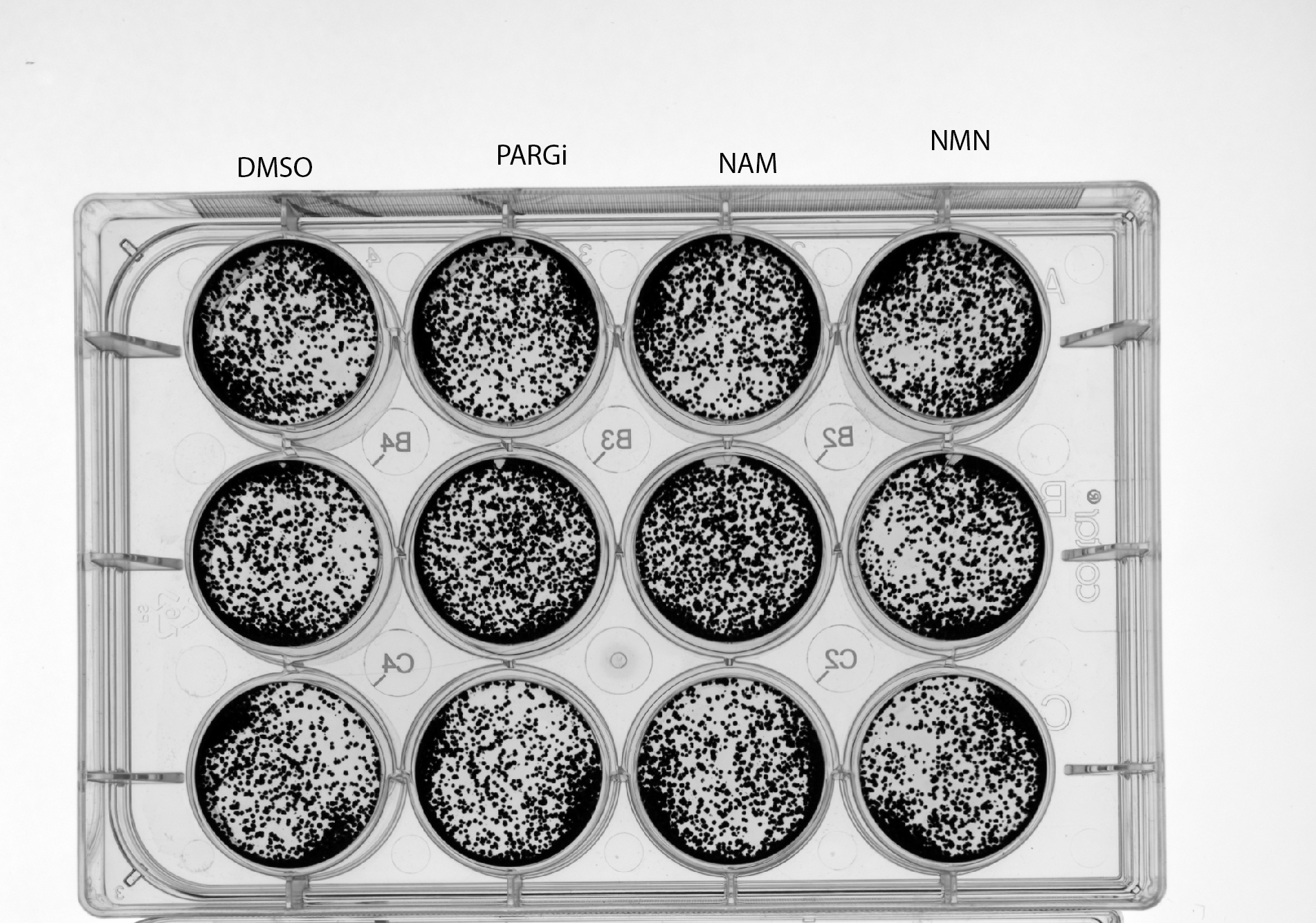

Supplement: Figure 2—figure supplement 1—source data 3. [file elife-89303-fig2-figsupp1-data3.zip › Figure 2-Figure Supplement 1-Source data 3/2F/hela-1.tif]

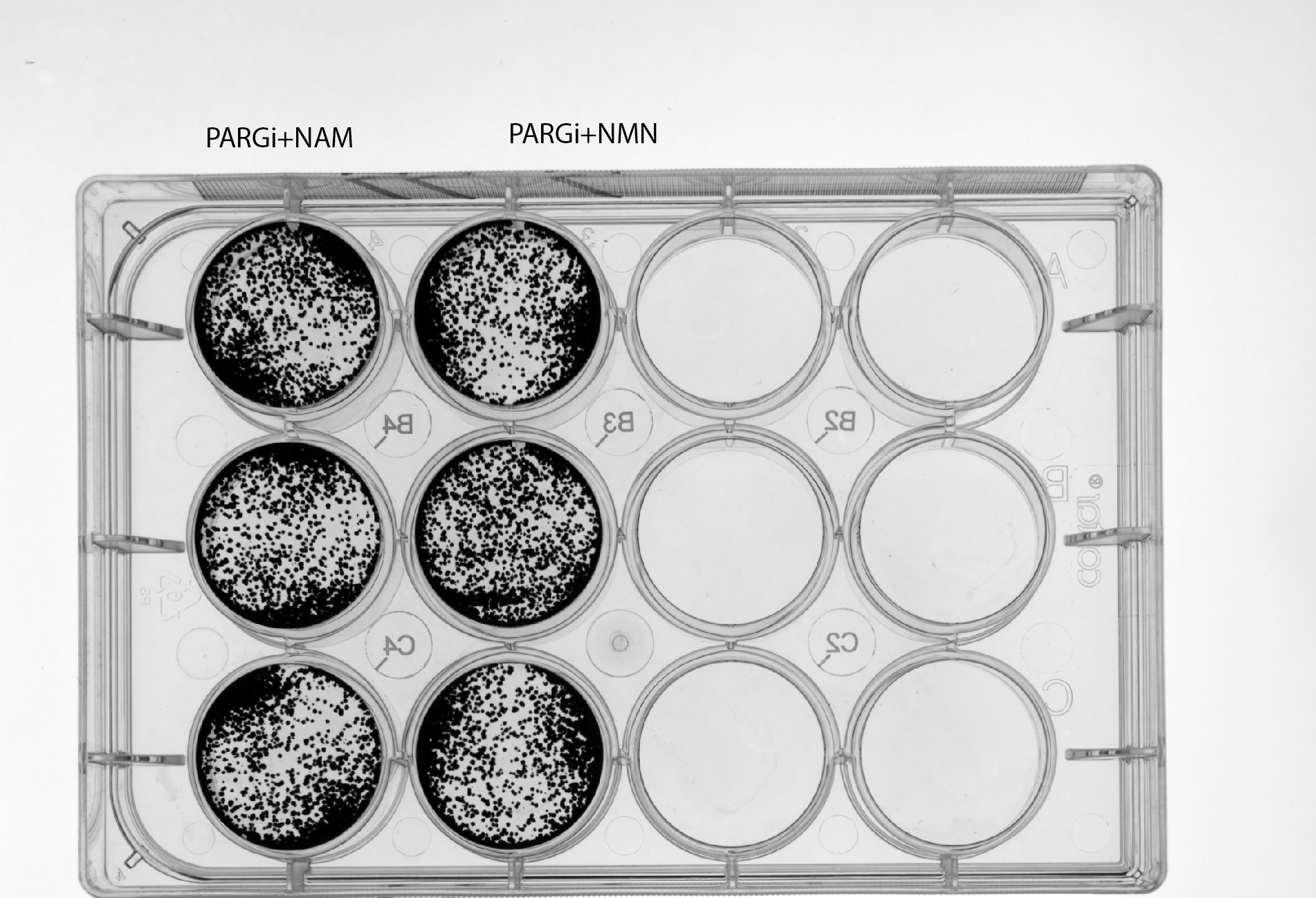

Supplement: Figure 2—figure supplement 1—source data 3. [file elife-89303-fig2-figsupp1-data3.zip › Figure 2-Figure Supplement 1-Source data 3/2F/Hela-2.tif]

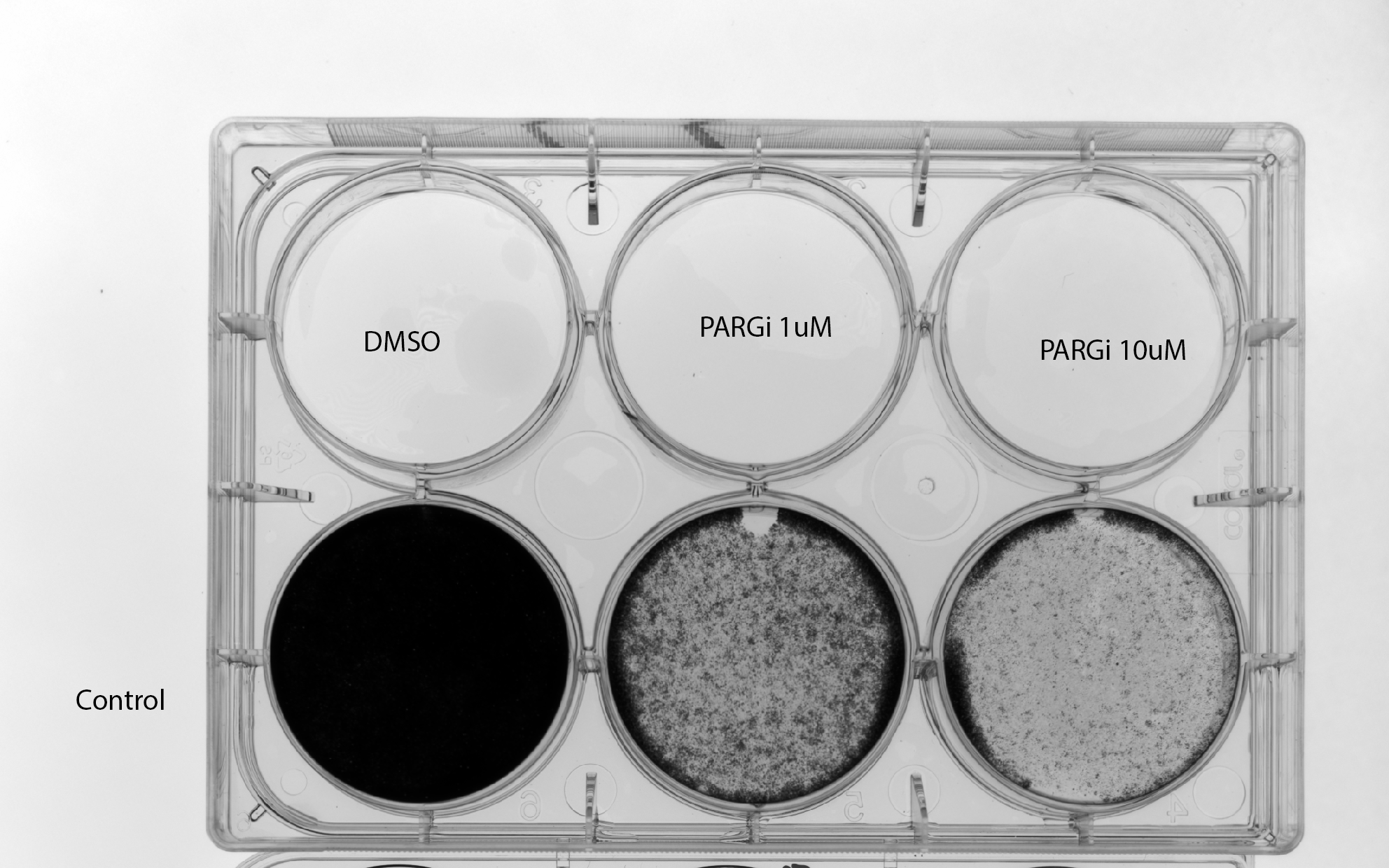

Supplement: Figure 3—source data 1. [file elife-89303-fig3-data1.zip › Figure 3-Source data 1/3D/1.tif]

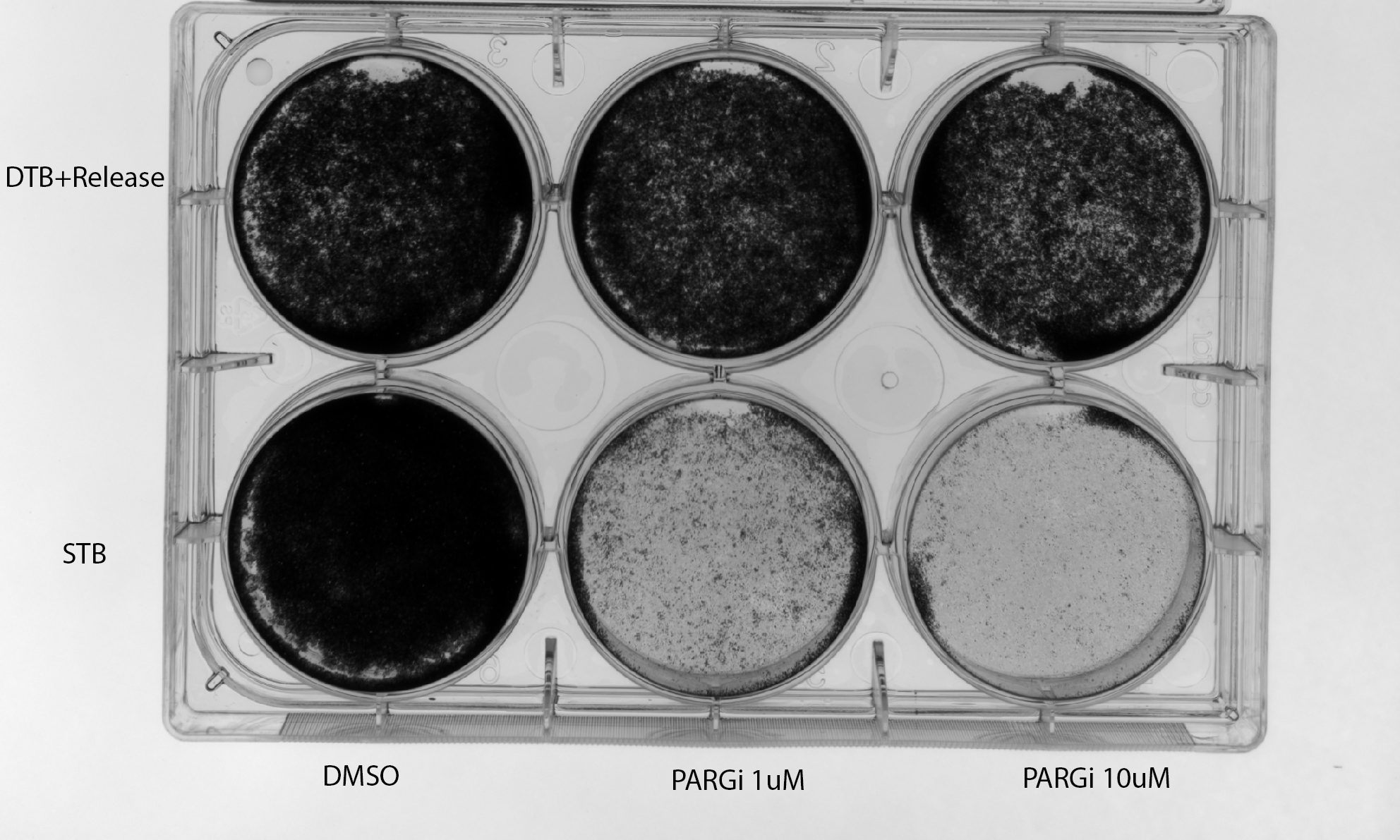

Supplement: Figure 3—source data 1. [file elife-89303-fig3-data1.zip › Figure 3-Source data 1/3D/2.tif]

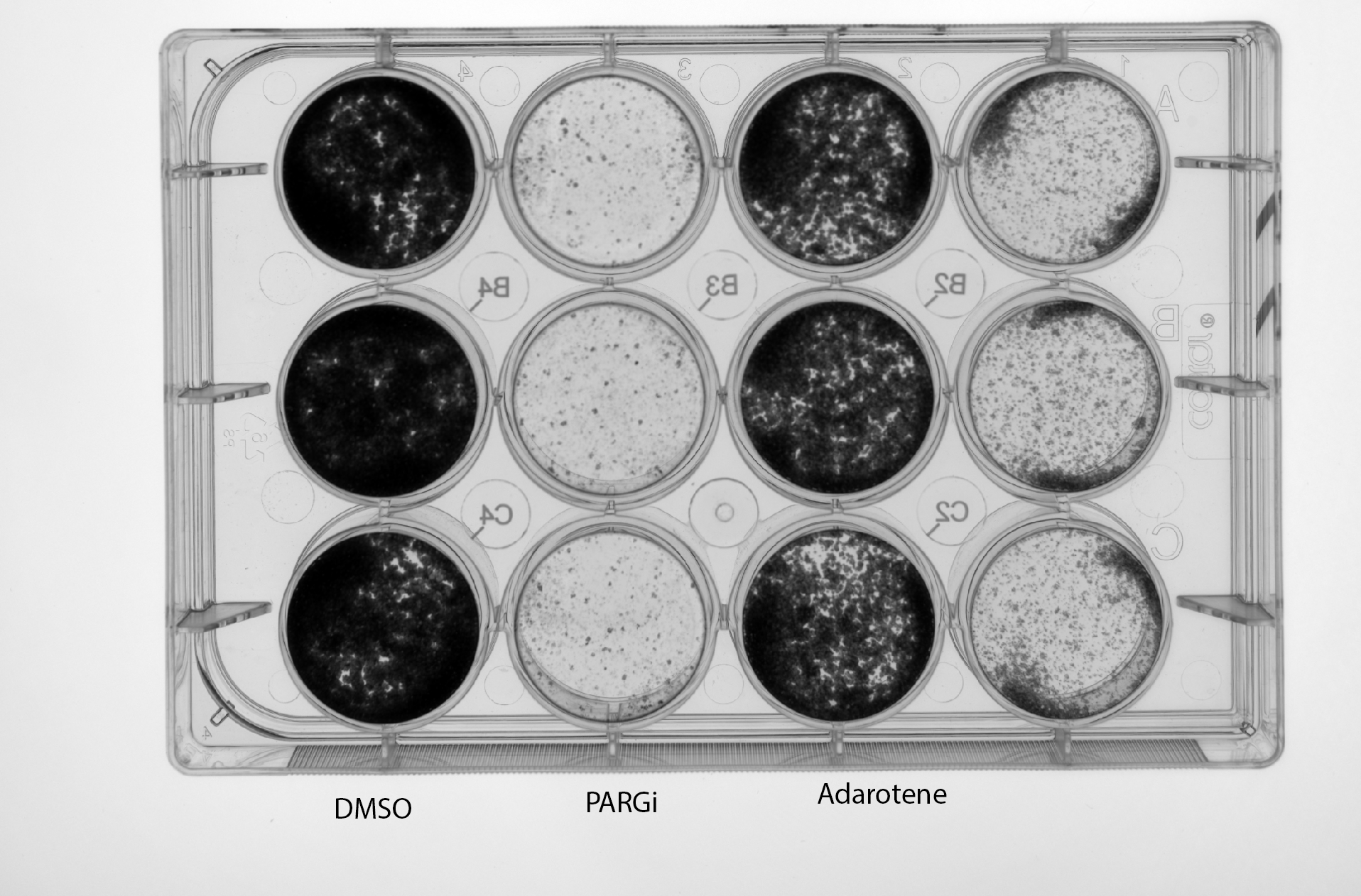

Supplement: Figure 3—source data 1. [file elife-89303-fig3-data1.zip › Figure 3-Source data 1/3E/1.tif]

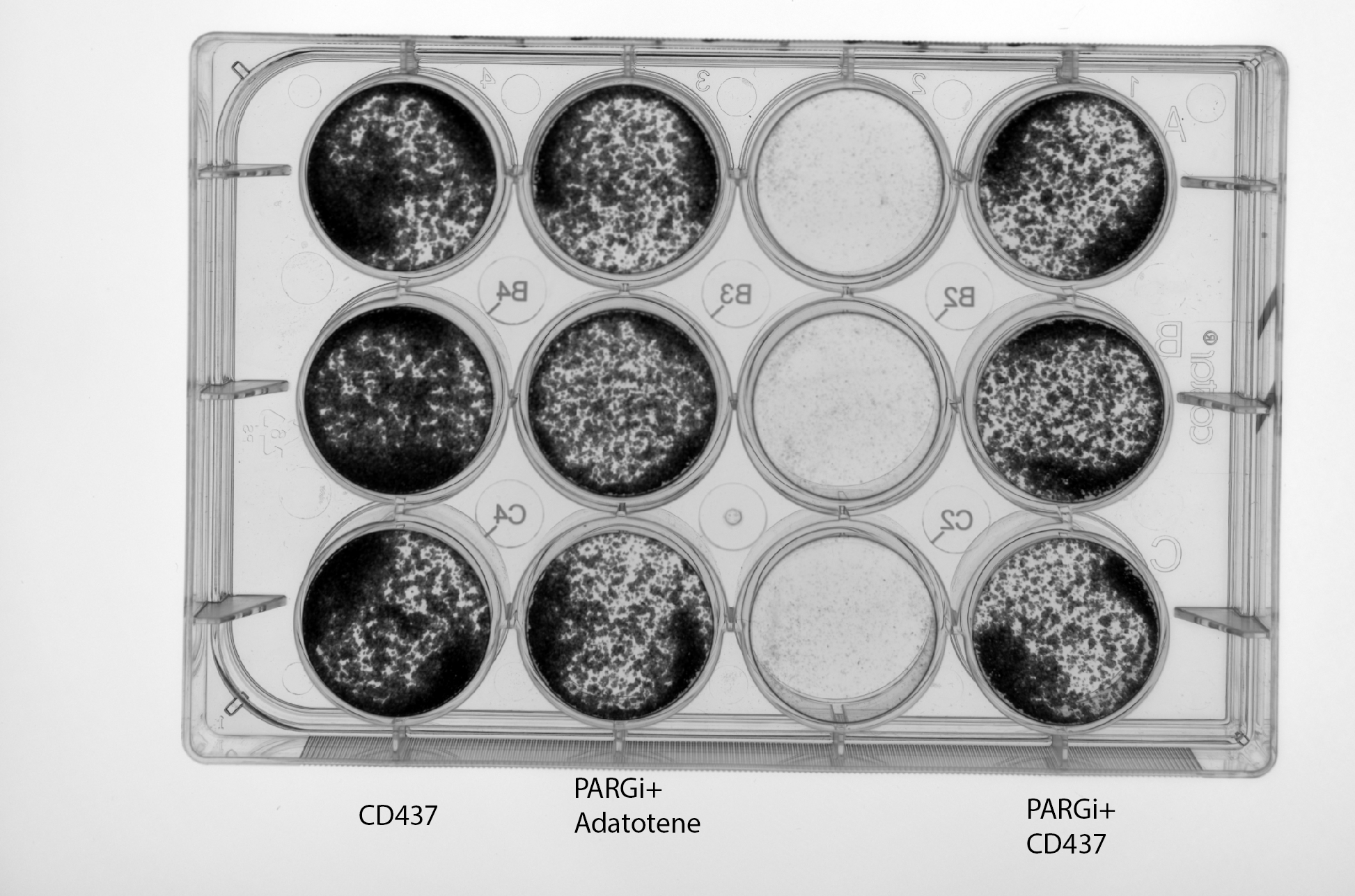

Supplement: Figure 3—source data 1. [file elife-89303-fig3-data1.zip › Figure 3-Source data 1/3E/2.tif]

D

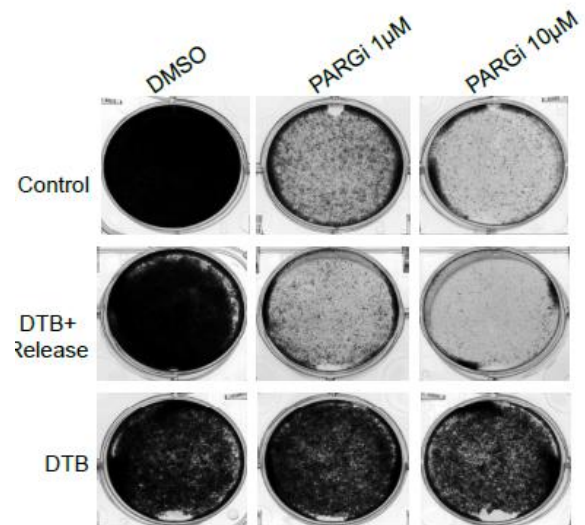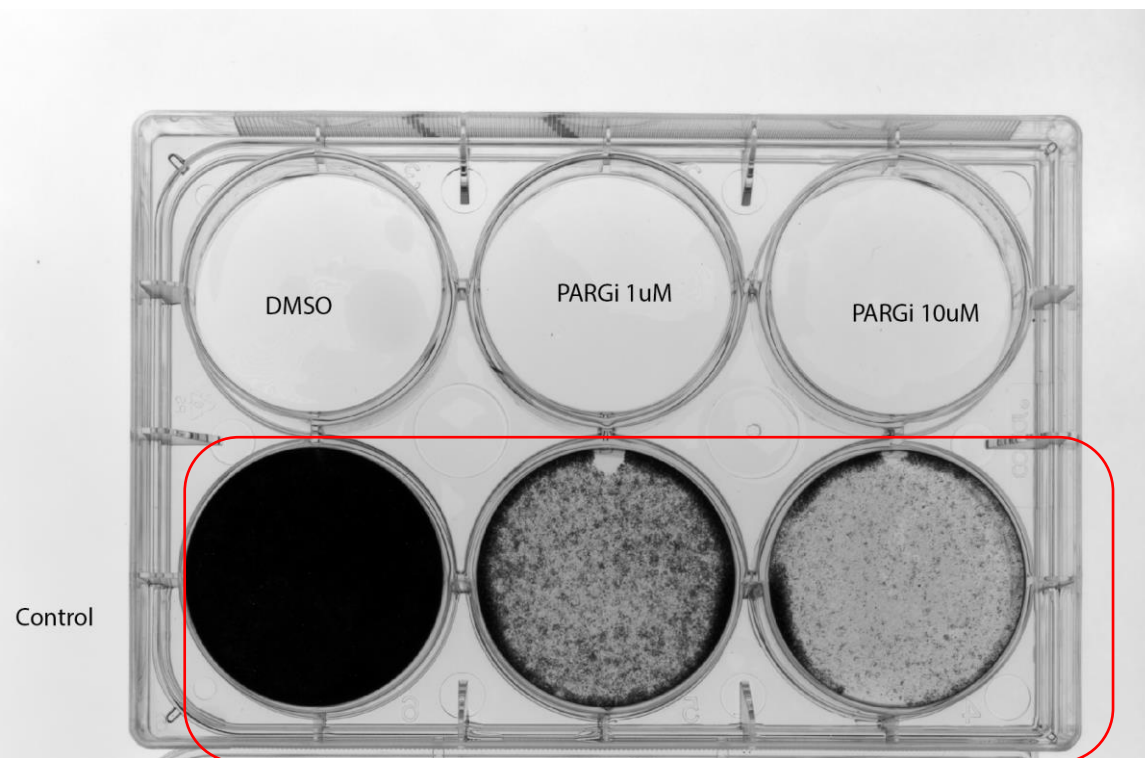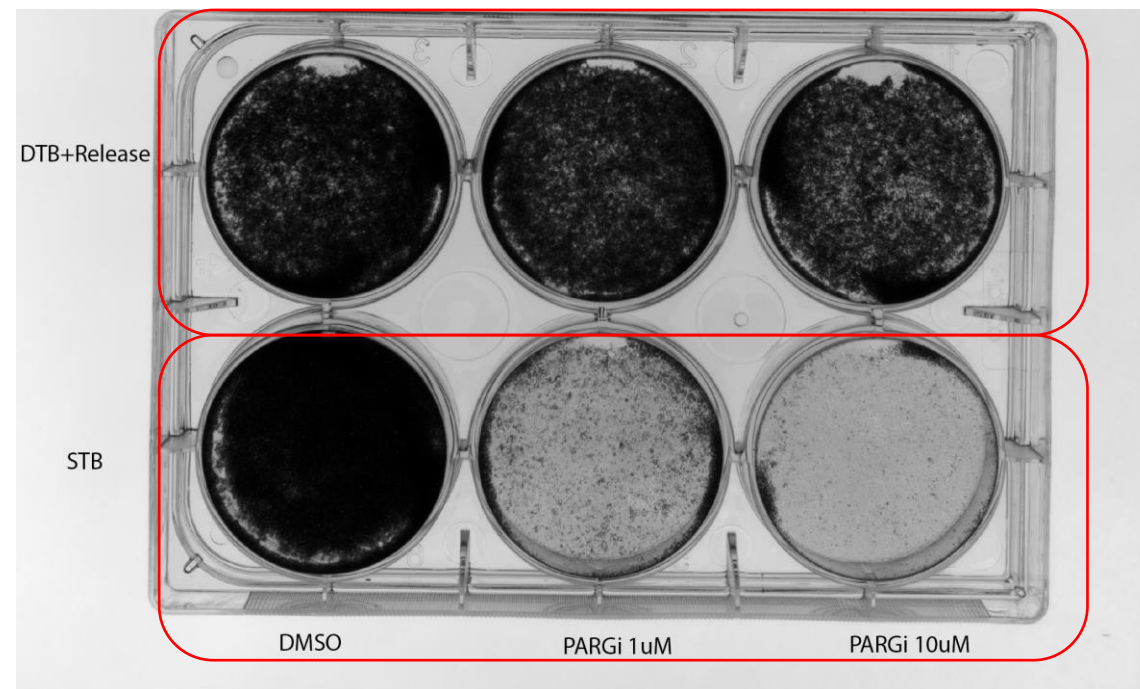

Figure 3

**E**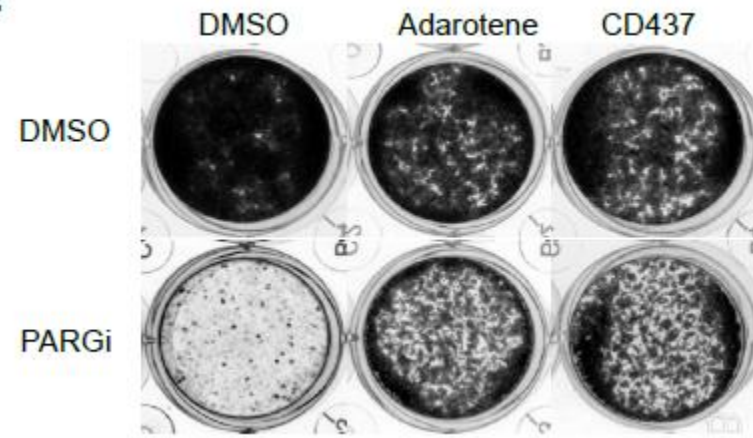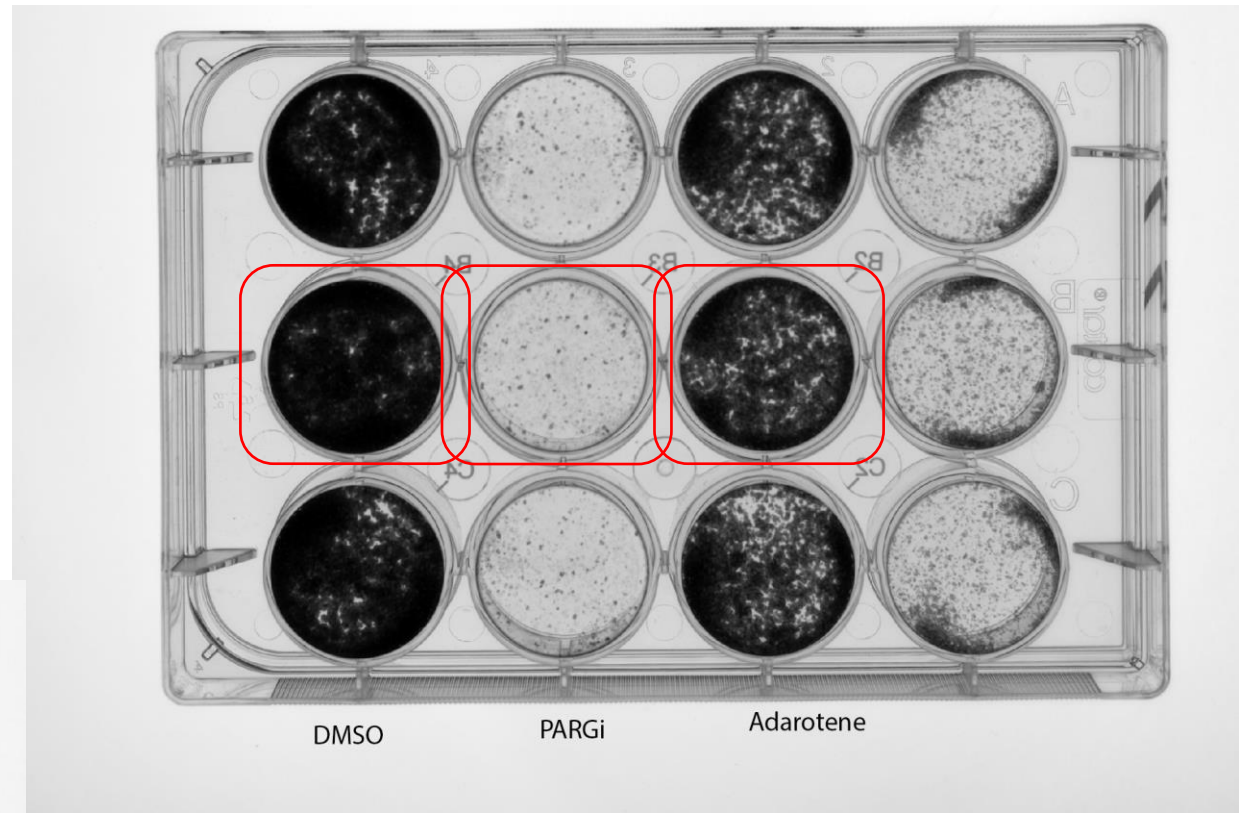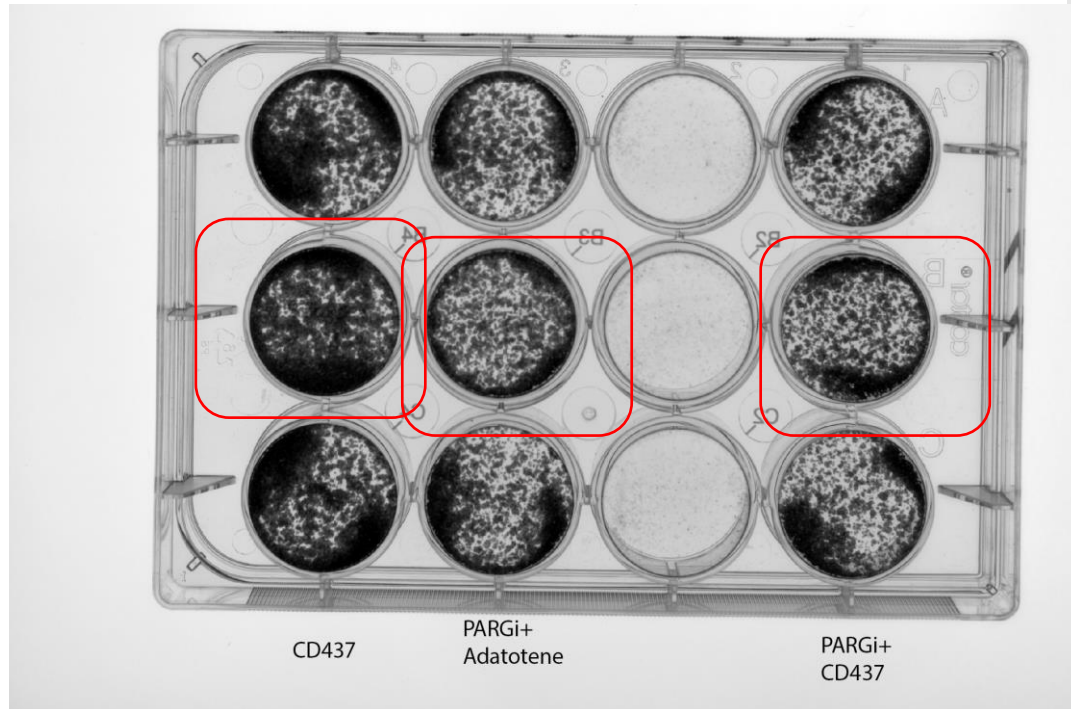

Figure 3

Supplement: Figure 3—source data 2. [file elife-89303-fig3-data2.zip › Figure 3-Source data 2/Figure 3-Source data 2.pdf]

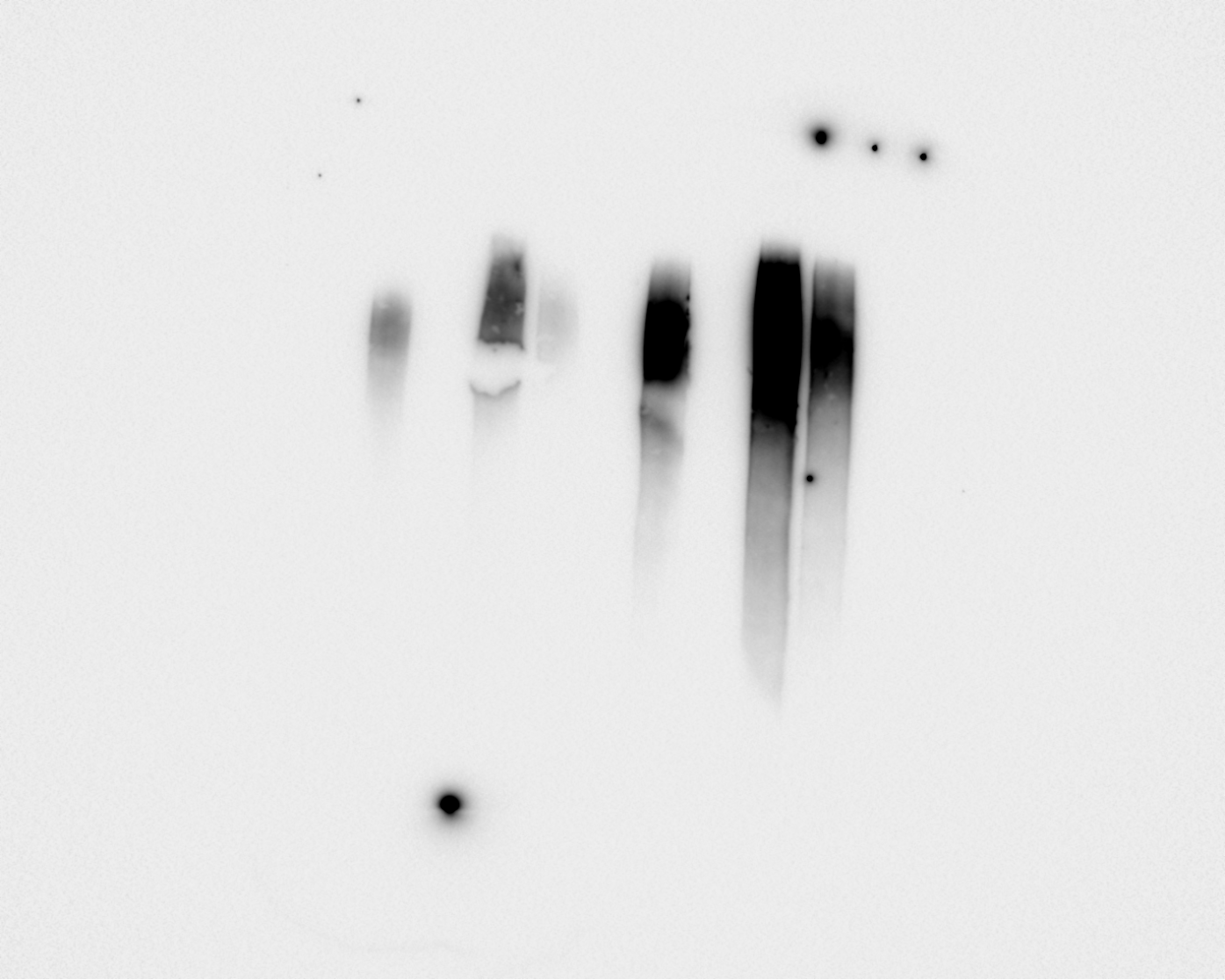

Supplement: Figure 4—source data 1. [file elife-89303-fig4-data1.zip › Figure 4-Source data 1/4A/Chro_pADPr.tif]

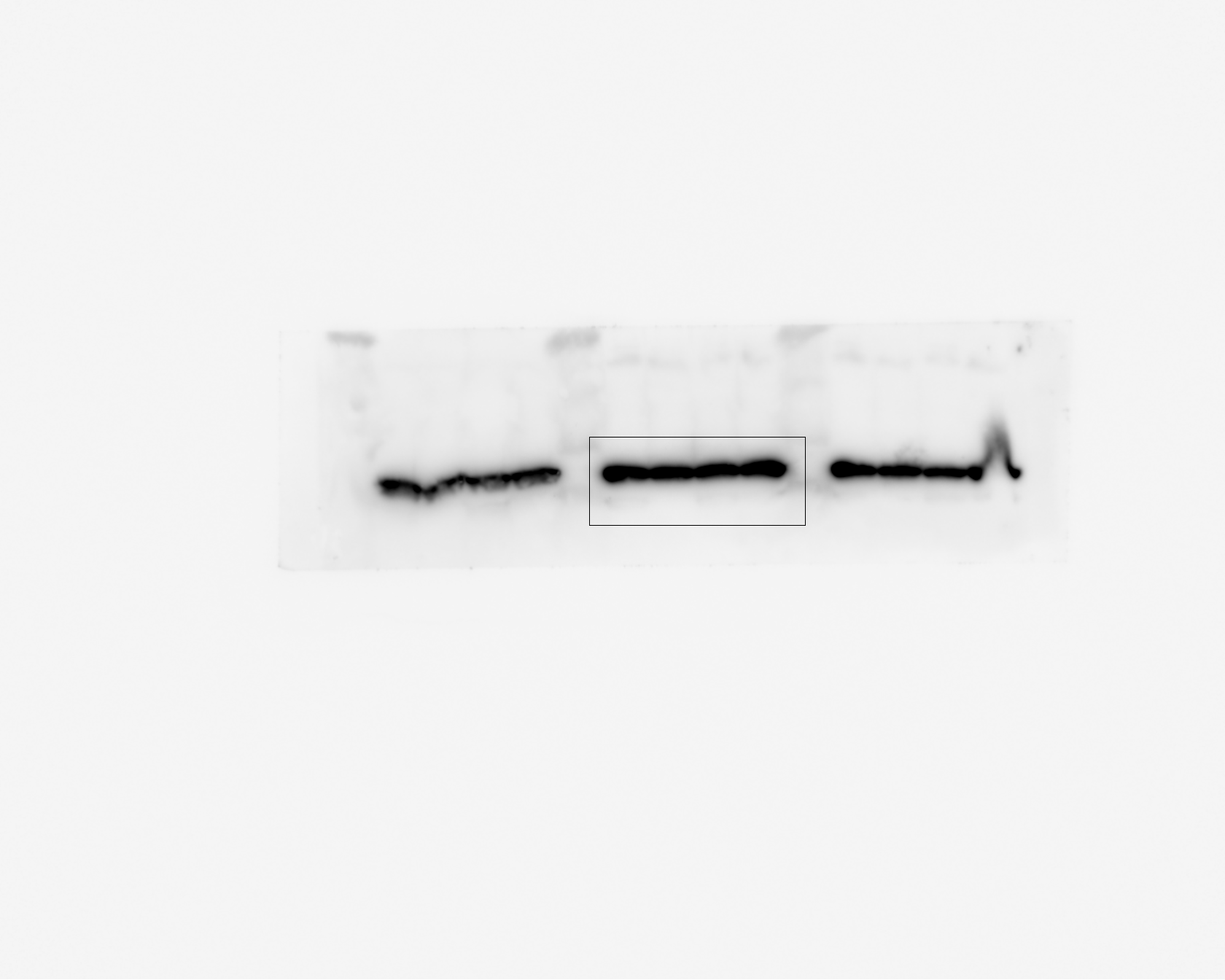

Supplement: Figure 4—source data 1. [file elife-89303-fig4-data1.zip › Figure 4-Source data 1/4A/H3.tif]

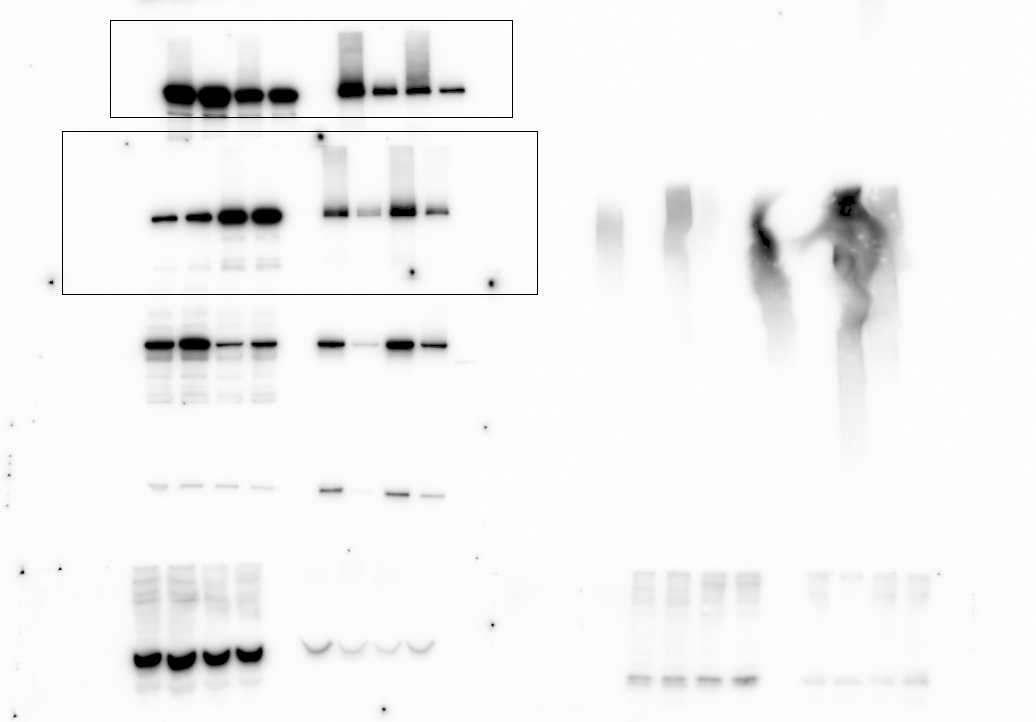

Supplement: Figure 4—source data 1. [file elife-89303-fig4-data1.zip › Figure 4-Source data 1/4A/SoL&Chr_PARP1.tif]

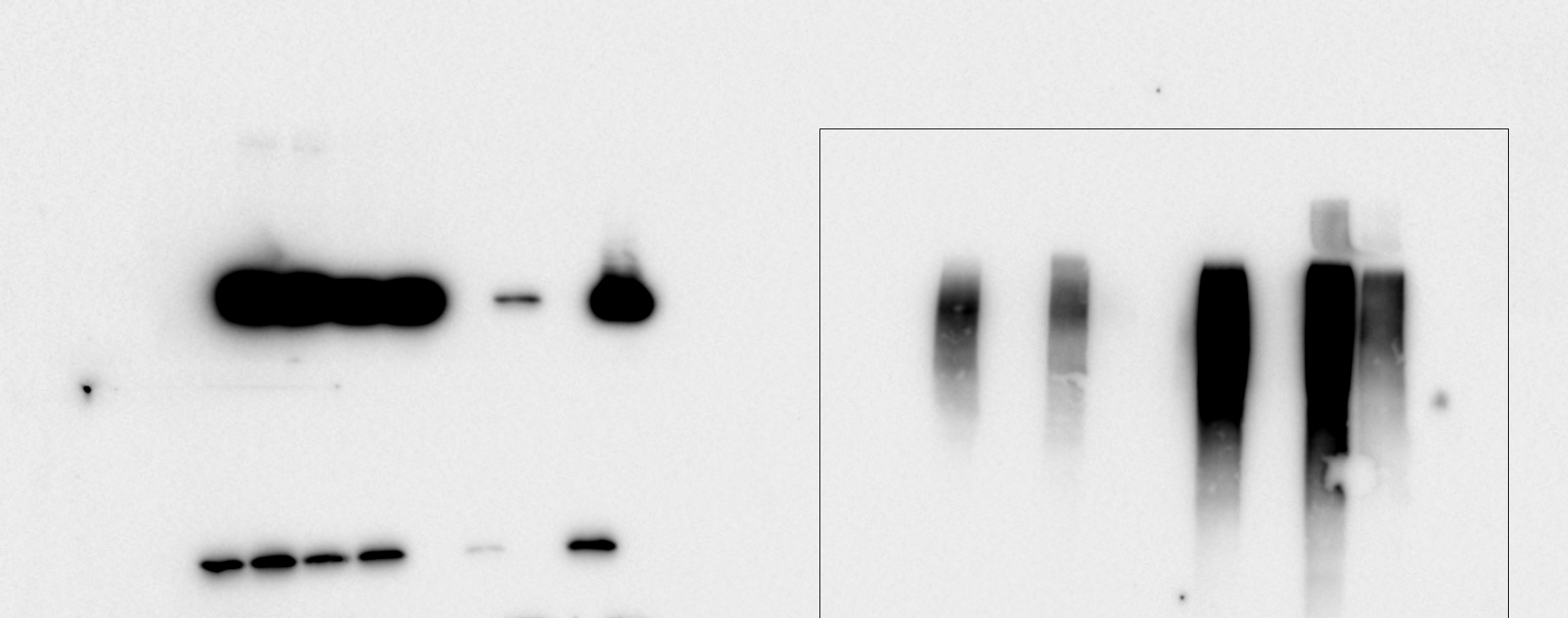

Supplement: Figure 4—source data 1. [file elife-89303-fig4-data1.zip › Figure 4-Source data 1/4A/Sol_pADPr.tif]

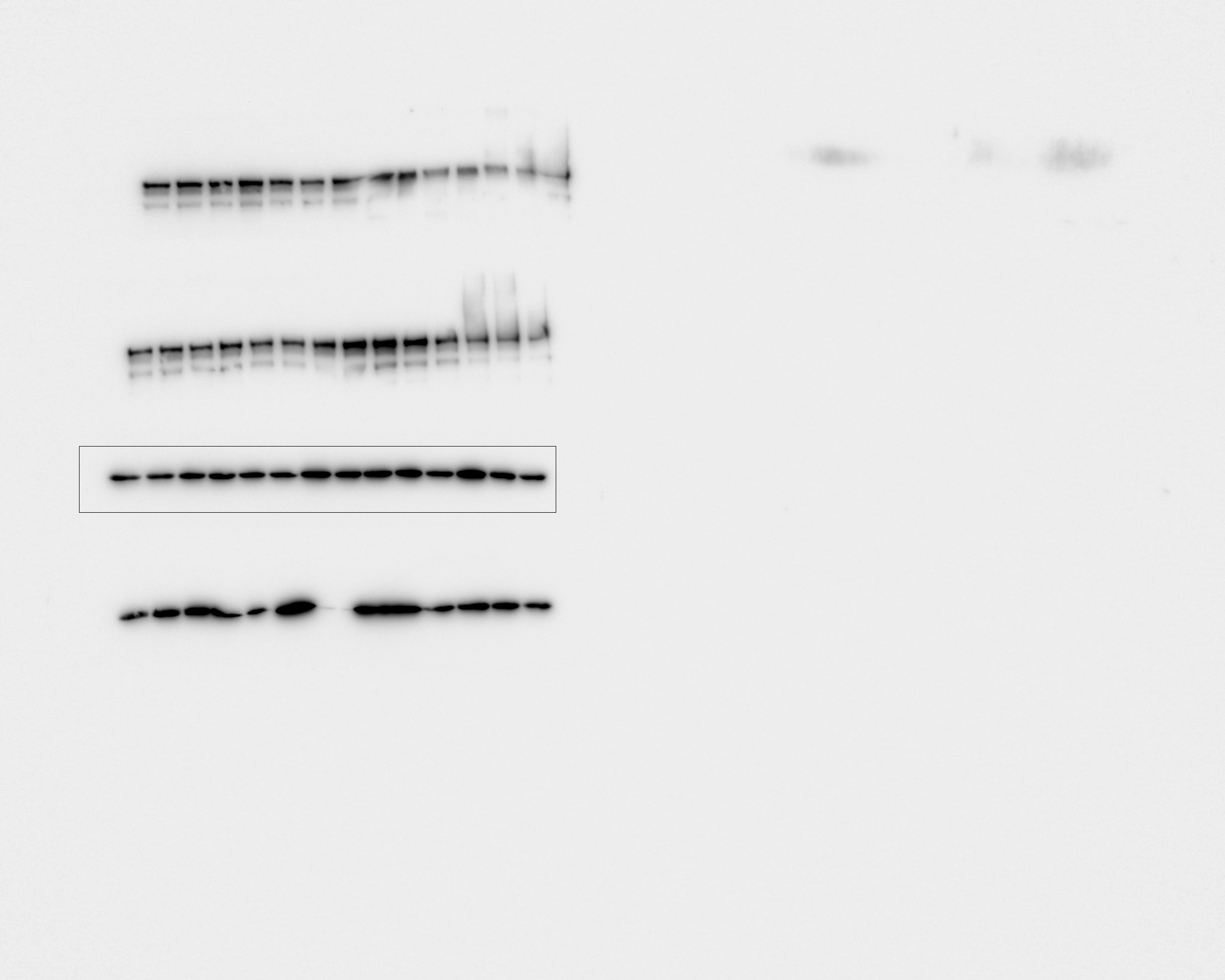

Supplement: Figure 4—source data 1. [file elife-89303-fig4-data1.zip › Figure 4-Source data 1/4D/actin.tif]

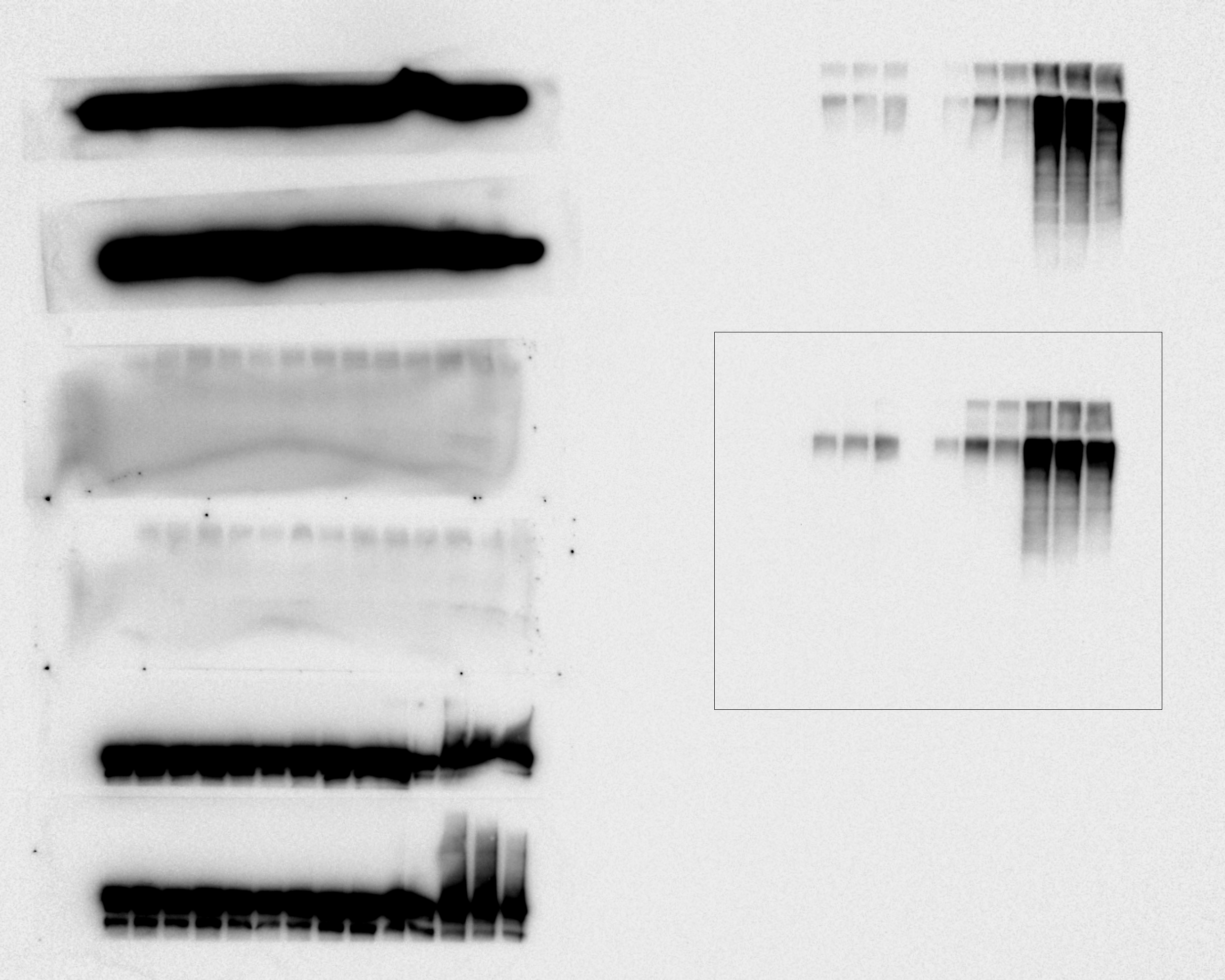

Supplement: Figure 4—source data 1. [file elife-89303-fig4-data1.zip › Figure 4-Source data 1/4D/pADPr.tif]

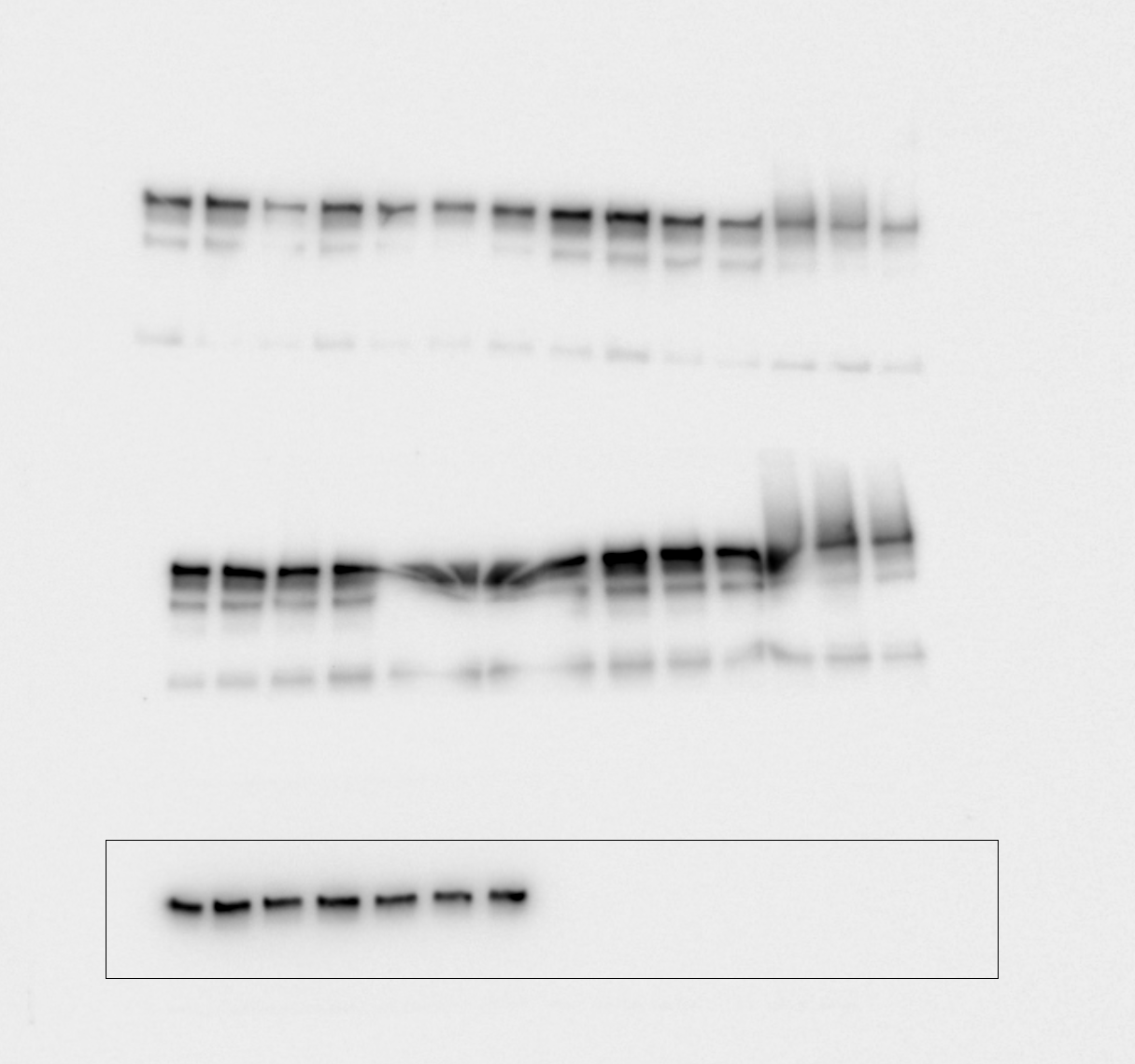

Supplement: Figure 4—source data 1. [file elife-89303-fig4-data1.zip › Figure 4-Source data 1/4D/PARG.tif]

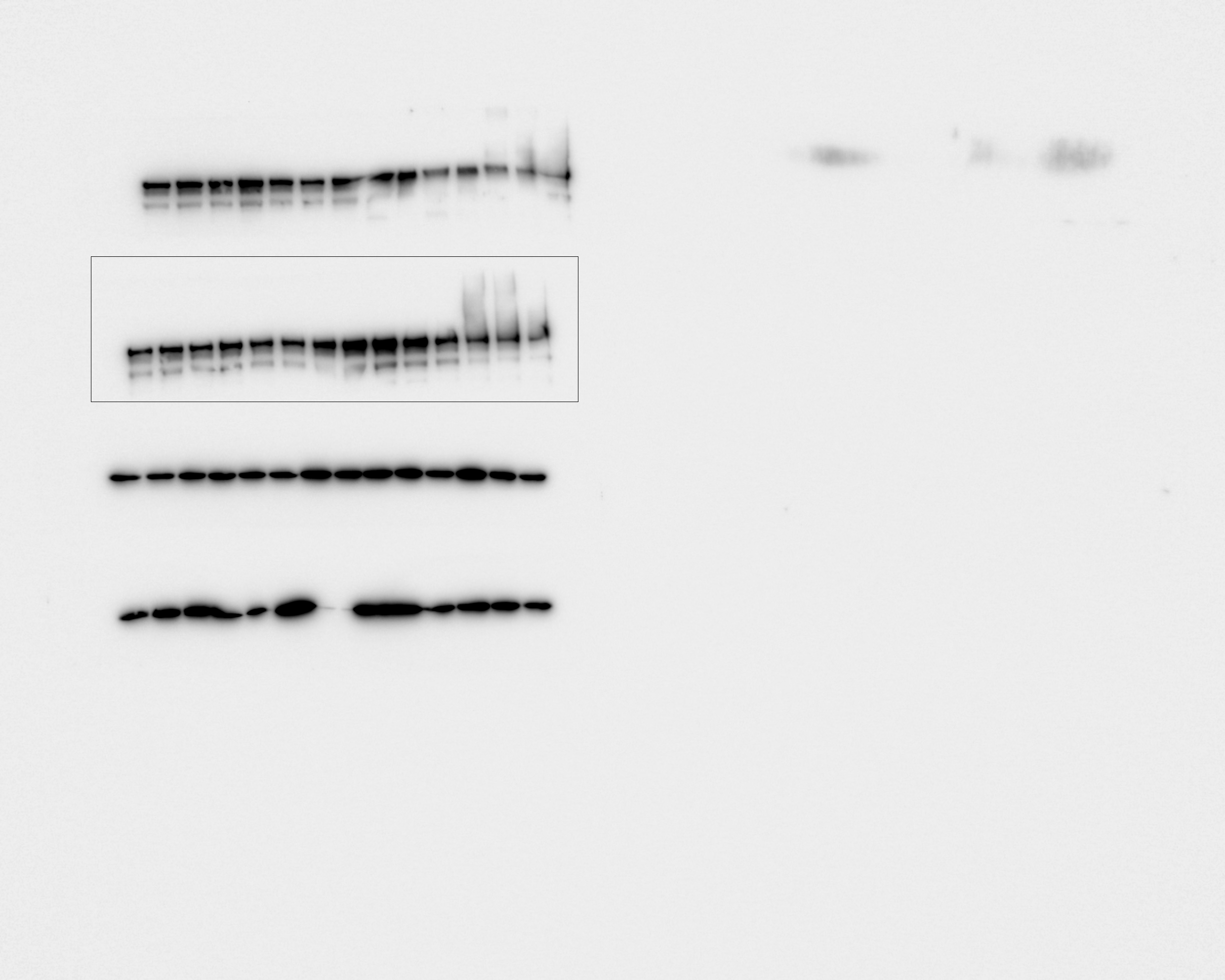

Supplement: Figure 4—source data 1. [file elife-89303-fig4-data1.zip › Figure 4-Source data 1/4D/PARP1.tif]

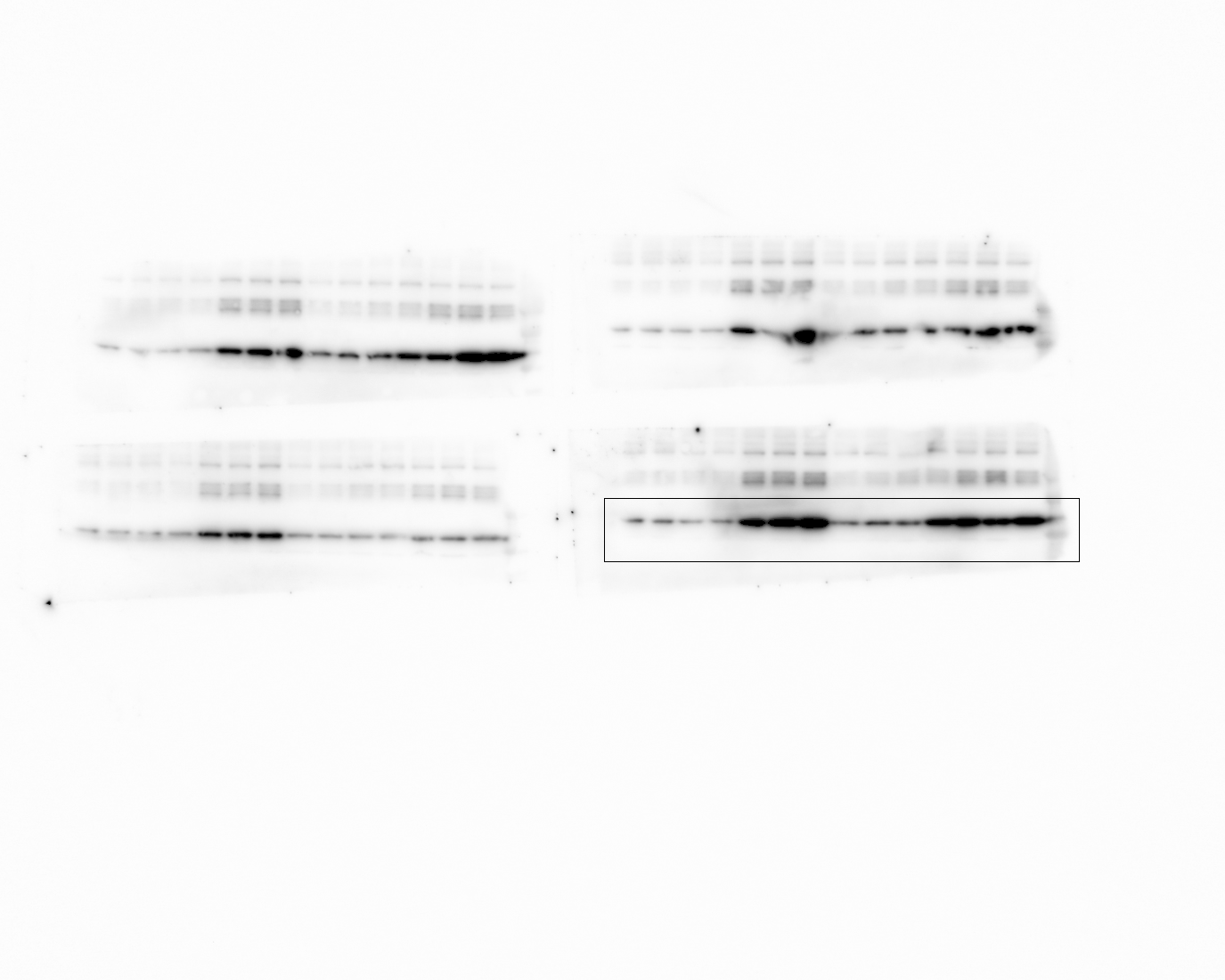

Supplement: Figure 4—source data 1. [file elife-89303-fig4-data1.zip › Figure 4-Source data 1/4D/rH2AX.tif]

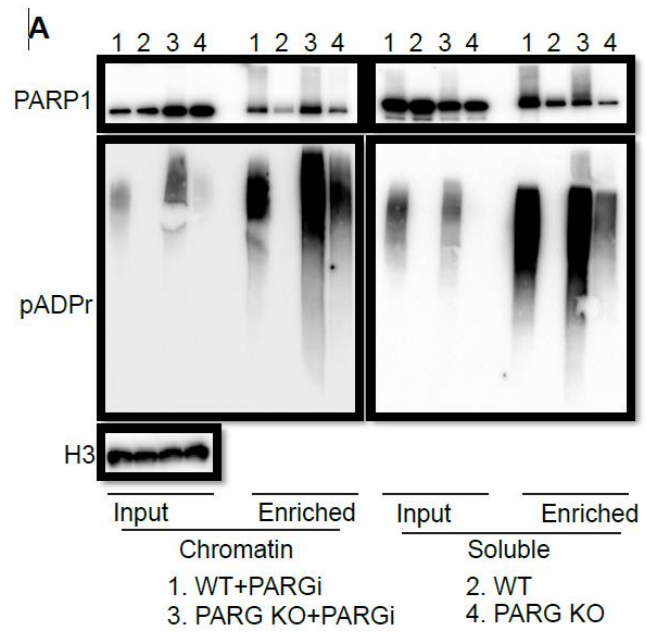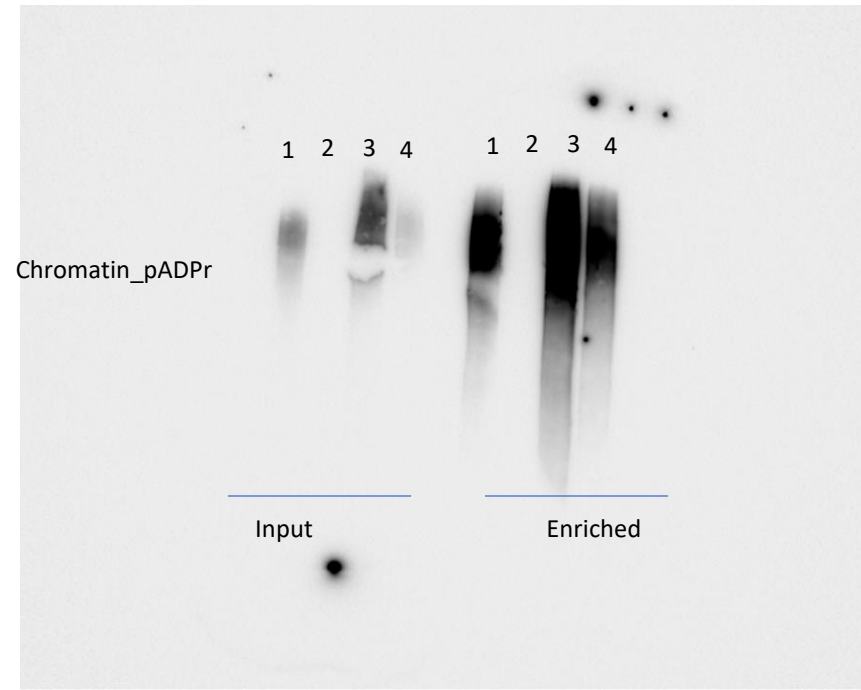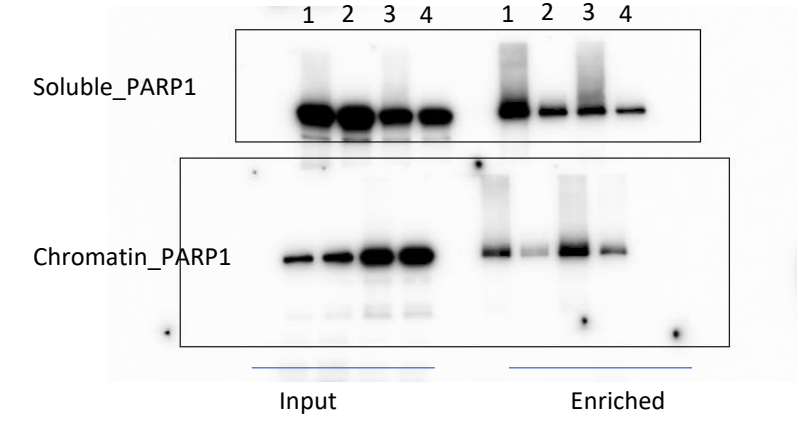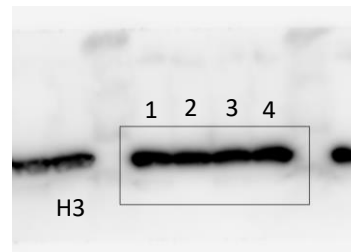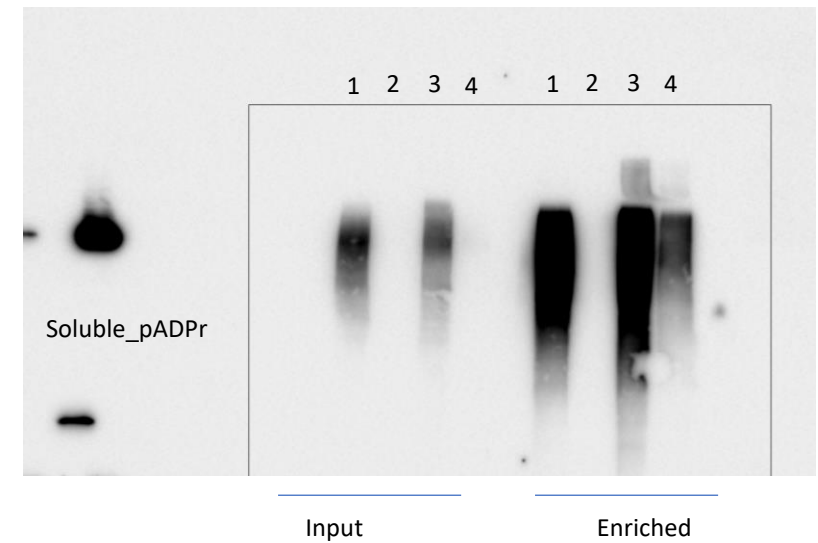

Figure 4

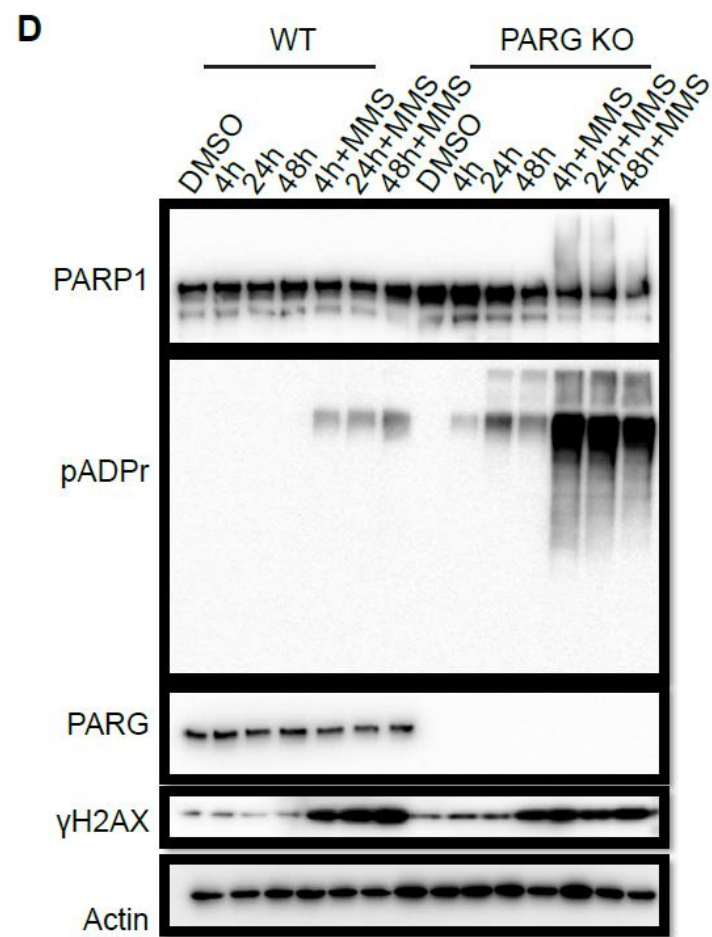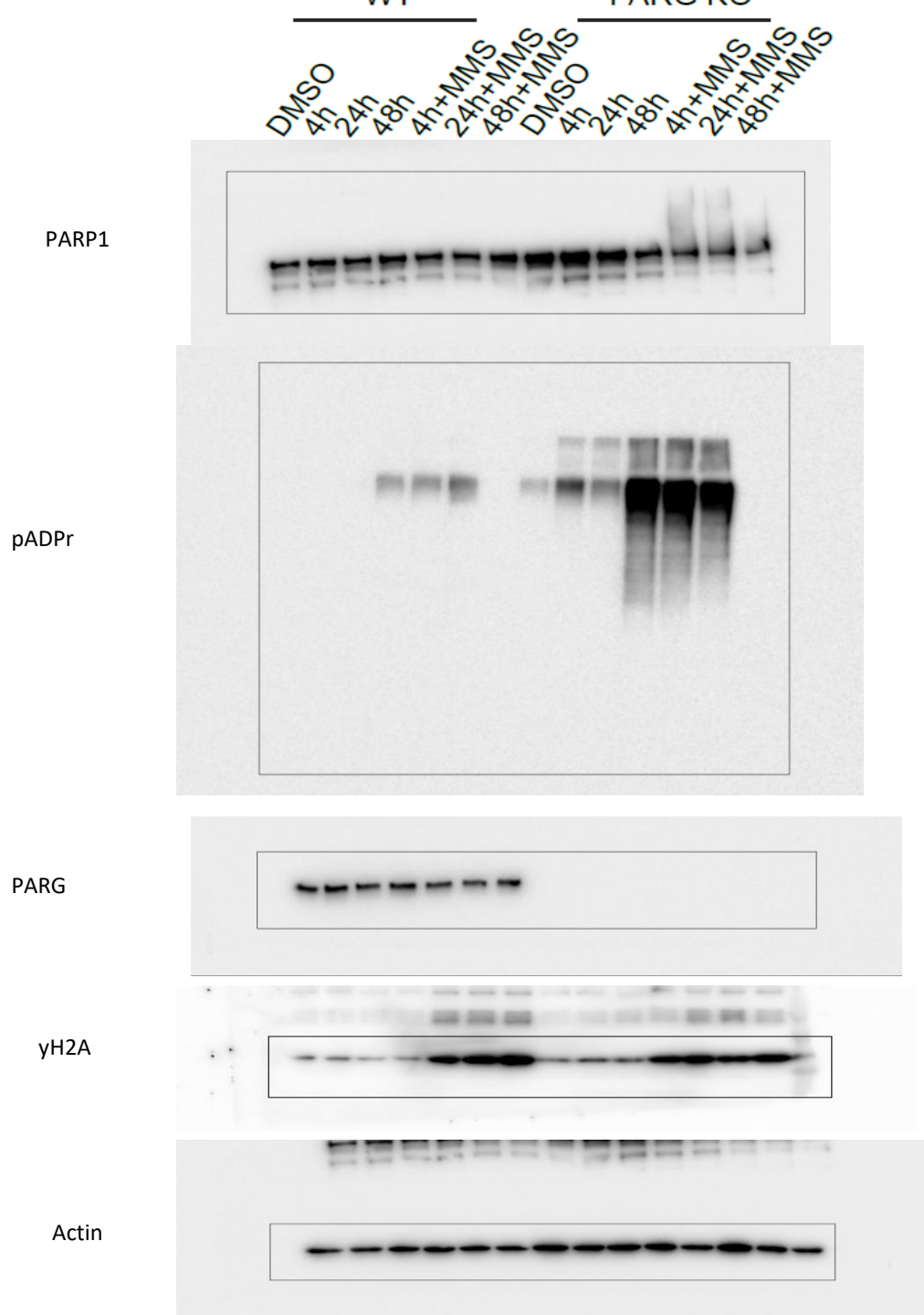

Figure 4

Supplement: Figure 4—source data 2. [file elife-89303-fig4-data2.zip › Figure 4-Source data 2/Figure 4-Source data 2.pdf]

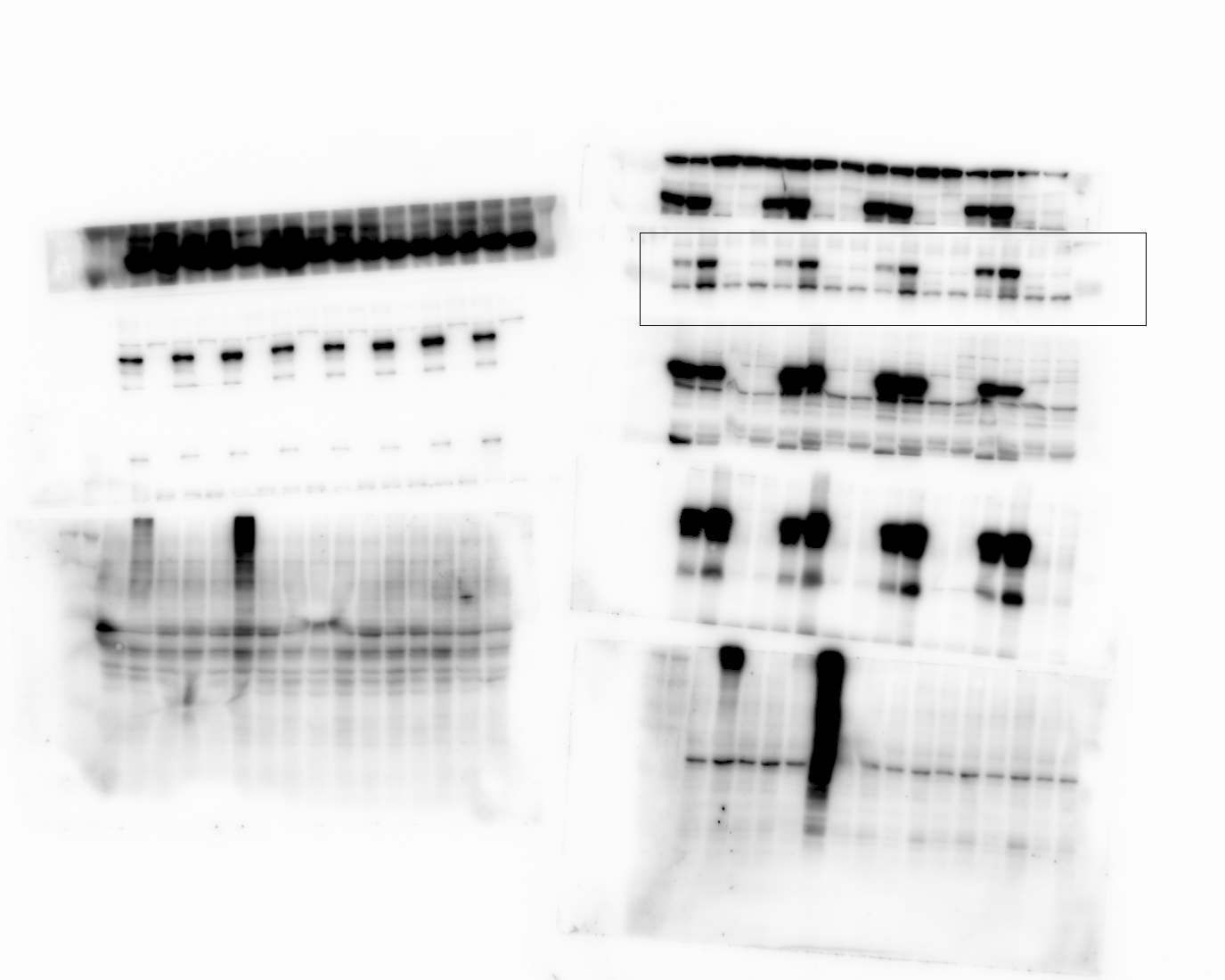

Supplement: Figure 4—figure supplement 1—source data 1. [file elife-89303-fig4-figsupp1-data1.zip › Figure 4-Figure Supplement 1-Source data 1/S4A/Chr_PARP2.tif]

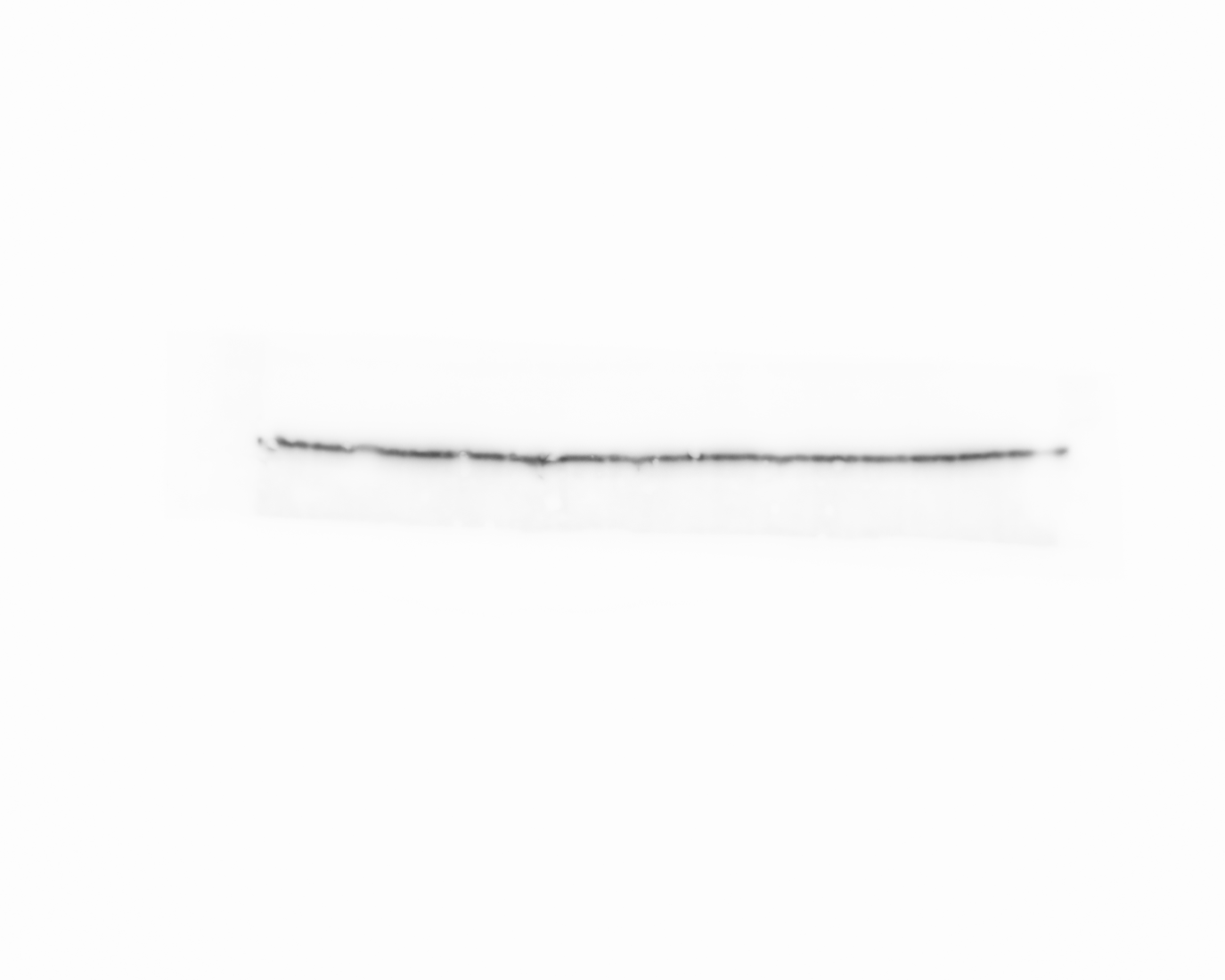

Supplement: Figure 4—figure supplement 1—source data 1. [file elife-89303-fig4-figsupp1-data1.zip › Figure 4-Figure Supplement 1-Source data 1/S4A/H3.tif]

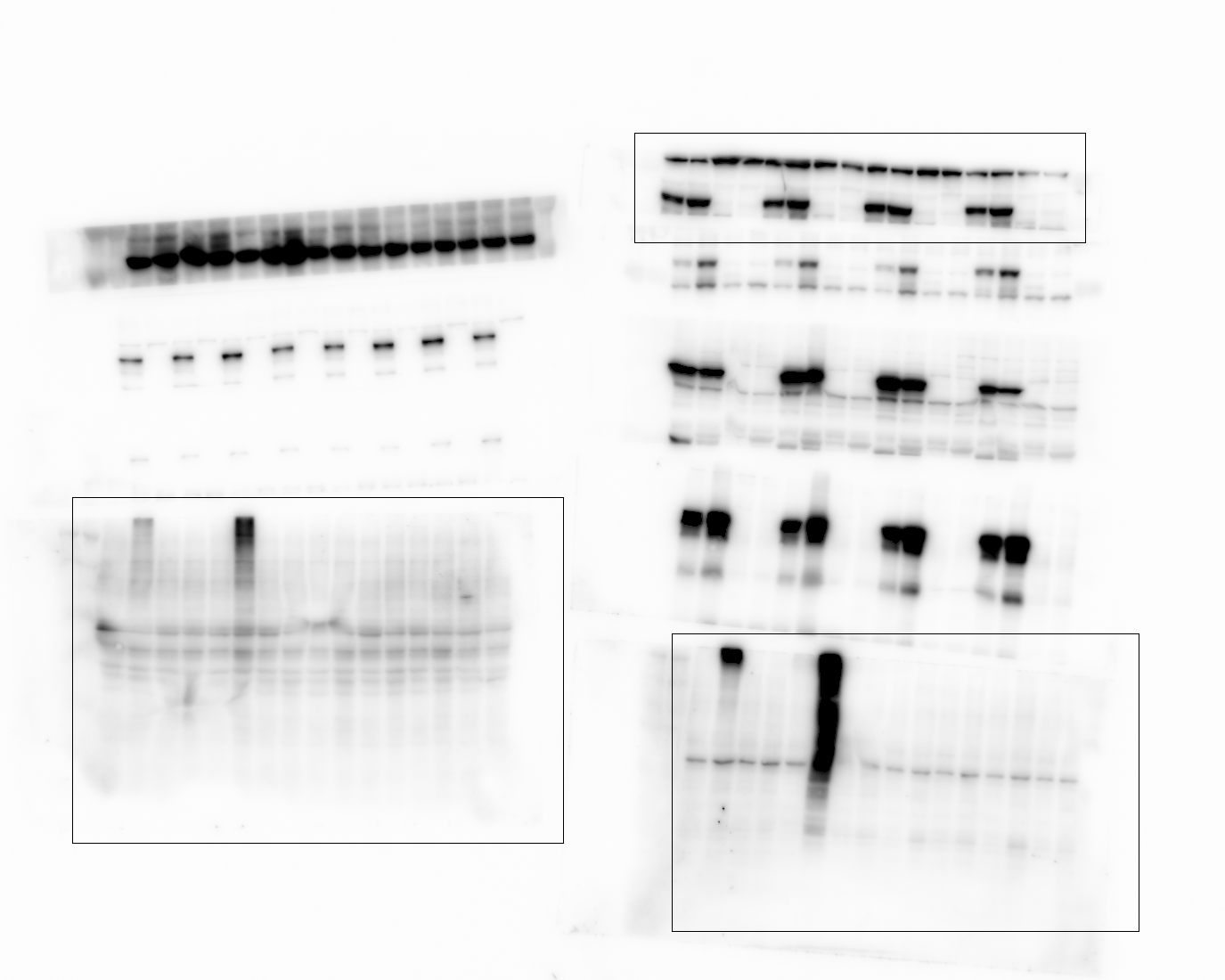

Supplement: Figure 4—figure supplement 1—source data 1. [file elife-89303-fig4-figsupp1-data1.zip › Figure 4-Figure Supplement 1-Source data 1/S4A/pADPr&Sol_PARP2.tif]

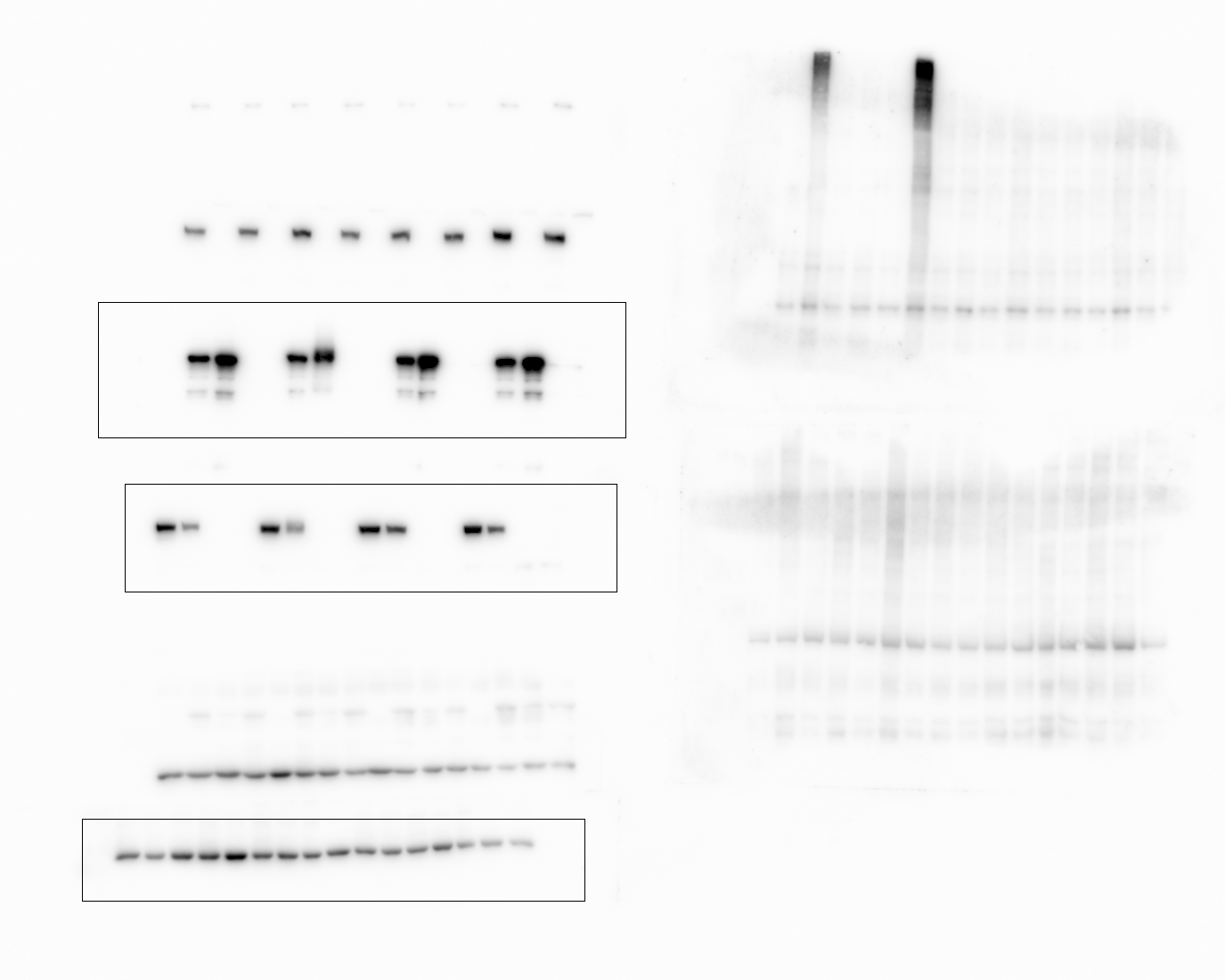

Supplement: Figure 4—figure supplement 1—source data 1. [file elife-89303-fig4-figsupp1-data1.zip › Figure 4-Figure Supplement 1-Source data 1/S4A/PARP1&actin.tif]

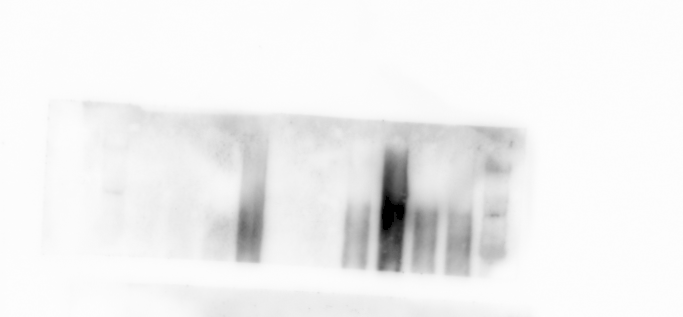

Supplement: Figure 4—figure supplement 1—source data 1. [file elife-89303-fig4-figsupp1-data1.zip › Figure 4-Figure Supplement 1-Source data 1/S4B/enriched_PARP1.tif]

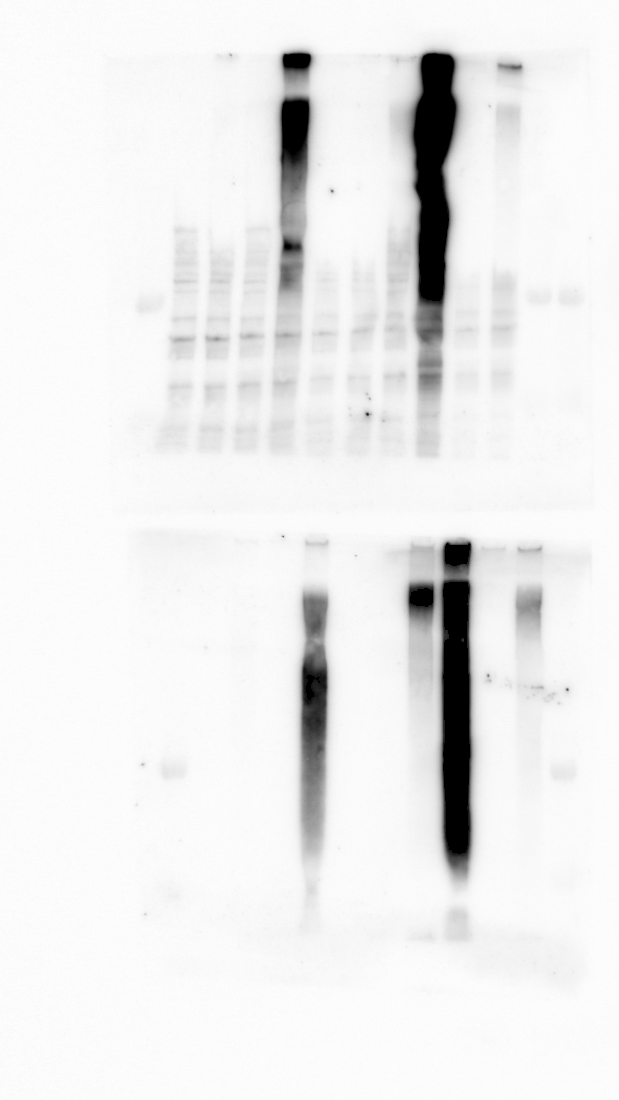

Supplement: Figure 4—figure supplement 1—source data 1. [file elife-89303-fig4-figsupp1-data1.zip › Figure 4-Figure Supplement 1-Source data 1/S4B/input&enriched_pADPr.tif]

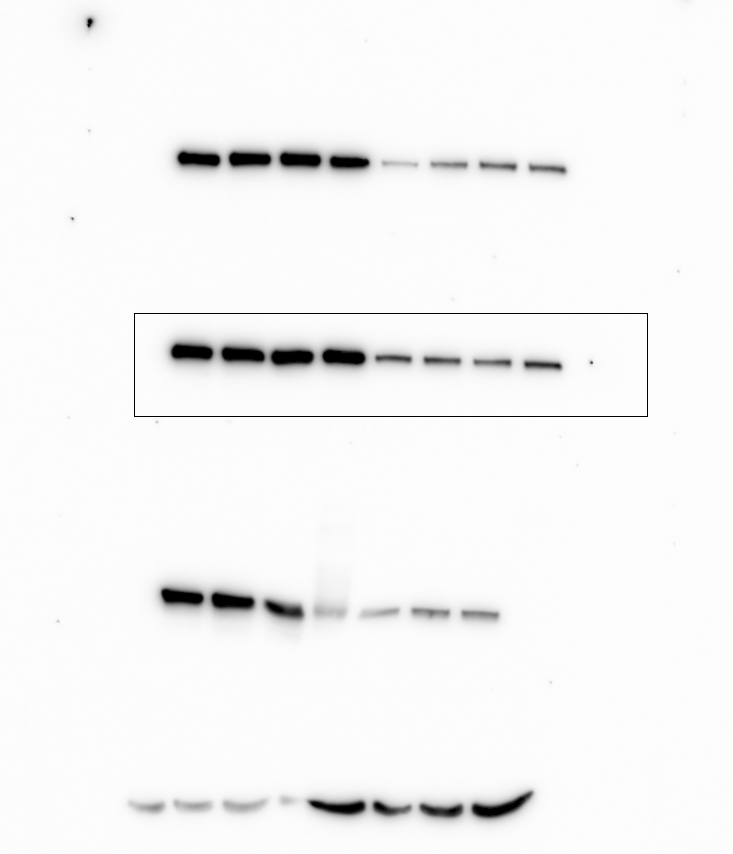

Supplement: Figure 4—figure supplement 1—source data 1. [file elife-89303-fig4-figsupp1-data1.zip › Figure 4-Figure Supplement 1-Source data 1/S4E/PARG.tif]

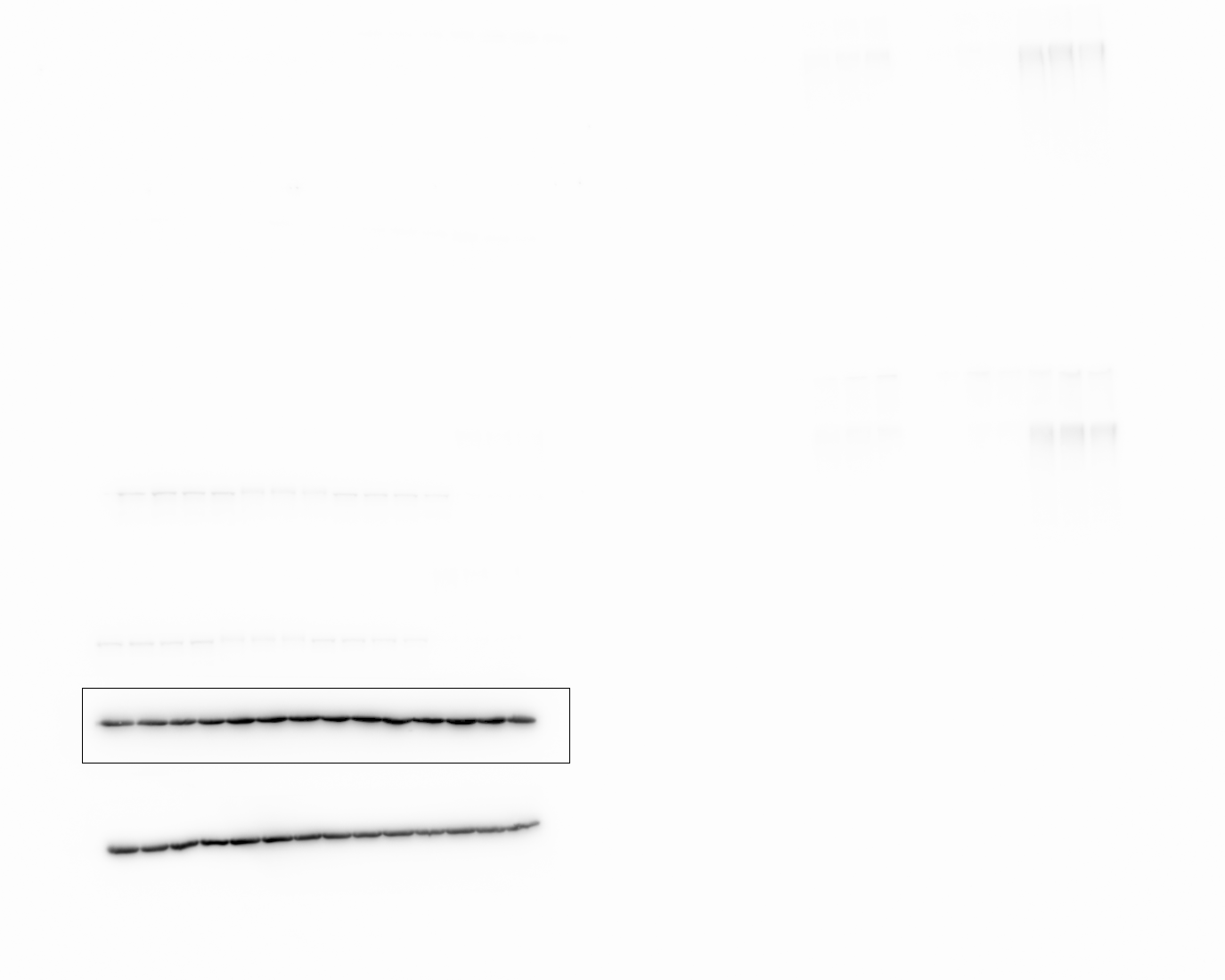

Supplement: Figure 4—figure supplement 1—source data 1. [file elife-89303-fig4-figsupp1-data1.zip › Figure 4-Figure Supplement 1-Source data 1/S4F/actin.tif]

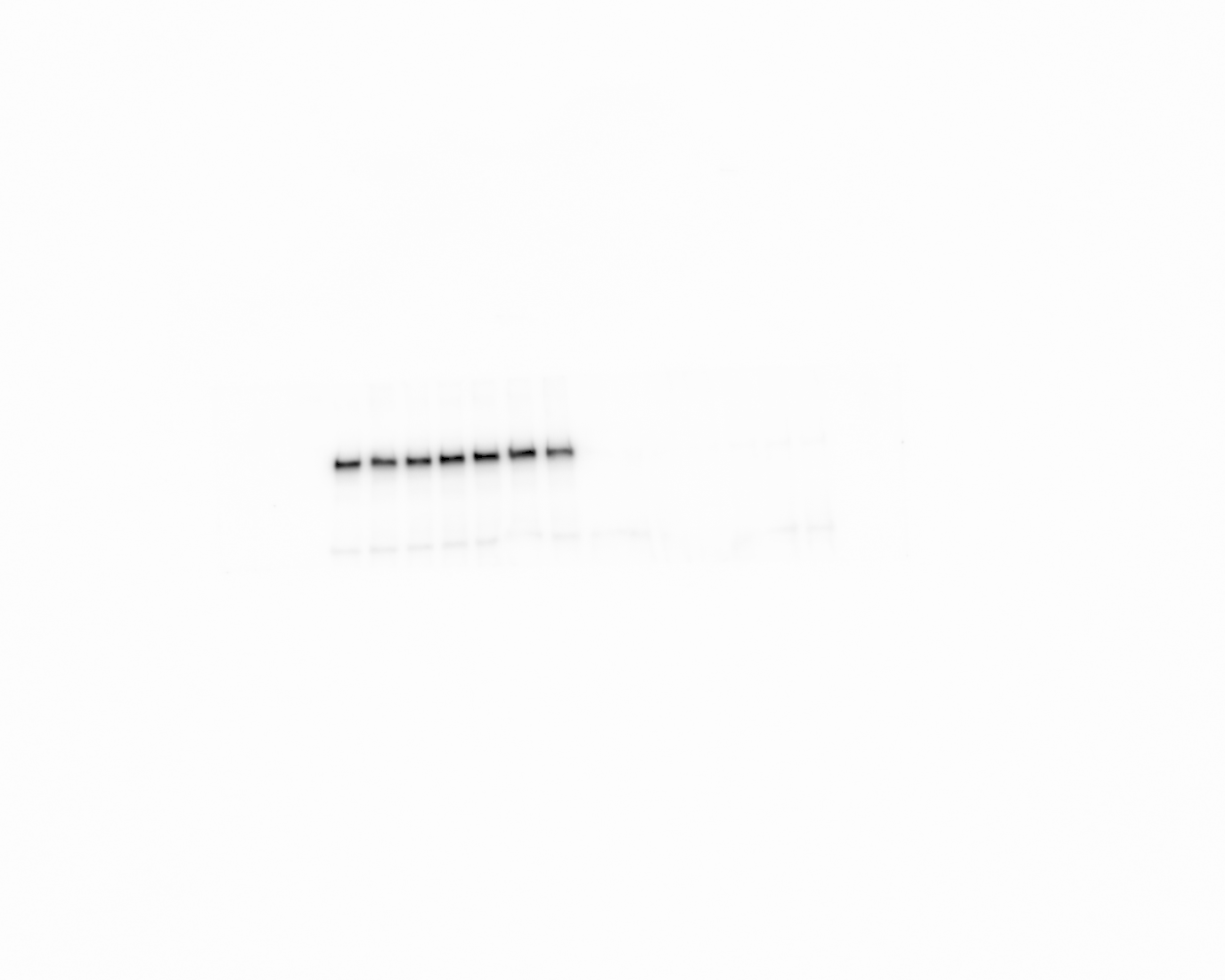

Supplement: Figure 4—figure supplement 1—source data 1. [file elife-89303-fig4-figsupp1-data1.zip › Figure 4-Figure Supplement 1-Source data 1/S4F/PARG.tif]

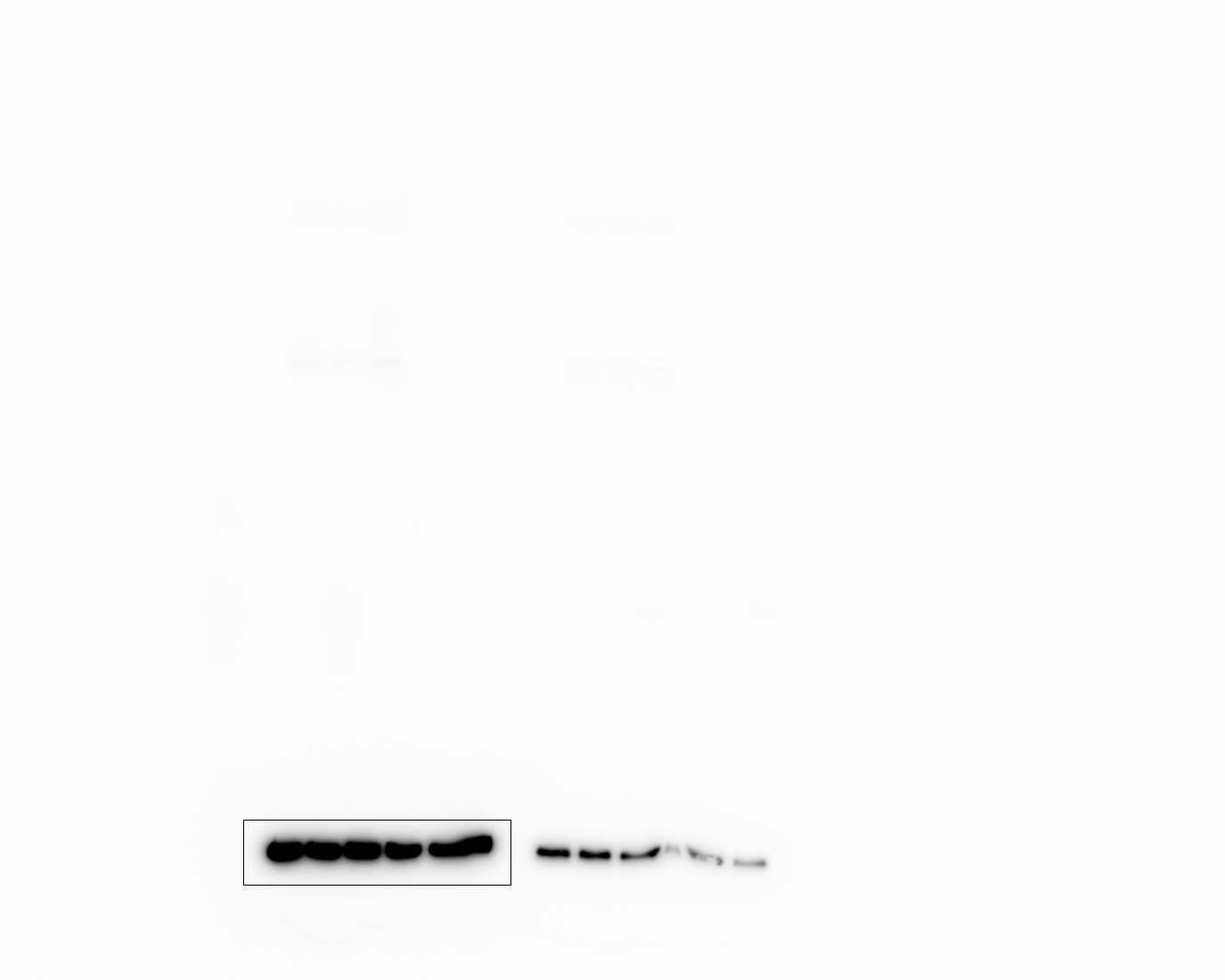

Supplement: Figure 5—figure supplement 1—source data 2. [file elife-89303-fig5-figsupp1-data2.zip › Figure 5-Figure Supplement 1-Source data 2/S5B/Actin.tif]

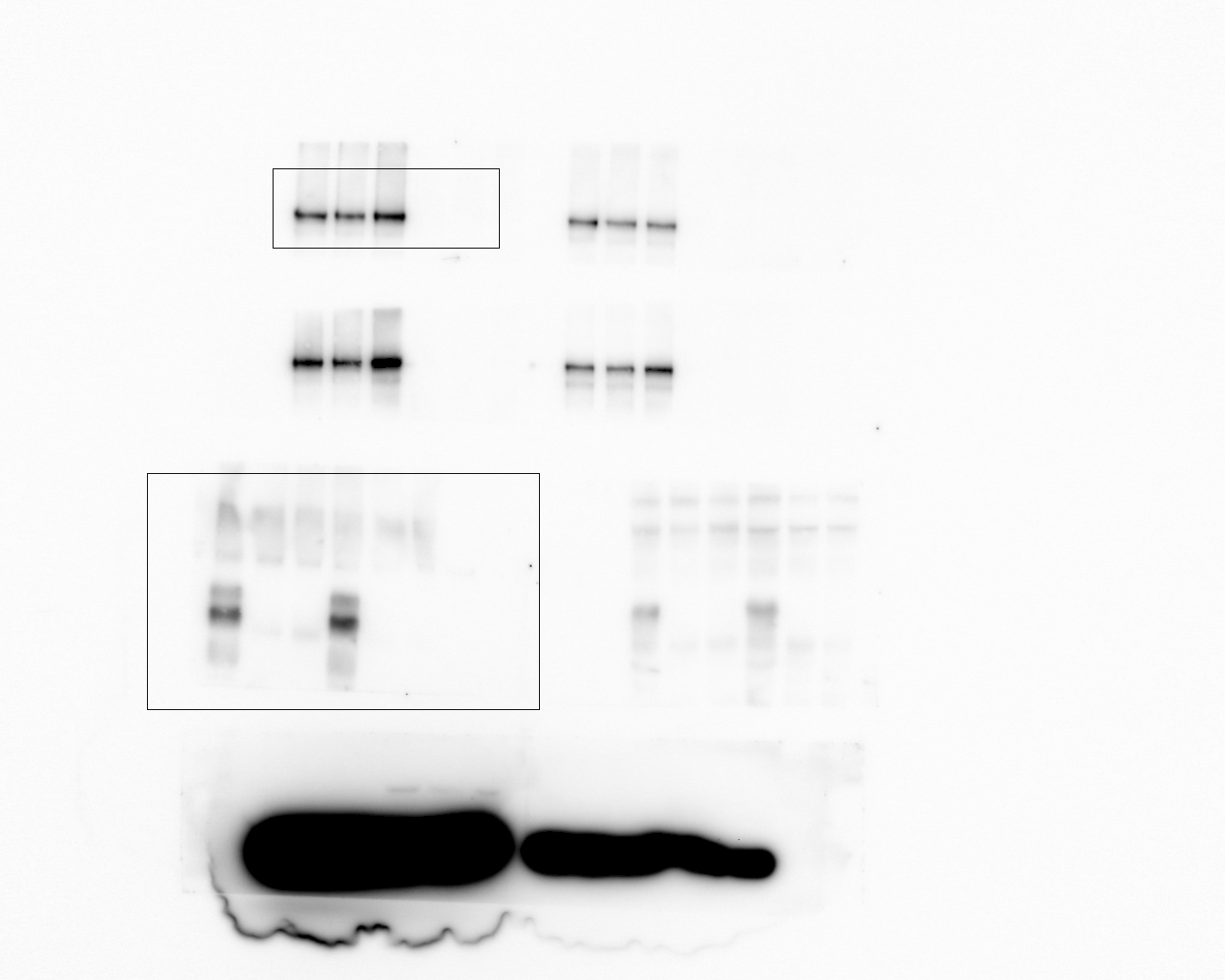

Supplement: Figure 5—figure supplement 1—source data 2. [file elife-89303-fig5-figsupp1-data2.zip › Figure 5-Figure Supplement 1-Source data 2/S5B/ARH3&PARG.tif]

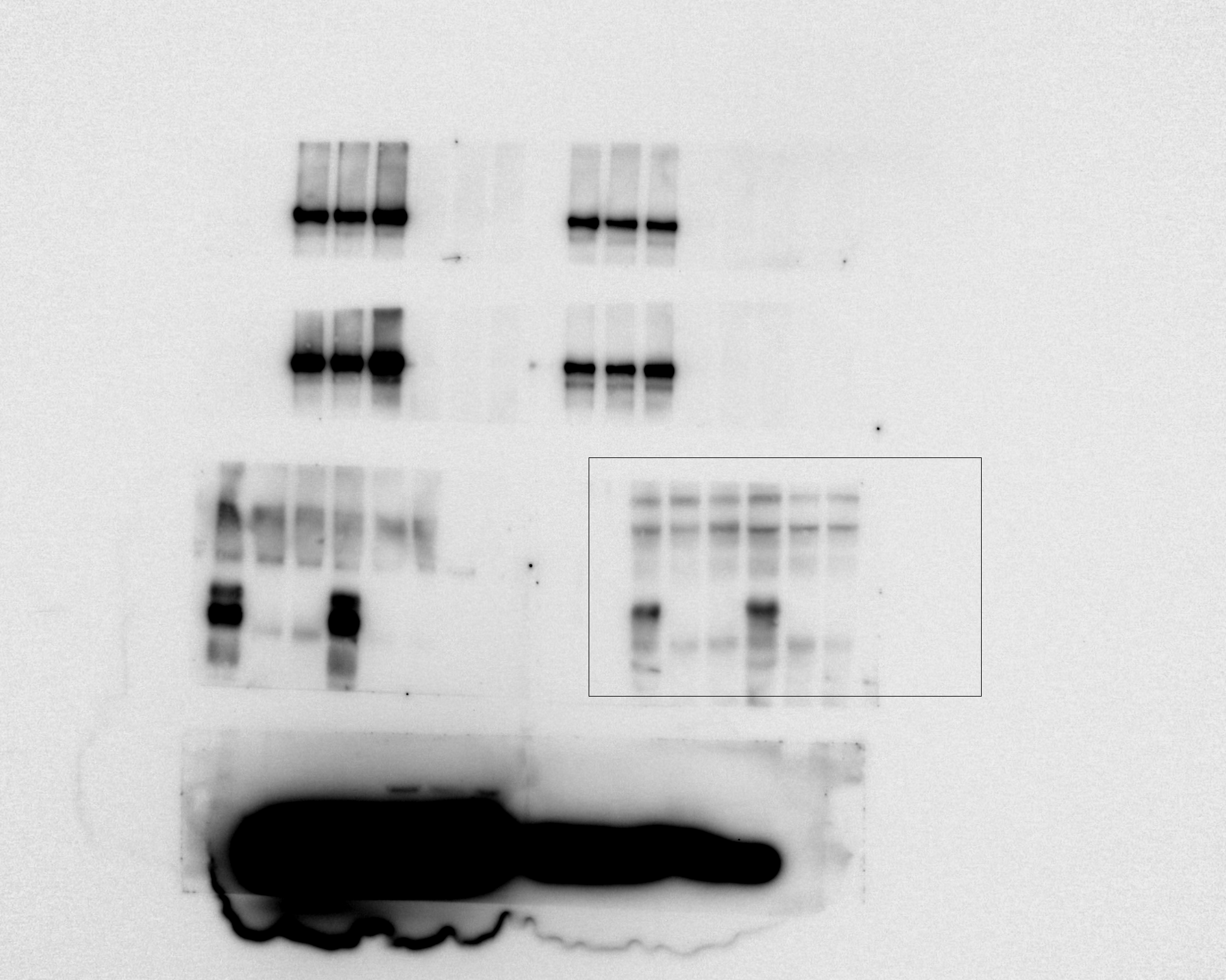

Supplement: Figure 5—figure supplement 1—source data 2. [file elife-89303-fig5-figsupp1-data2.zip › Figure 5-Figure Supplement 1-Source data 2/S5C/HPF1.tif]

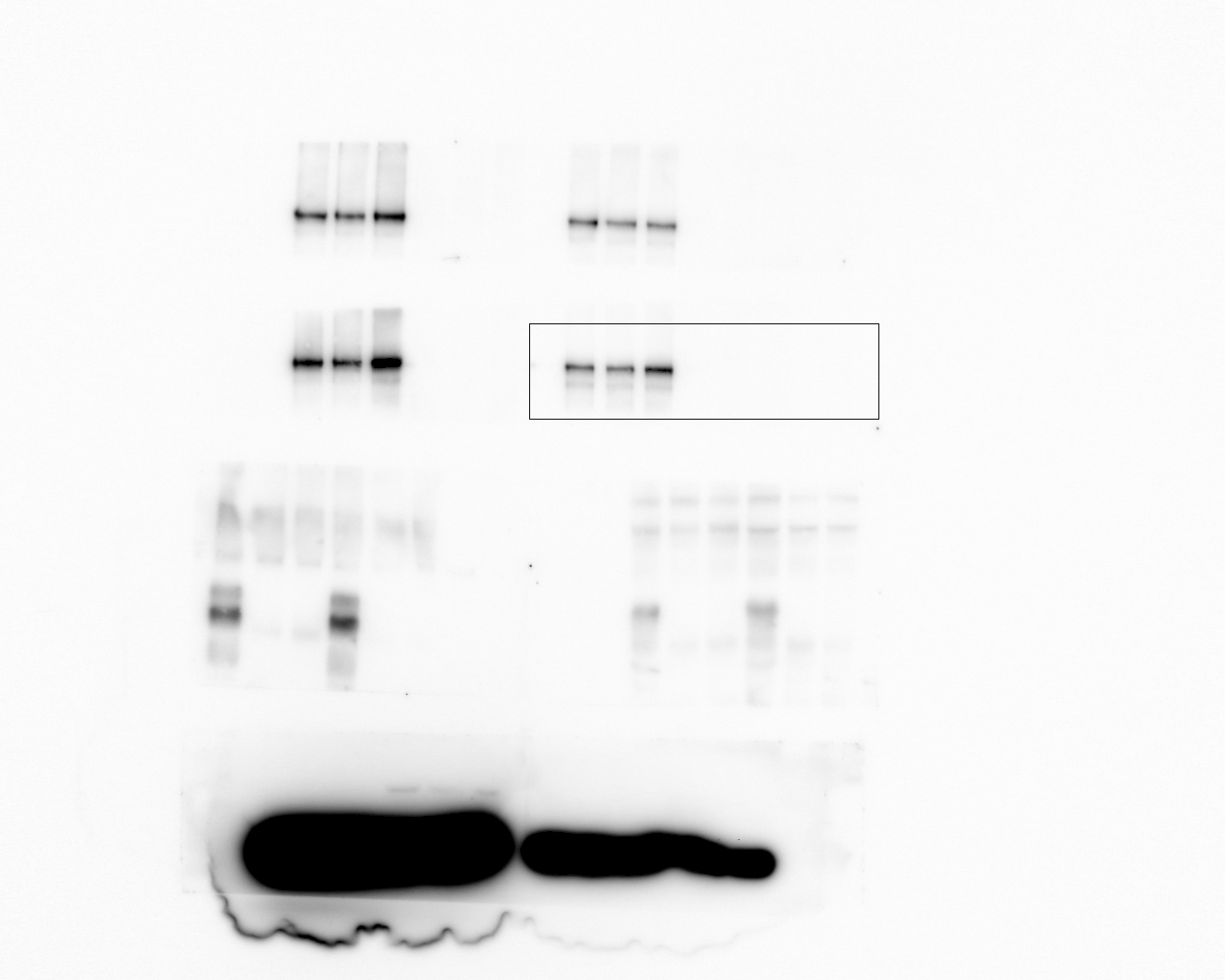

Supplement: Figure 5—figure supplement 1—source data 2. [file elife-89303-fig5-figsupp1-data2.zip › Figure 5-Figure Supplement 1-Source data 2/S5C/PARG.tif]

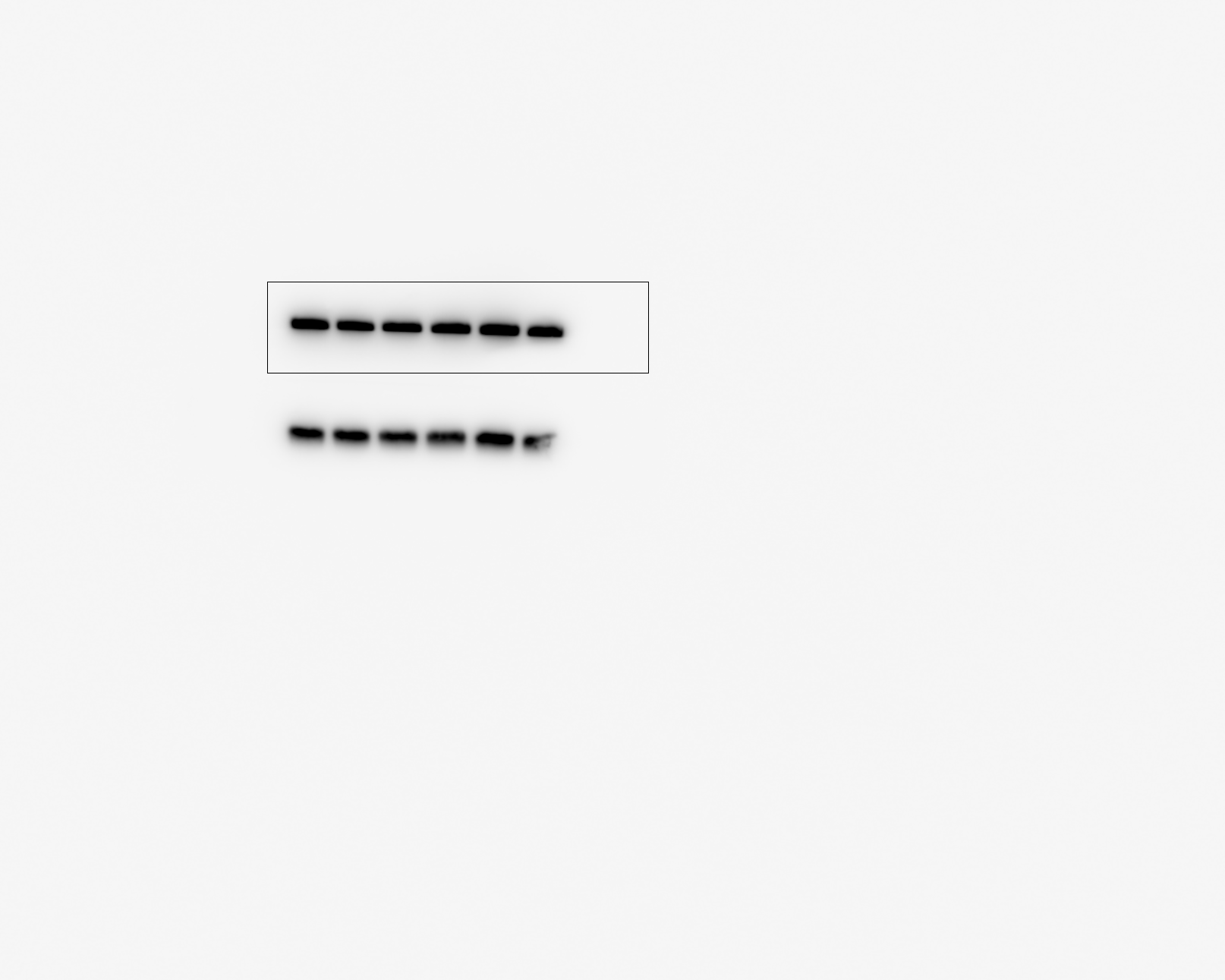

Supplement: Figure 5—figure supplement 1—source data 2. [file elife-89303-fig5-figsupp1-data2.zip › Figure 5-Figure Supplement 1-Source data 2/S5C/Tubulin.tif]

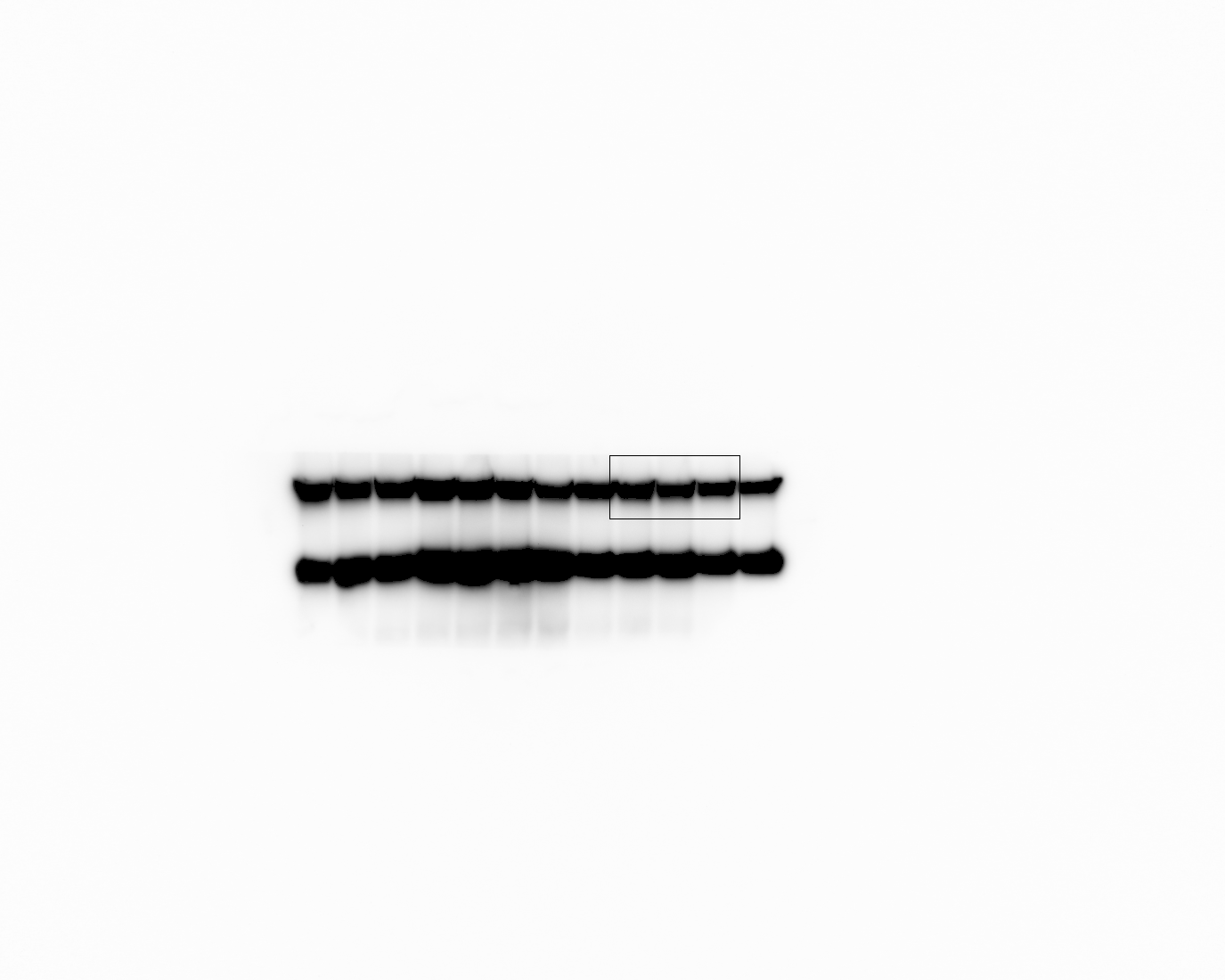

Supplement: Figure 5—figure supplement 1—source data 2. [file elife-89303-fig5-figsupp1-data2.zip › Figure 5-Figure Supplement 1-Source data 2/S5D/actin.tif]

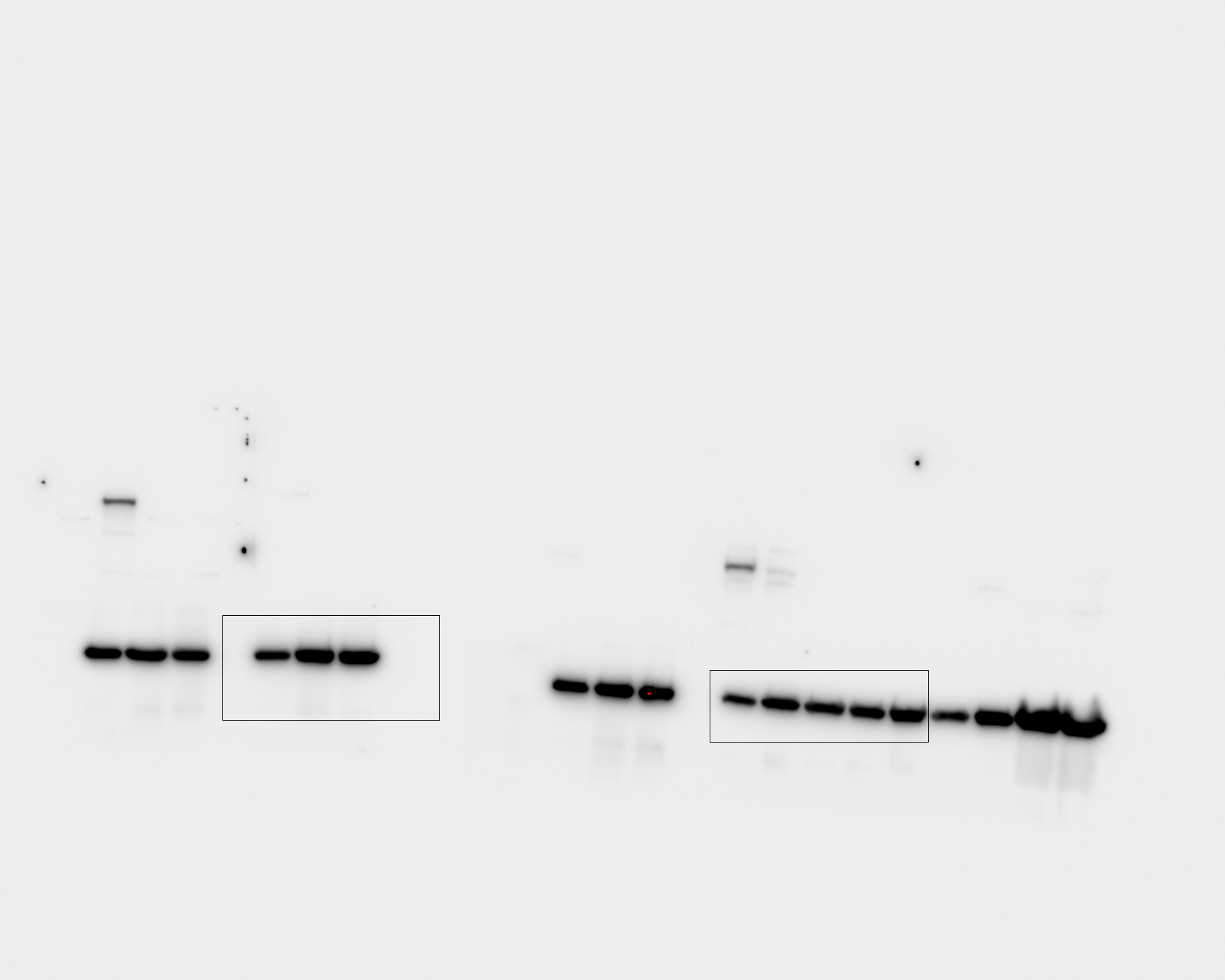

Supplement: Figure 5—figure supplement 1—source data 2. [file elife-89303-fig5-figsupp1-data2.zip › Figure 5-Figure Supplement 1-Source data 2/S5D/Actin2.tif]

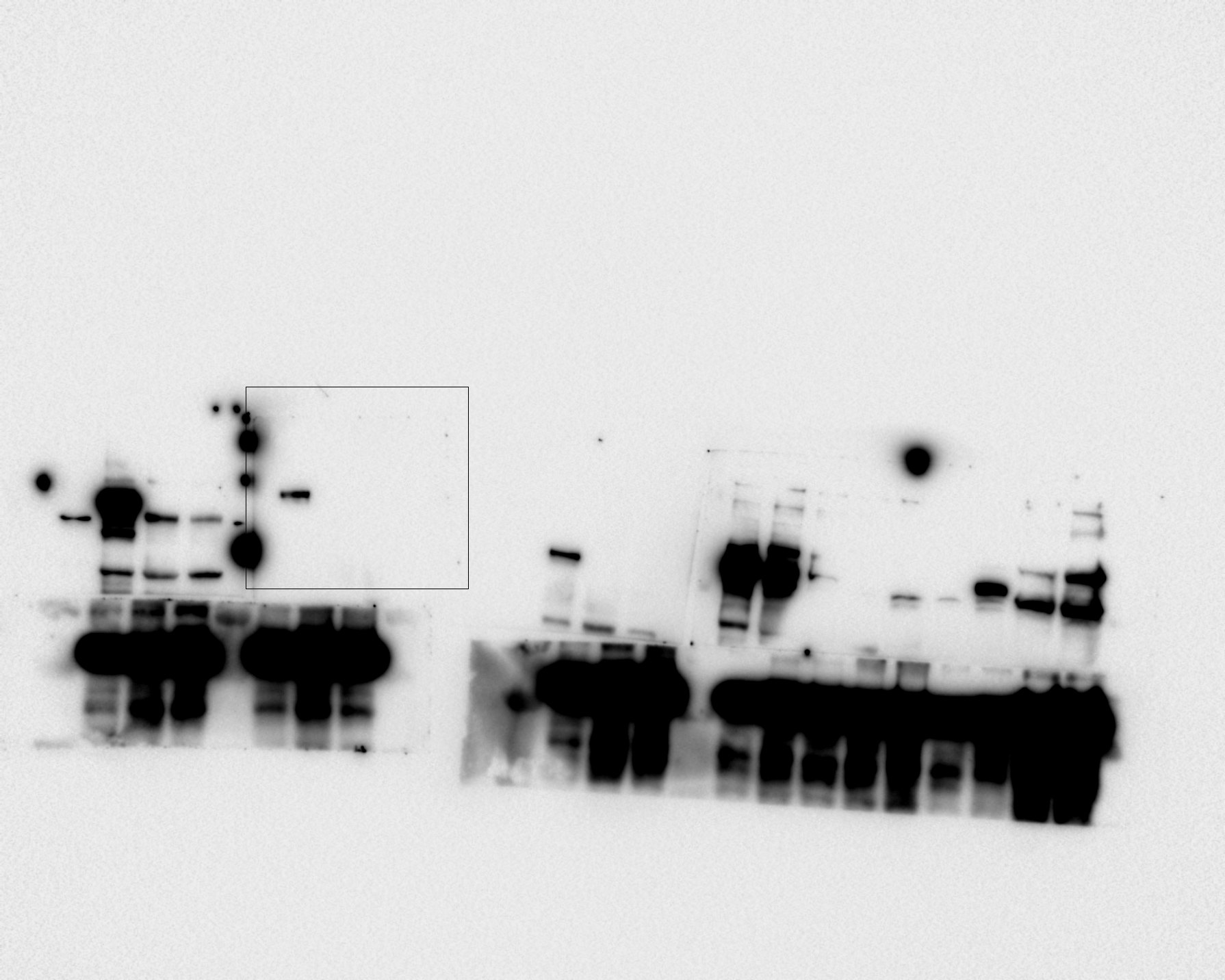

Supplement: Figure 5—figure supplement 1—source data 2. [file elife-89303-fig5-figsupp1-data2.zip › Figure 5-Figure Supplement 1-Source data 2/S5D/LIG1.tif]

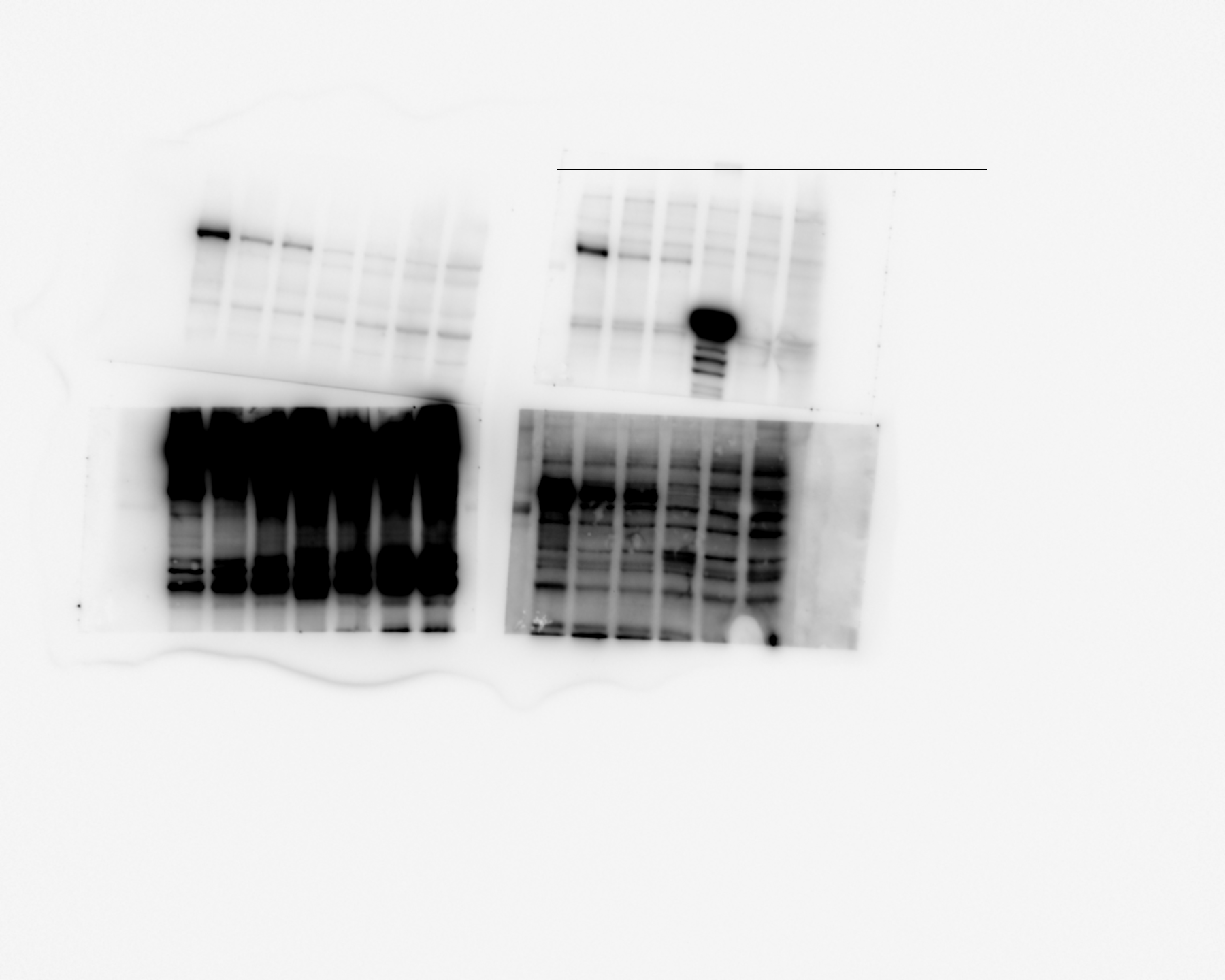

Supplement: Figure 5—figure supplement 1—source data 2. [file elife-89303-fig5-figsupp1-data2.zip › Figure 5-Figure Supplement 1-Source data 2/S5D/LIG3.tif]

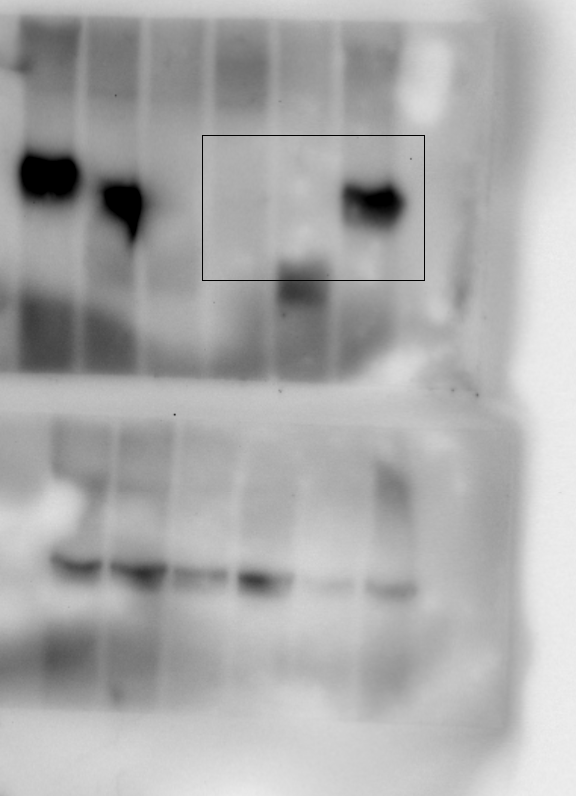

Supplement: Figure 5—figure supplement 1—source data 2. [file elife-89303-fig5-figsupp1-data2.zip › Figure 5-Figure Supplement 1-Source data 2/S5D/Polb.tif]

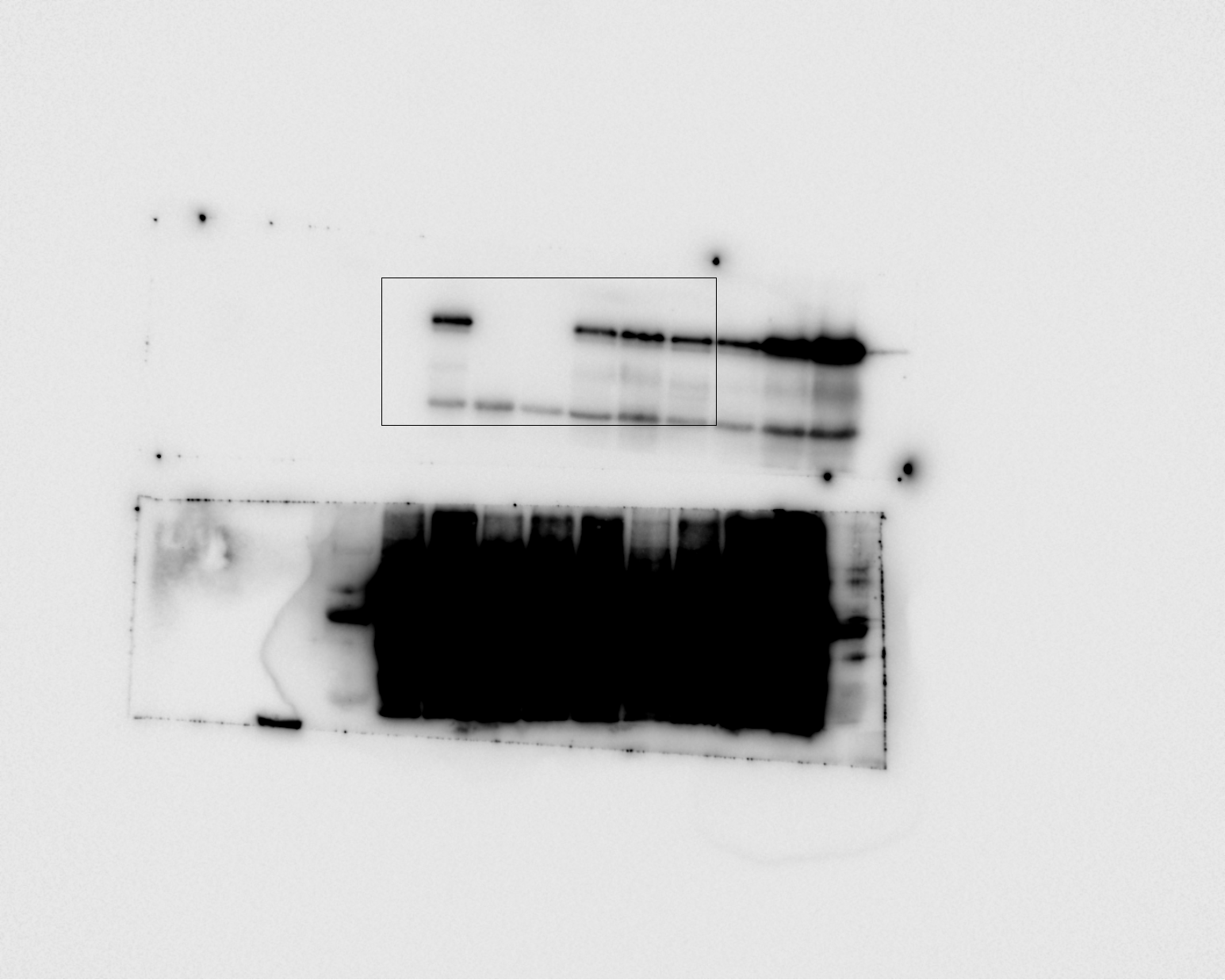

Supplement: Figure 5—figure supplement 1—source data 2. [file elife-89303-fig5-figsupp1-data2.zip › Figure 5-Figure Supplement 1-Source data 2/S5D/XRCC1.tif]

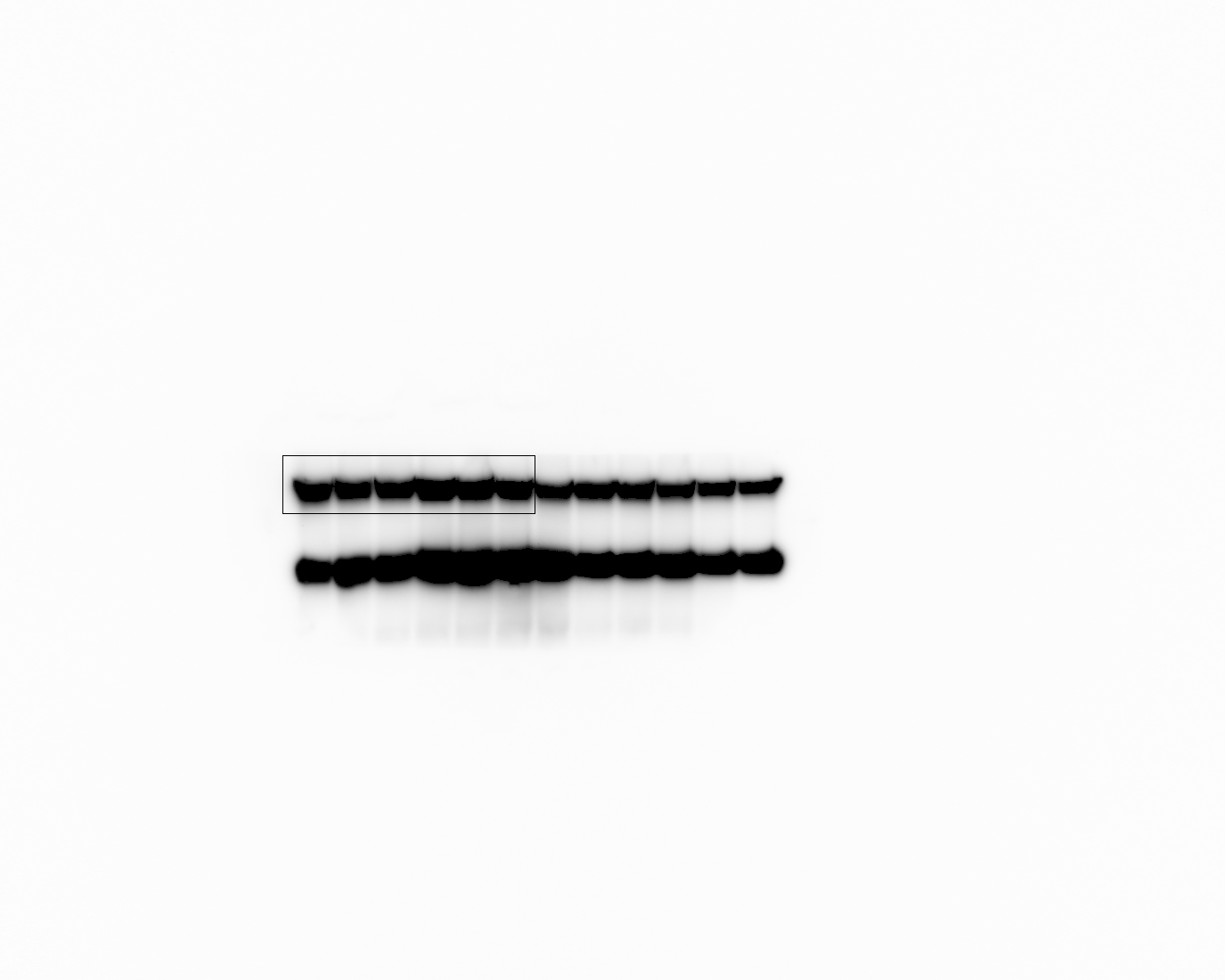

Supplement: Figure 5—figure supplement 1—source data 2. [file elife-89303-fig5-figsupp1-data2.zip › Figure 5-Figure Supplement 1-Source data 2/S5F/actin.tif]

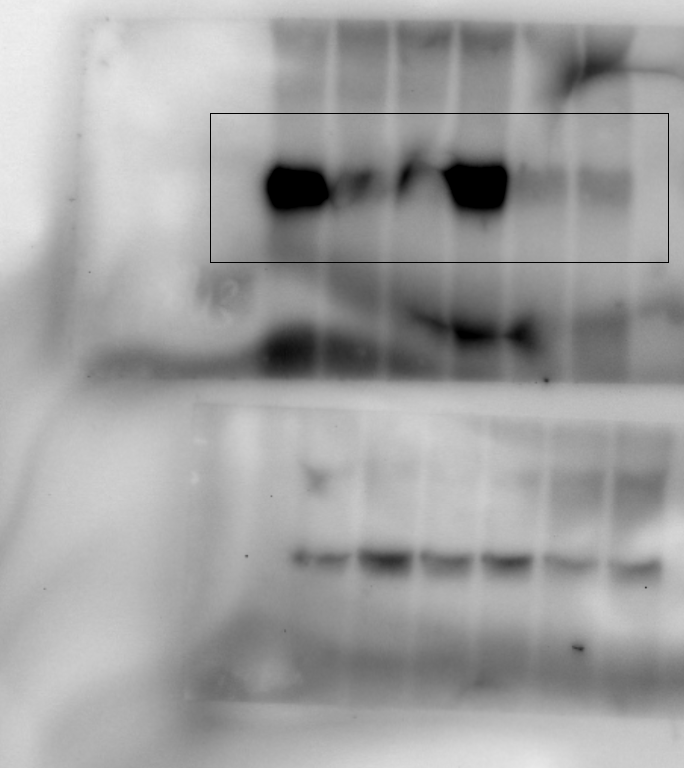

Supplement: Figure 5—figure supplement 1—source data 2. [file elife-89303-fig5-figsupp1-data2.zip › Figure 5-Figure Supplement 1-Source data 2/S5F/POLB.tif]

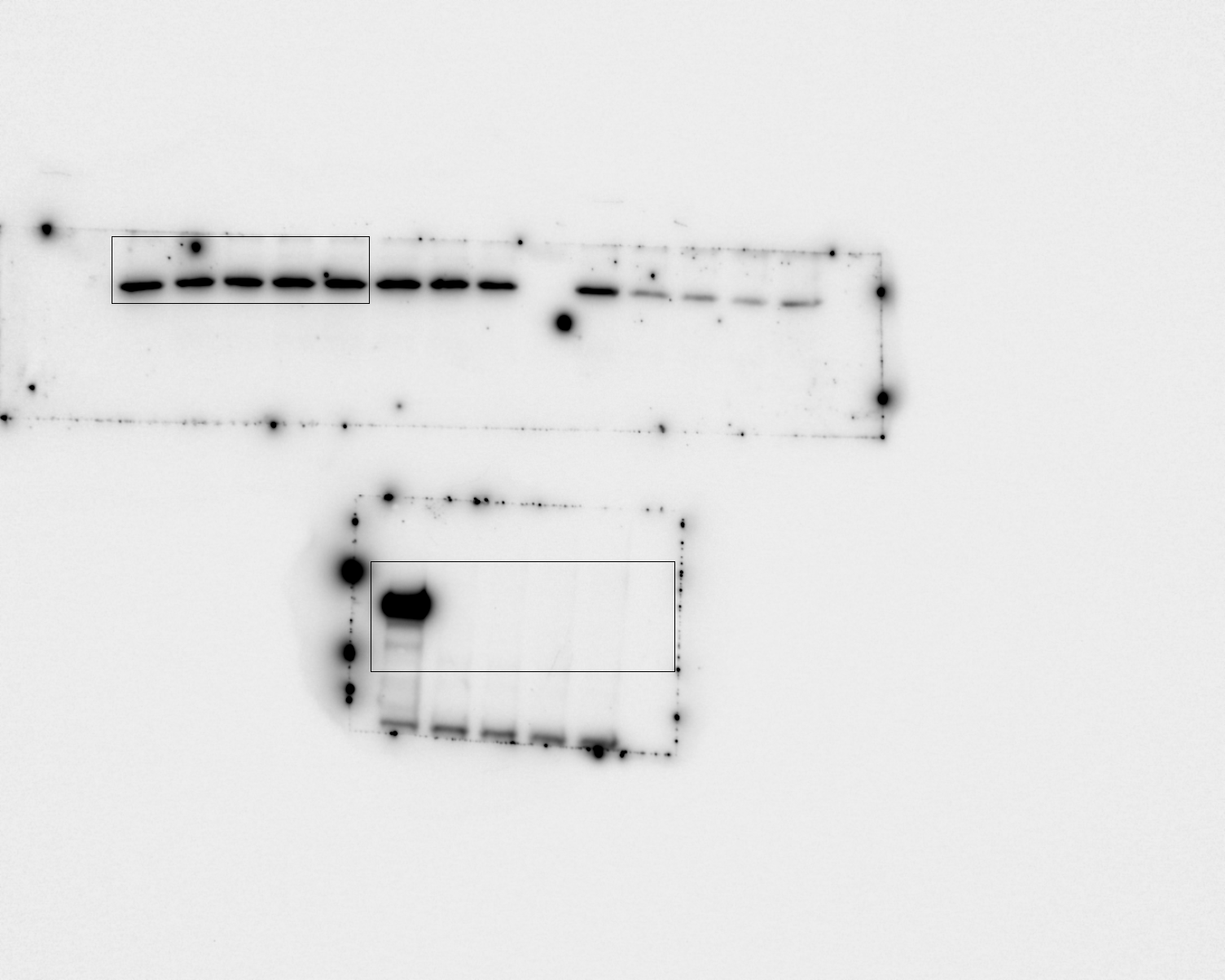

Supplement: Figure 6—source data 1. [file elife-89303-fig6-data1.zip › Figure 6-Source data 1/6B/actin&PARG.tif]

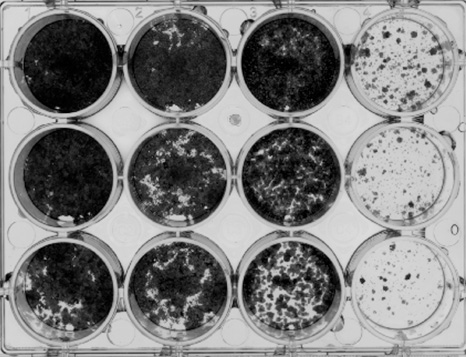

Supplement: Figure 6—source data 1. [file elife-89303-fig6-data1.zip › Figure 6-Source data 1/6D.jpg]

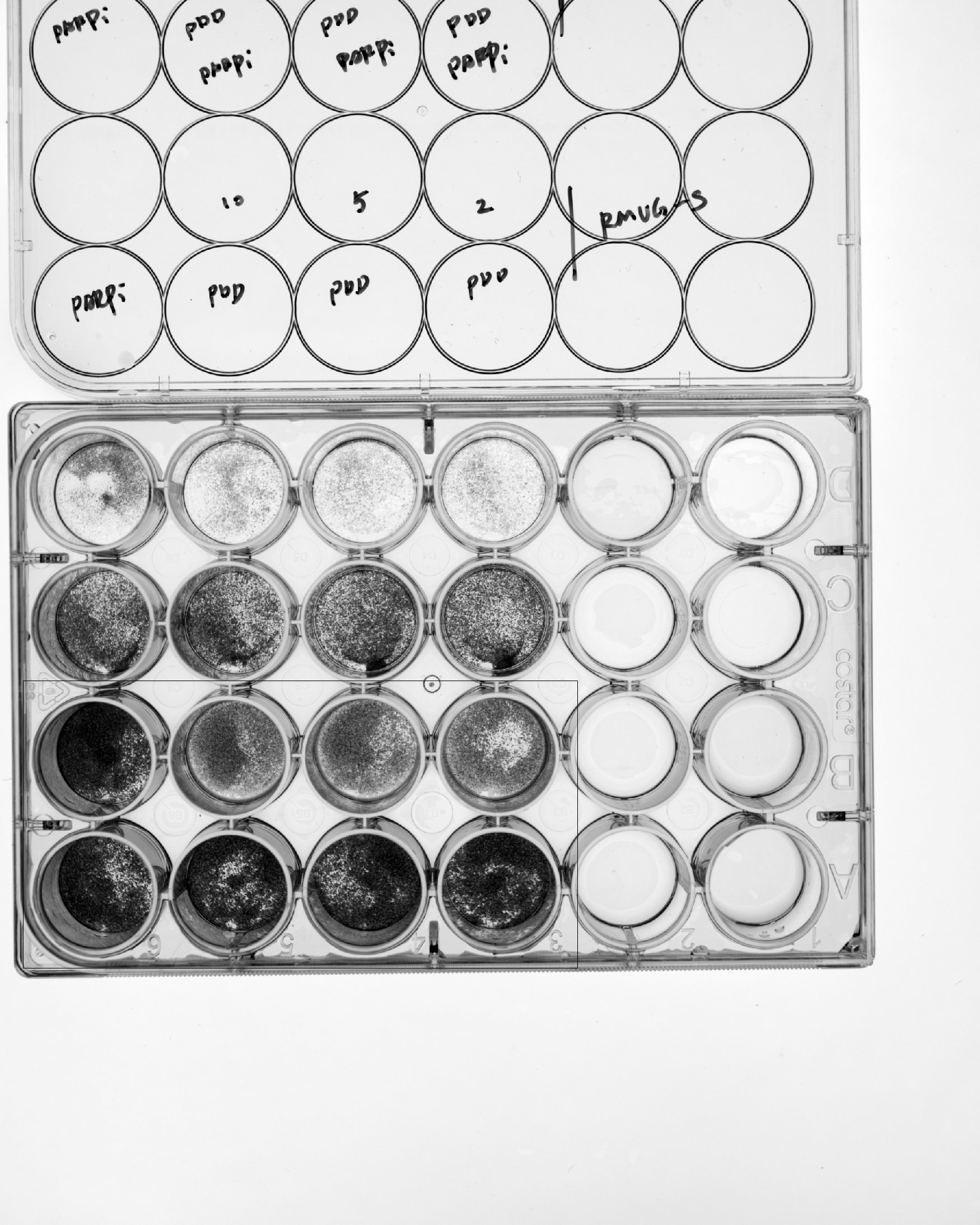

Supplement: Figure 6—source data 1. [file elife-89303-fig6-data1.zip › Figure 6-Source data 1/6E.tif]

**B**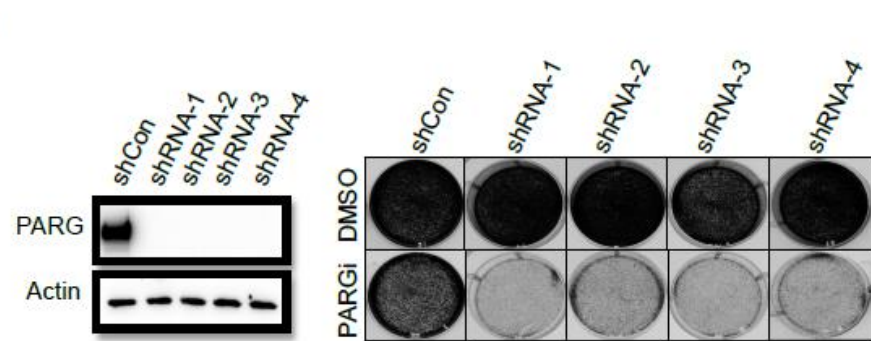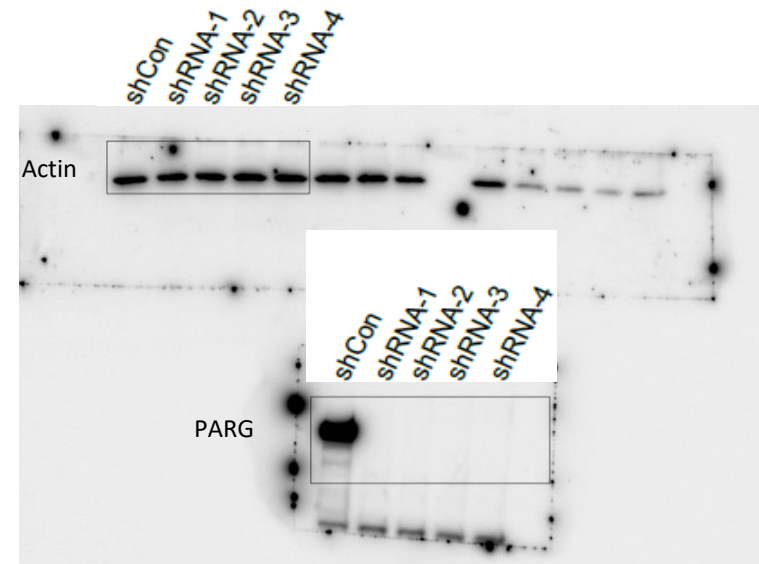

DMSO

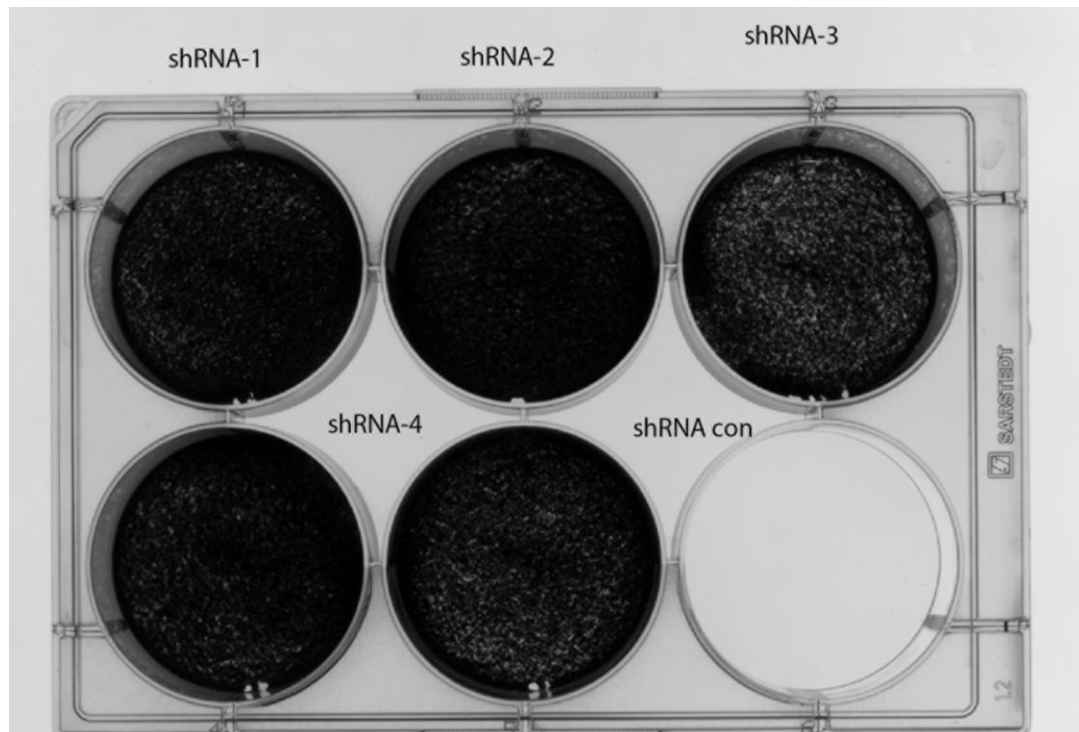

PARGi

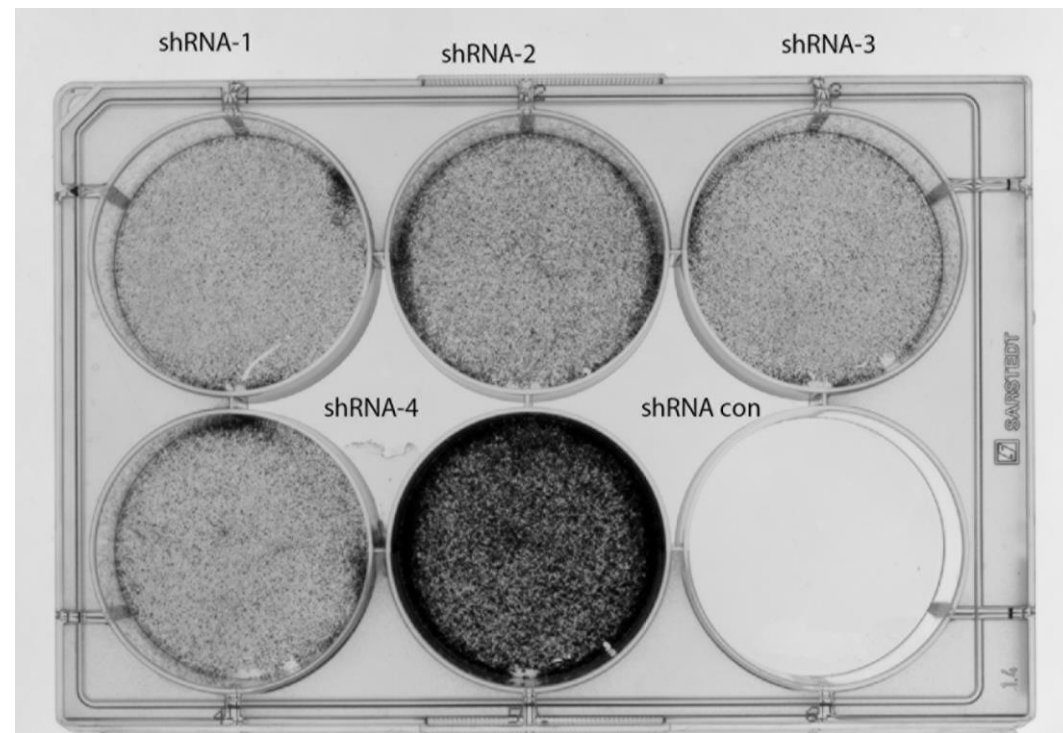

Figure 6

D

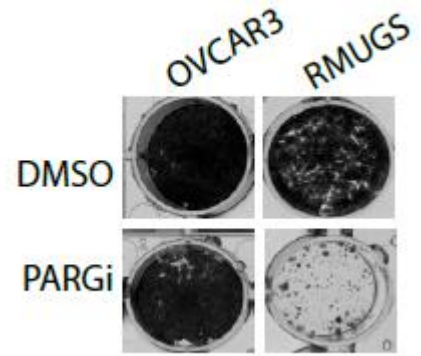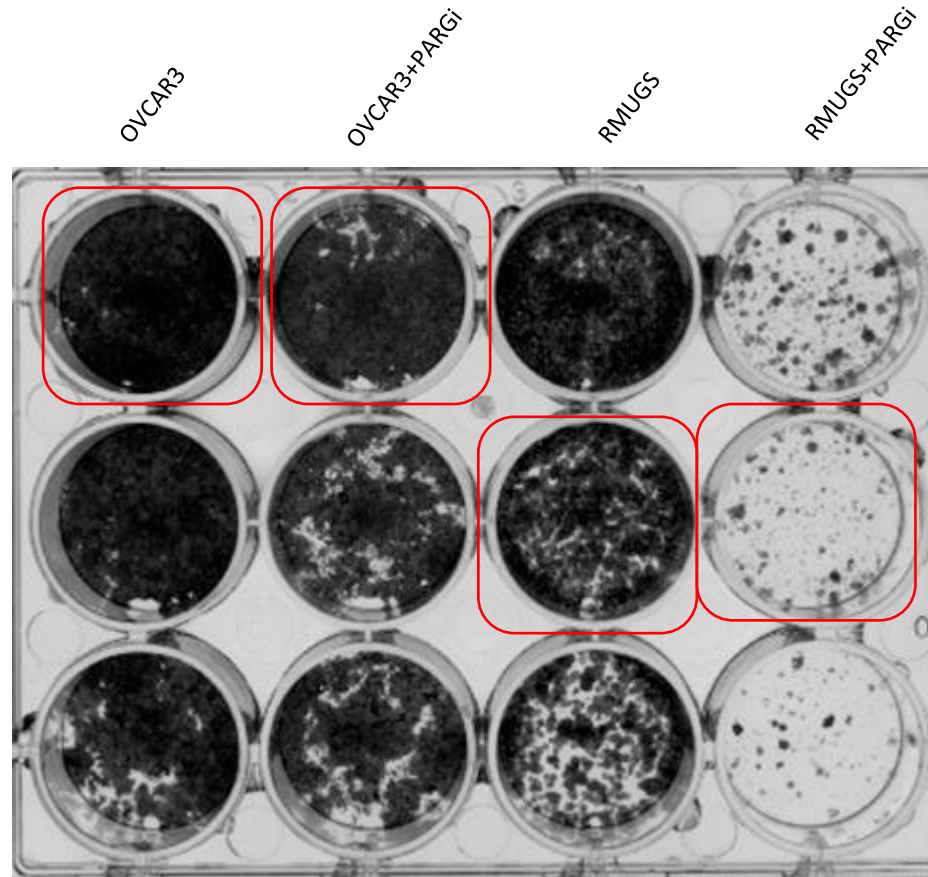

Figure 6

E

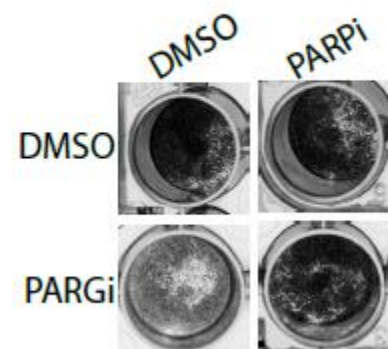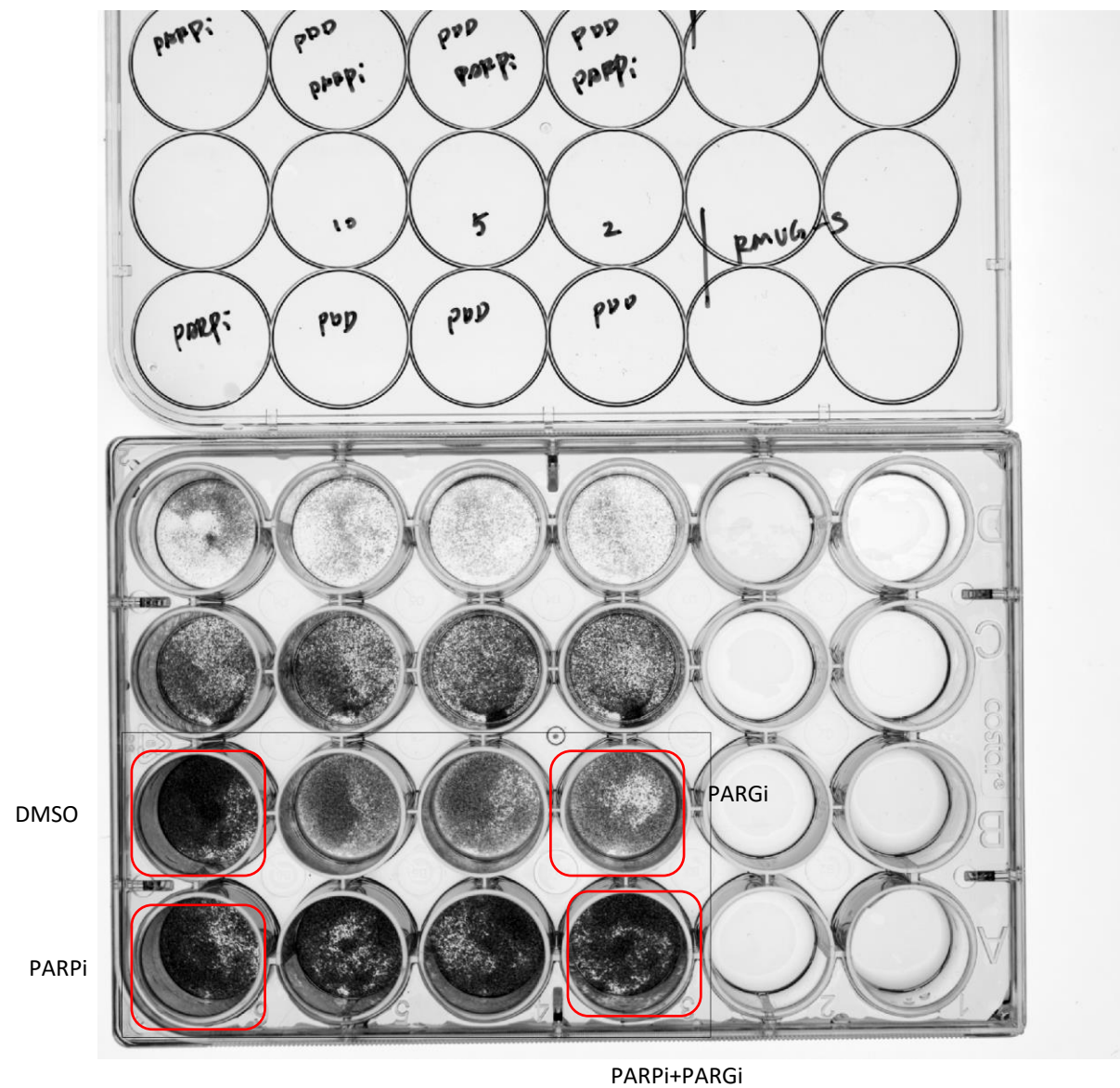

Figure 6

Supplement: Figure 6—source data 2. [file elife-89303-fig6-data2.zip › Figure 6-Source data 2/Figure 6-Source data 2.pdf]

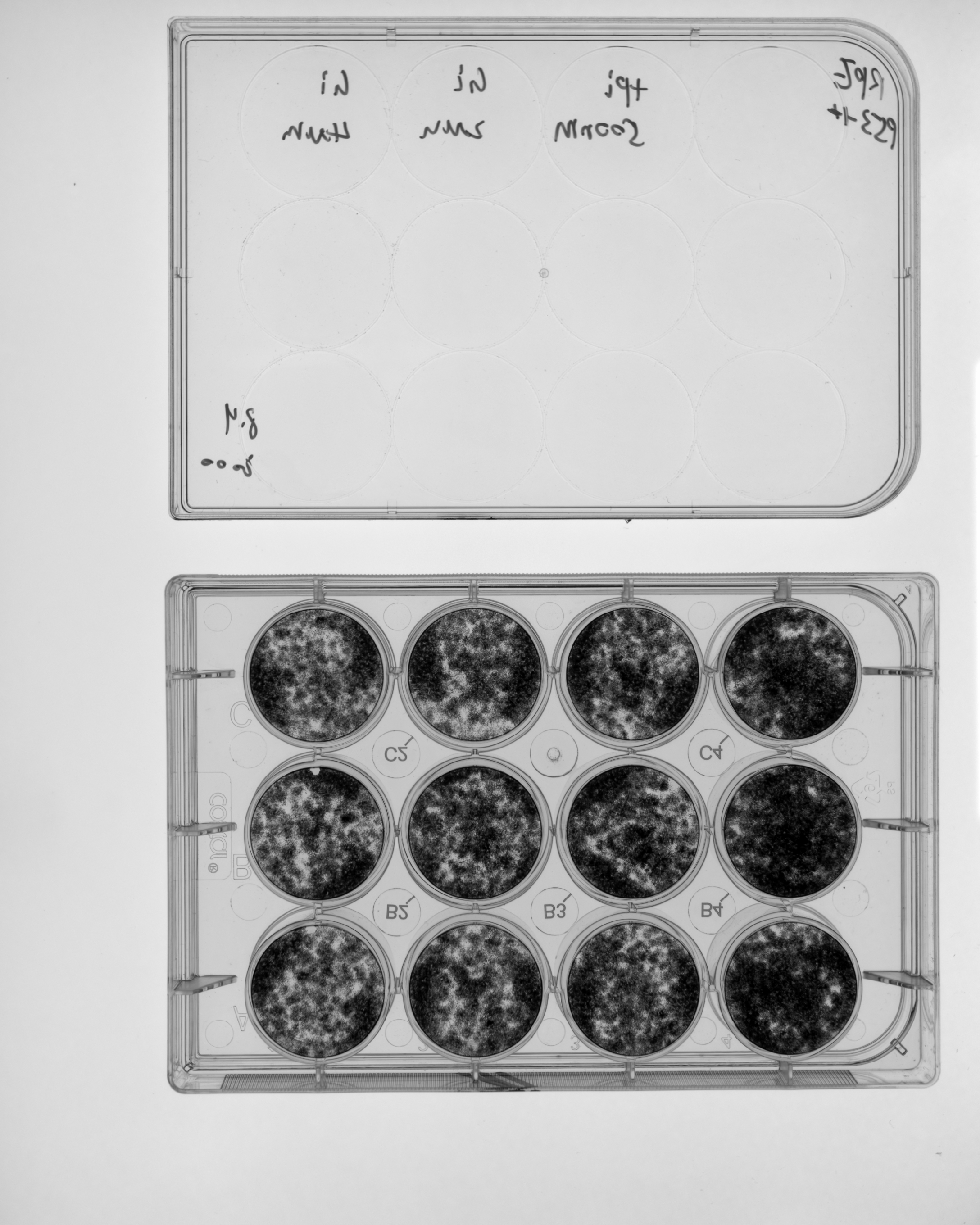

Supplement: Figure 6—figure supplement 1—source data 1. [file elife-89303-fig6-figsupp1-data1.zip › Figure 6-Figure Supplement 1-Source data 1/S6A/litong nie 2022-08-19 11h23m46s(Coomassie Blue).tif]

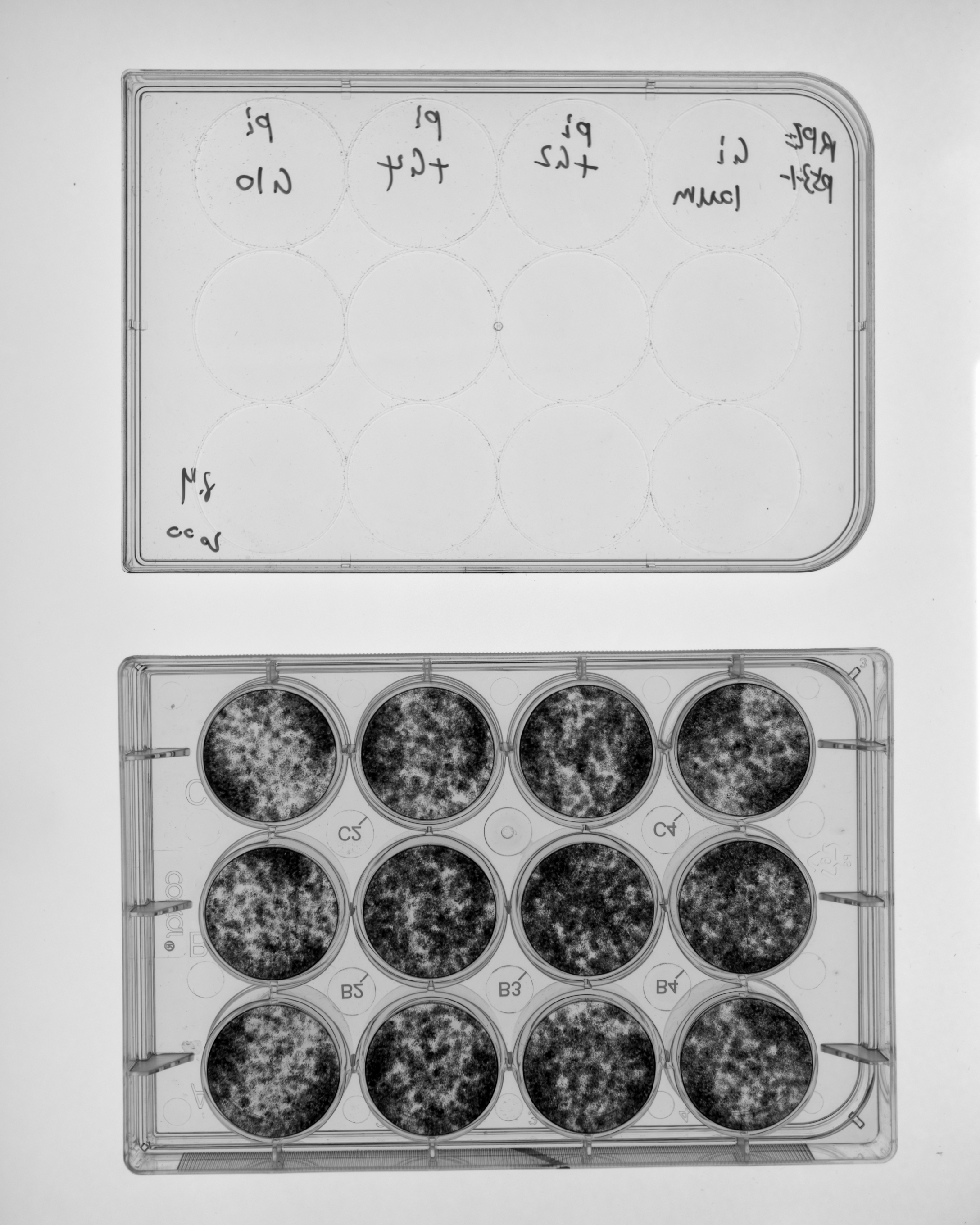

Supplement: Figure 6—figure supplement 1—source data 1. [file elife-89303-fig6-figsupp1-data1.zip › Figure 6-Figure Supplement 1-Source data 1/S6A/litong nie 2022-08-19 11h25m16s(Coomassie Blue).tif]

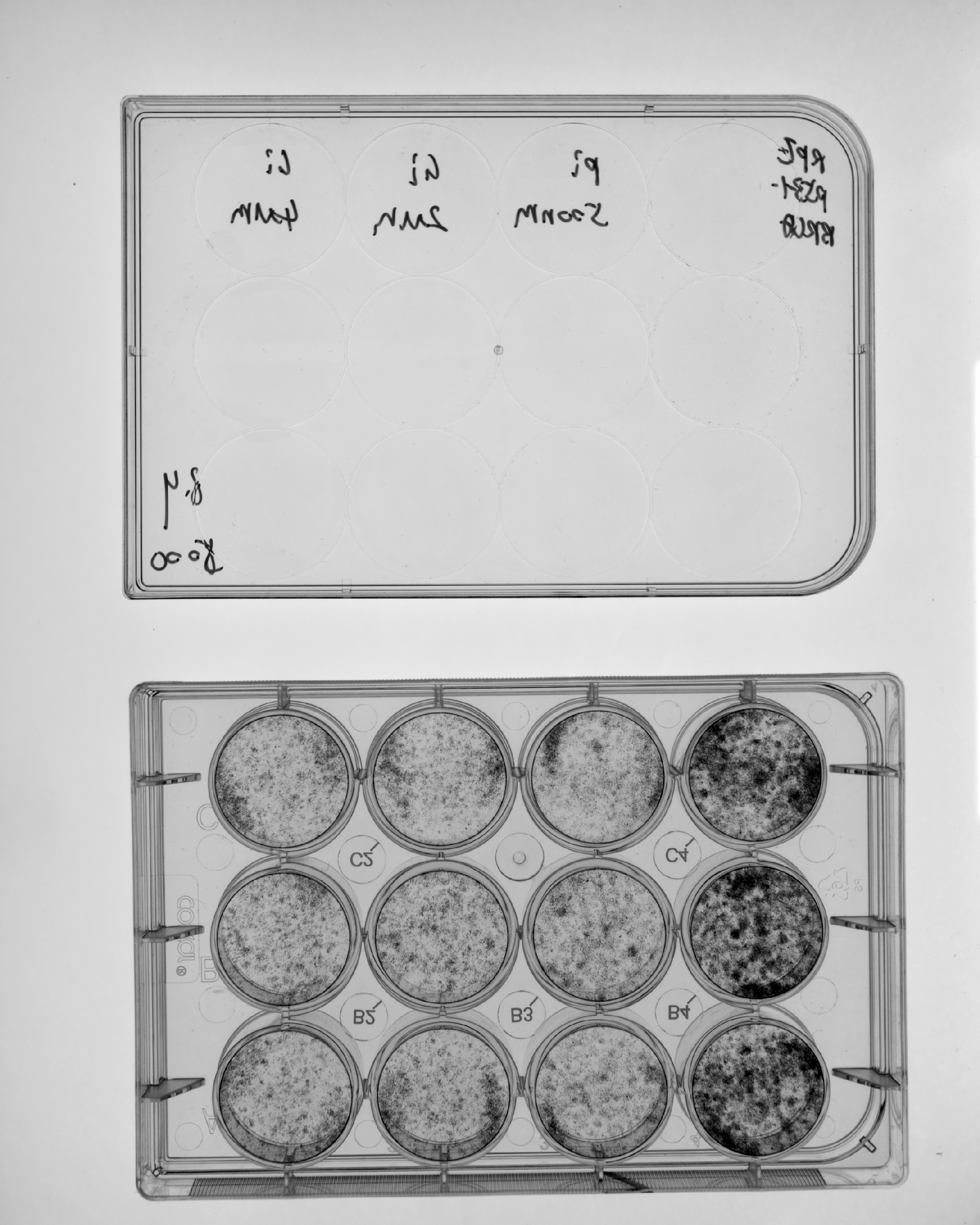

Supplement: Figure 6—figure supplement 1—source data 1. [file elife-89303-fig6-figsupp1-data1.zip › Figure 6-Figure Supplement 1-Source data 1/S6A/litong nie 2022-08-19 11h26m09s(Coomassie Blue).tif]

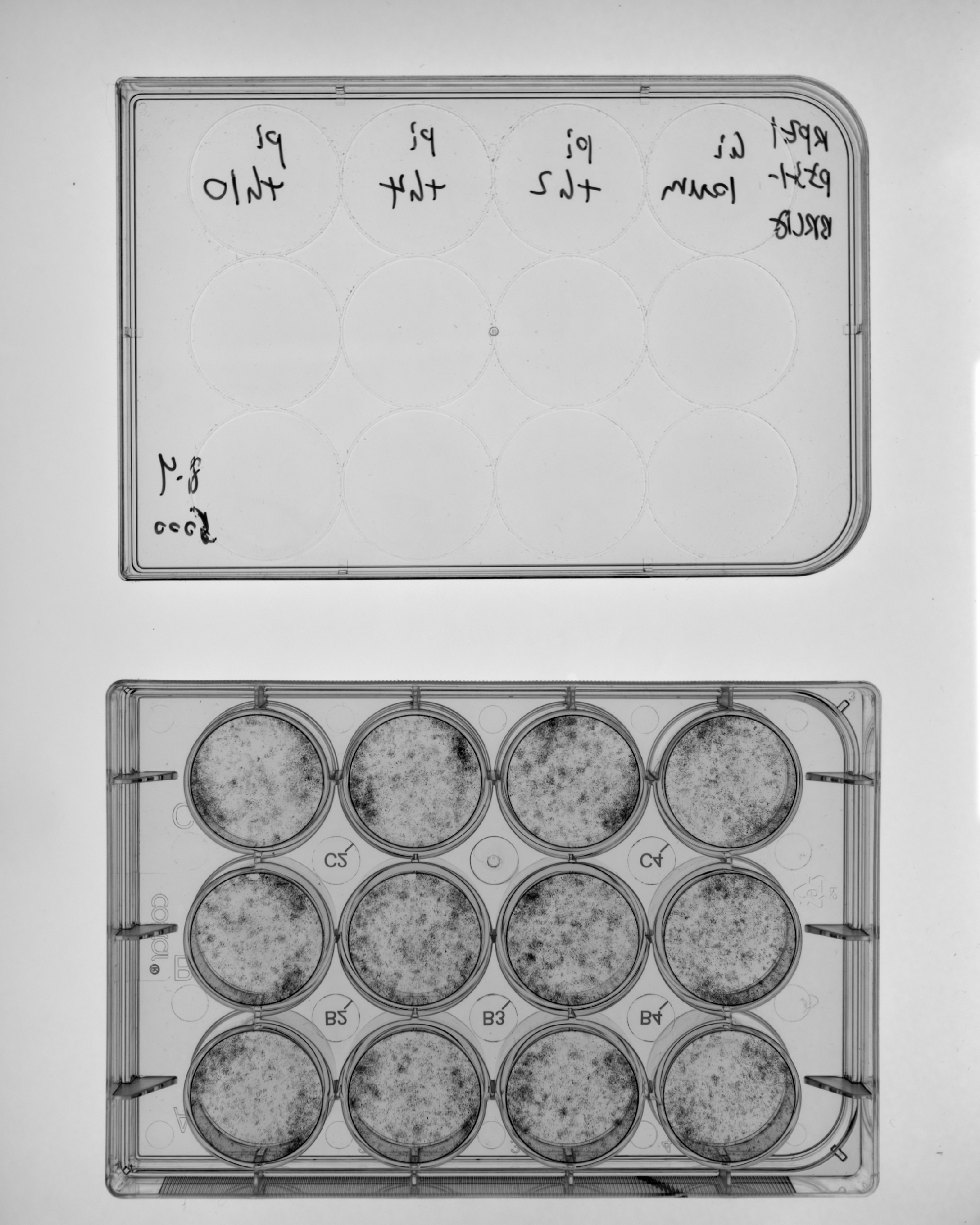

Supplement: Figure 6—figure supplement 1—source data 1. [file elife-89303-fig6-figsupp1-data1.zip › Figure 6-Figure Supplement 1-Source data 1/S6A/litong nie 2022-08-19 11h27m04s(Coomassie Blue).tif]

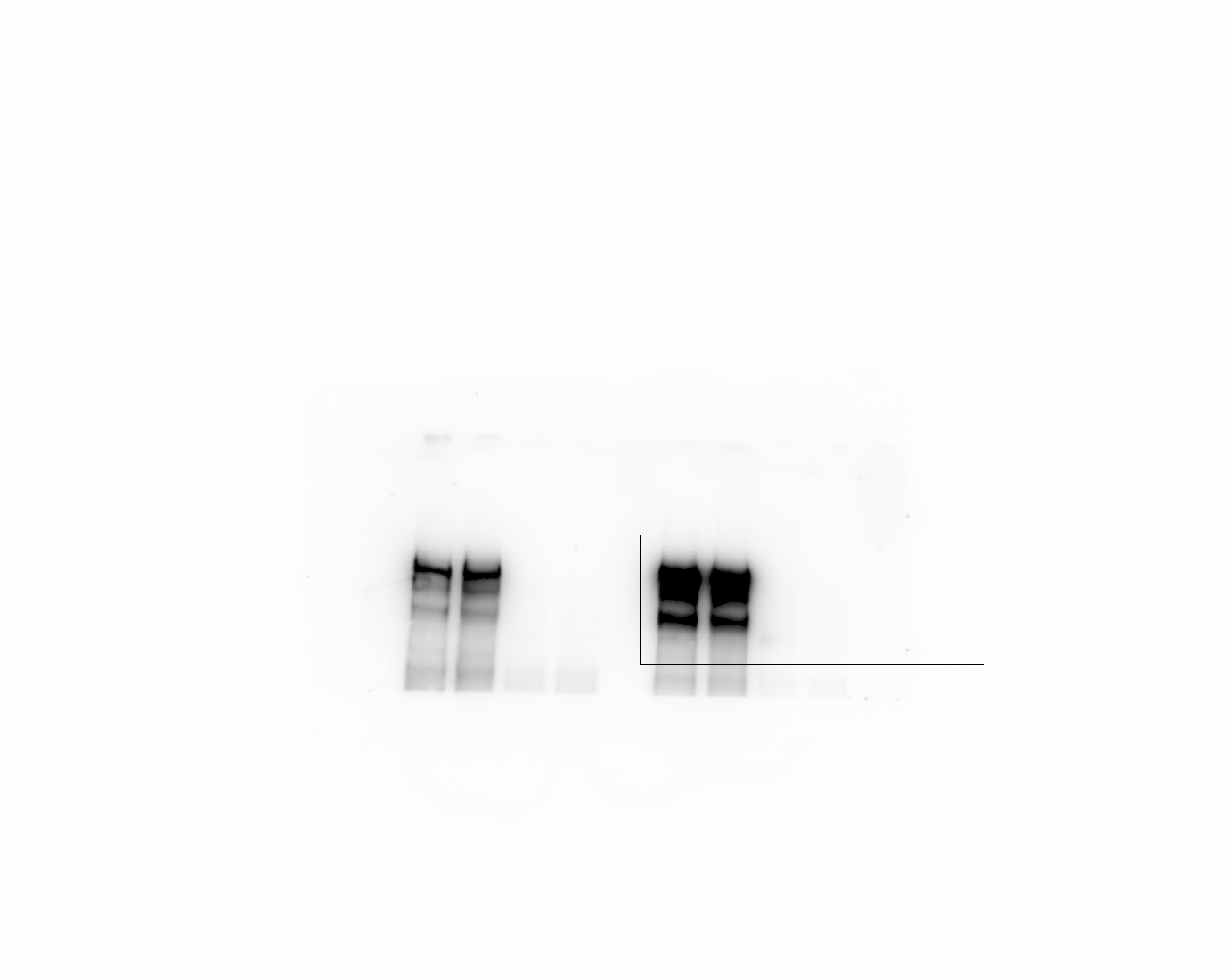

Supplement: Figure 6—figure supplement 1—source data 1. [file elife-89303-fig6-figsupp1-data1.zip › Figure 6-Figure Supplement 1-Source data 1/S6B/53BP1.tif]

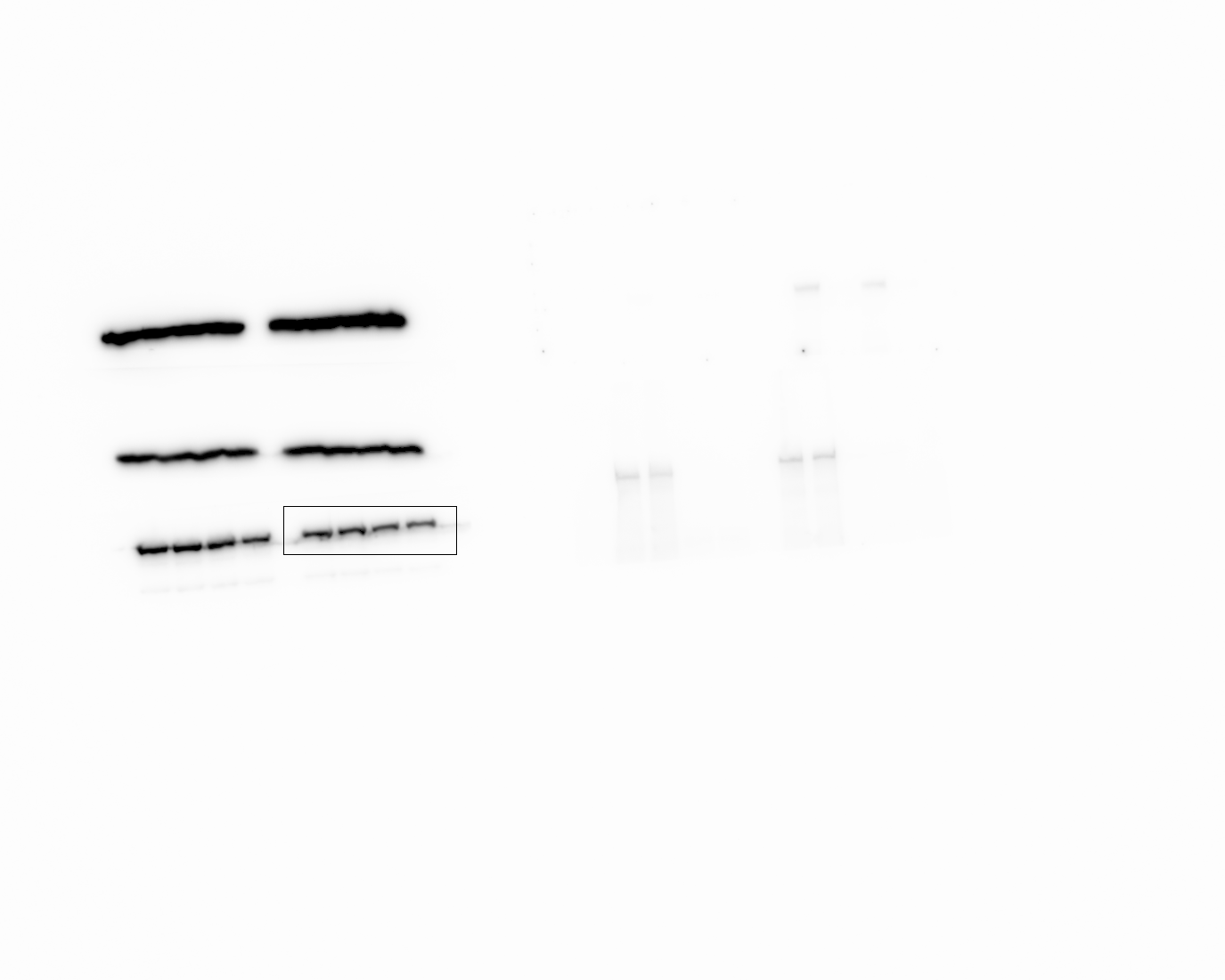

Supplement: Figure 6—figure supplement 1—source data 1. [file elife-89303-fig6-figsupp1-data1.zip › Figure 6-Figure Supplement 1-Source data 1/S6B/actin.tif]

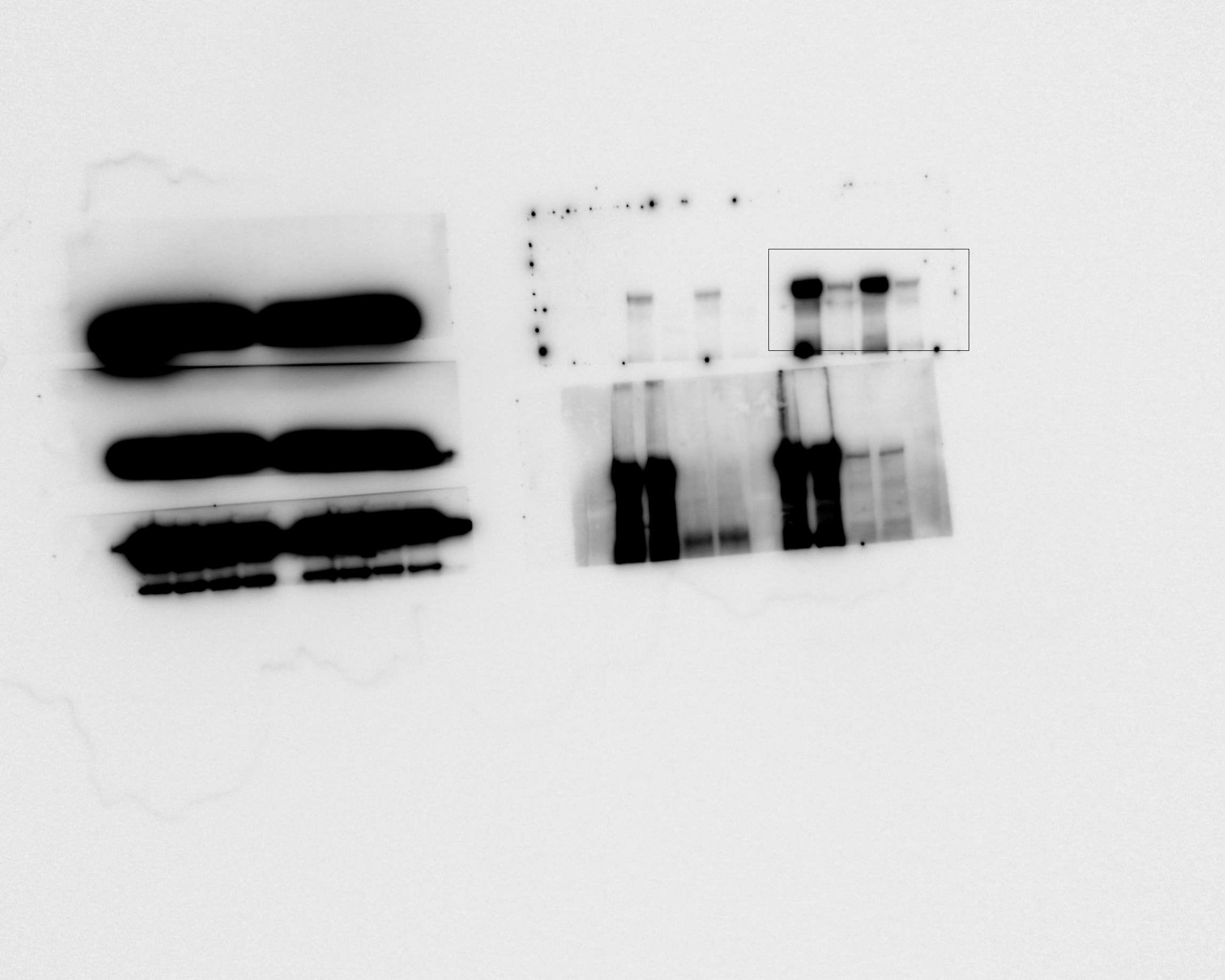

Supplement: Figure 6—figure supplement 1—source data 1. [file elife-89303-fig6-figsupp1-data1.zip › Figure 6-Figure Supplement 1-Source data 1/S6B/BRCA1.tif]

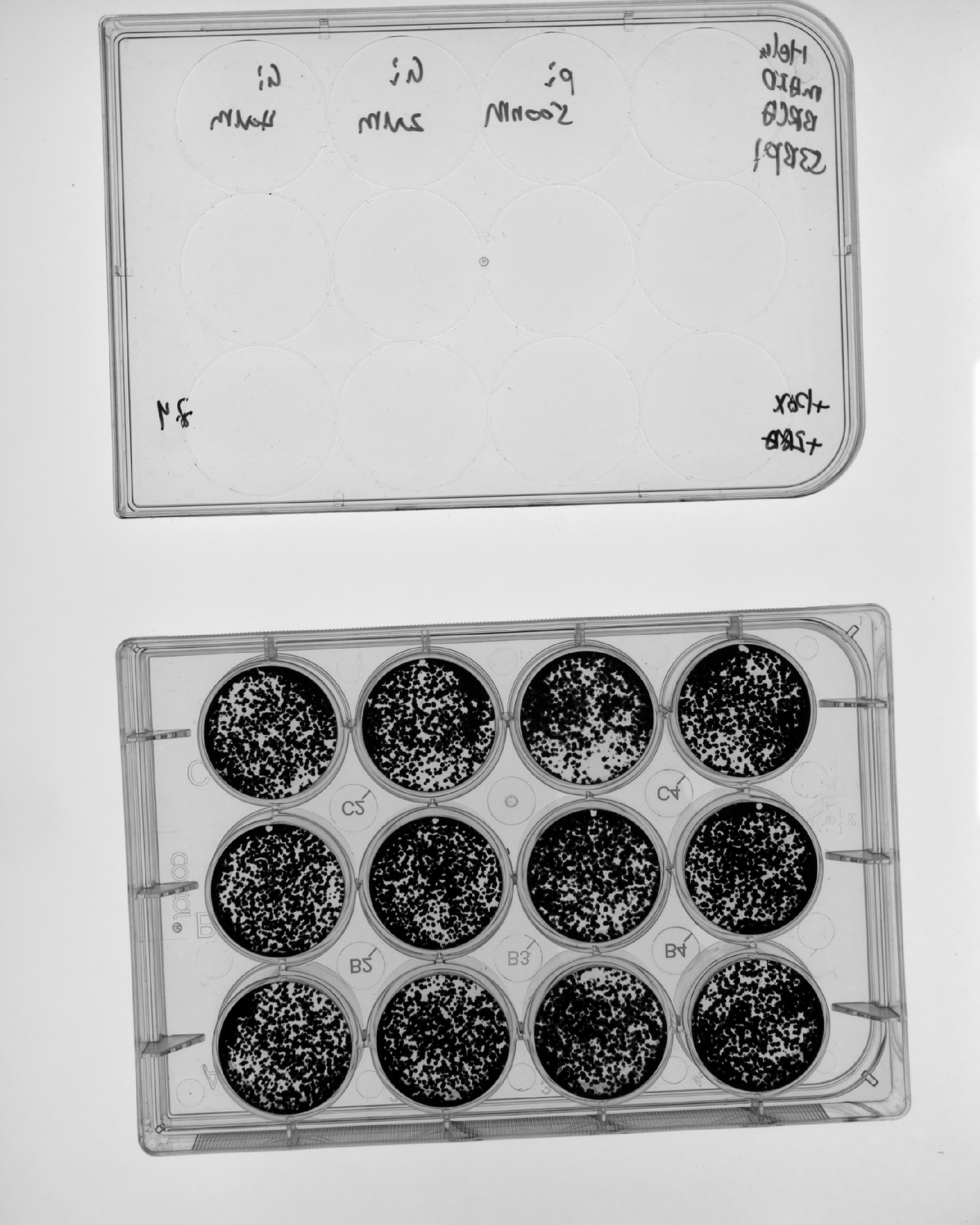

Supplement: Figure 6—figure supplement 1—source data 1. [file elife-89303-fig6-figsupp1-data1.zip › Figure 6-Figure Supplement 1-Source data 1/S6B/litong nie 2022-08-19 11h27m57s(Coomassie Blue).tif]

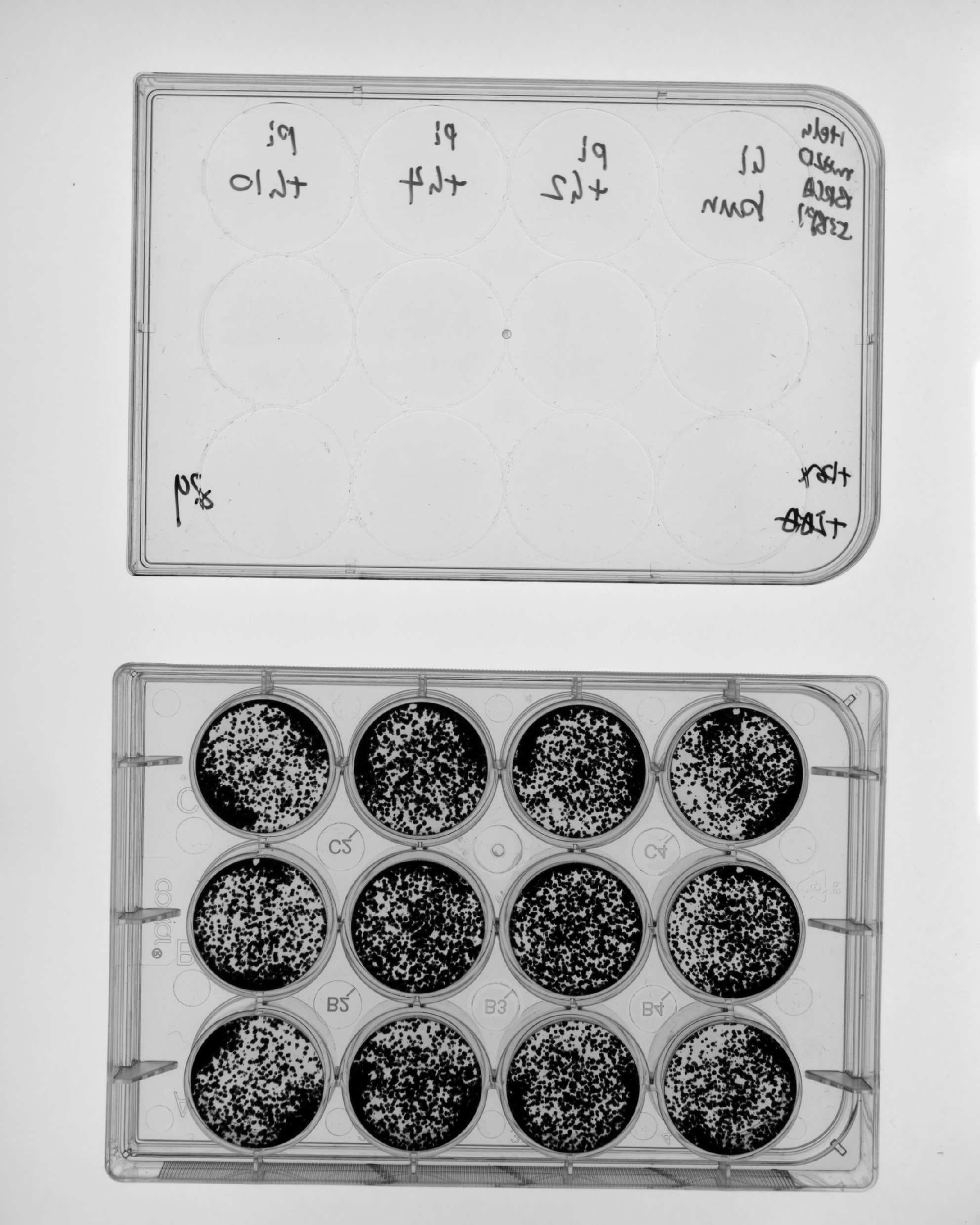

Supplement: Figure 6—figure supplement 1—source data 1. [file elife-89303-fig6-figsupp1-data1.zip › Figure 6-Figure Supplement 1-Source data 1/S6B/litong nie 2022-08-19 11h28m56s(Coomassie Blue).tif]

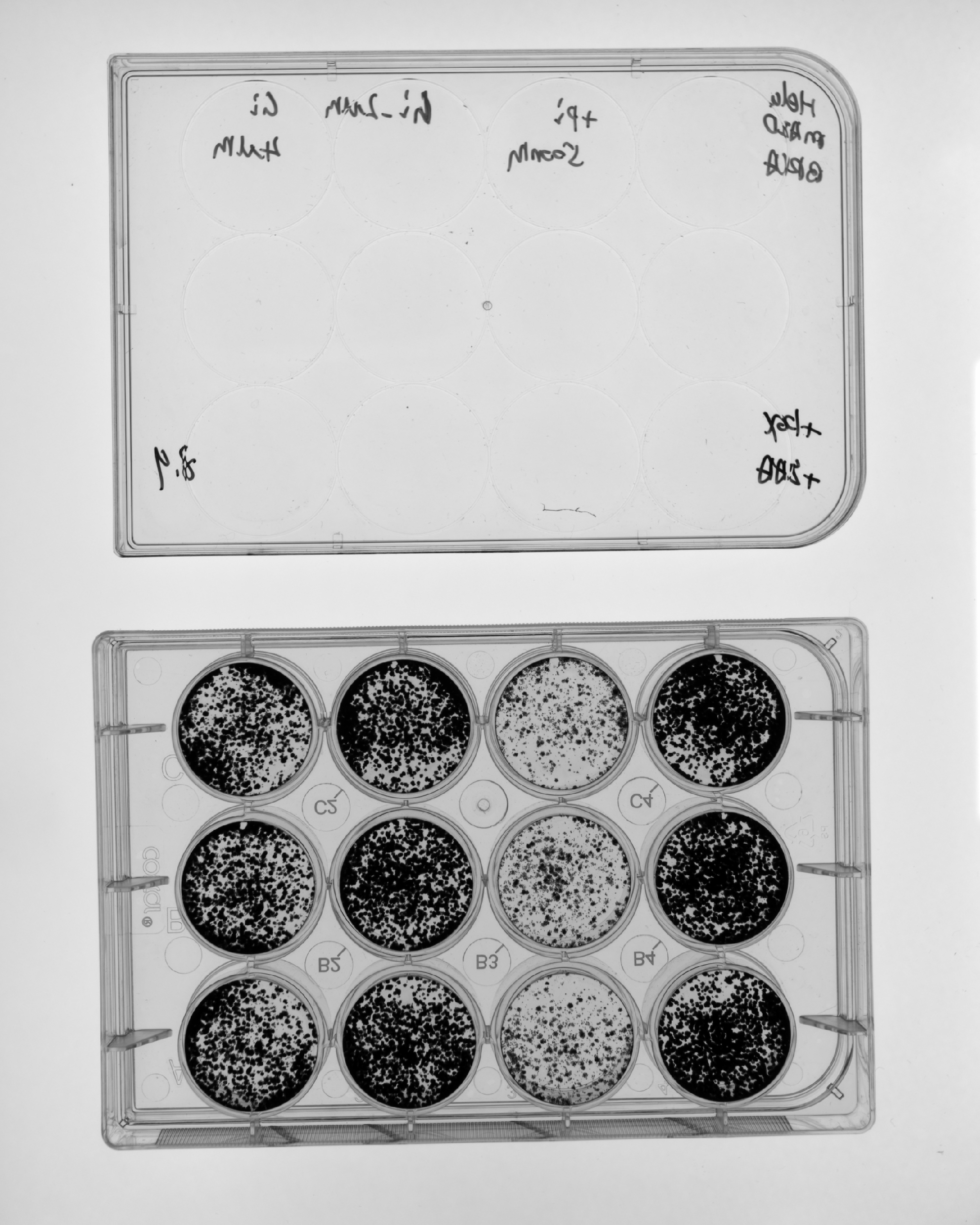

Supplement: Figure 6—figure supplement 1—source data 1. [file elife-89303-fig6-figsupp1-data1.zip › Figure 6-Figure Supplement 1-Source data 1/S6B/litong nie 2022-08-19 11h29m53s(Coomassie Blue).tif]

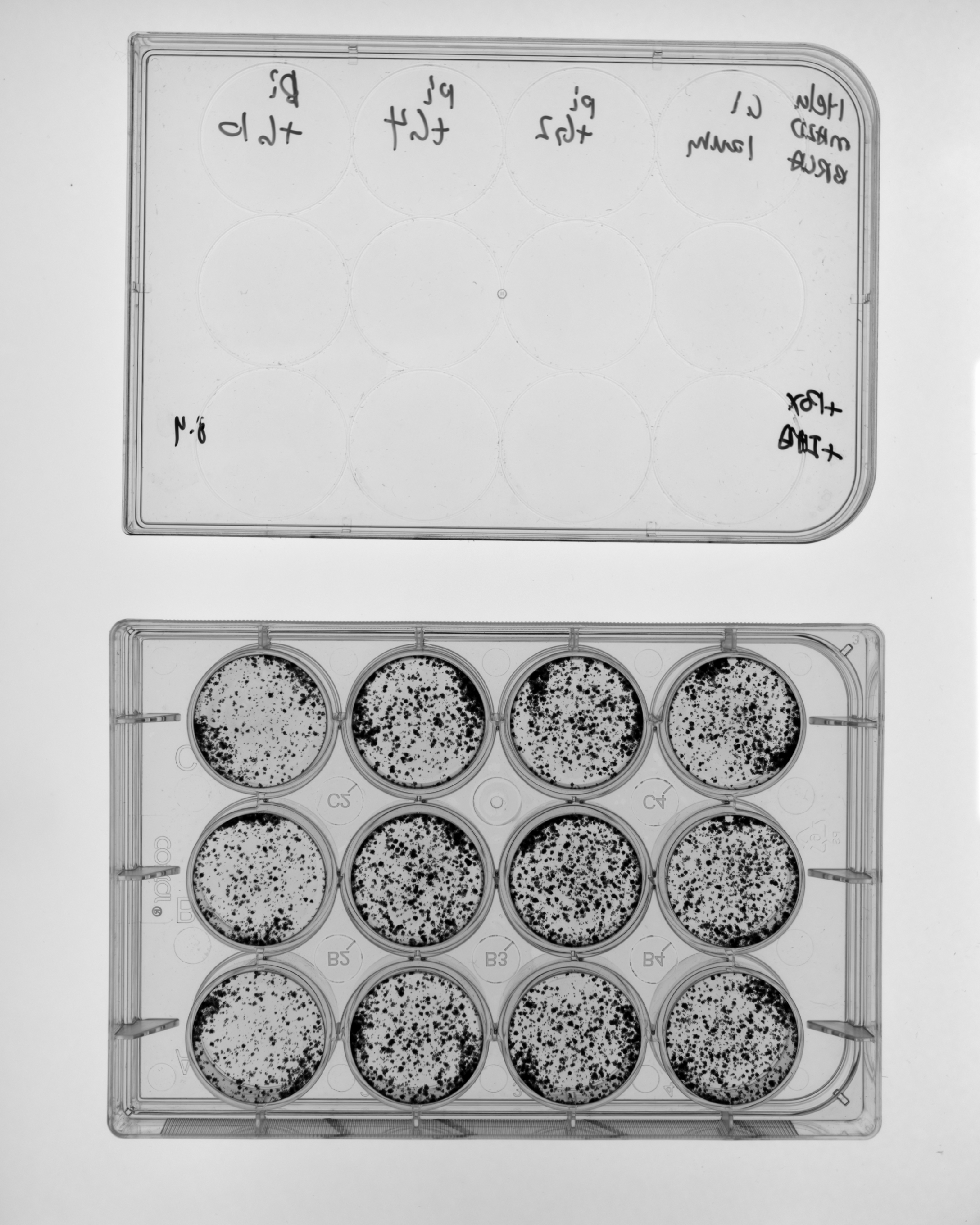

Supplement: Figure 6—figure supplement 1—source data 1. [file elife-89303-fig6-figsupp1-data1.zip › Figure 6-Figure Supplement 1-Source data 1/S6B/litong nie 2022-08-19 11h30m52s(Coomassie Blue).tif]

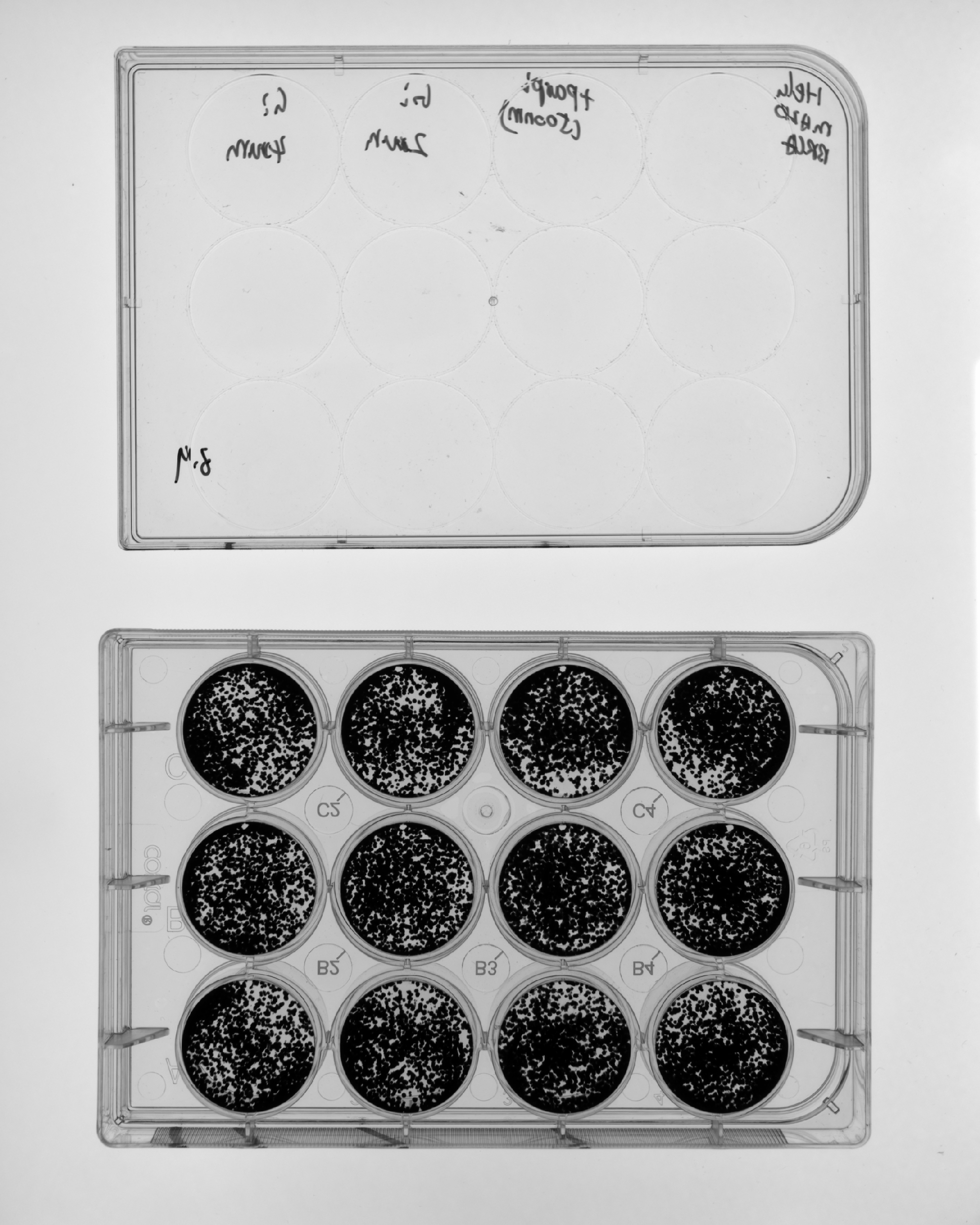

Supplement: Figure 6—figure supplement 1—source data 1. [file elife-89303-fig6-figsupp1-data1.zip › Figure 6-Figure Supplement 1-Source data 1/S6B/litong nie 2022-08-19 11h31m52s(Coomassie Blue).tif]

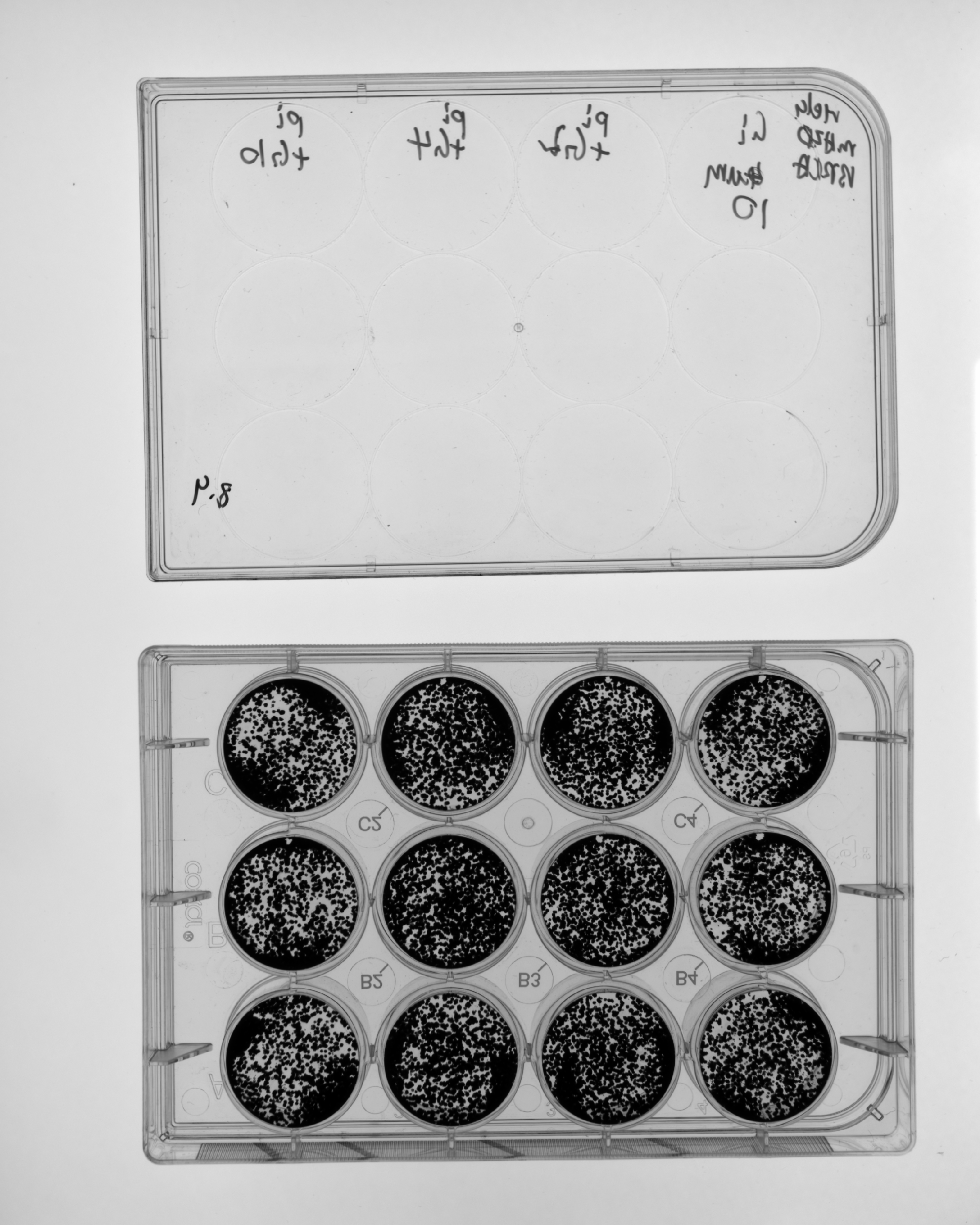

Supplement: Figure 6—figure supplement 1—source data 1. [file elife-89303-fig6-figsupp1-data1.zip › Figure 6-Figure Supplement 1-Source data 1/S6B/litong nie 2022-08-19 11h32m49s(Coomassie Blue).tif]

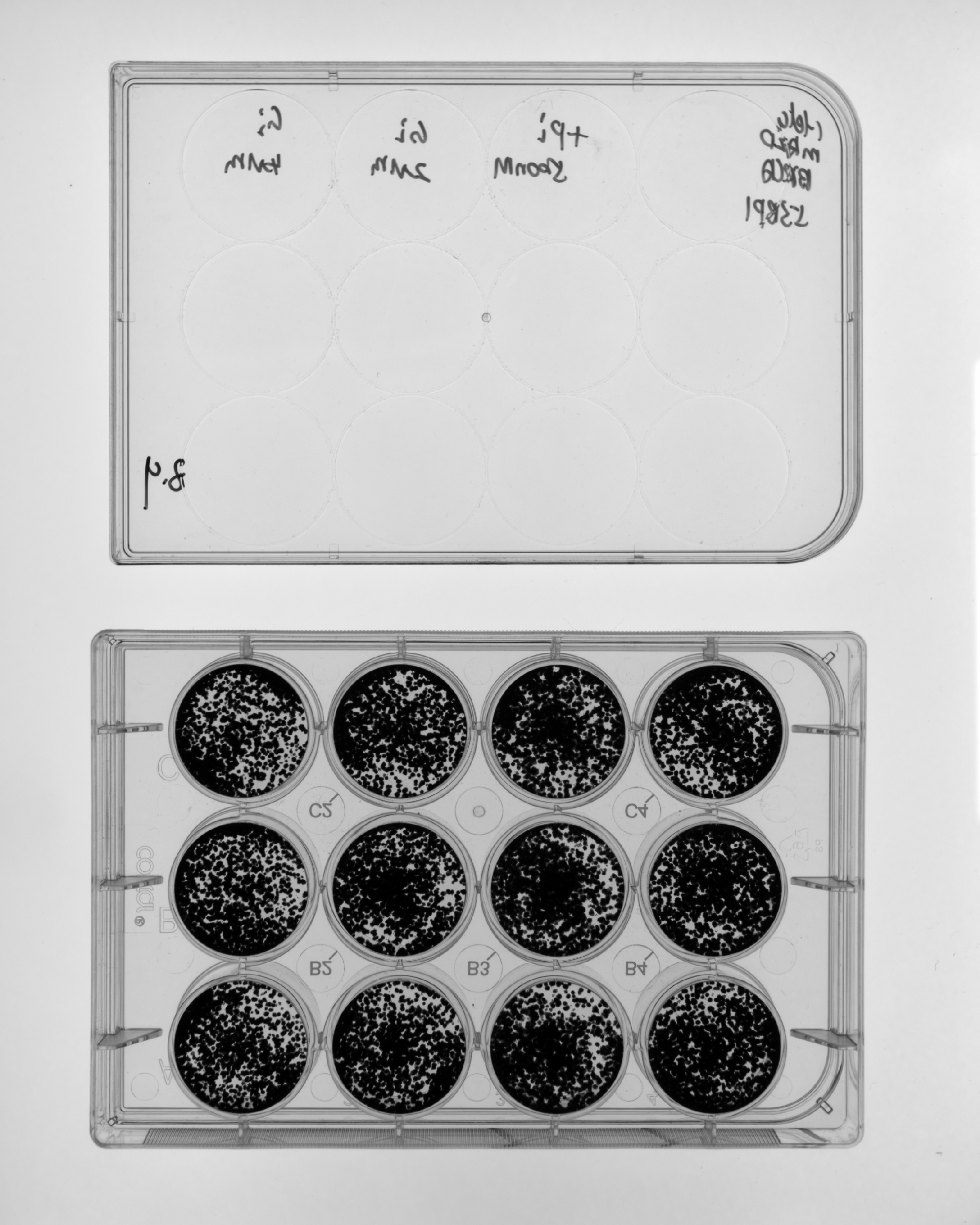

Supplement: Figure 6—figure supplement 1—source data 1. [file elife-89303-fig6-figsupp1-data1.zip › Figure 6-Figure Supplement 1-Source data 1/S6B/litong nie 2022-08-19 11h33m48s(Coomassie Blue).tif]

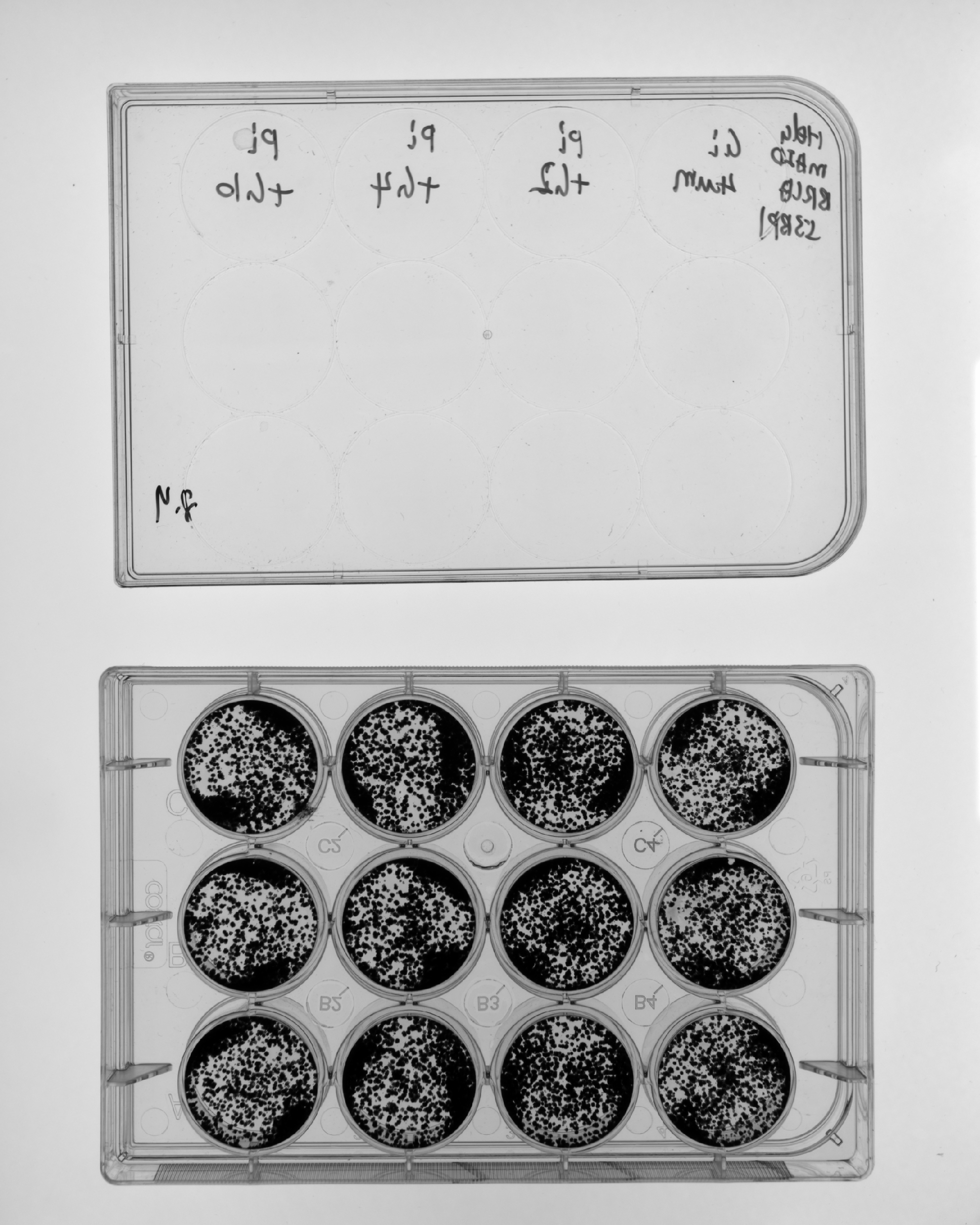

Supplement: Figure 6—figure supplement 1—source data 1. [file elife-89303-fig6-figsupp1-data1.zip › Figure 6-Figure Supplement 1-Source data 1/S6B/litong nie 2022-08-19 11h34m46s(Coomassie Blue).tif]

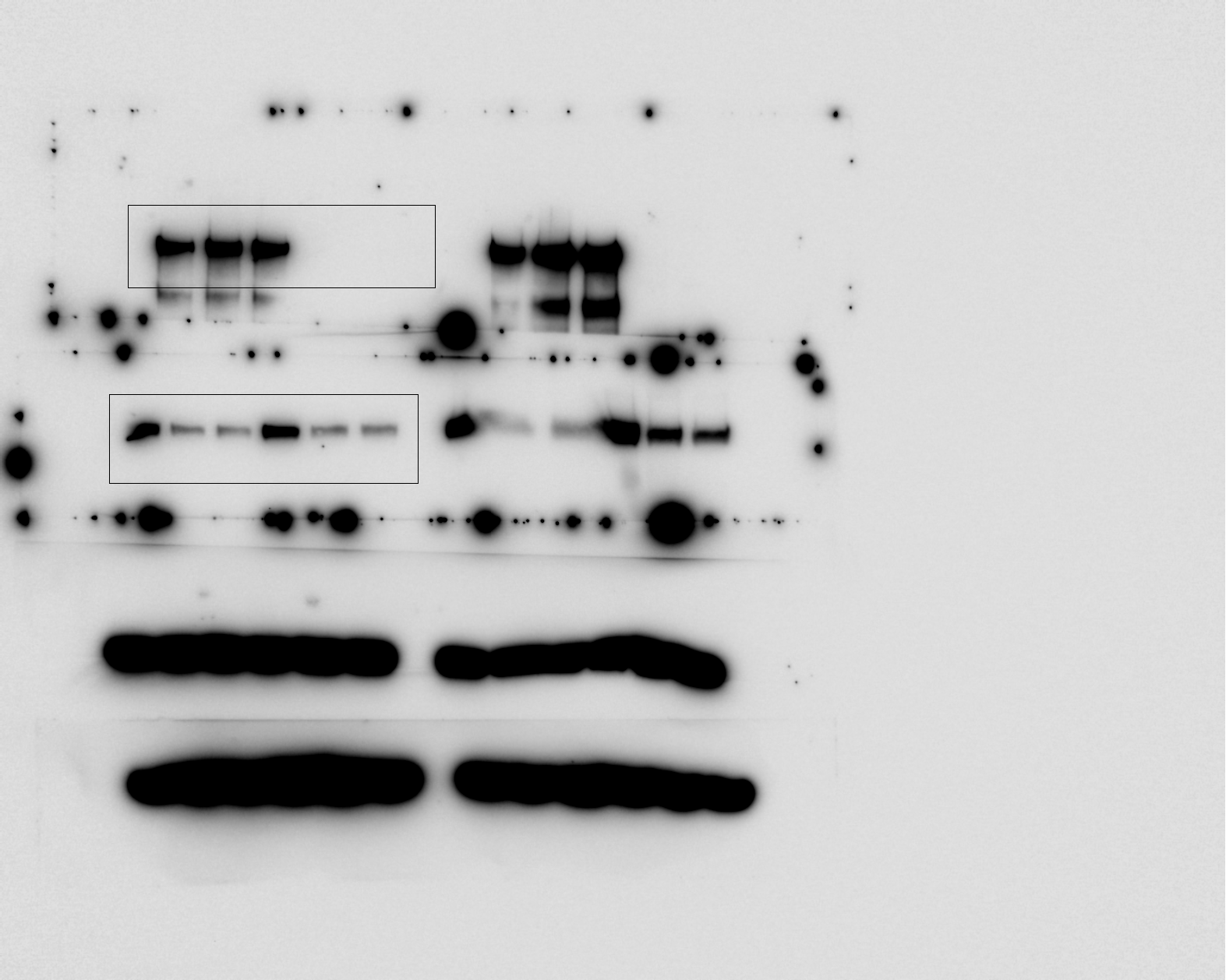

Supplement: Figure 6—figure supplement 2—source data 1. [file elife-89303-fig6-figsupp2-data1.zip › Figure 6-Figure Supplement 2-Source data 1/S7A/53BP1&PARg.tif]

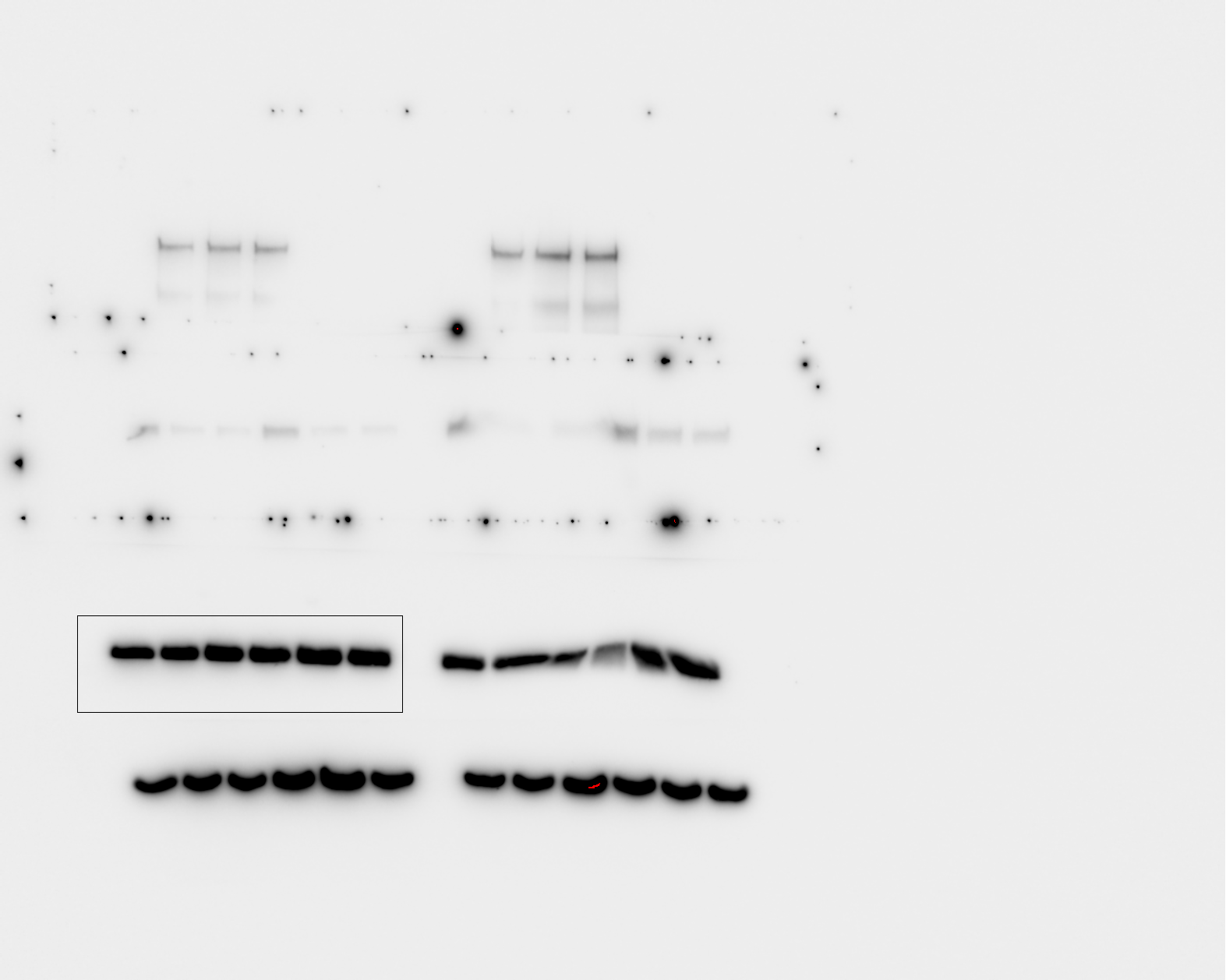

Supplement: Figure 6—figure supplement 2—source data 1. [file elife-89303-fig6-figsupp2-data1.zip › Figure 6-Figure Supplement 2-Source data 1/S7A/Tubulin.tif]

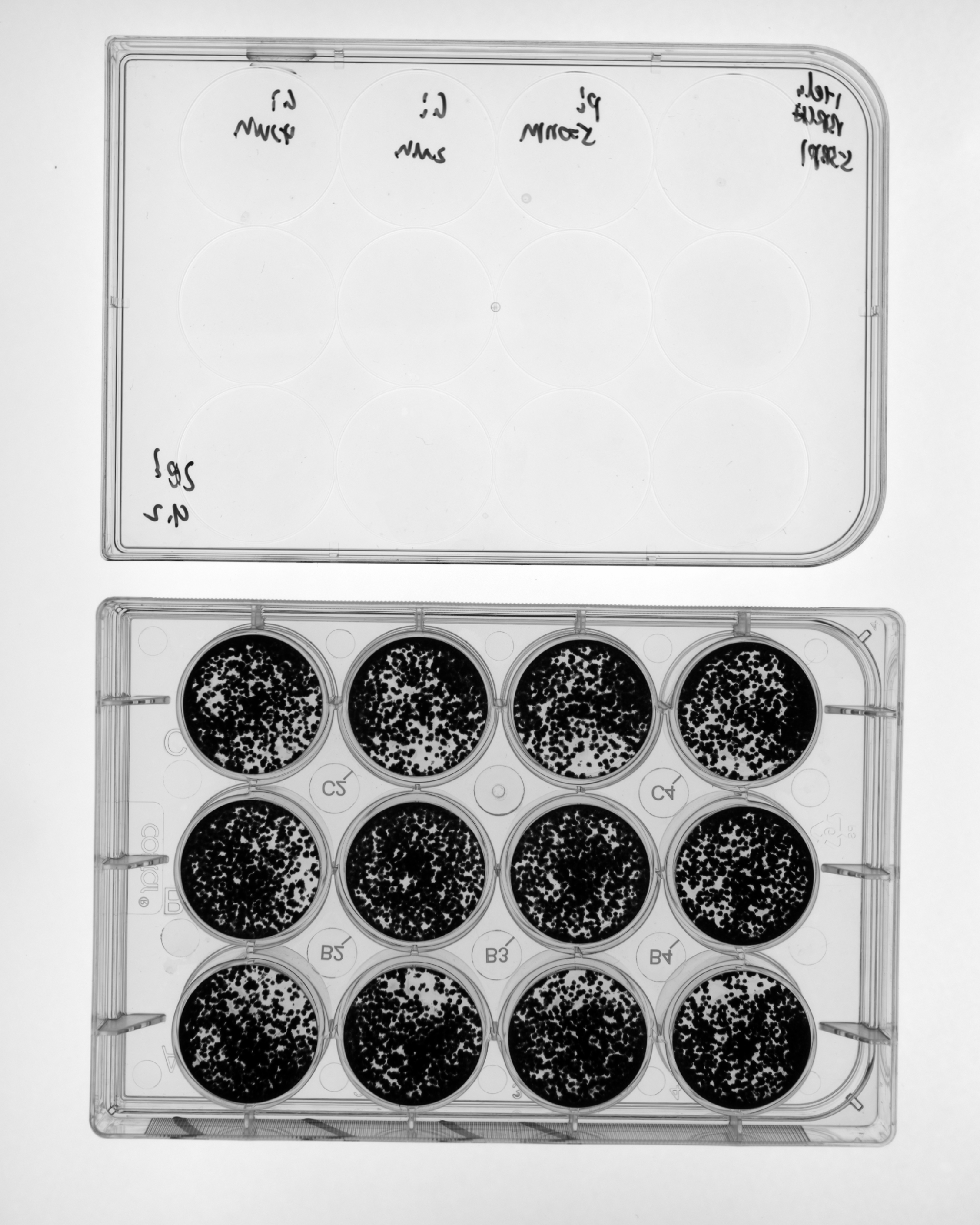

Supplement: Figure 6—figure supplement 2—source data 1. [file elife-89303-fig6-figsupp2-data1.zip › Figure 6-Figure Supplement 2-Source data 1/S7B/litong nie 2022-09-12 10h56m09s(Coomassie Blue).tif]

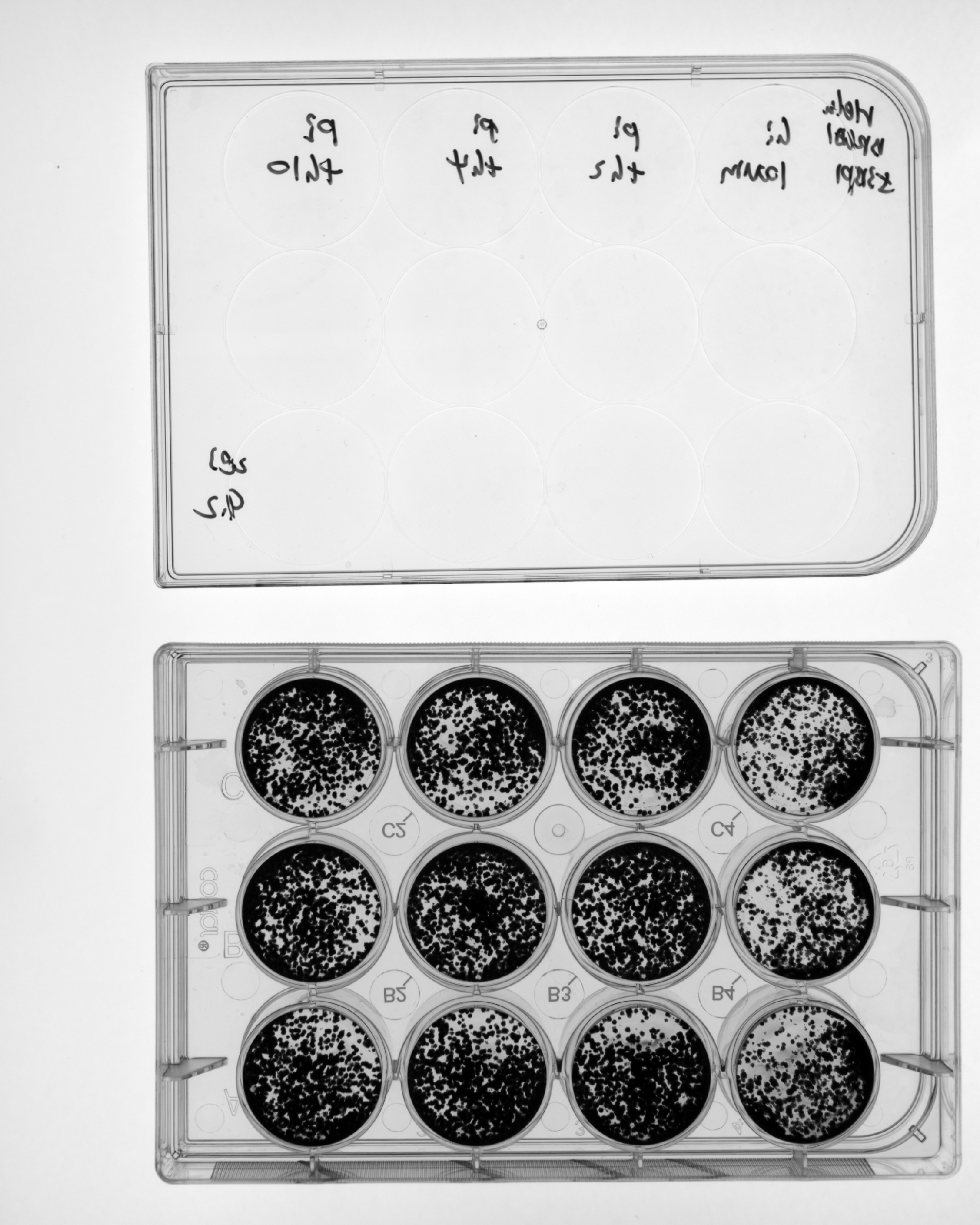

Supplement: Figure 6—figure supplement 2—source data 1. [file elife-89303-fig6-figsupp2-data1.zip › Figure 6-Figure Supplement 2-Source data 1/S7B/litong nie 2022-09-12 10h57m05s(Coomassie Blue).tif]

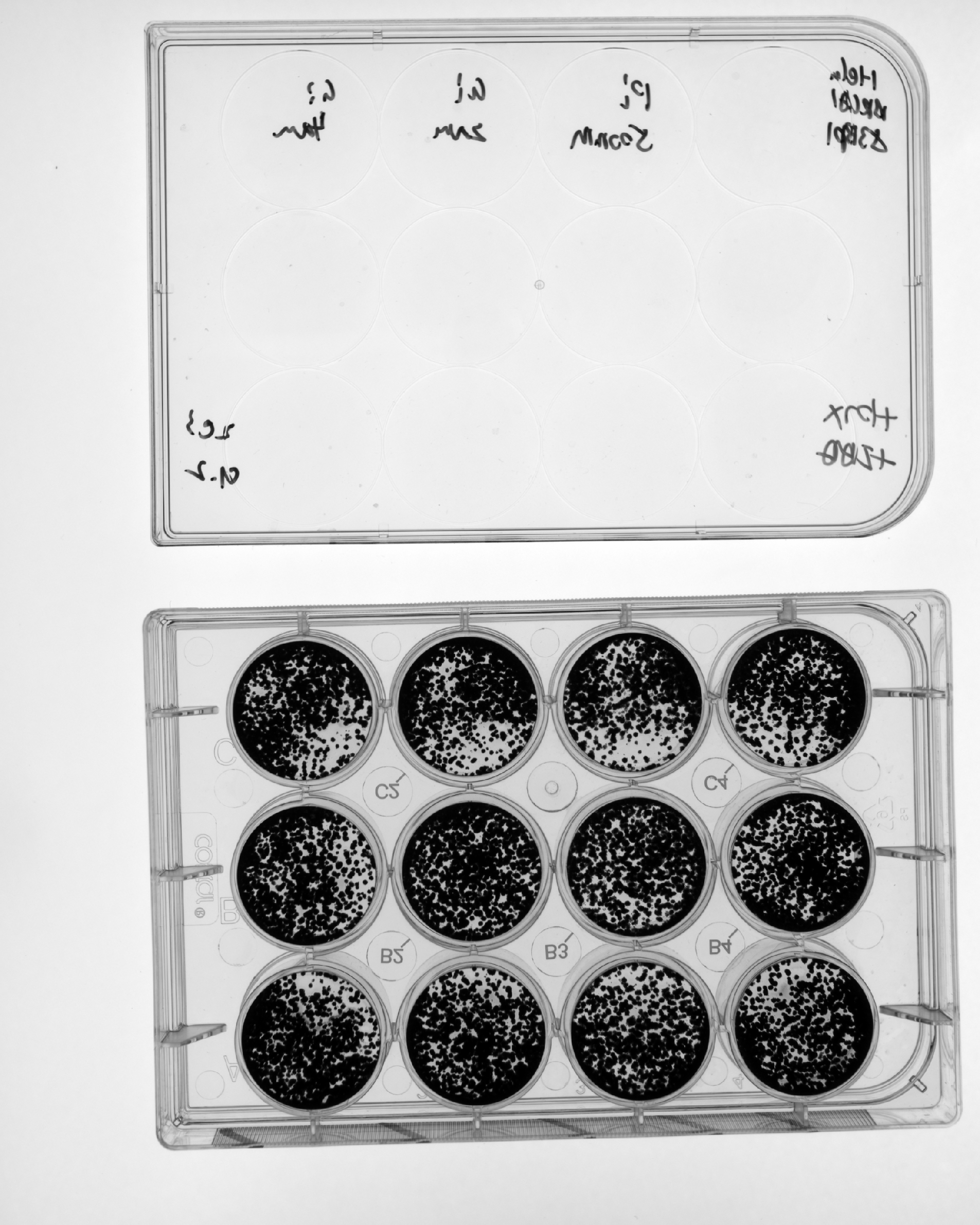

Supplement: Figure 6—figure supplement 2—source data 1. [file elife-89303-fig6-figsupp2-data1.zip › Figure 6-Figure Supplement 2-Source data 1/S7B/litong nie 2022-09-12 10h58m01s(Coomassie Blue).tif]

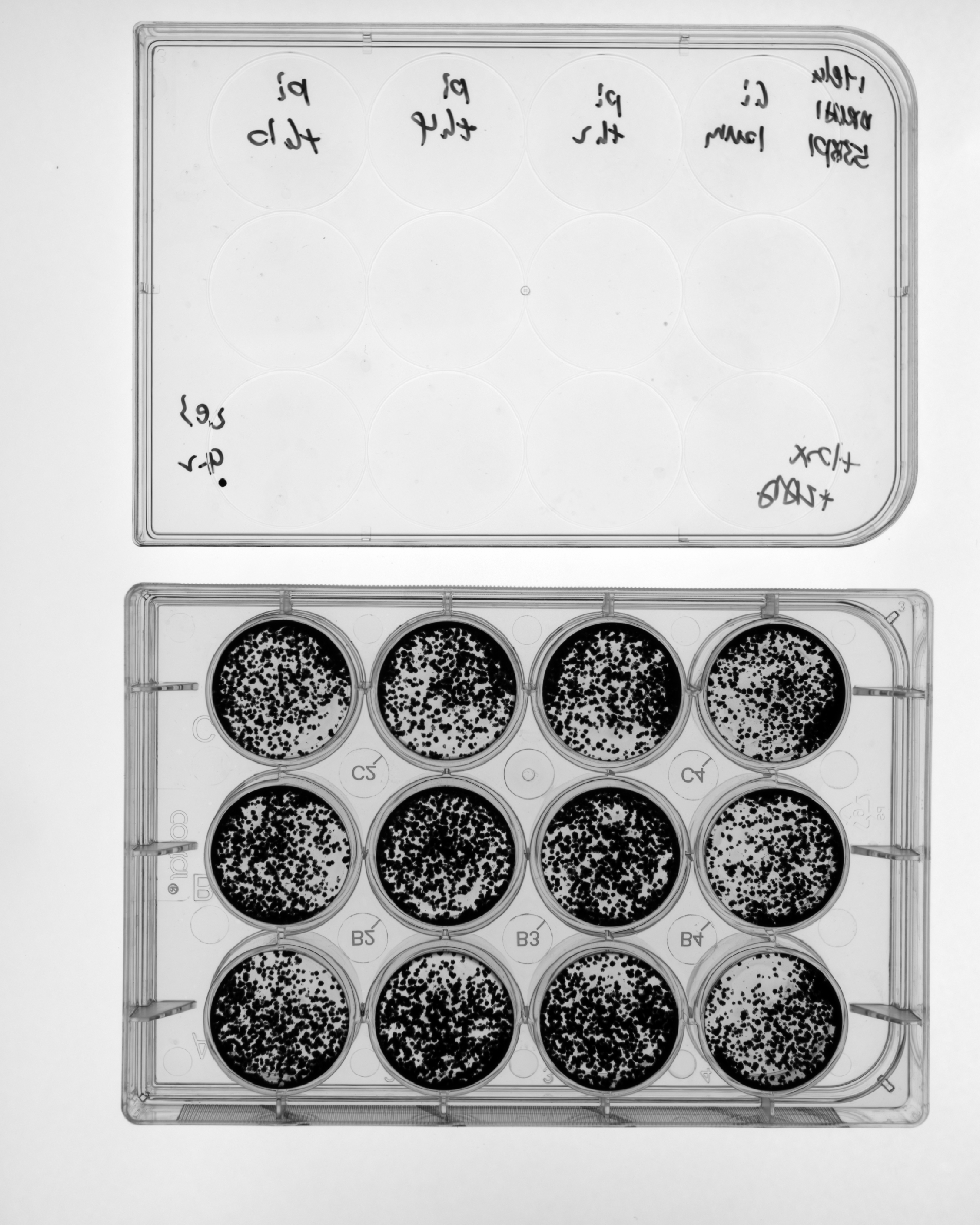

Supplement: Figure 6—figure supplement 2—source data 1. [file elife-89303-fig6-figsupp2-data1.zip › Figure 6-Figure Supplement 2-Source data 1/S7B/litong nie 2022-09-12 10h59m00s(Coomassie Blue).tif]

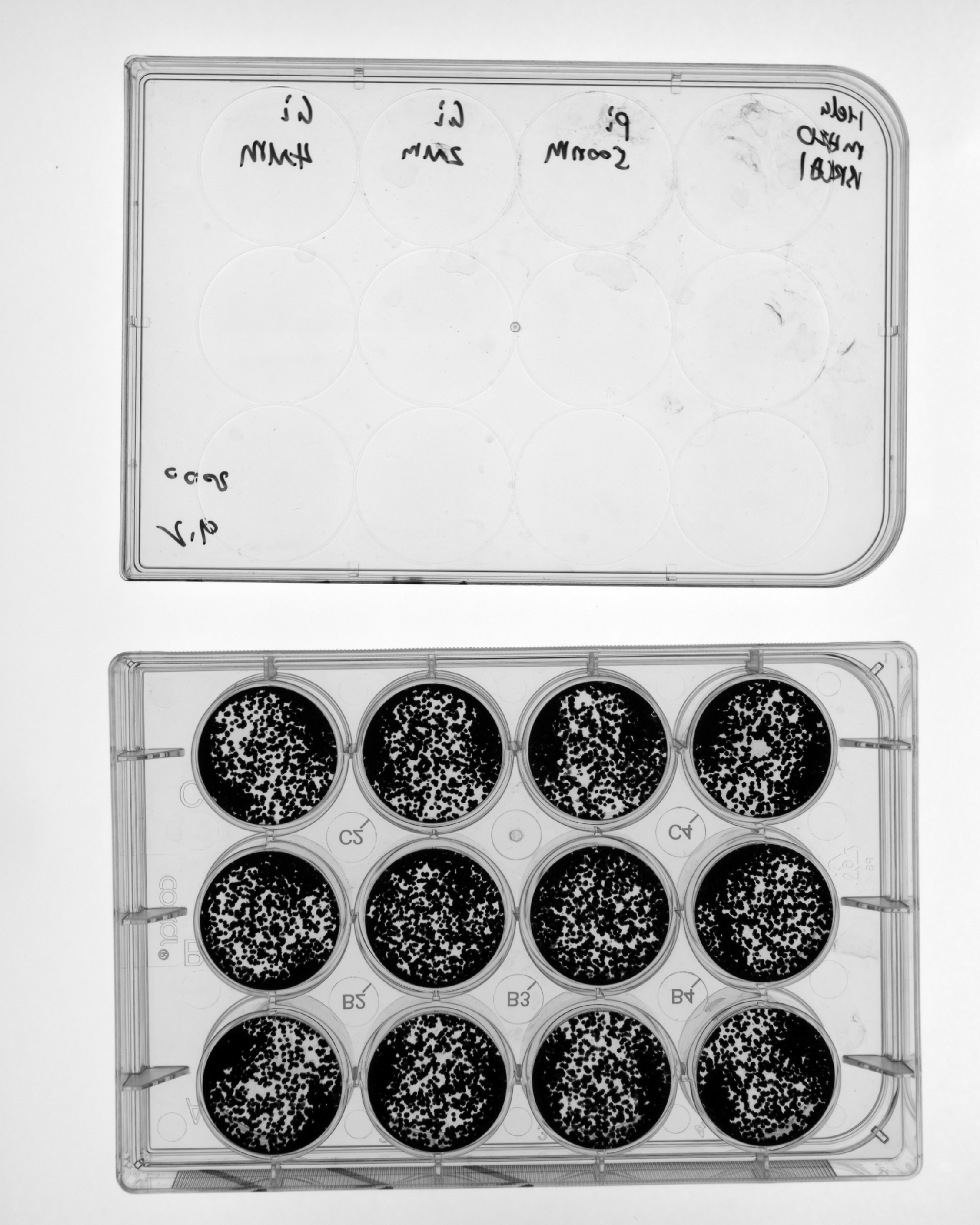

Supplement: Figure 6—figure supplement 2—source data 1. [file elife-89303-fig6-figsupp2-data1.zip › Figure 6-Figure Supplement 2-Source data 1/S7B/litong nie 2022-09-12 11h00m18s(Coomassie Blue).tif]

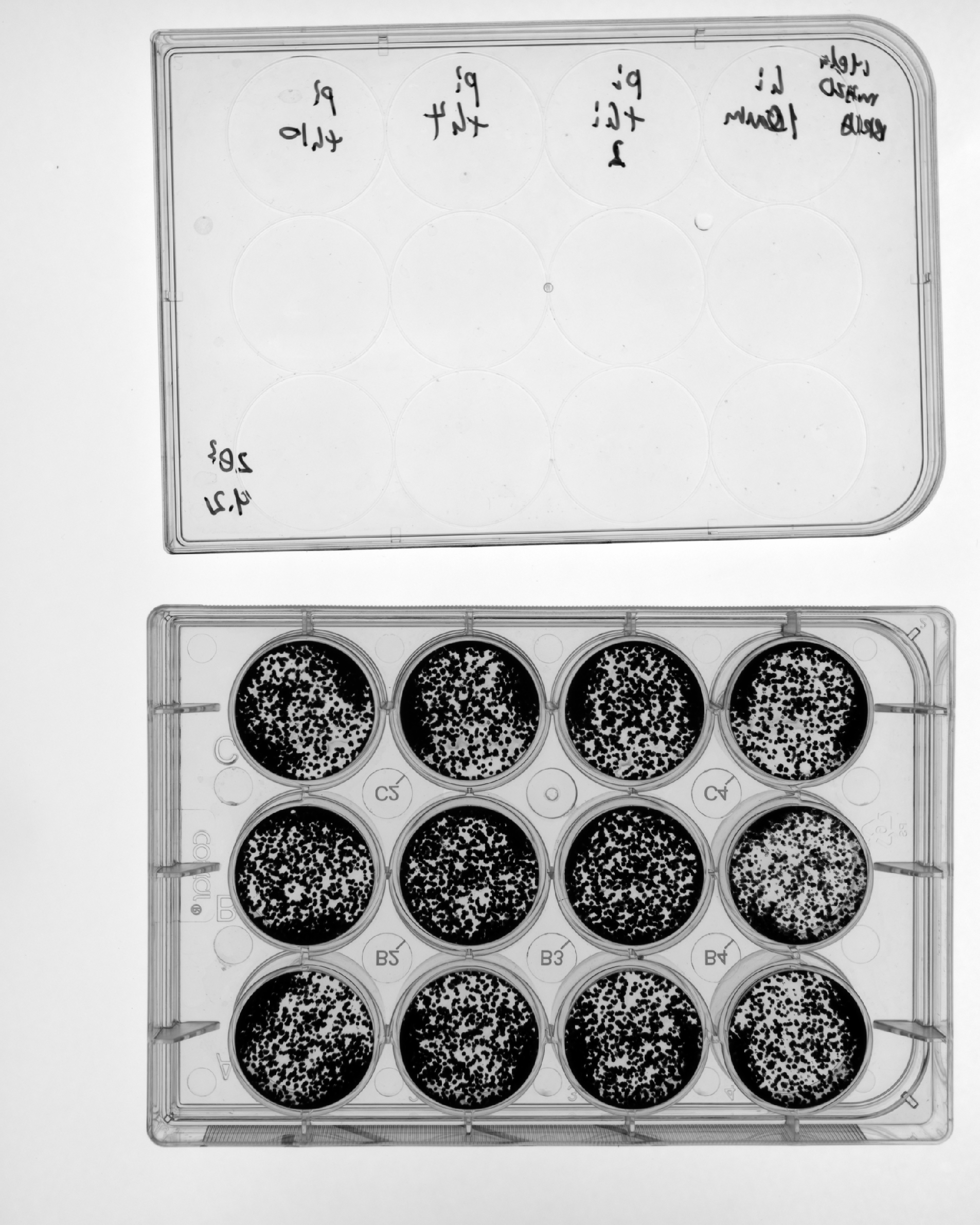

Supplement: Figure 6—figure supplement 2—source data 1. [file elife-89303-fig6-figsupp2-data1.zip › Figure 6-Figure Supplement 2-Source data 1/S7B/litong nie 2022-09-12 11h01m35s(Coomassie Blue).tif]

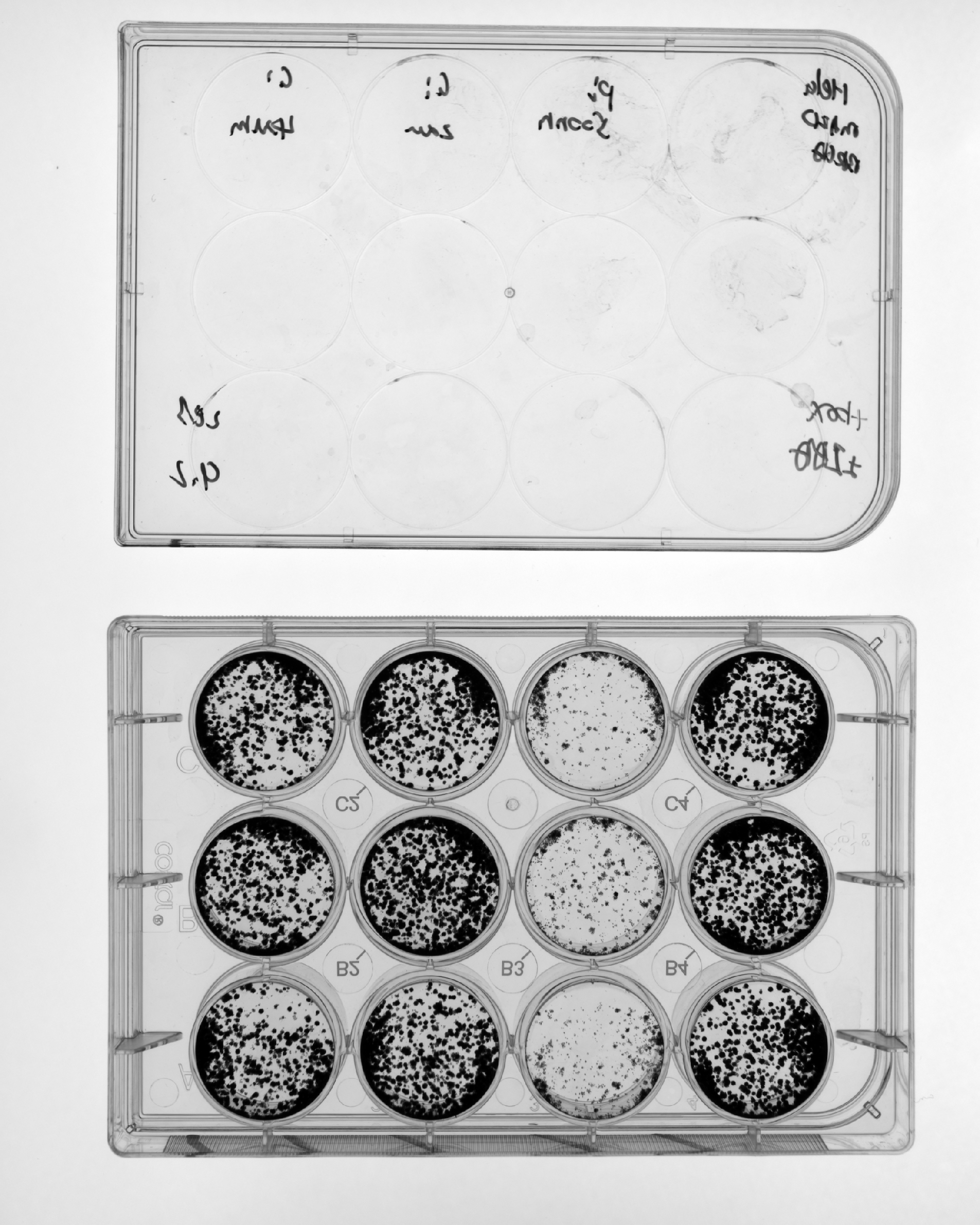

Supplement: Figure 6—figure supplement 2—source data 1. [file elife-89303-fig6-figsupp2-data1.zip › Figure 6-Figure Supplement 2-Source data 1/S7B/litong nie 2022-09-12 11h02m42s(Coomassie Blue).tif]

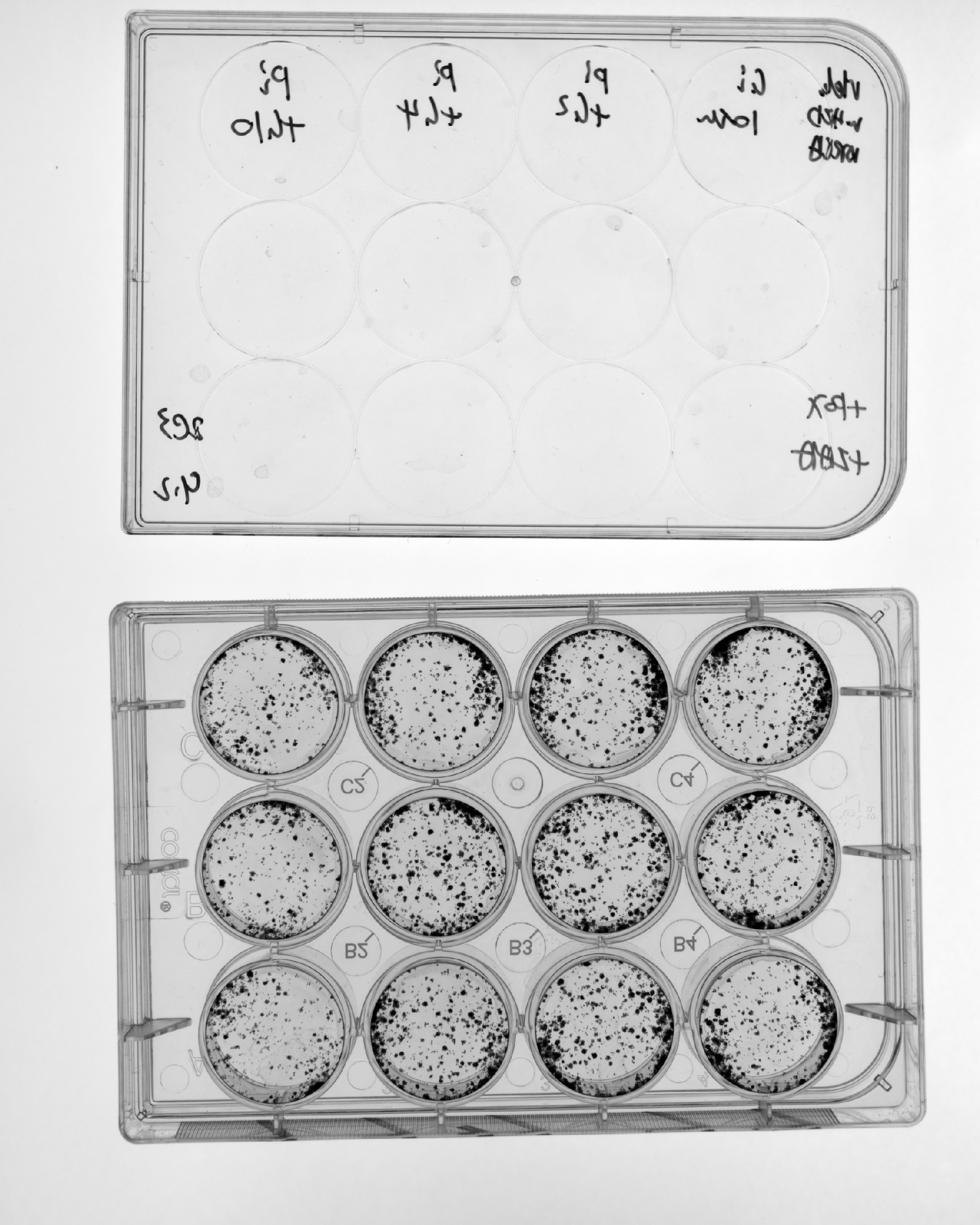

Supplement: Figure 6—figure supplement 2—source data 1. [file elife-89303-fig6-figsupp2-data1.zip › Figure 6-Figure Supplement 2-Source data 1/S7B/litong nie 2022-09-12 11h03m47s(Coomassie Blue).tif]

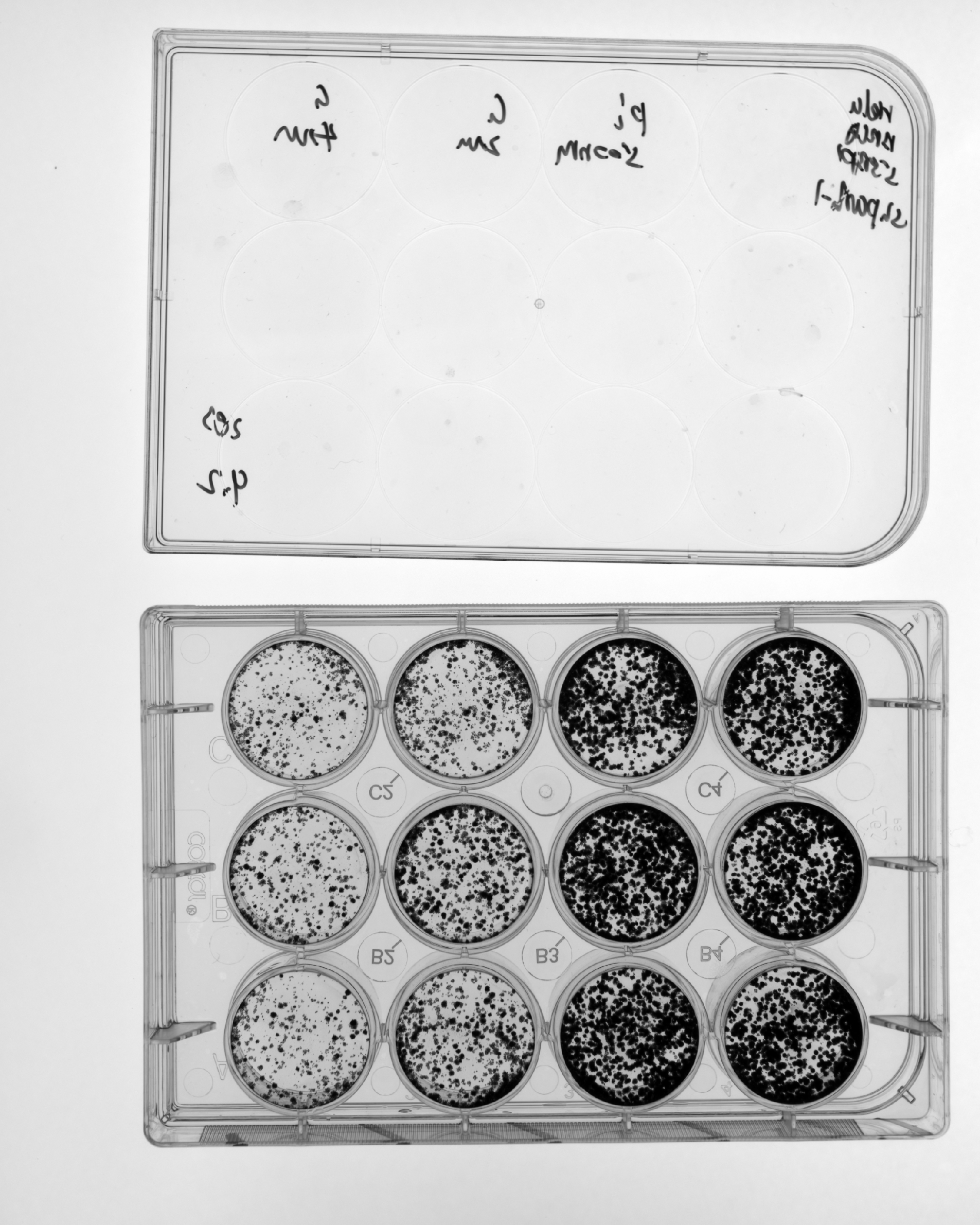

Supplement: Figure 6—figure supplement 2—source data 1. [file elife-89303-fig6-figsupp2-data1.zip › Figure 6-Figure Supplement 2-Source data 1/S7B/litong nie 2022-09-12 11h05m01s(Coomassie Blue).tif]

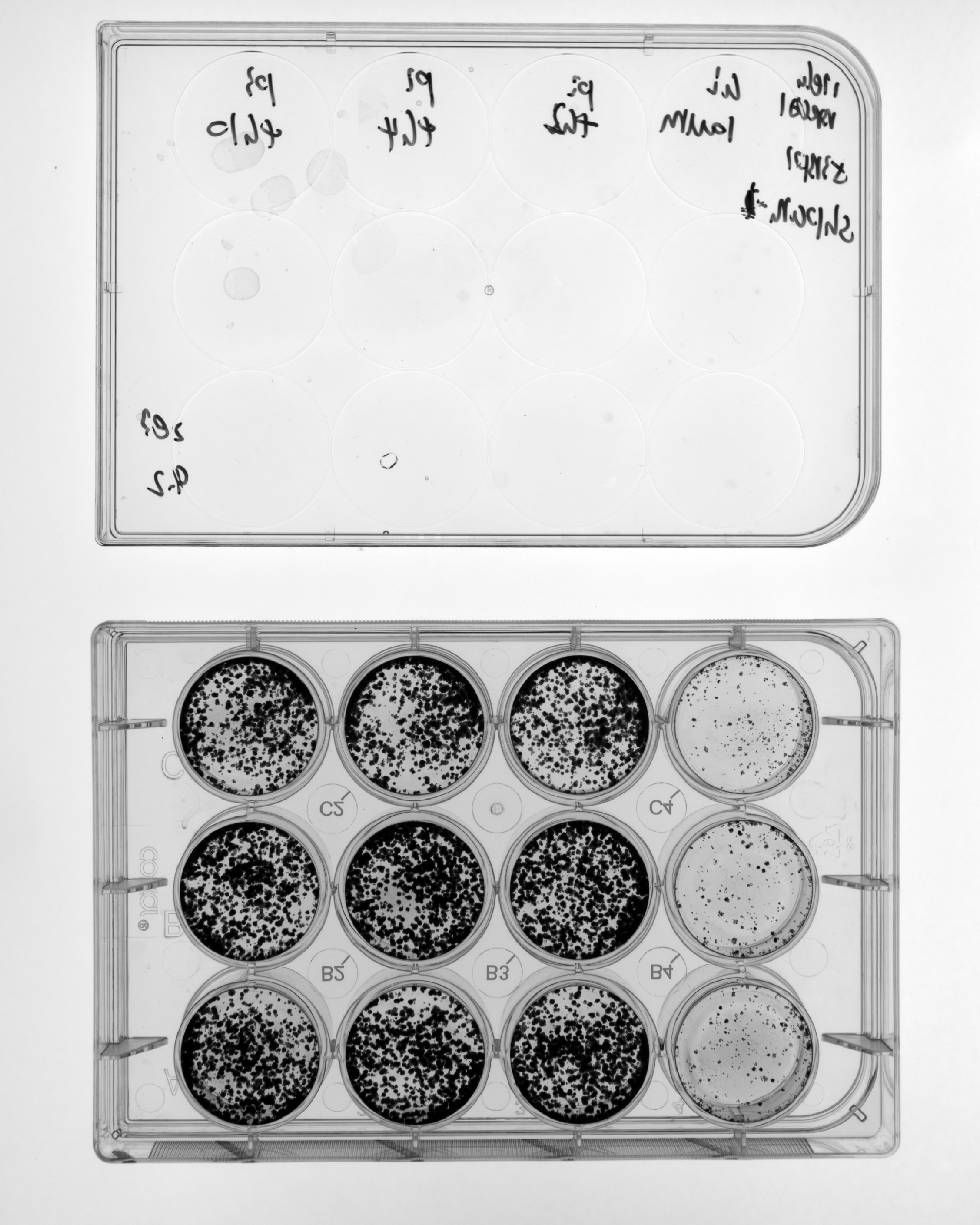

Supplement: Figure 6—figure supplement 2—source data 1. [file elife-89303-fig6-figsupp2-data1.zip › Figure 6-Figure Supplement 2-Source data 1/S7B/litong nie 2022-09-12 11h06m21s(Coomassie Blue).tif]

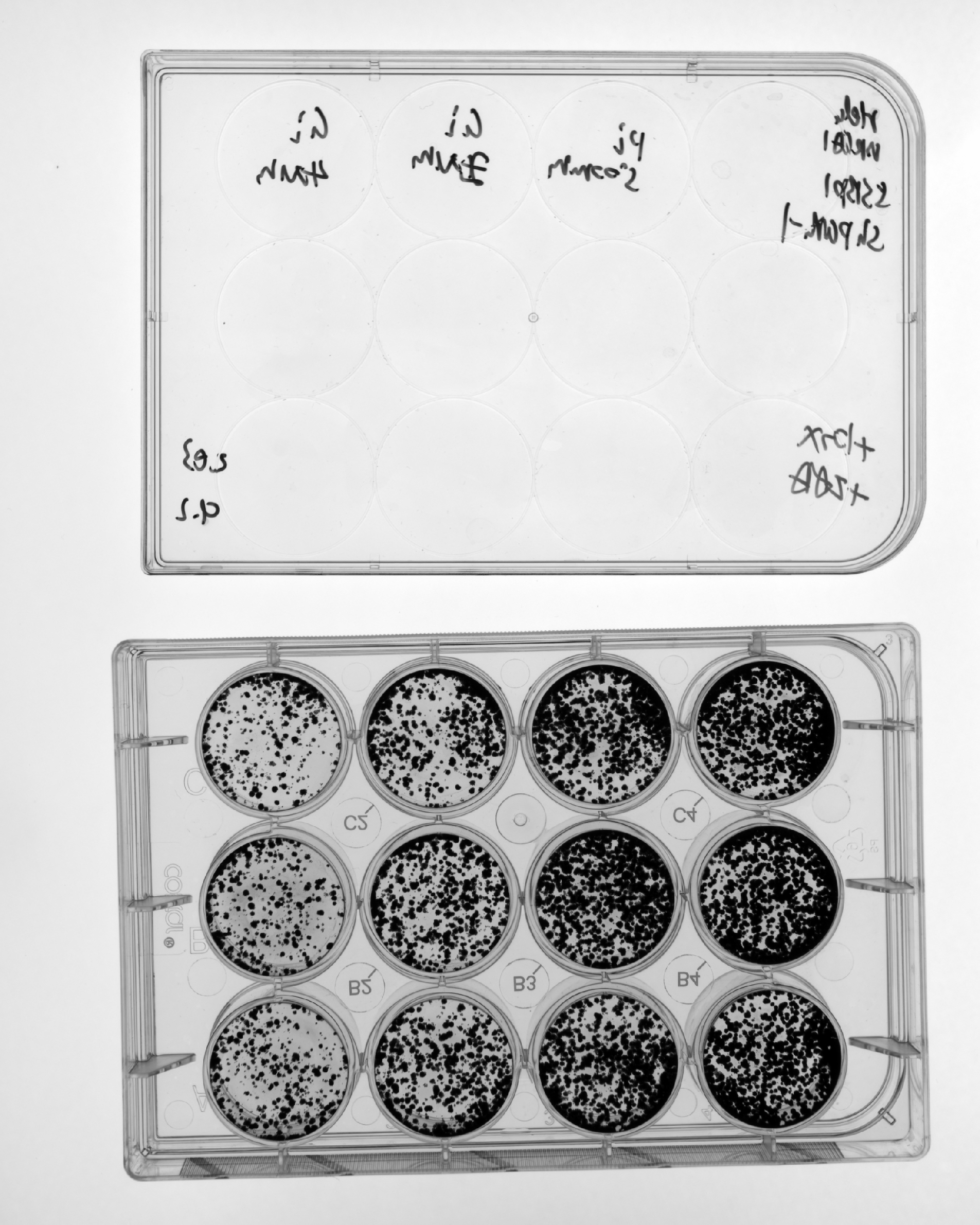

Supplement: Figure 6—figure supplement 2—source data 1. [file elife-89303-fig6-figsupp2-data1.zip › Figure 6-Figure Supplement 2-Source data 1/S7B/litong nie 2022-09-12 11h07m19s(Coomassie Blue).tif]

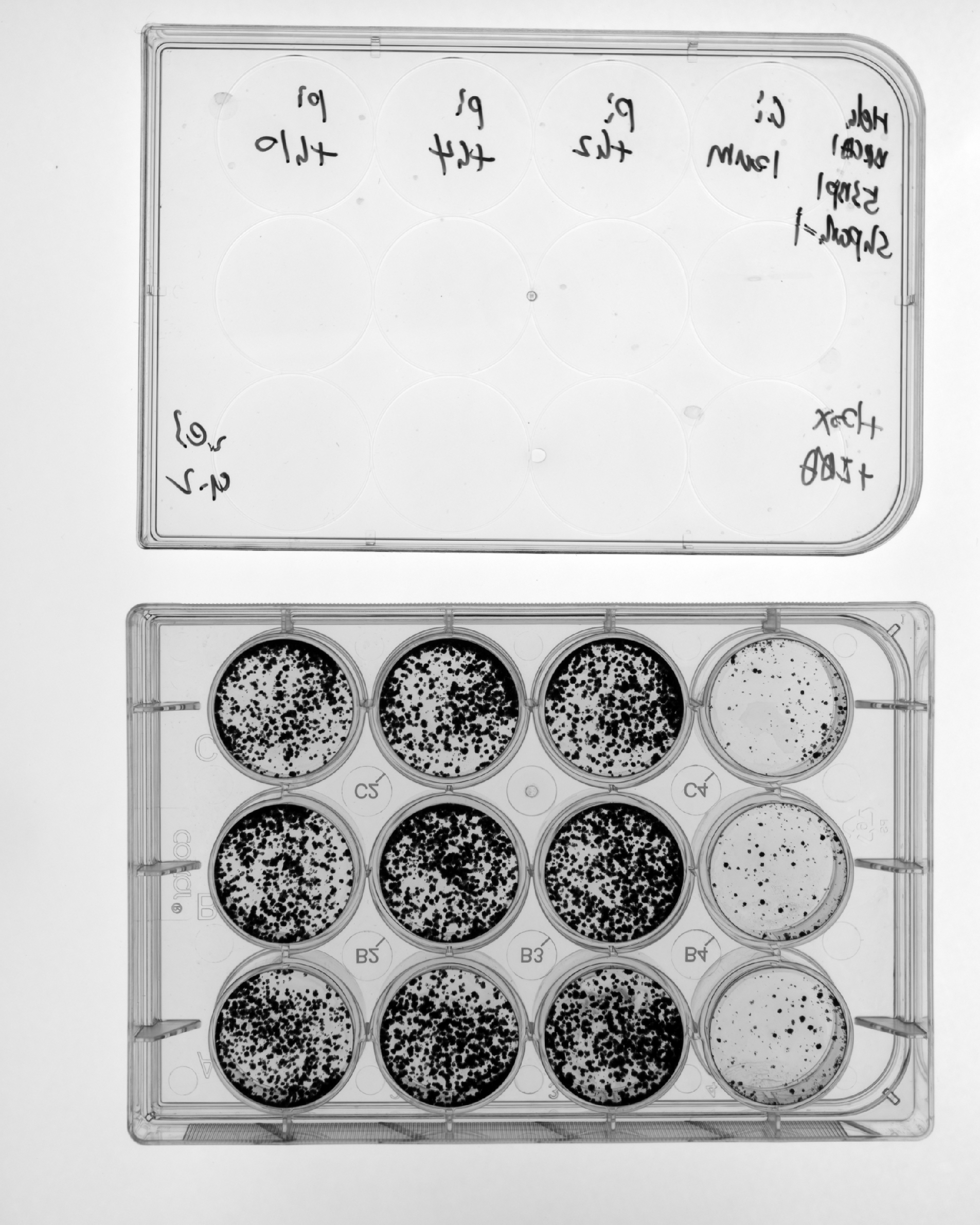

Supplement: Figure 6—figure supplement 2—source data 1. [file elife-89303-fig6-figsupp2-data1.zip › Figure 6-Figure Supplement 2-Source data 1/S7B/litong nie 2022-09-12 11h08m44s(Coomassie Blue).tif]

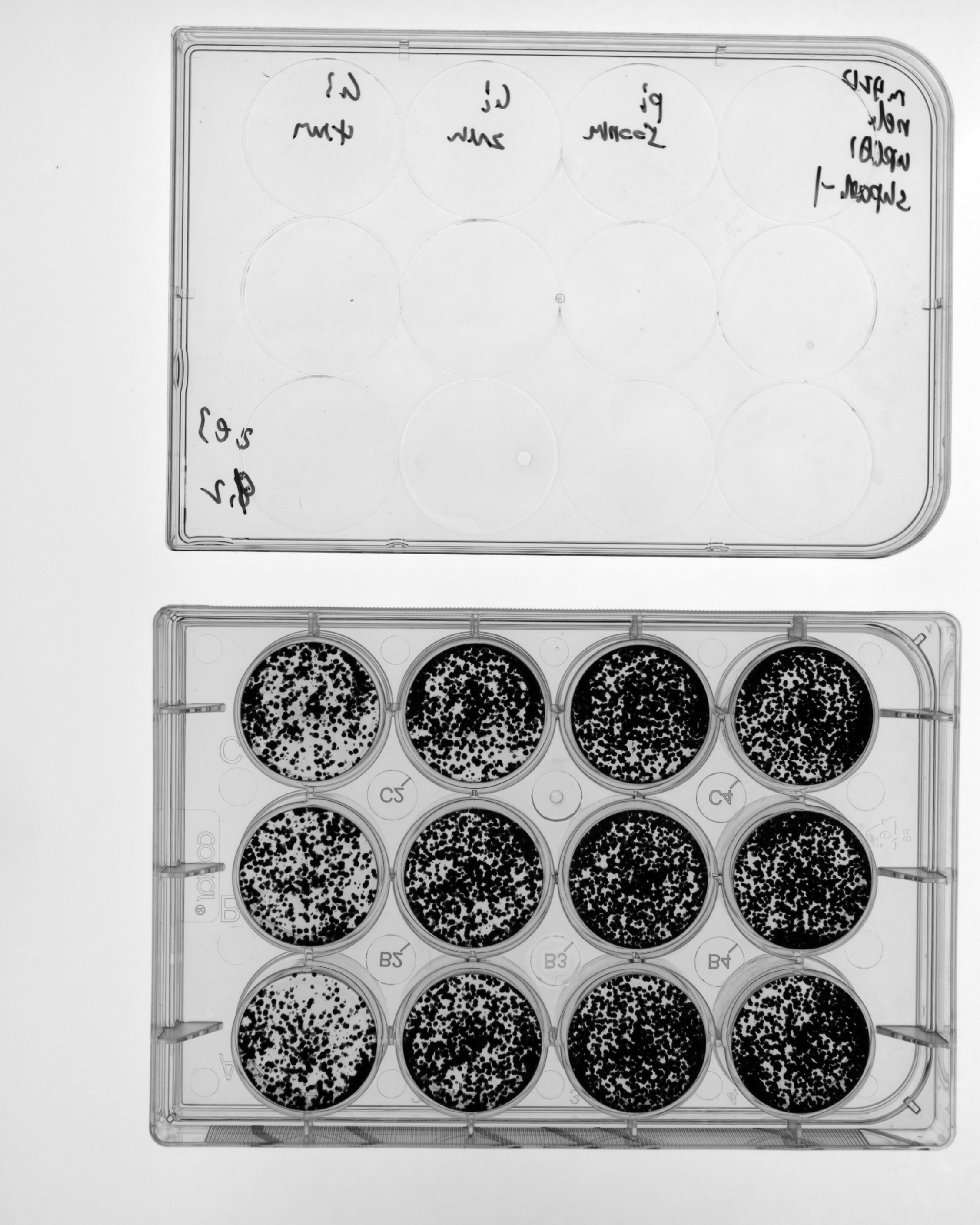

Supplement: Figure 6—figure supplement 2—source data 1. [file elife-89303-fig6-figsupp2-data1.zip › Figure 6-Figure Supplement 2-Source data 1/S7B/litong nie 2022-09-12 11h10m00s(Coomassie Blue).tif]

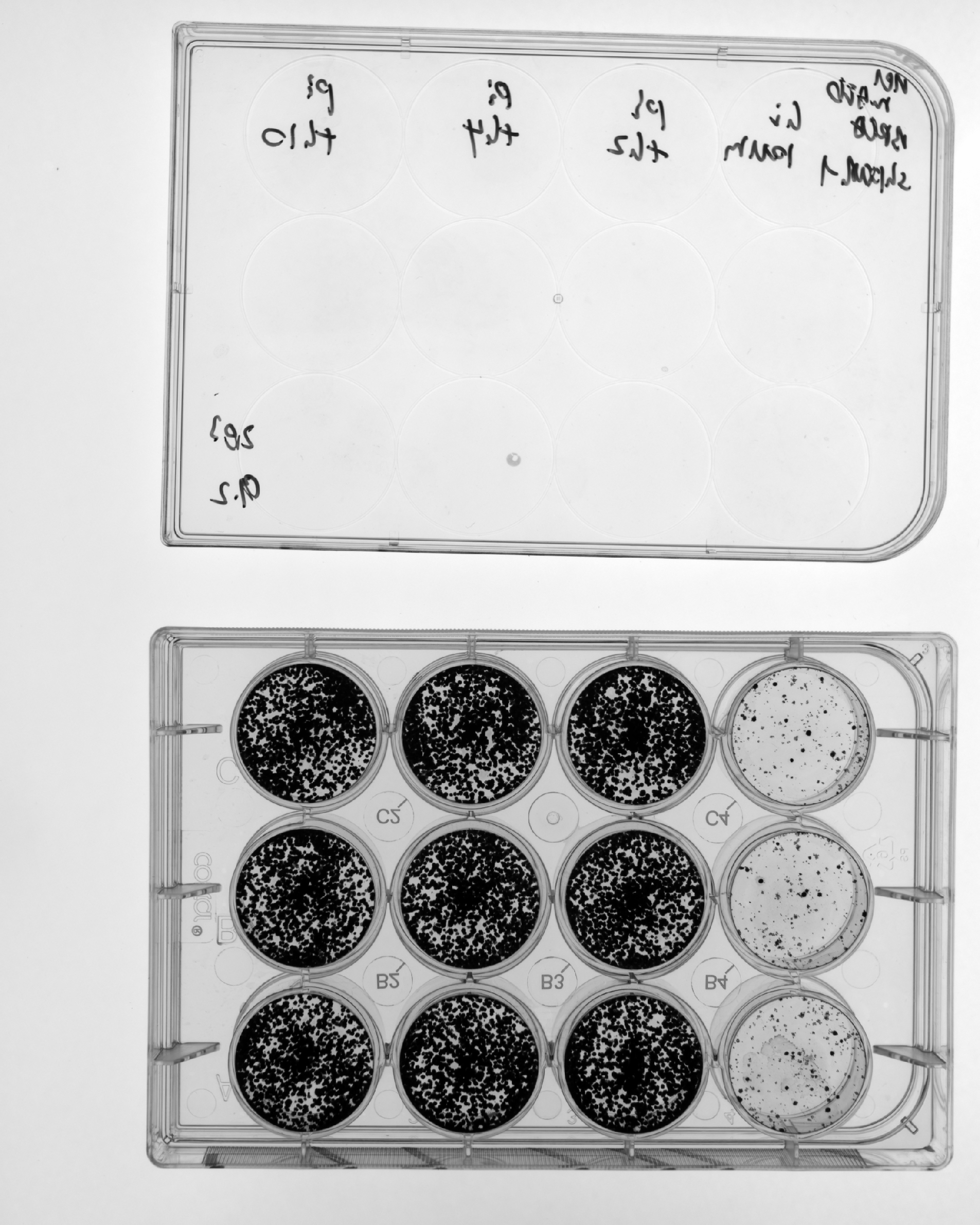

Supplement: Figure 6—figure supplement 2—source data 1. [file elife-89303-fig6-figsupp2-data1.zip › Figure 6-Figure Supplement 2-Source data 1/S7B/litong nie 2022-09-12 11h11m08s(Coomassie Blue).tif]

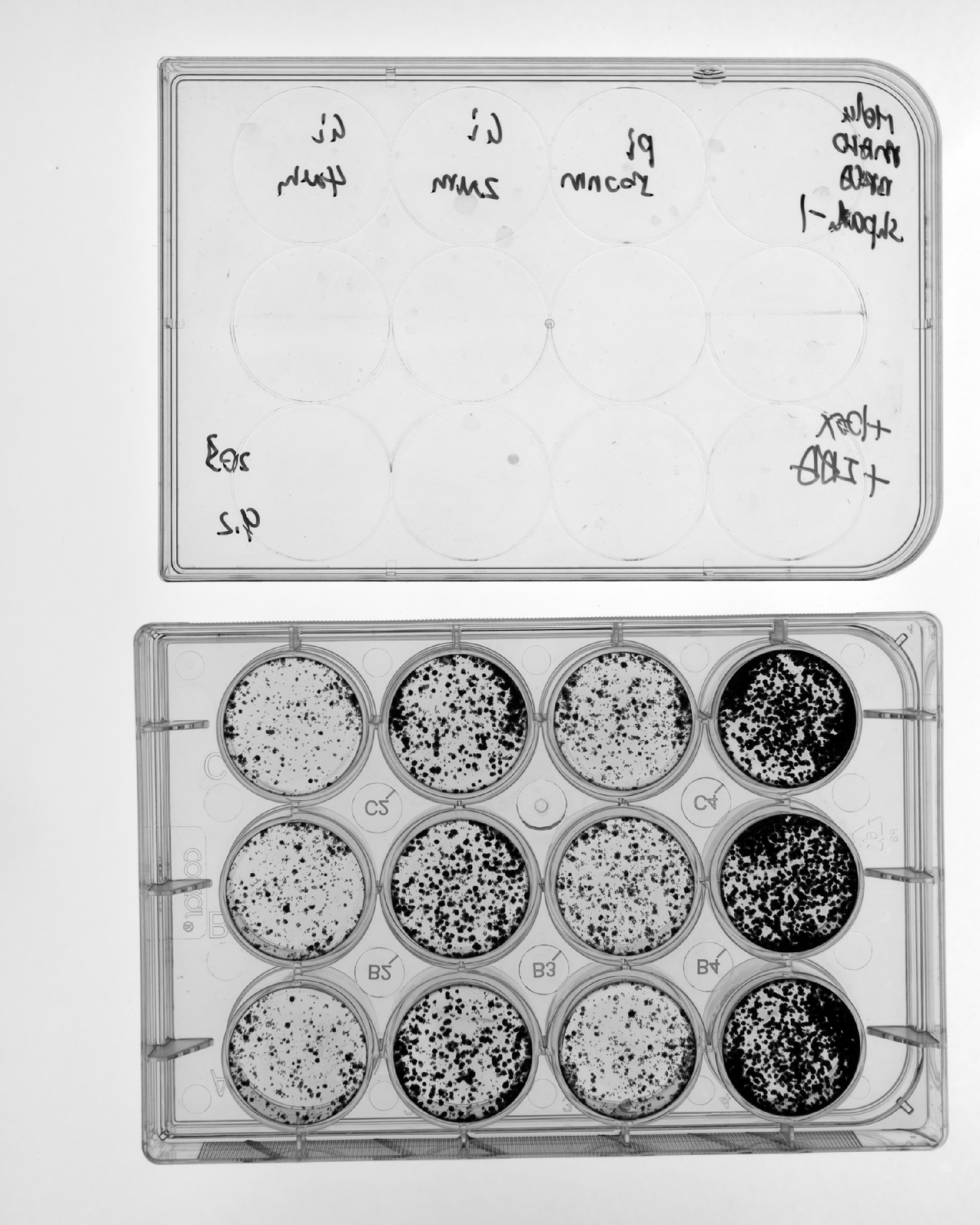

Supplement: Figure 6—figure supplement 2—source data 1. [file elife-89303-fig6-figsupp2-data1.zip › Figure 6-Figure Supplement 2-Source data 1/S7B/litong nie 2022-09-12 11h12m33s(Coomassie Blue).tif]

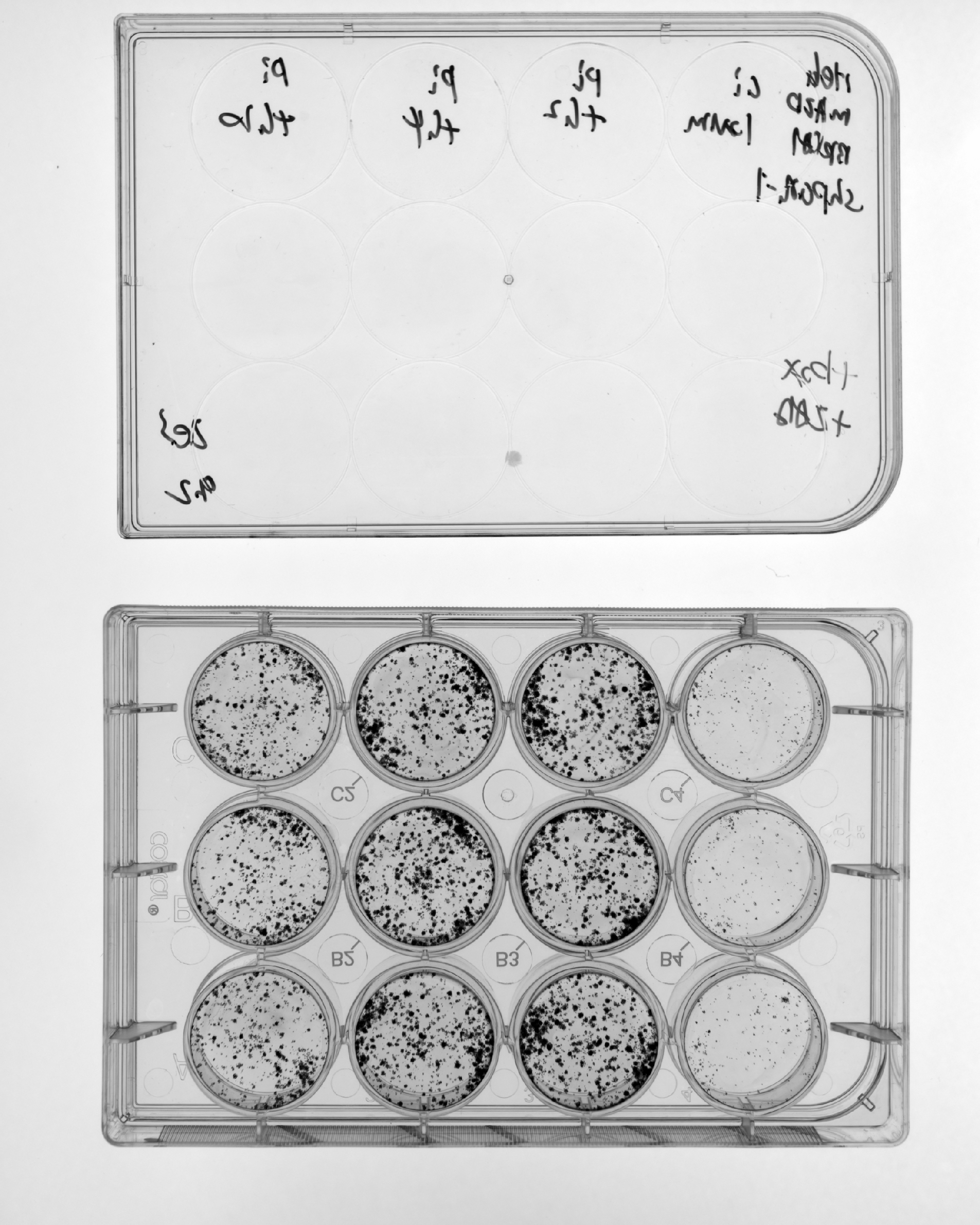

Supplement: Figure 6—figure supplement 2—source data 1. [file elife-89303-fig6-figsupp2-data1.zip › Figure 6-Figure Supplement 2-Source data 1/S7B/litong nie 2022-09-12 11h13m29s(Coomassie Blue).tif]

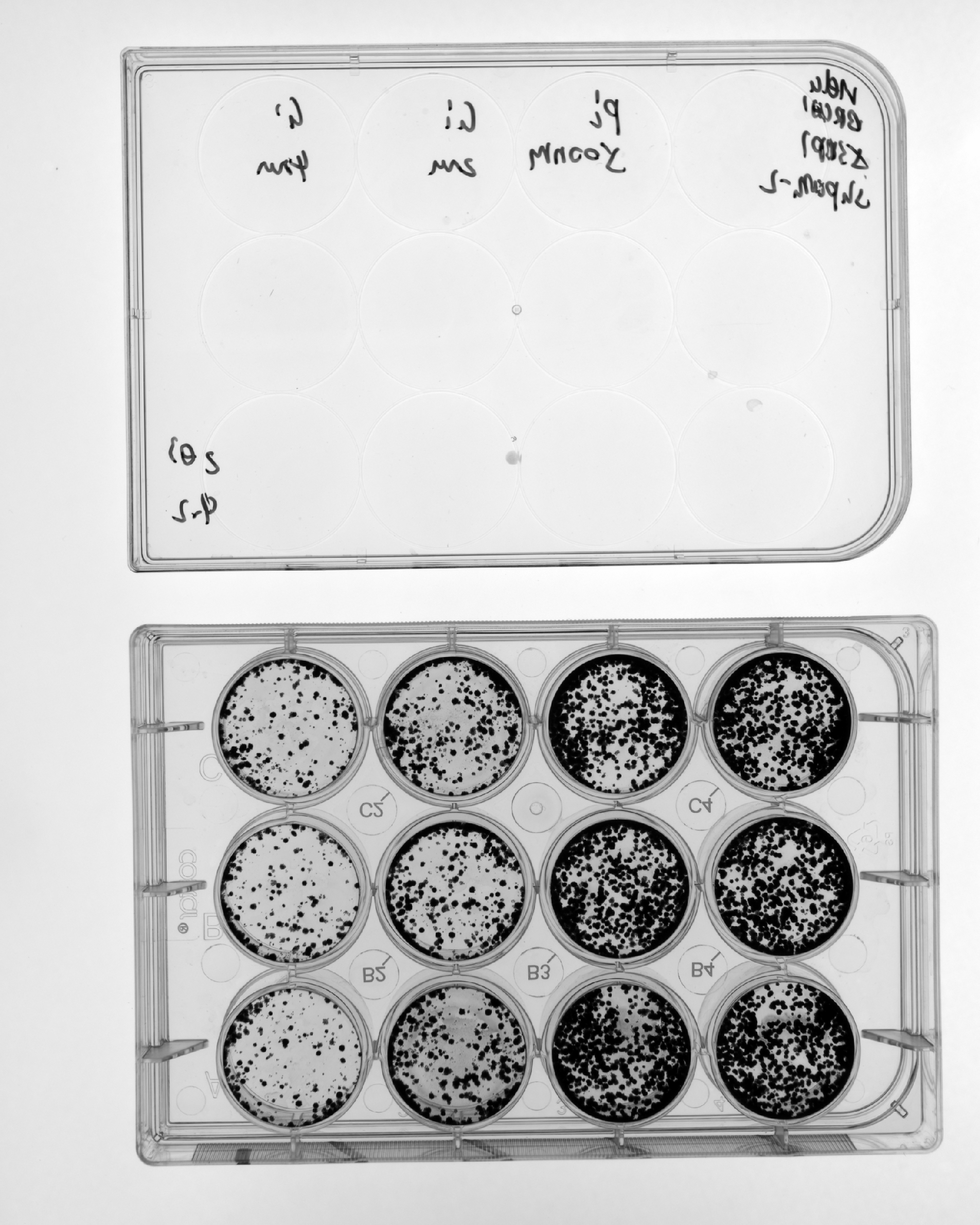

Supplement: Figure 6—figure supplement 2—source data 1. [file elife-89303-fig6-figsupp2-data1.zip › Figure 6-Figure Supplement 2-Source data 1/S7B/litong nie 2022-09-12 11h14m28s(Coomassie Blue).tif]

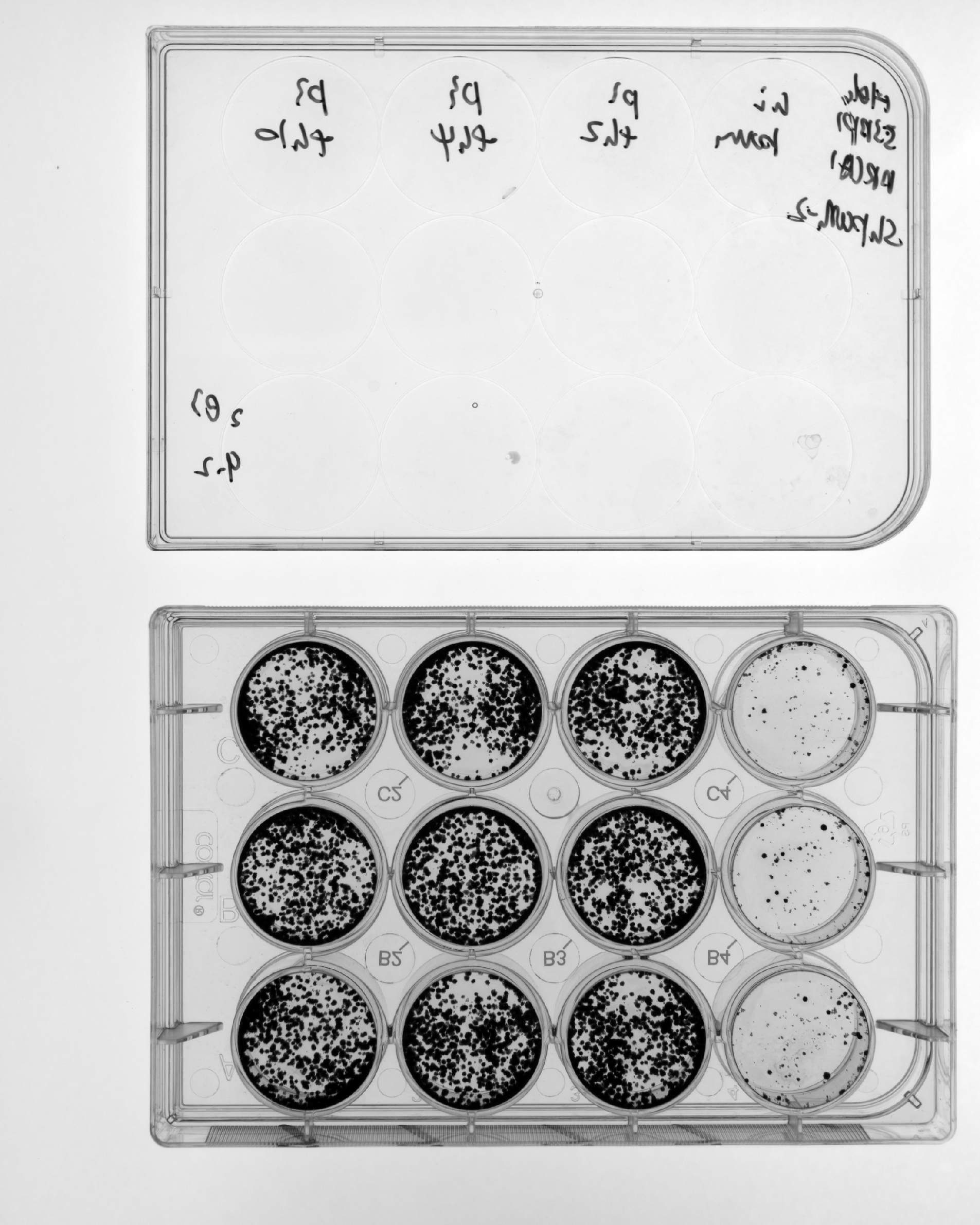

Supplement: Figure 6—figure supplement 2—source data 1. [file elife-89303-fig6-figsupp2-data1.zip › Figure 6-Figure Supplement 2-Source data 1/S7B/litong nie 2022-09-12 11h15m25s(Coomassie Blue).tif]

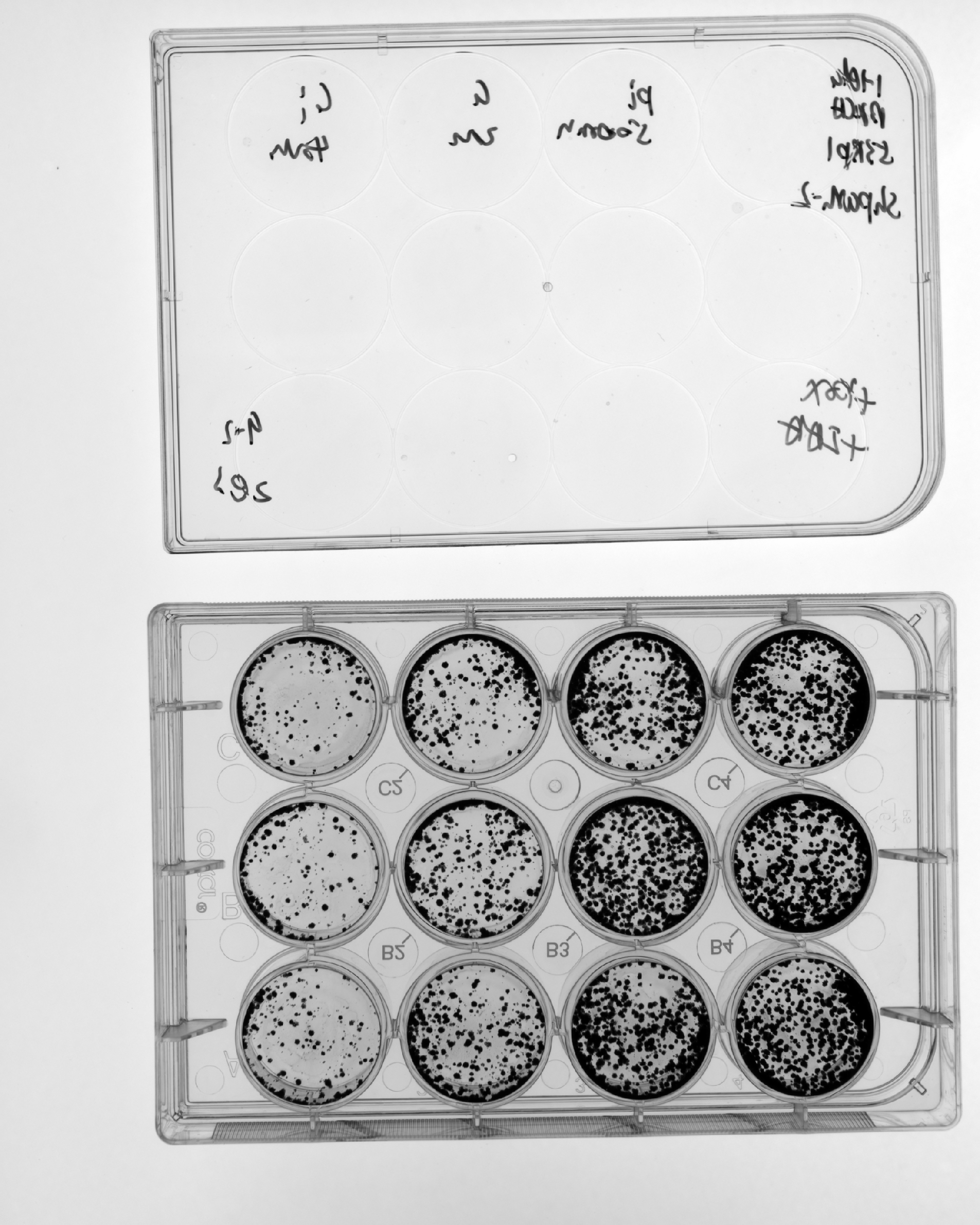

Supplement: Figure 6—figure supplement 2—source data 1. [file elife-89303-fig6-figsupp2-data1.zip › Figure 6-Figure Supplement 2-Source data 1/S7B/litong nie 2022-09-12 11h16m24s(Coomassie Blue).tif]

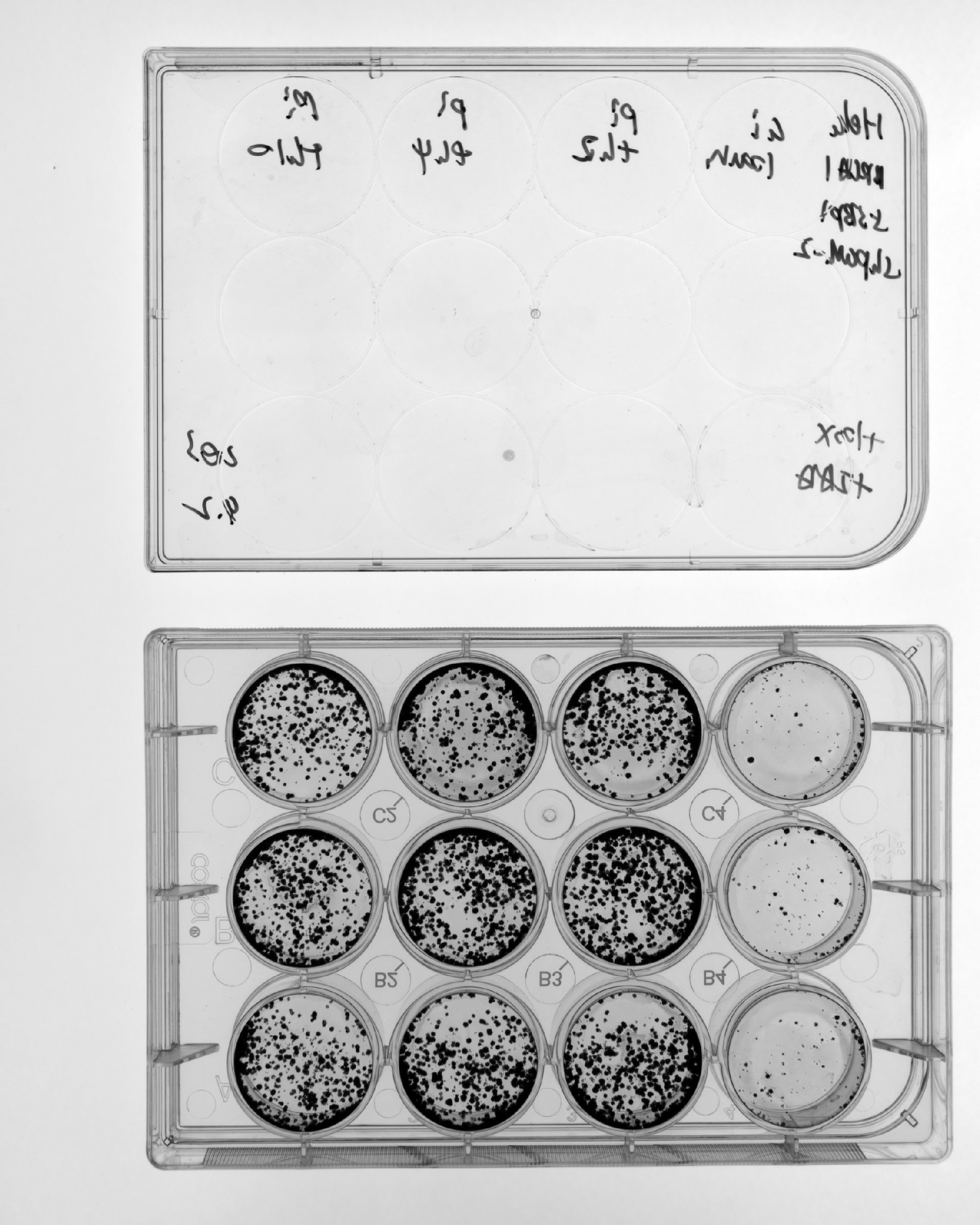

Supplement: Figure 6—figure supplement 2—source data 1. [file elife-89303-fig6-figsupp2-data1.zip › Figure 6-Figure Supplement 2-Source data 1/S7B/litong nie 2022-09-12 11h17m22s(Coomassie Blue).tif]

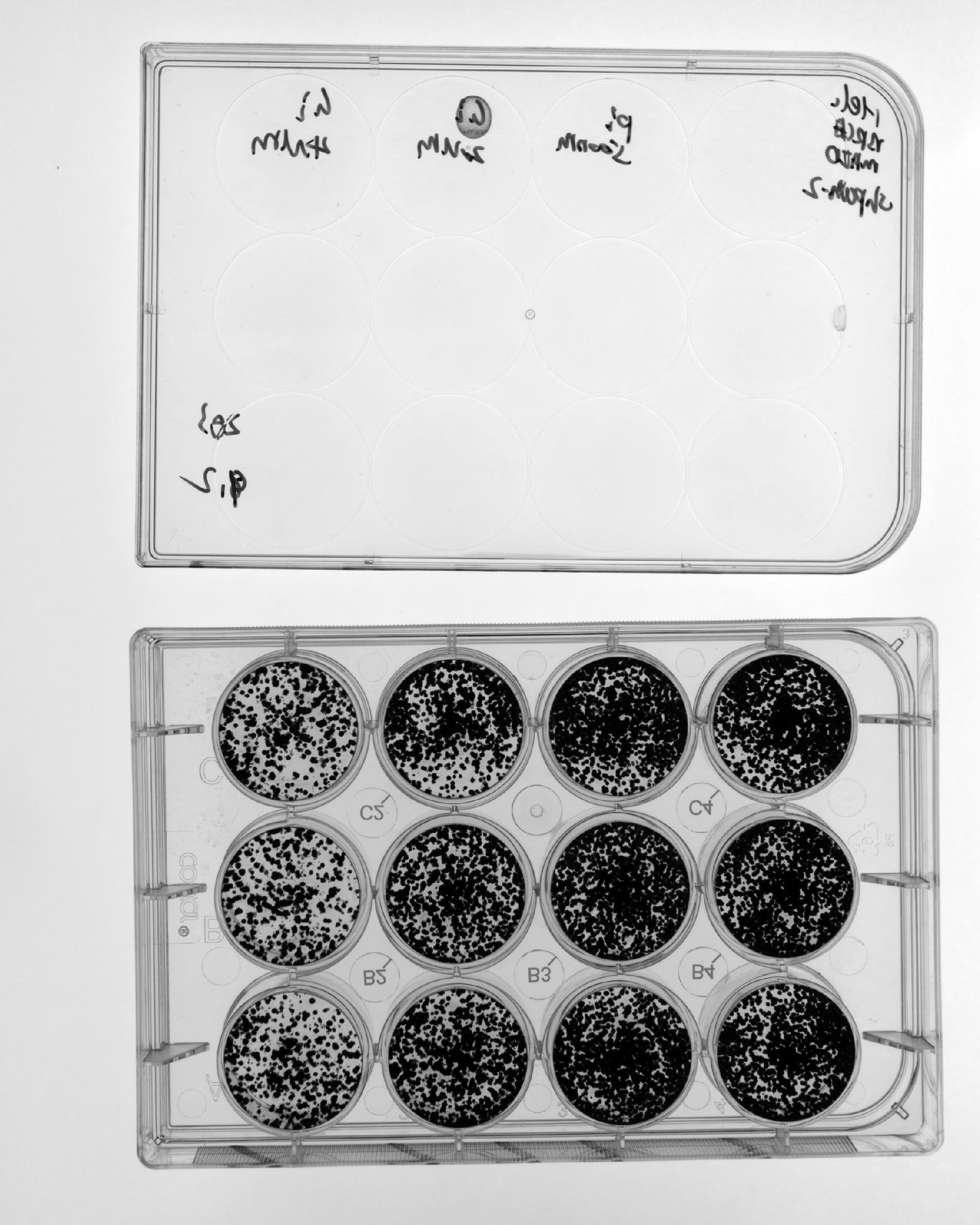

Supplement: Figure 6—figure supplement 2—source data 1. [file elife-89303-fig6-figsupp2-data1.zip › Figure 6-Figure Supplement 2-Source data 1/S7B/litong nie 2022-09-12 11h18m41s(Coomassie Blue).tif]

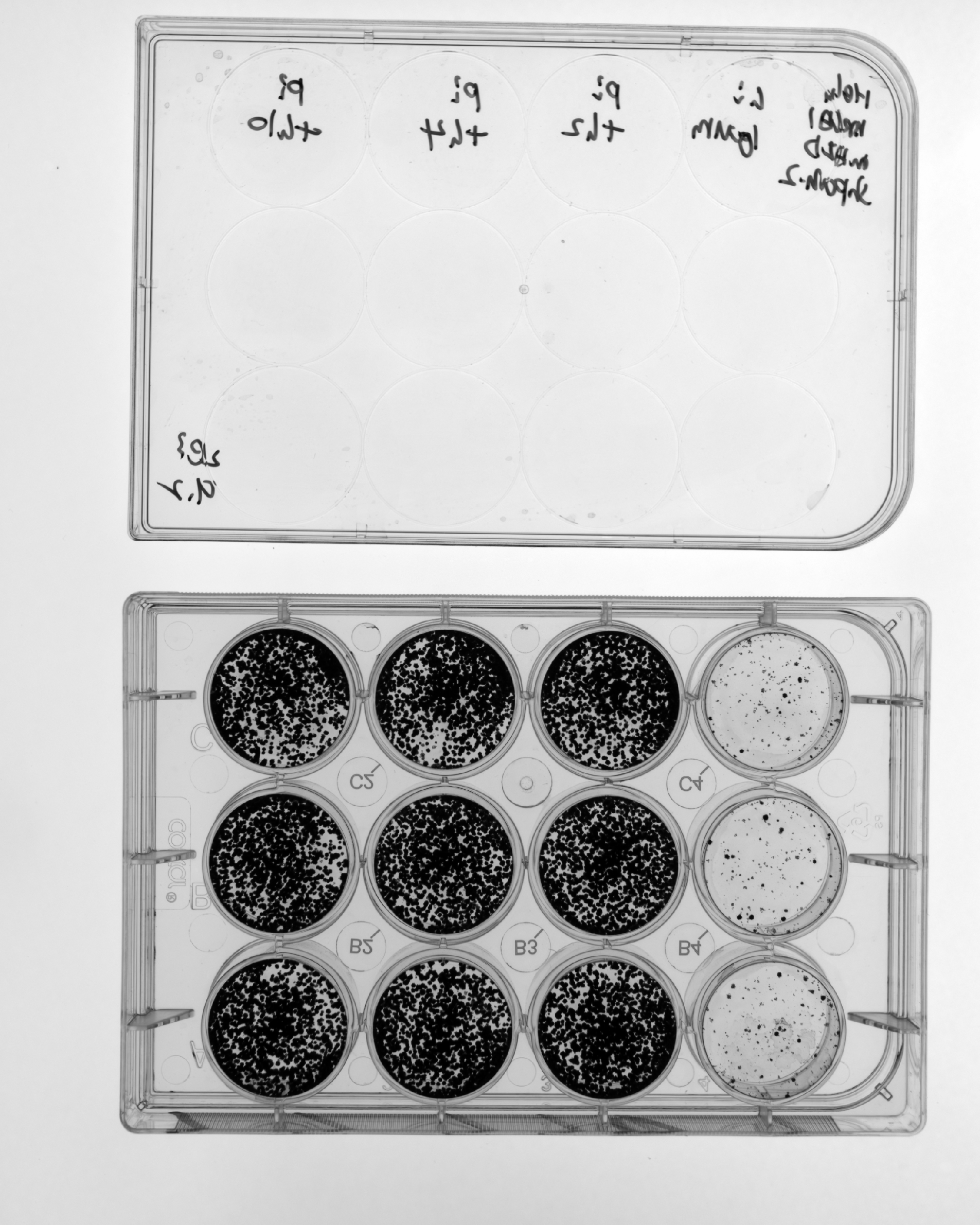

Supplement: Figure 6—figure supplement 2—source data 1. [file elife-89303-fig6-figsupp2-data1.zip › Figure 6-Figure Supplement 2-Source data 1/S7B/litong nie 2022-09-12 11h19m45s(Coomassie Blue).tif]
