# Supplementary material for: DePARylation is critical for S phase progression and cell survival
Source: eLife. 2024 Apr 5;12:RP89303. doi: 10.7554/eLife.89303 (PMC10997334; doi:10.7554/eLife.89303)
Supplement: Figure 2—figure supplement 1—source data 4. [file elife-89303-fig2-figsupp1-data4.zip › Figure 2-Figure Supplement 1-Source data 4/Figure 2-Figure Supplement 1-Source data 4.pdf]

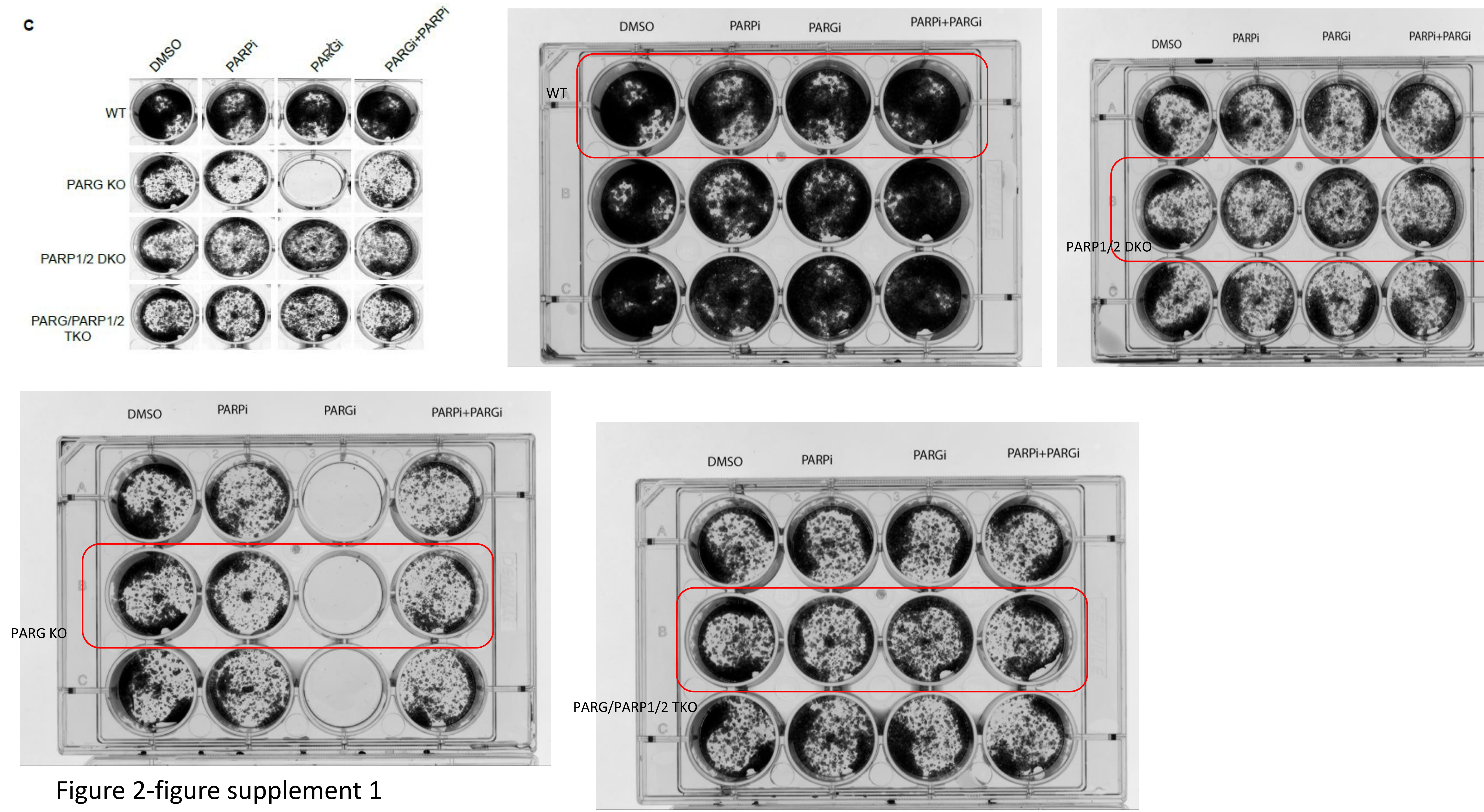

Figure 2-figure supplement 1

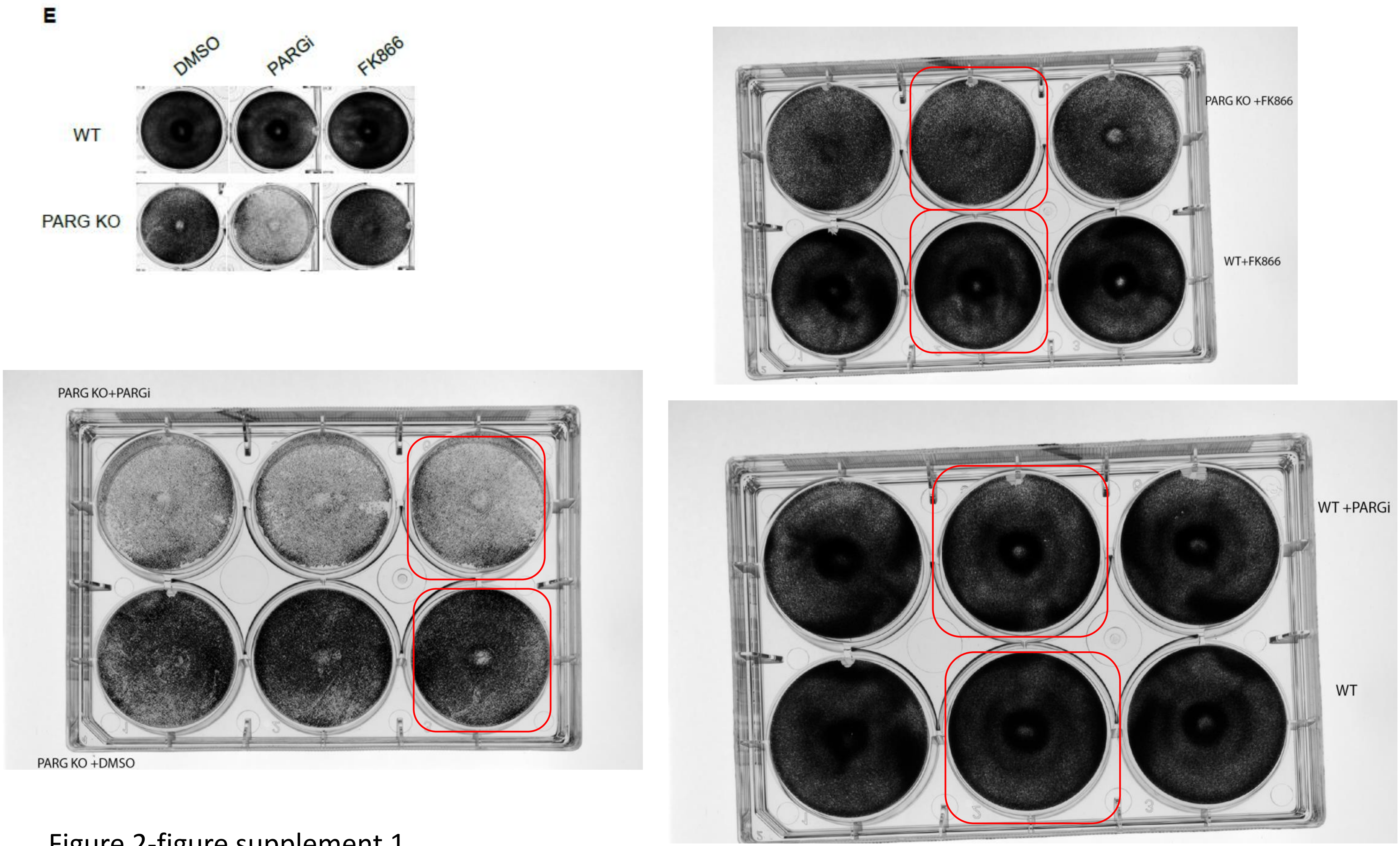

Figure 2-figure supplement 1

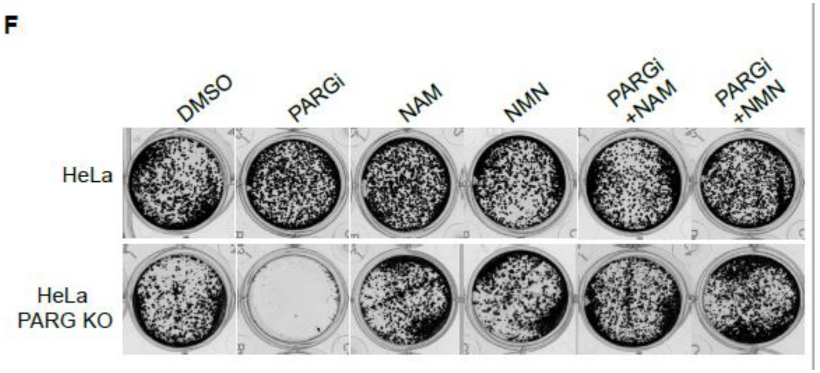

HeLa PARG KO

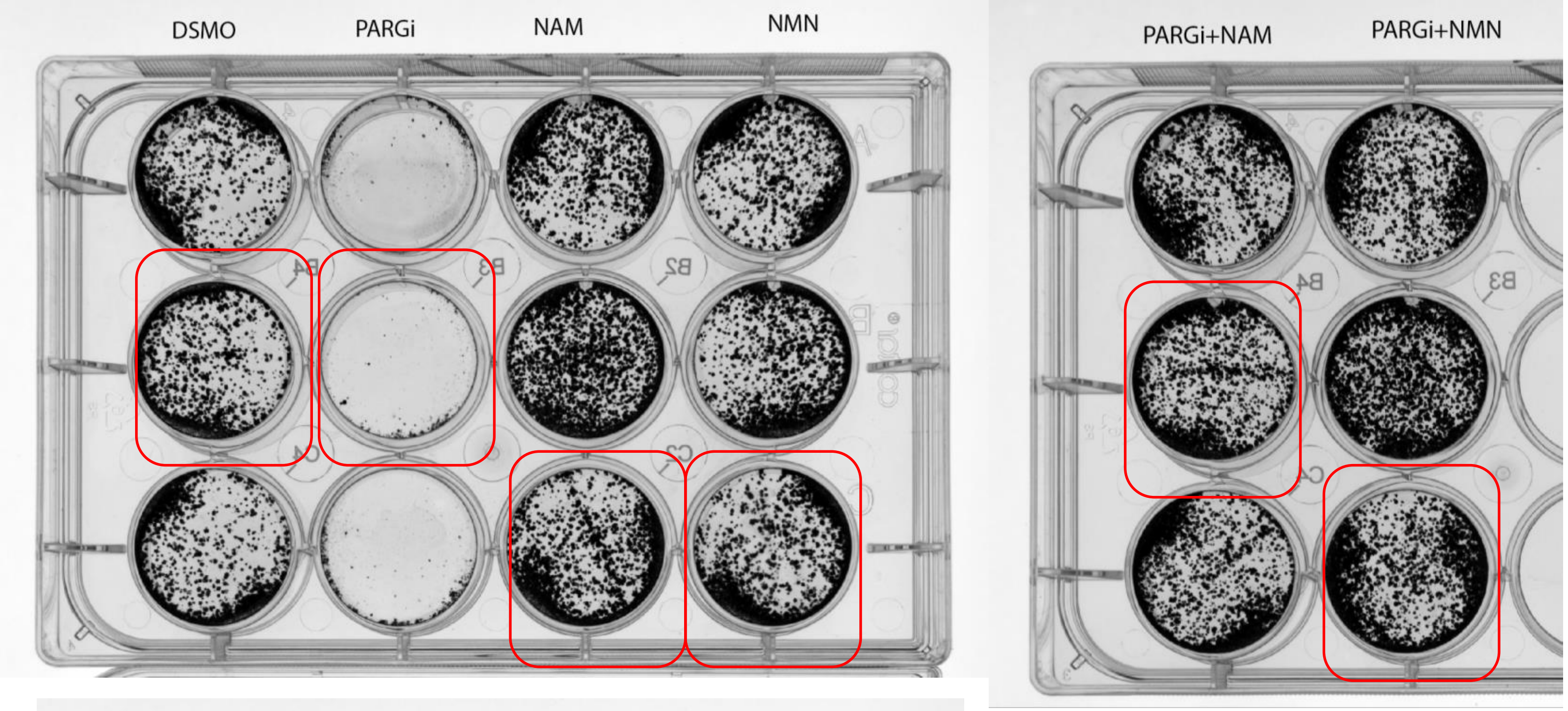

HeLa

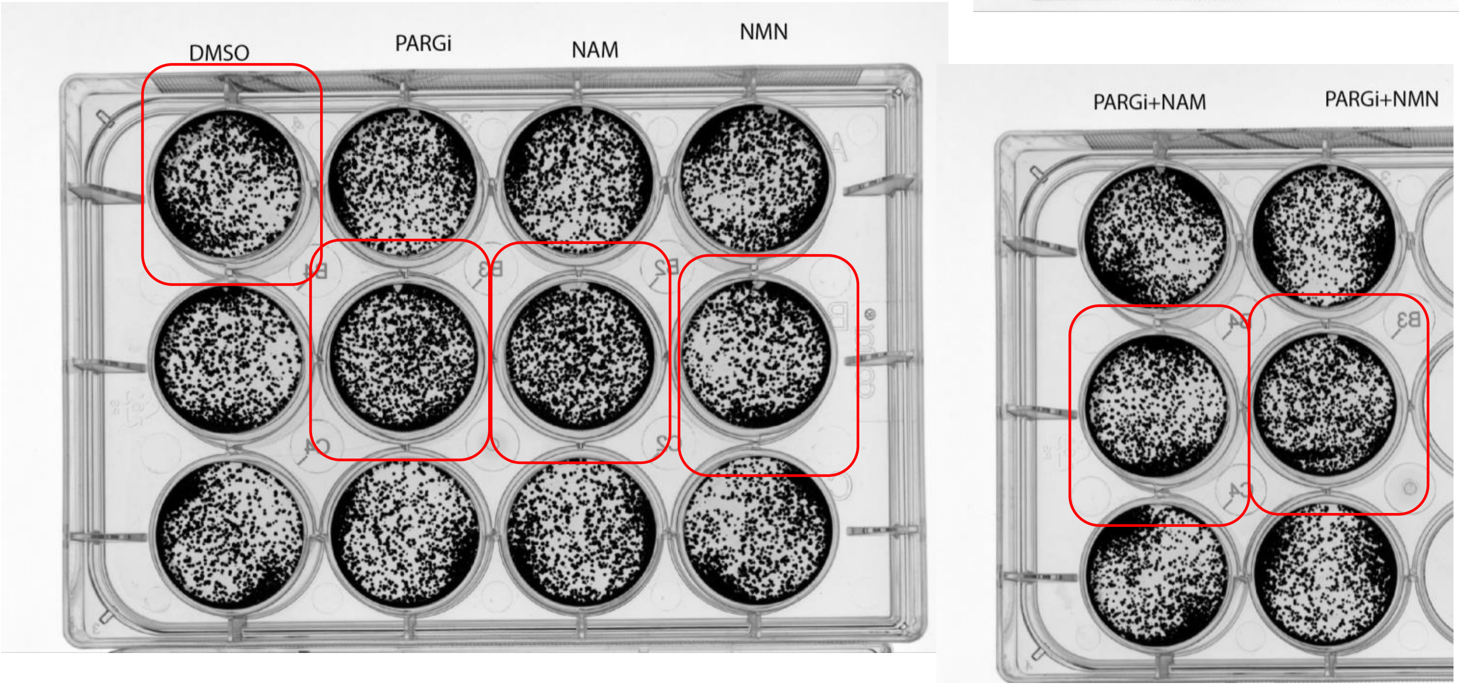

Figure 2-figure supplement 1
