# Supplementary material for: DePARylation is critical for S phase progression and cell survival
Source: eLife. 2024 Apr 5;12:RP89303. doi: 10.7554/eLife.89303 (PMC10997334; doi:10.7554/eLife.89303)
Supplement: Figure 4—figure supplement 1—source data 2. [file elife-89303-fig4-figsupp1-data2.zip › Figure 4-Figure Supplement 1-Source data 2/Figure 4-Figure Supplement 1-Source data 2.pdf]

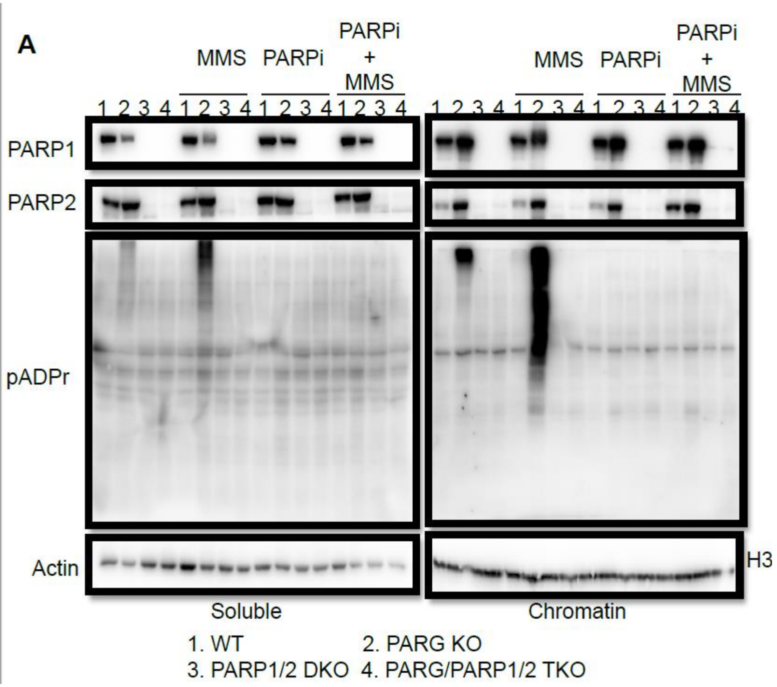

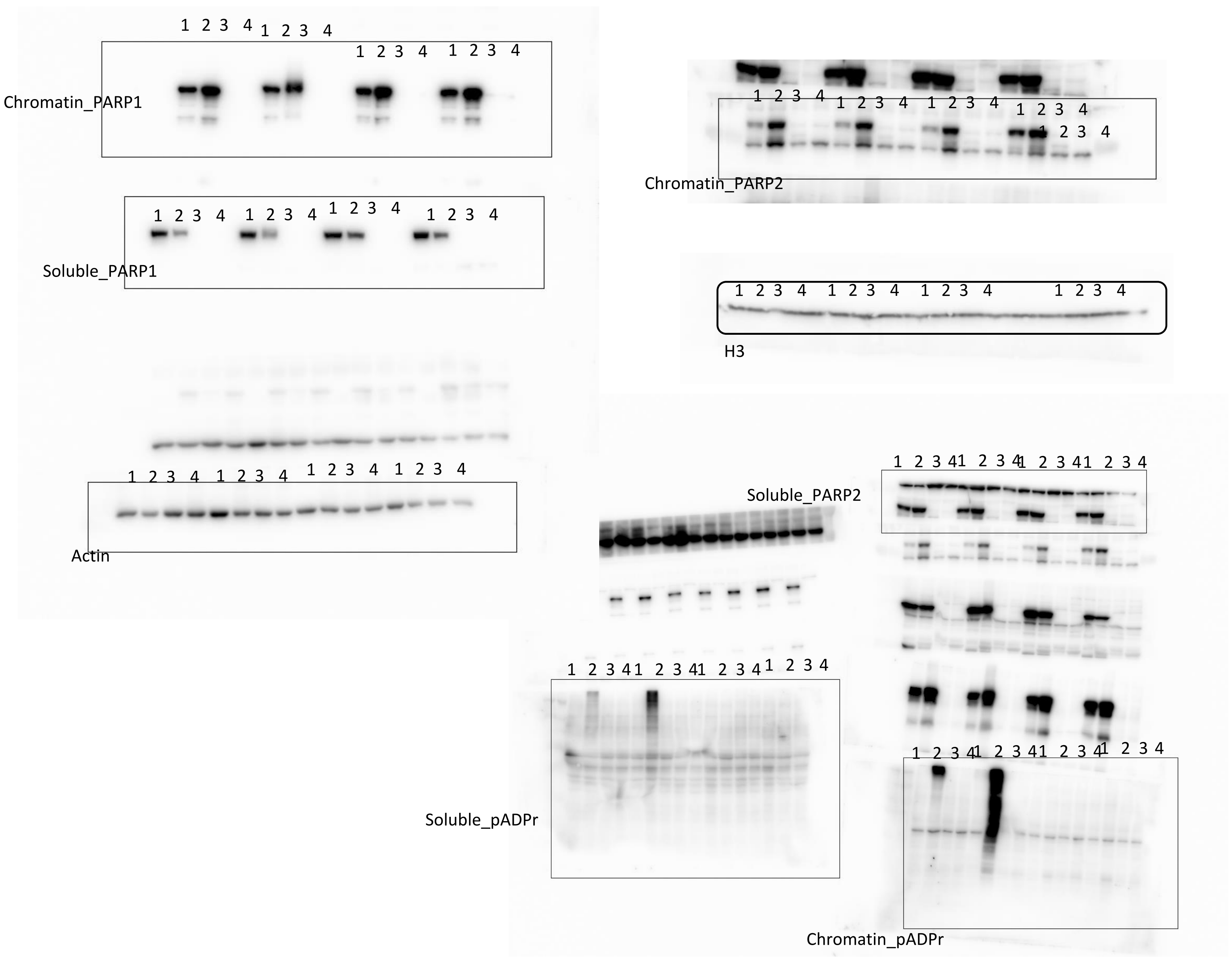

Figure 4-Figure Supplement 1

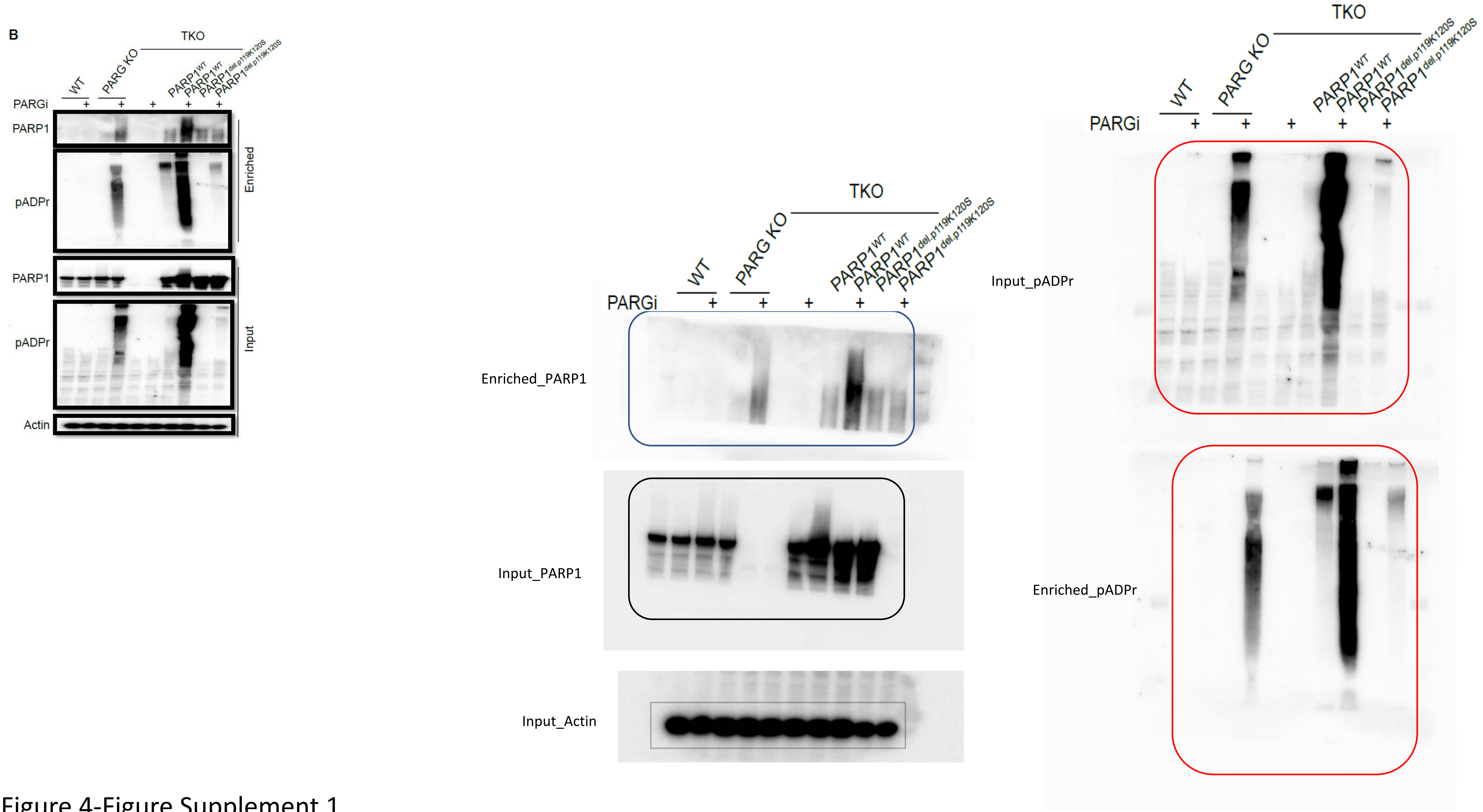

Figure 4-Figure Supplement 1

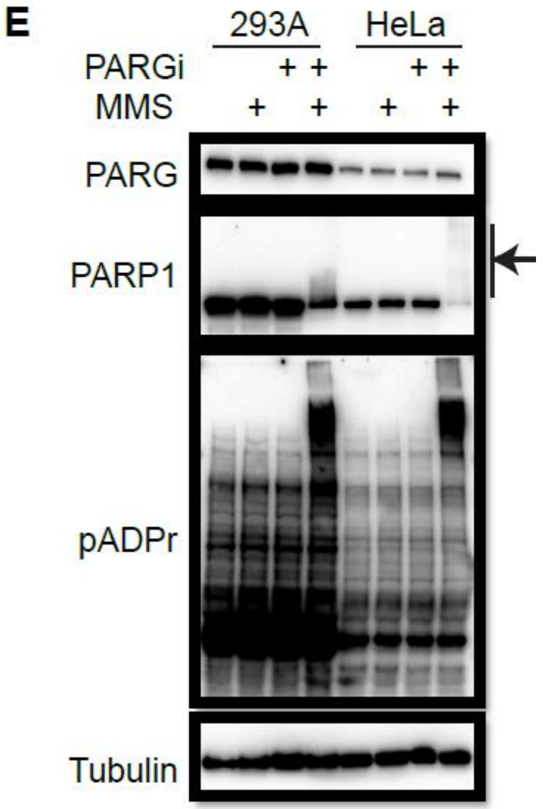

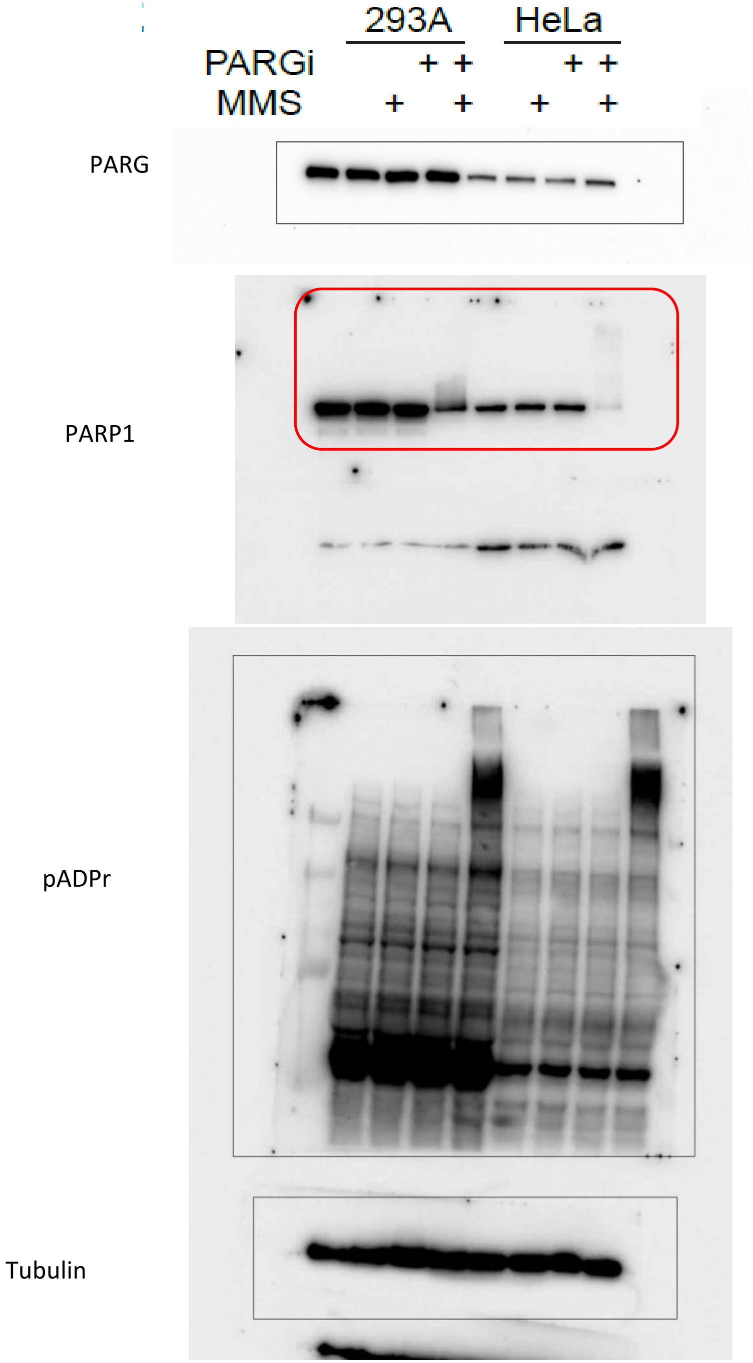

Figure 4-Figure Supplement 1

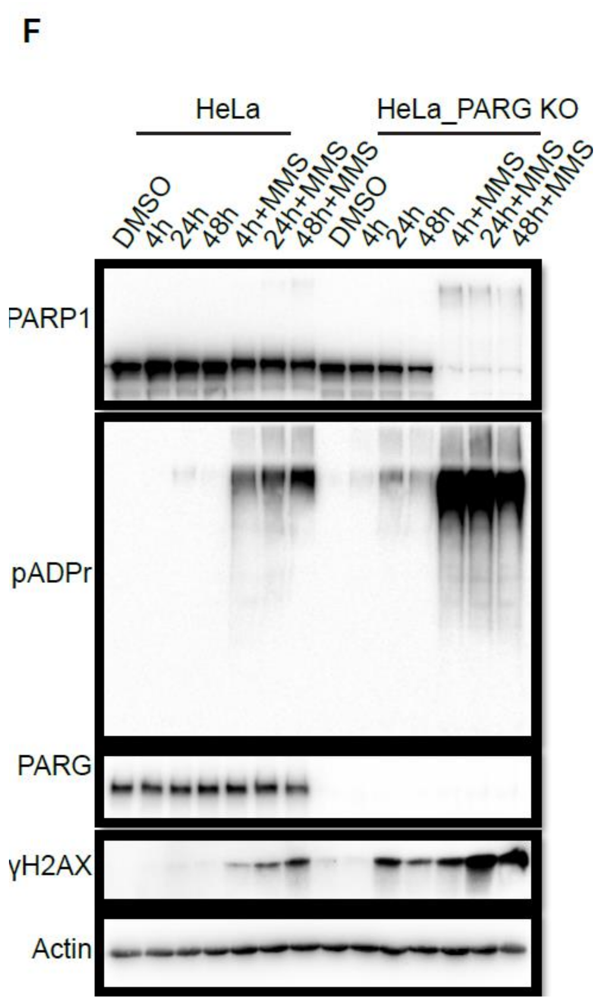

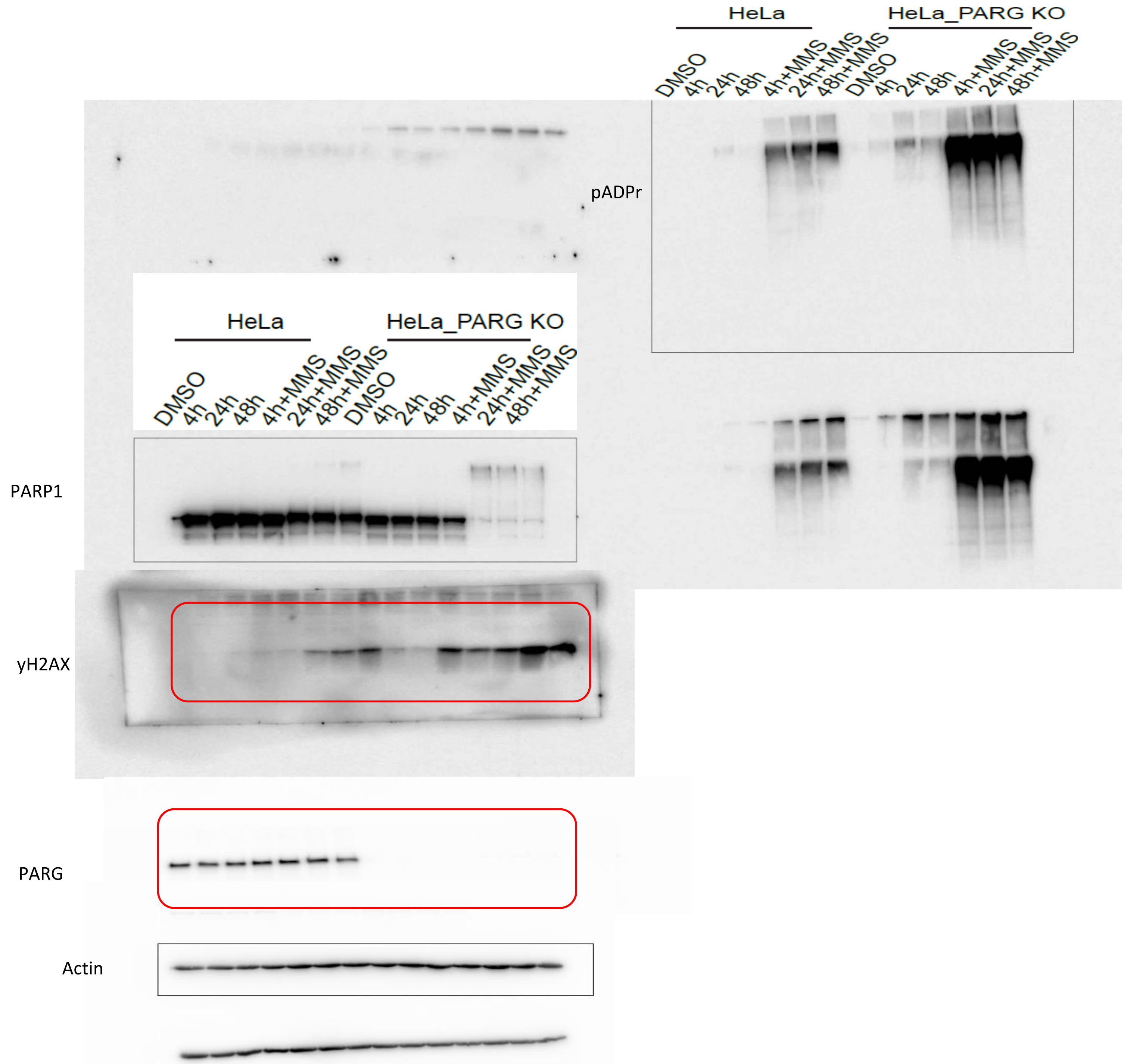

Figure 4-Figure Supplement 1
