# Supplementary material for: DePARylation is critical for S phase progression and cell survival
Source: eLife. 2024 Apr 5;12:RP89303. doi: 10.7554/eLife.89303 (PMC10997334; doi:10.7554/eLife.89303)
Supplement: Figure 5—figure supplement 1—source data 3. [file elife-89303-fig5-figsupp1-data3.zip › Figure 5-Figure Supplement 1-Source data 3/Figure 5-Figure Supplement 1-Source data 3.pdf]

B

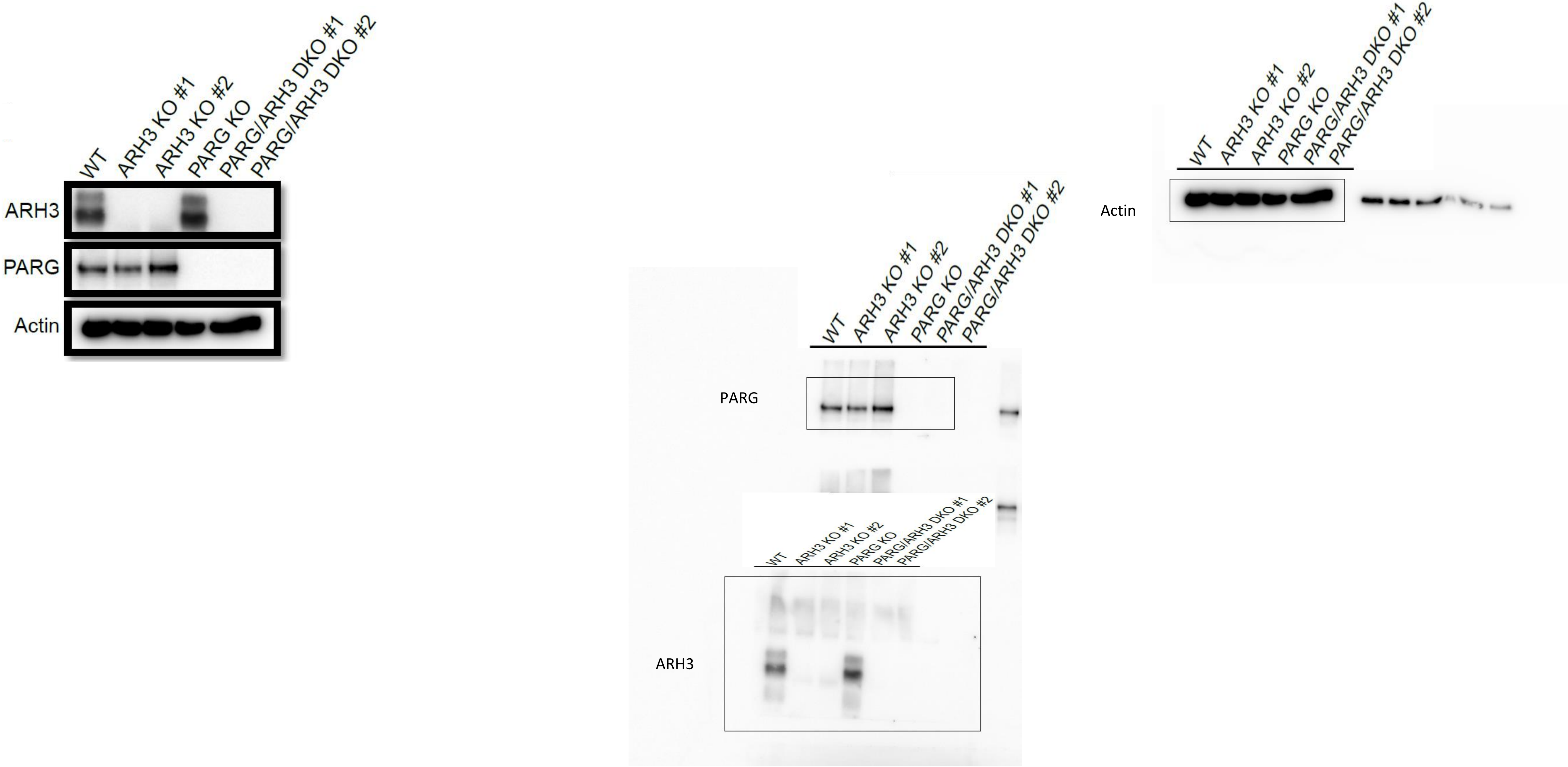

Figure 5-Figure Supplement 1

C

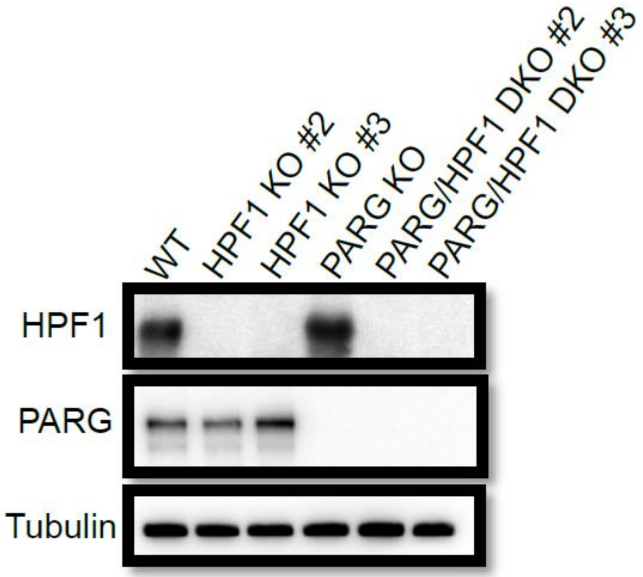

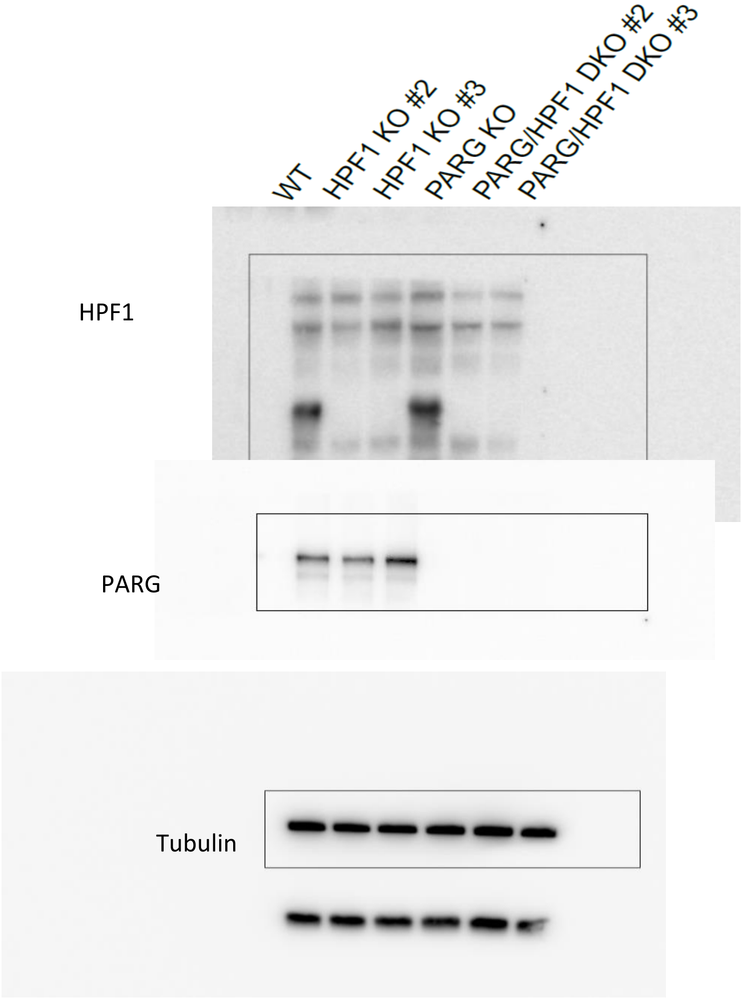

Figure 5-Figure Supplement 1

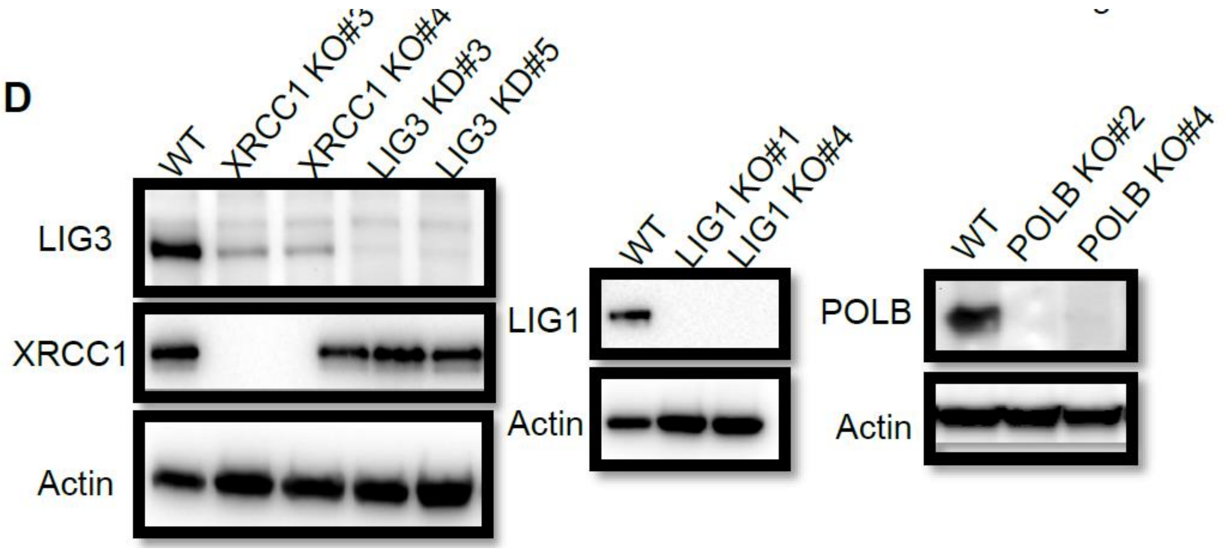

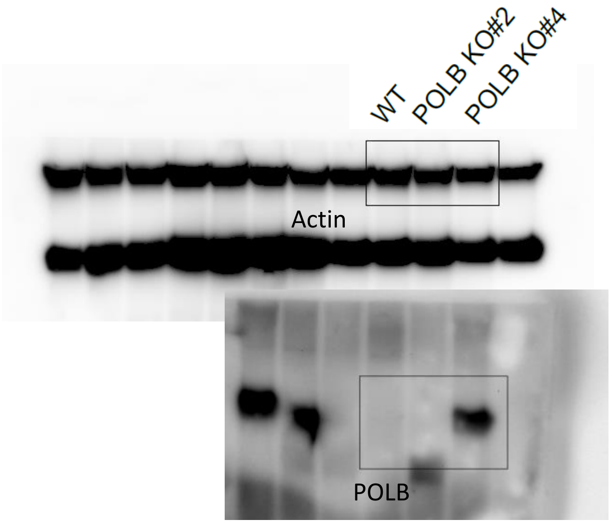

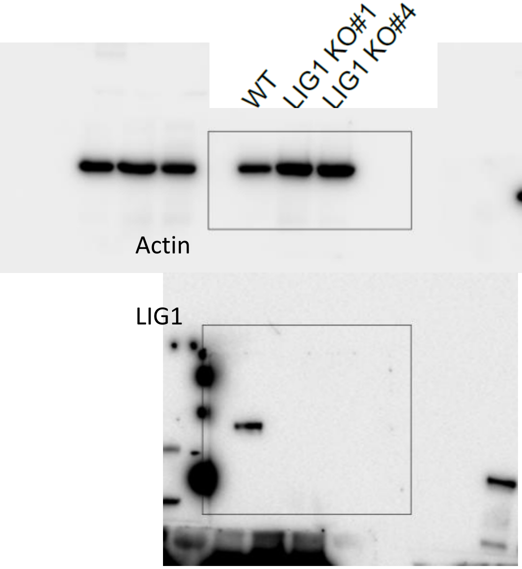

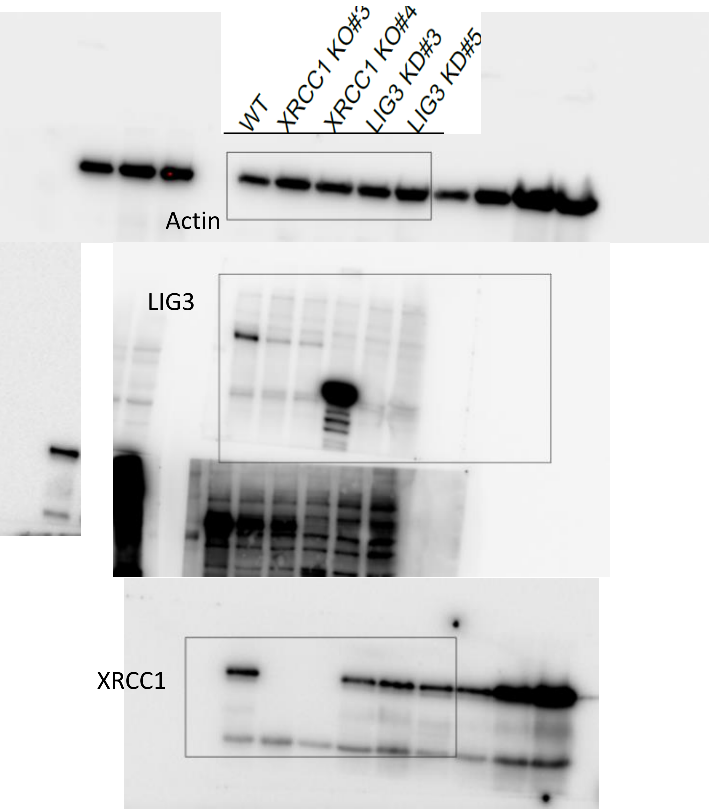

Figure 5-Figure Supplement 1

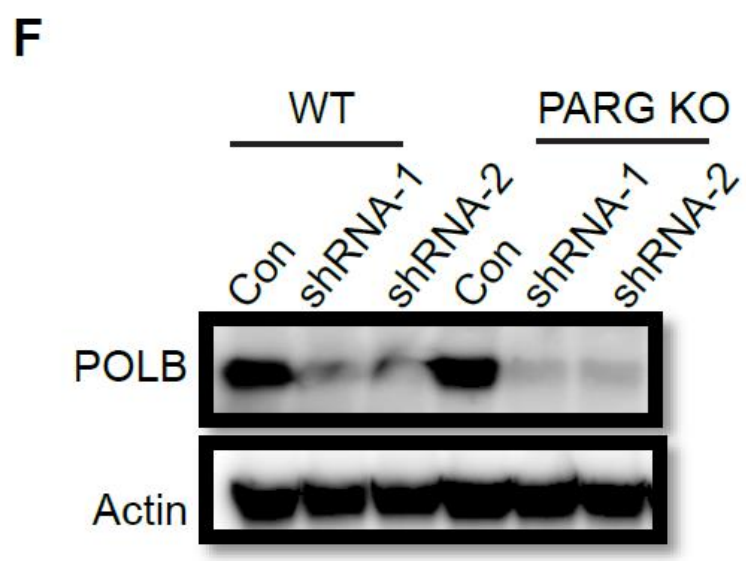

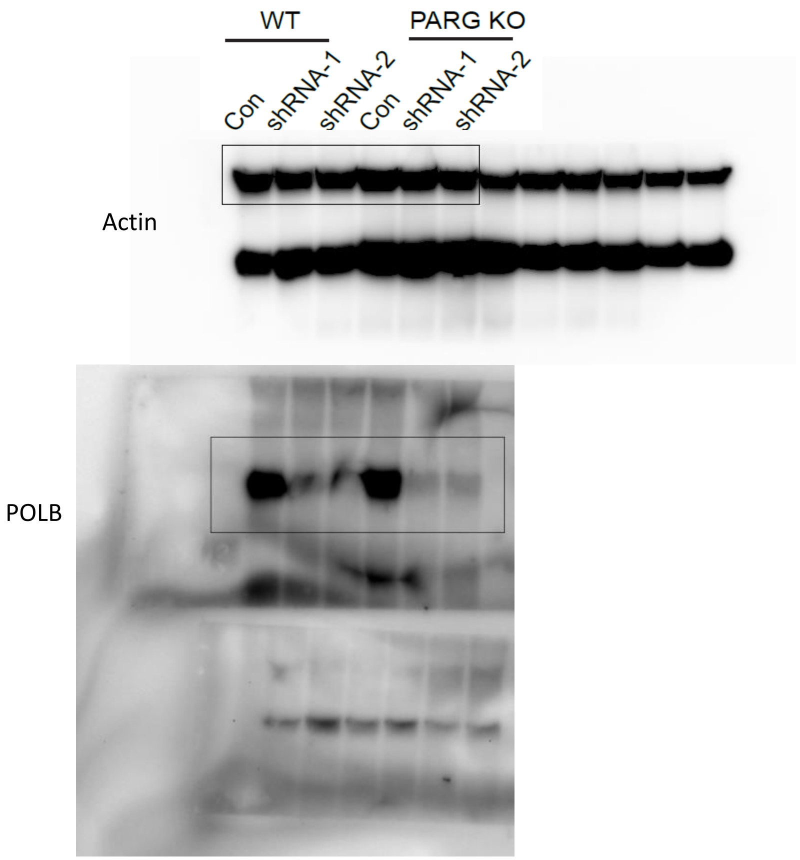

Figure 5-Figure Supplement 1
