# Supplementary material for: DePARylation is critical for S phase progression and cell survival
Source: eLife. 2024 Apr 5;12:RP89303. doi: 10.7554/eLife.89303 (PMC10997334; doi:10.7554/eLife.89303)
Supplement: Figure 6—figure supplement 1—source data 2. [file elife-89303-fig6-figsupp1-data2.zip › Figure 6-Figure Supplement 1-Source data 2/Figure 6-Figure Supplement 1-Source data 2.pdf]

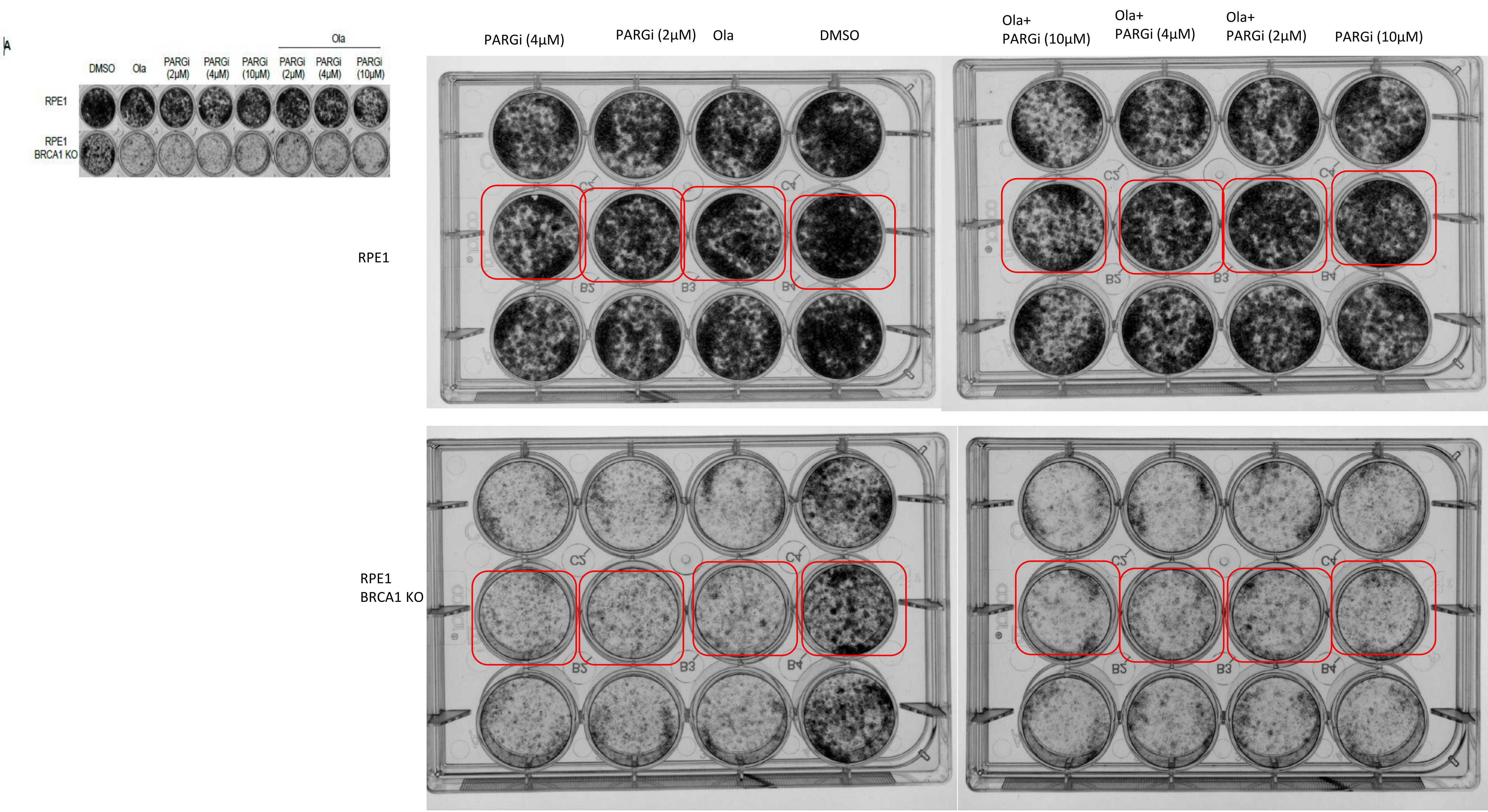

Figure 6-figure supplement 1

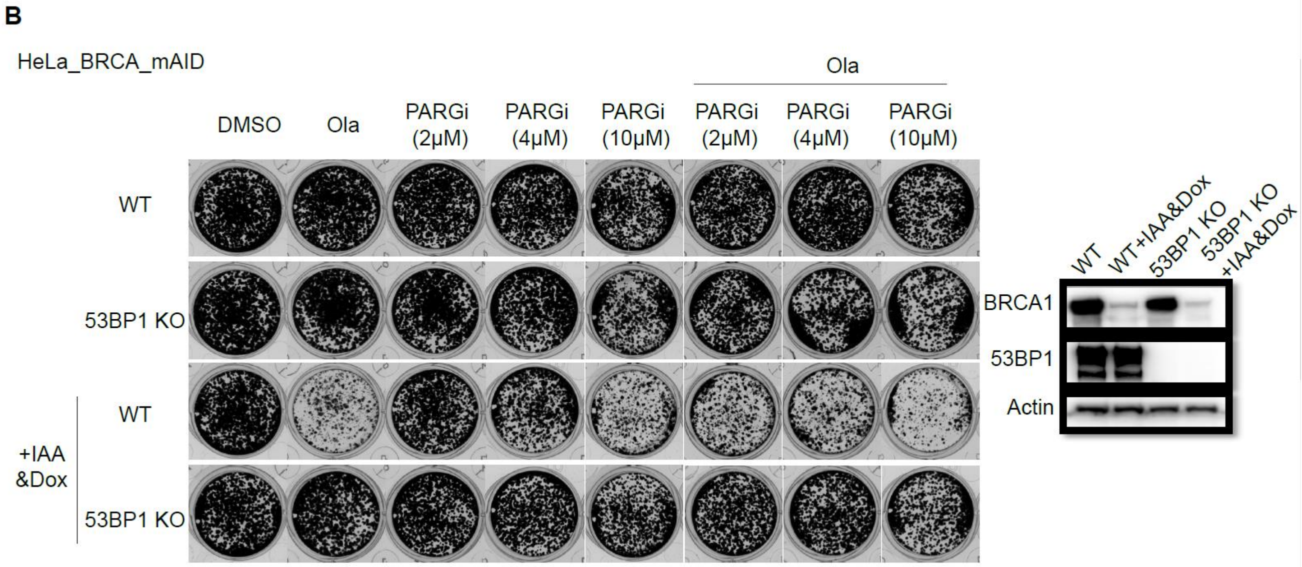

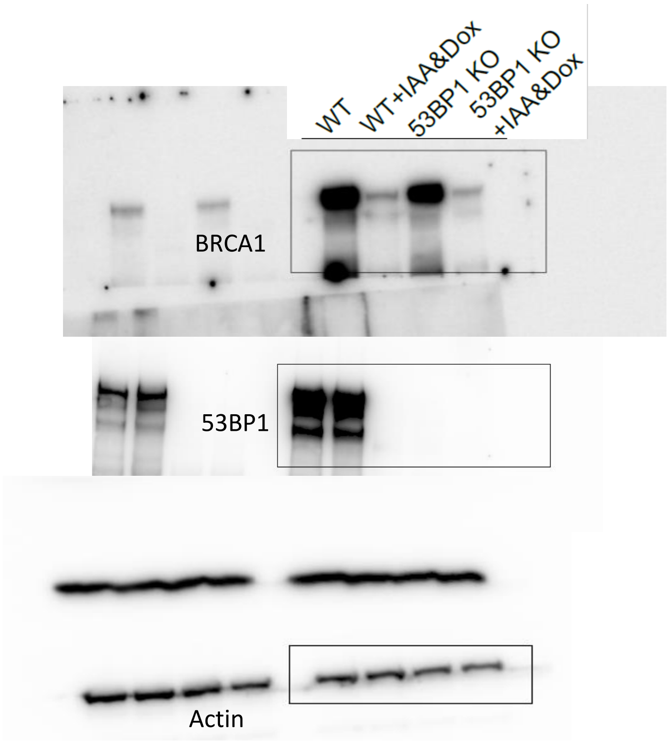

Figure 6-figure supplement 1

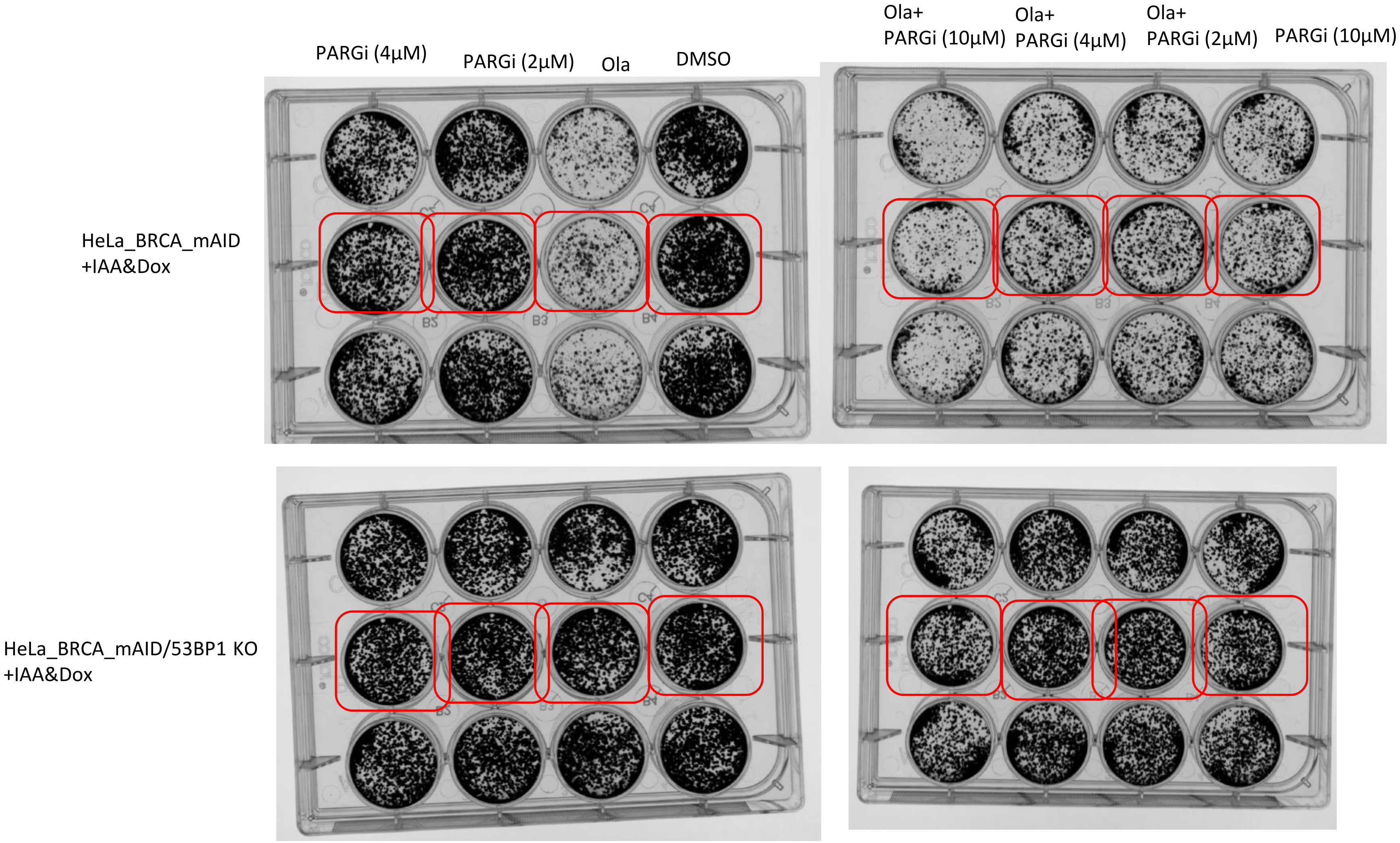

Figure 6-figure supplement 1

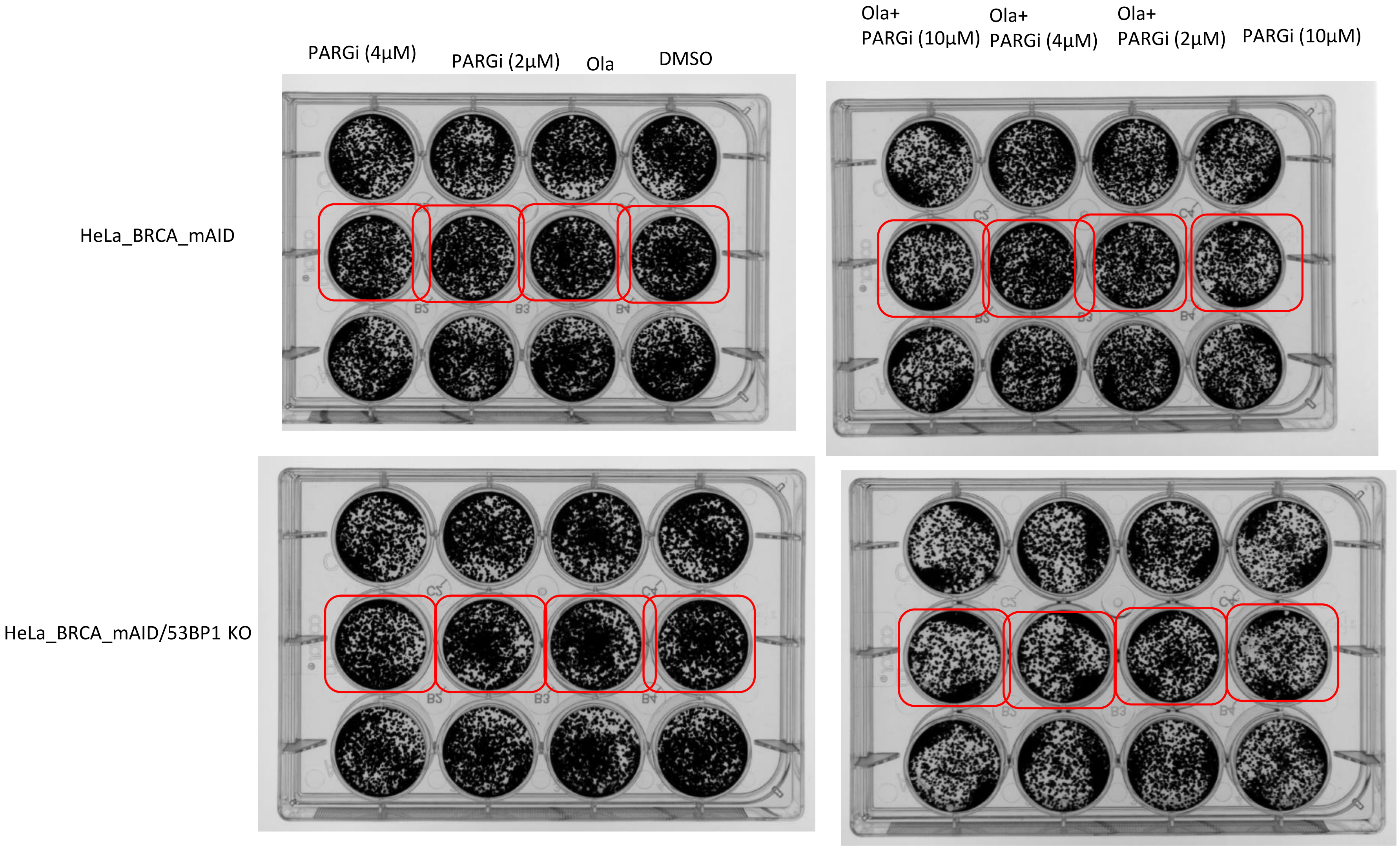
PARGi (4μM)
PARGi (2μM)
Ola
DMSO
Ola+ PARGi (10μM)
Ola+ PARGi (4μM)
Ola+ PARGi (2μM)
PARGi (10μM)
HeLa_BRCA_mAID
HeLa_BRCA_mAID/53BP1 KO
