# Supplementary figures and images for: DePARylation is critical for S phase progression and cell survival (part 2 of 2)
Source: eLife. 2024 Apr 5;12:RP89303. doi: 10.7554/eLife.89303 (PMC10997334; doi:10.7554/eLife.89303)

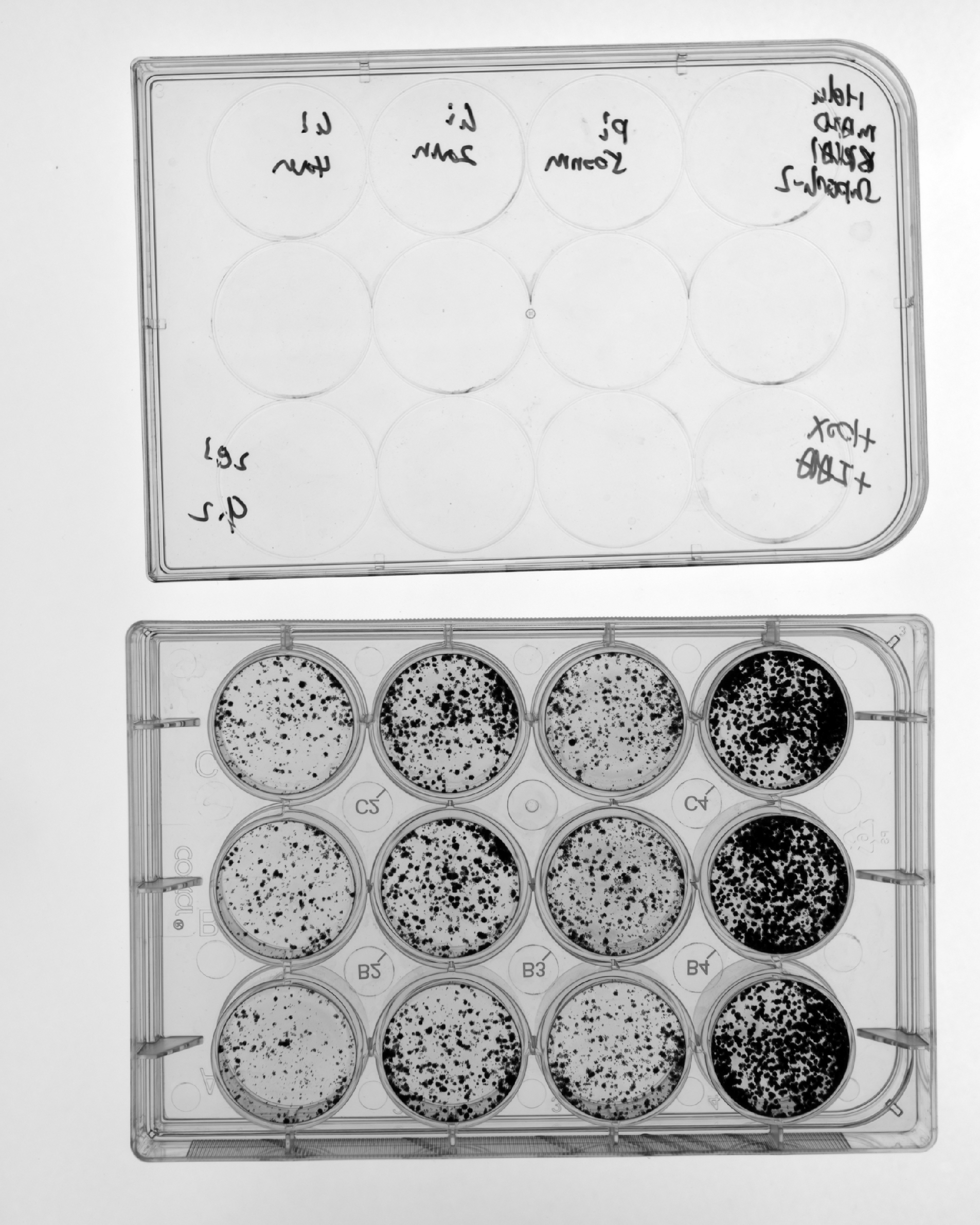

Supplement: Figure 6—figure supplement 2—source data 1. [file elife-89303-fig6-figsupp2-data1.zip › Figure 6-Figure Supplement 2-Source data 1/S7B/litong nie 2022-09-12 11h20m48s(Coomassie Blue).tif]

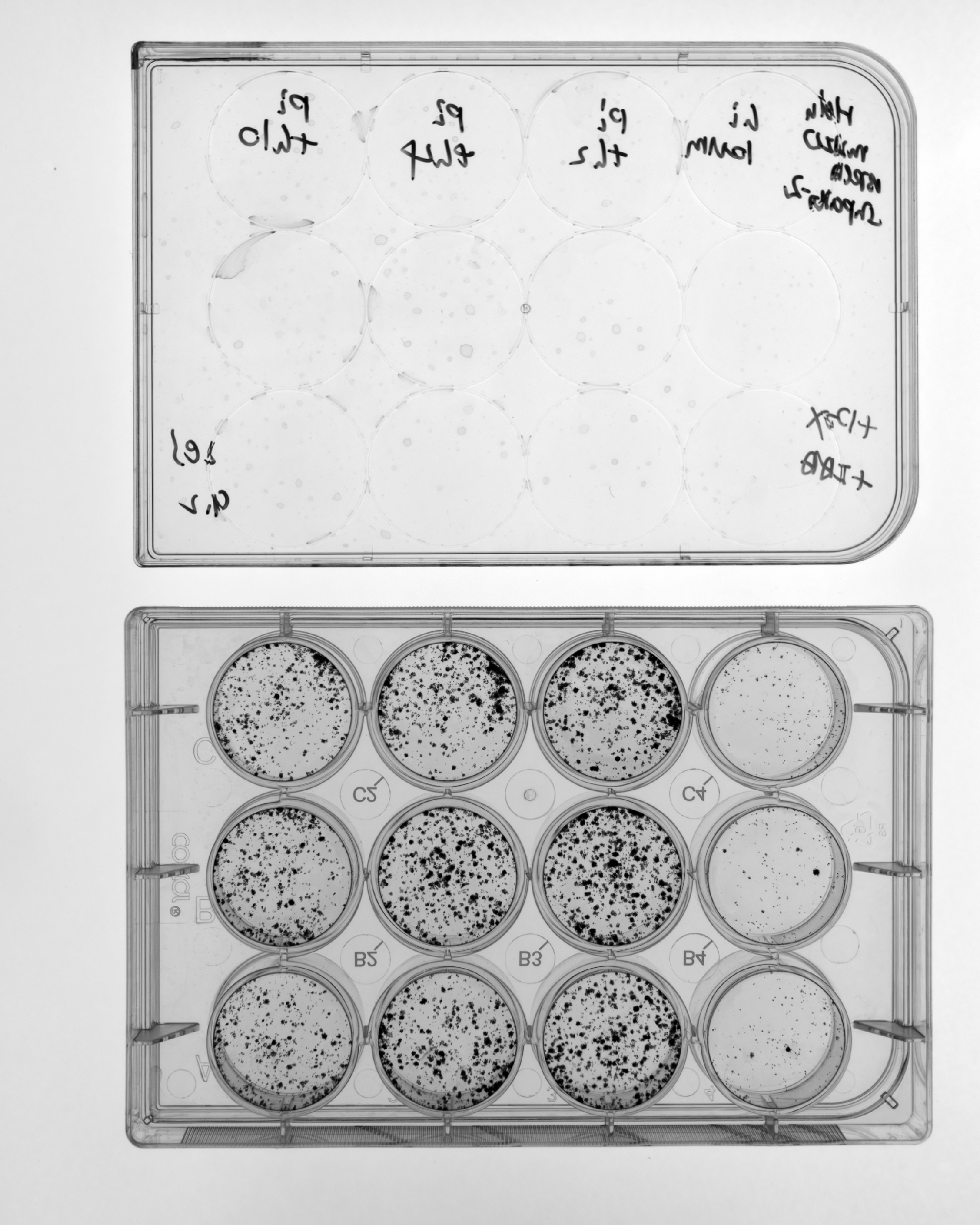

Supplement: Figure 6—figure supplement 2—source data 1. [file elife-89303-fig6-figsupp2-data1.zip › Figure 6-Figure Supplement 2-Source data 1/S7B/litong nie 2022-09-12 11h22m03s(Coomassie Blue).tif]

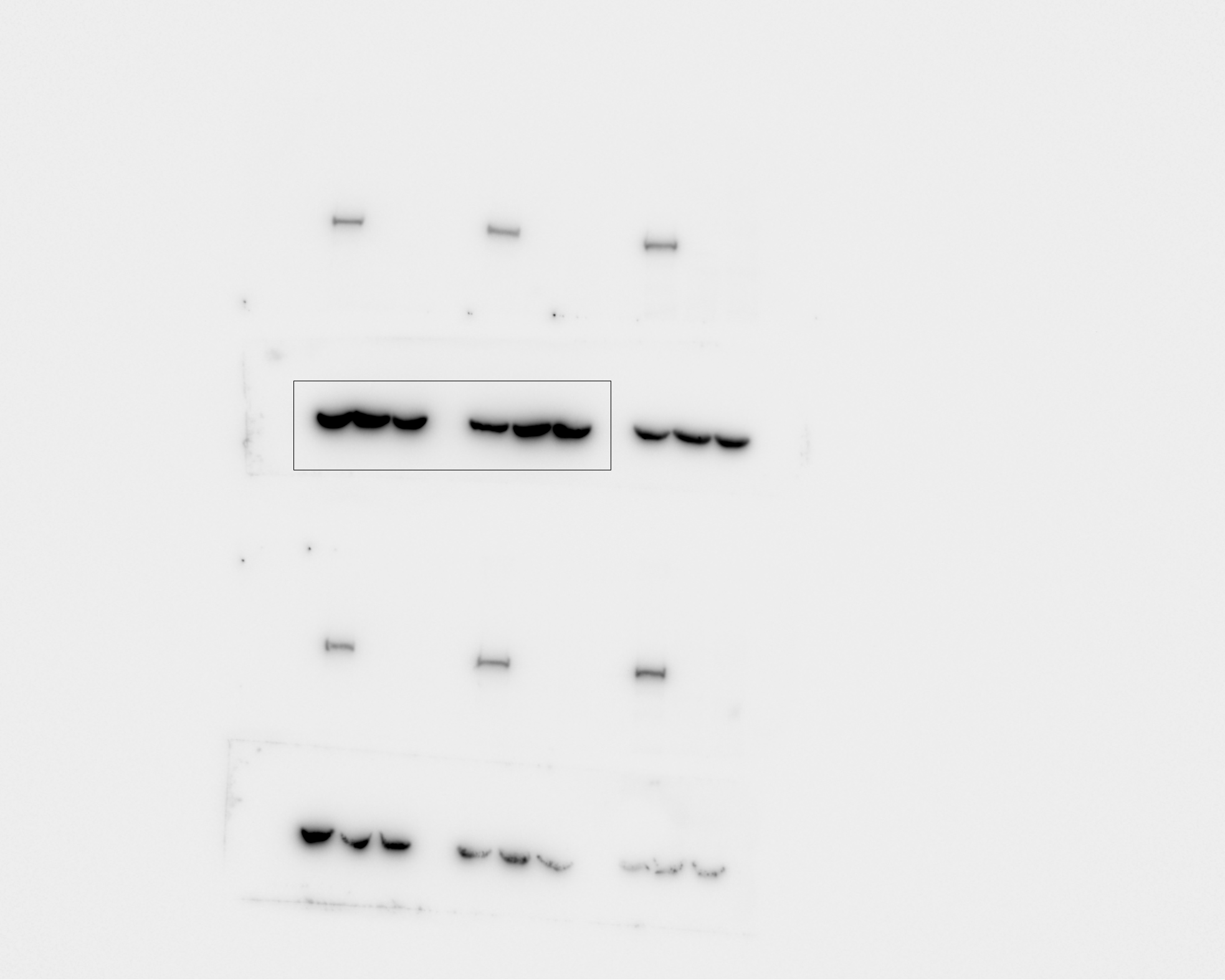

Supplement: Figure 7—source data 1. [file elife-89303-fig7-data1.zip › Figure 7-Source data 1/7B/Actin.tif]

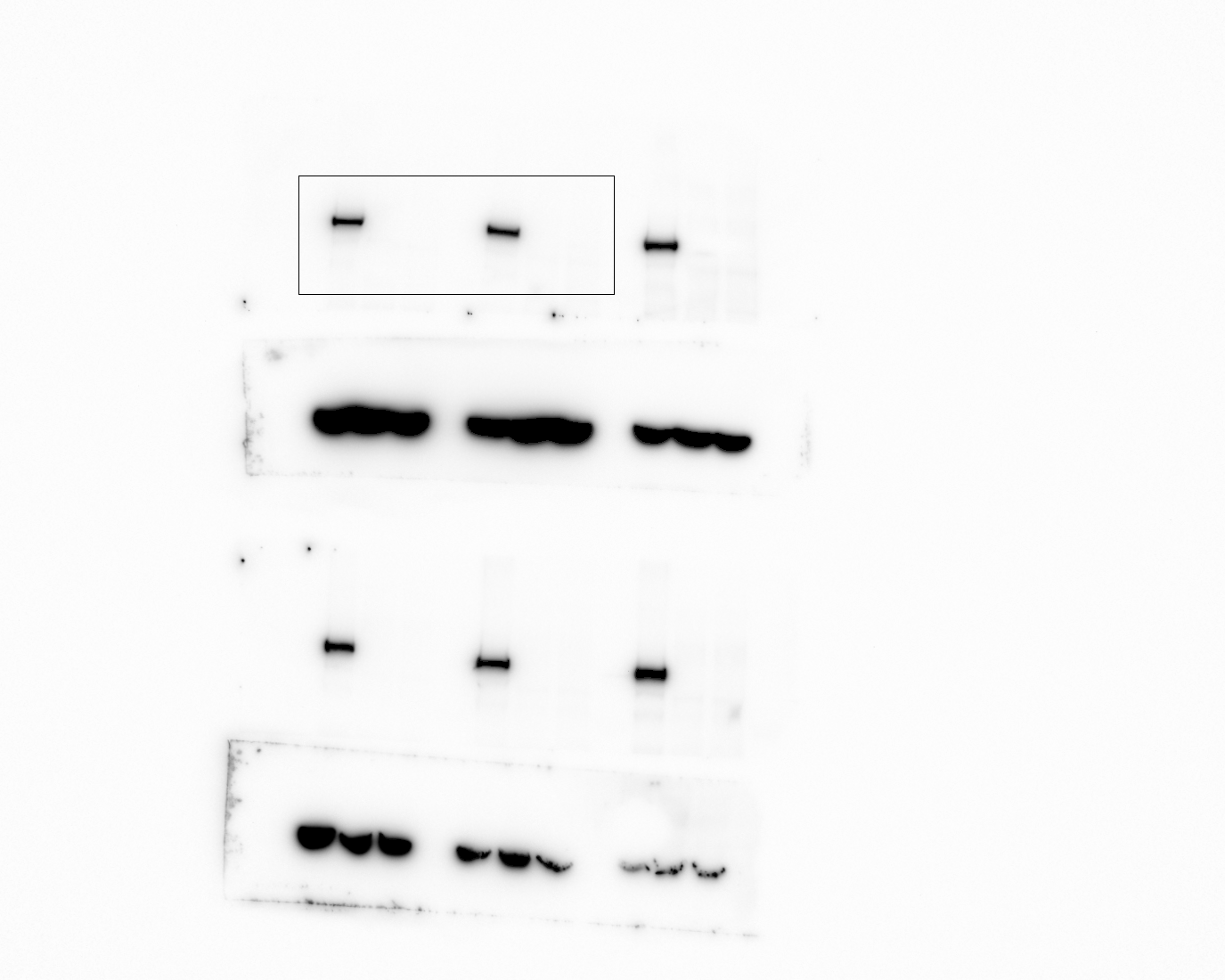

Supplement: Figure 7—source data 1. [file elife-89303-fig7-data1.zip › Figure 7-Source data 1/7B/PARG.tif]

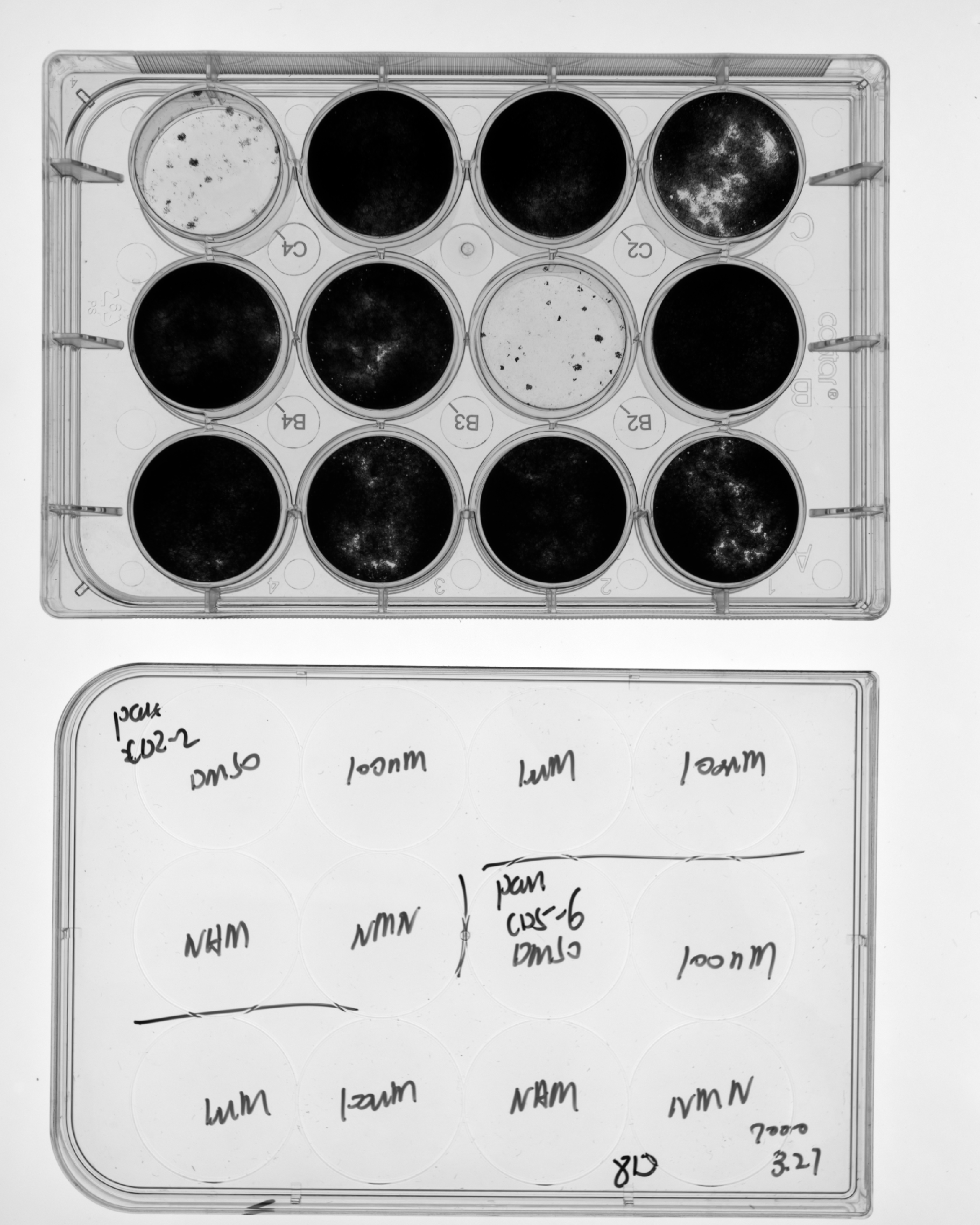

Supplement: Figure 7—source data 1. [file elife-89303-fig7-data1.zip › Figure 7-Source data 1/7C/293A cKO#3_1.tif]

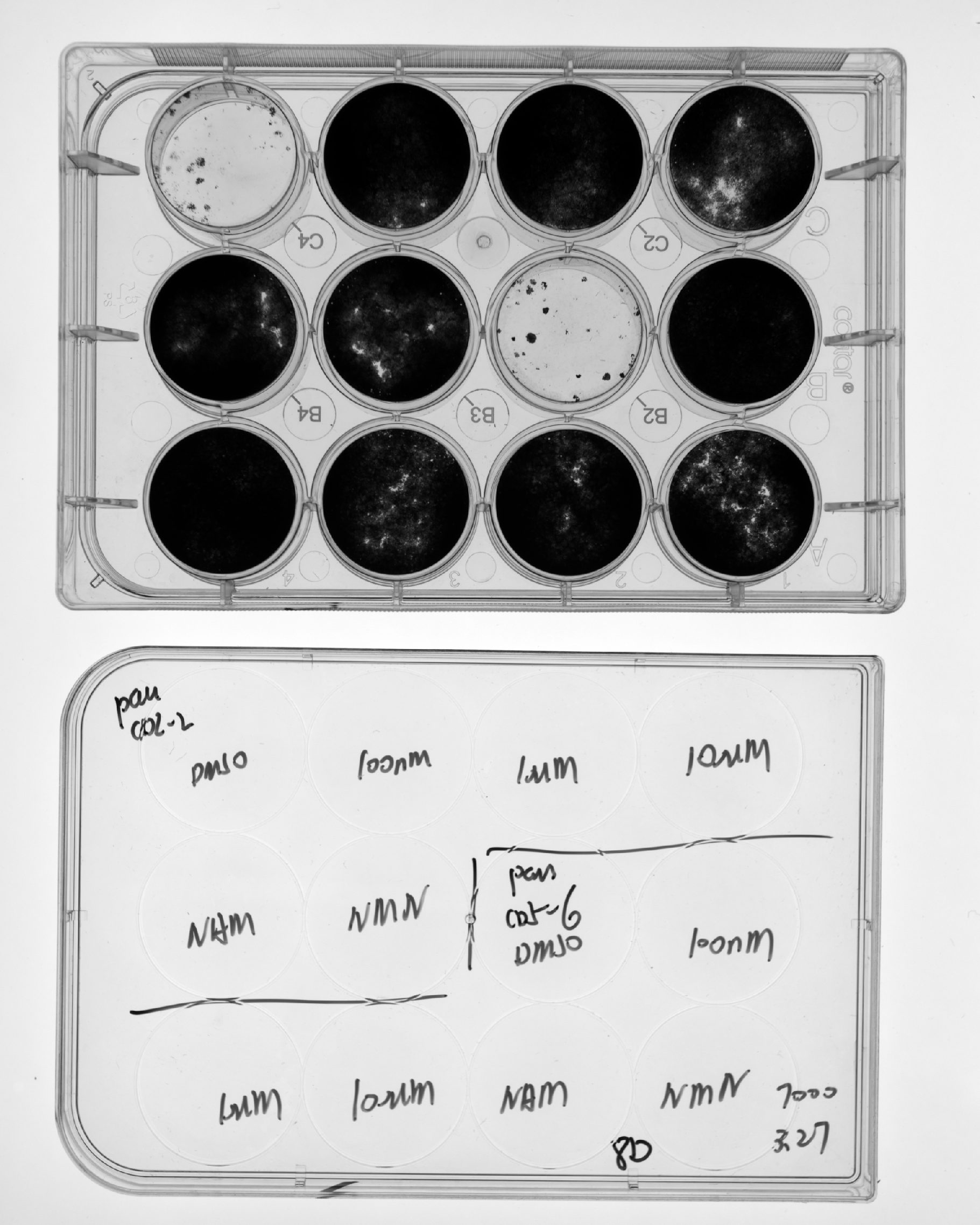

Supplement: Figure 7—source data 1. [file elife-89303-fig7-data1.zip › Figure 7-Source data 1/7C/293A cKO#3_2.tif]

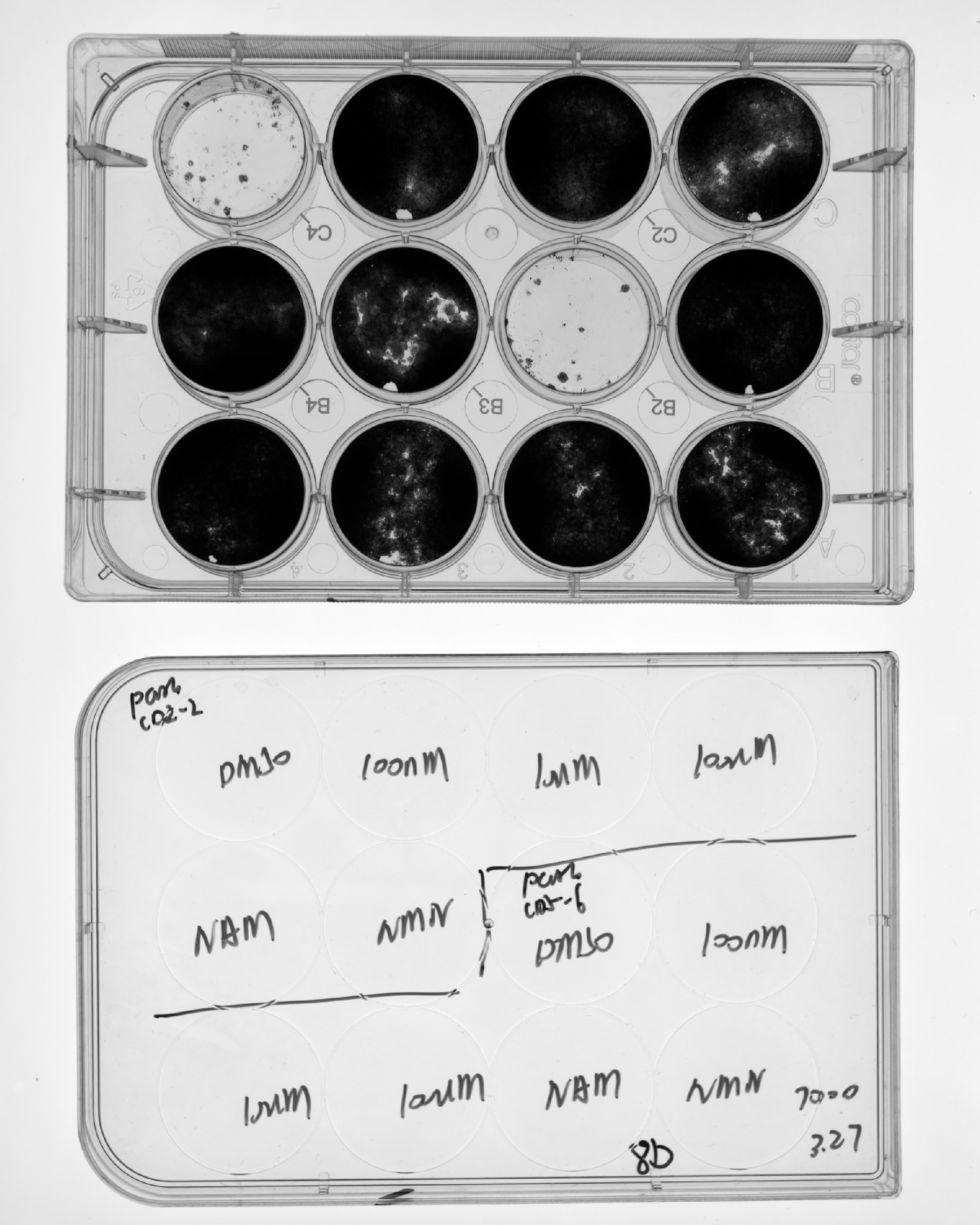

Supplement: Figure 7—source data 1. [file elife-89303-fig7-data1.zip › Figure 7-Source data 1/7C/293A cKO#3_3.tif]

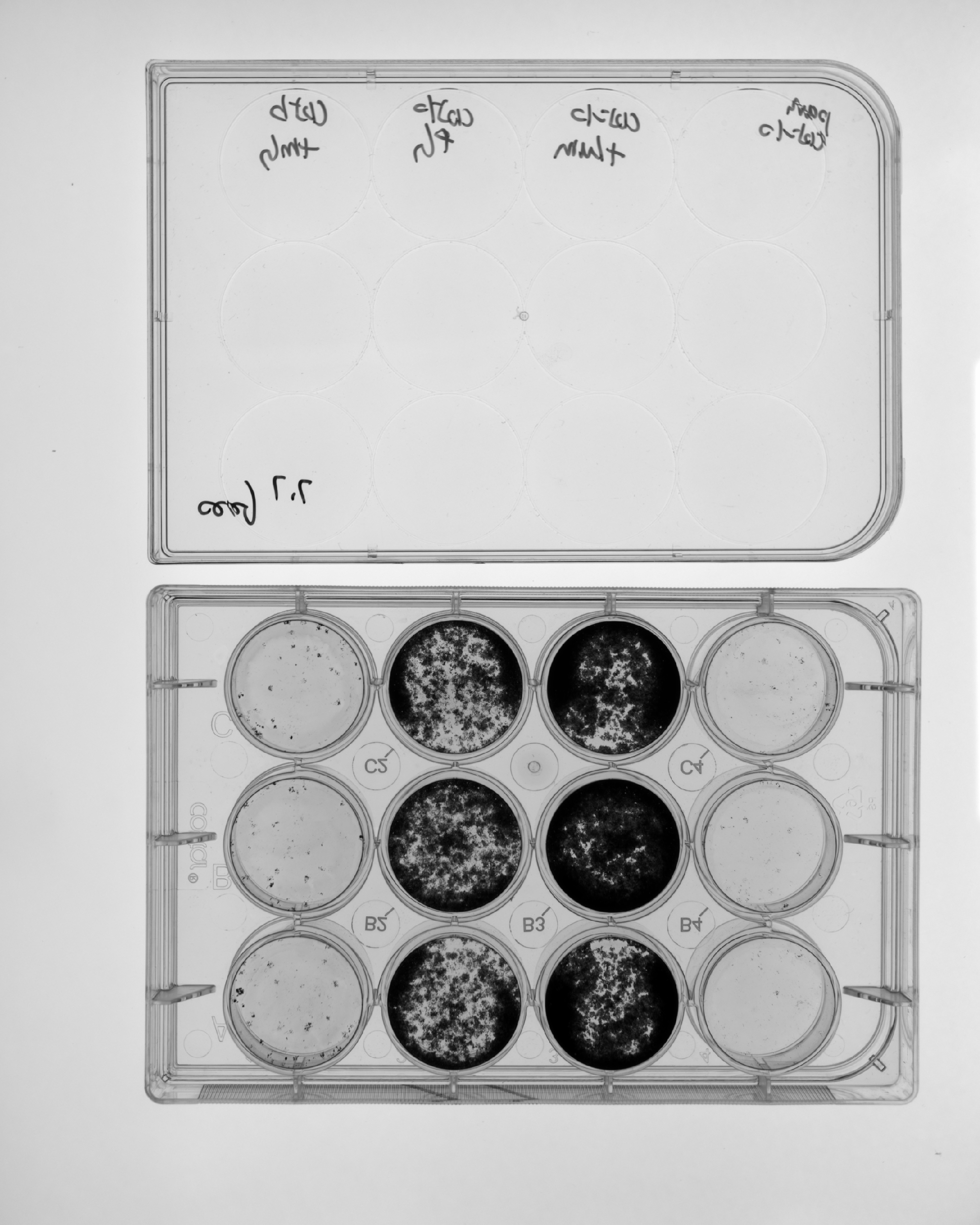

Supplement: Figure 7—source data 1. [file elife-89303-fig7-data1.zip › Figure 7-Source data 1/7C/293A cKO#4.tif]

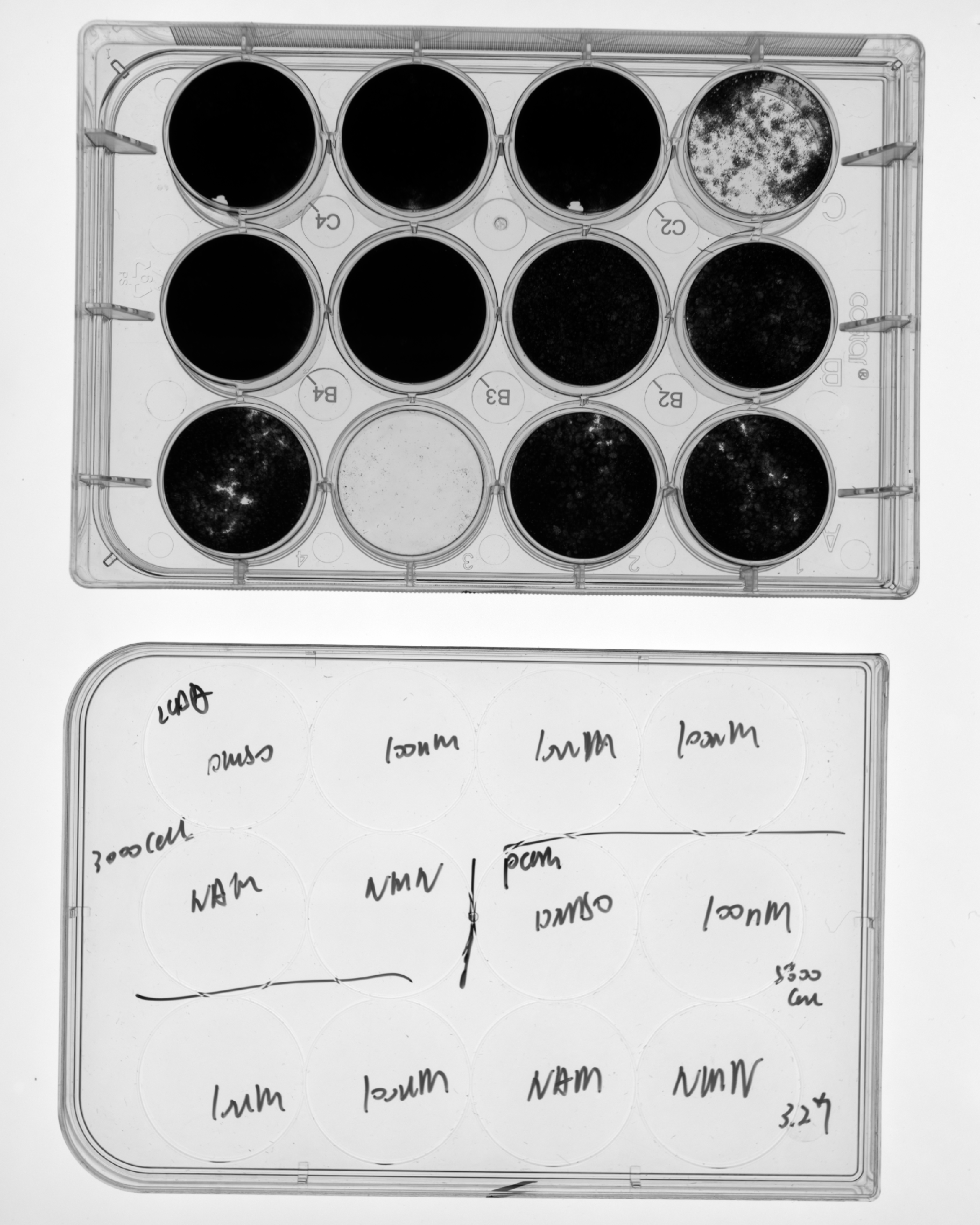

Supplement: Figure 7—source data 1. [file elife-89303-fig7-data1.zip › Figure 7-Source data 1/7C/293A_1.tif]

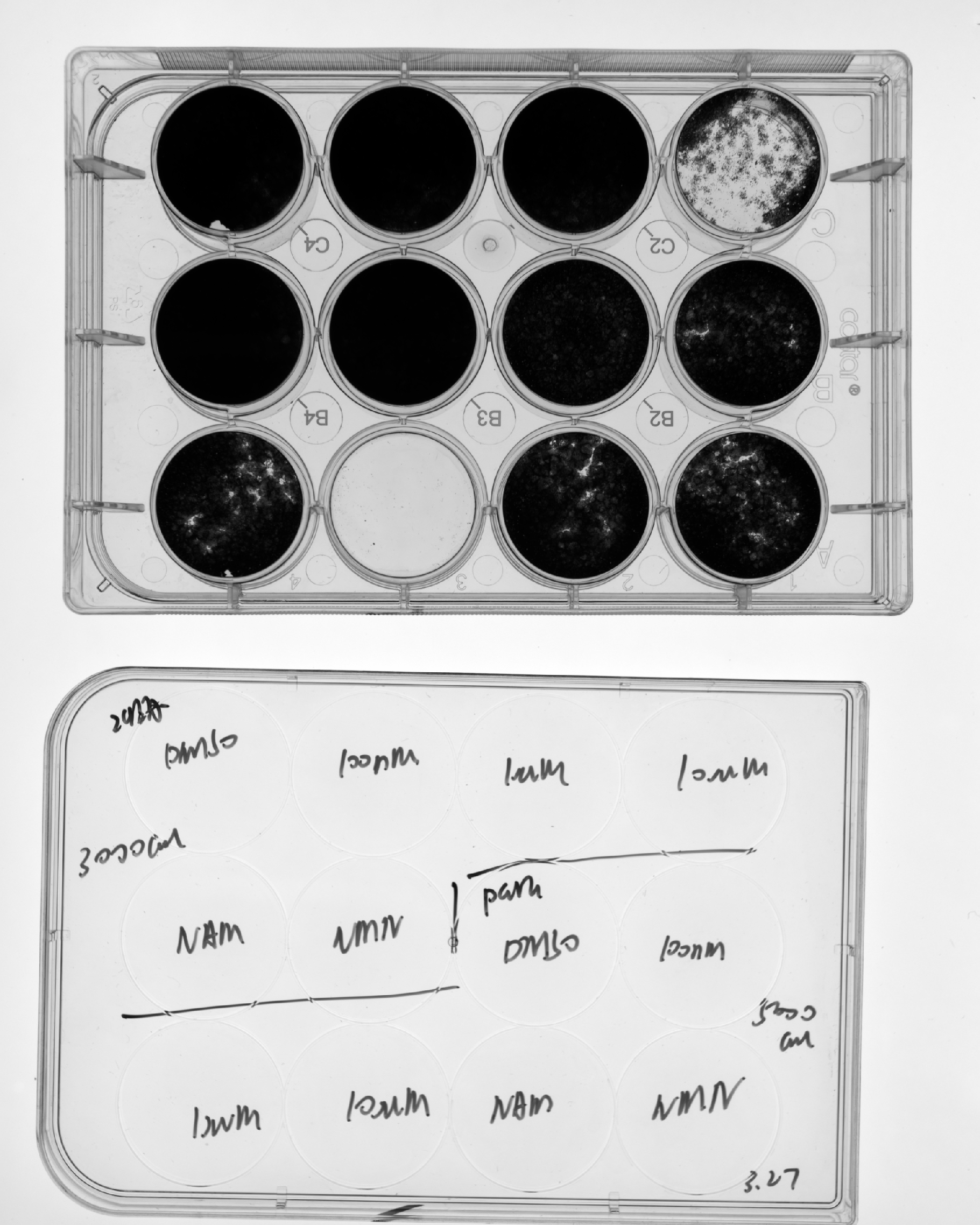

Supplement: Figure 7—source data 1. [file elife-89303-fig7-data1.zip › Figure 7-Source data 1/7C/293A_2.tif]

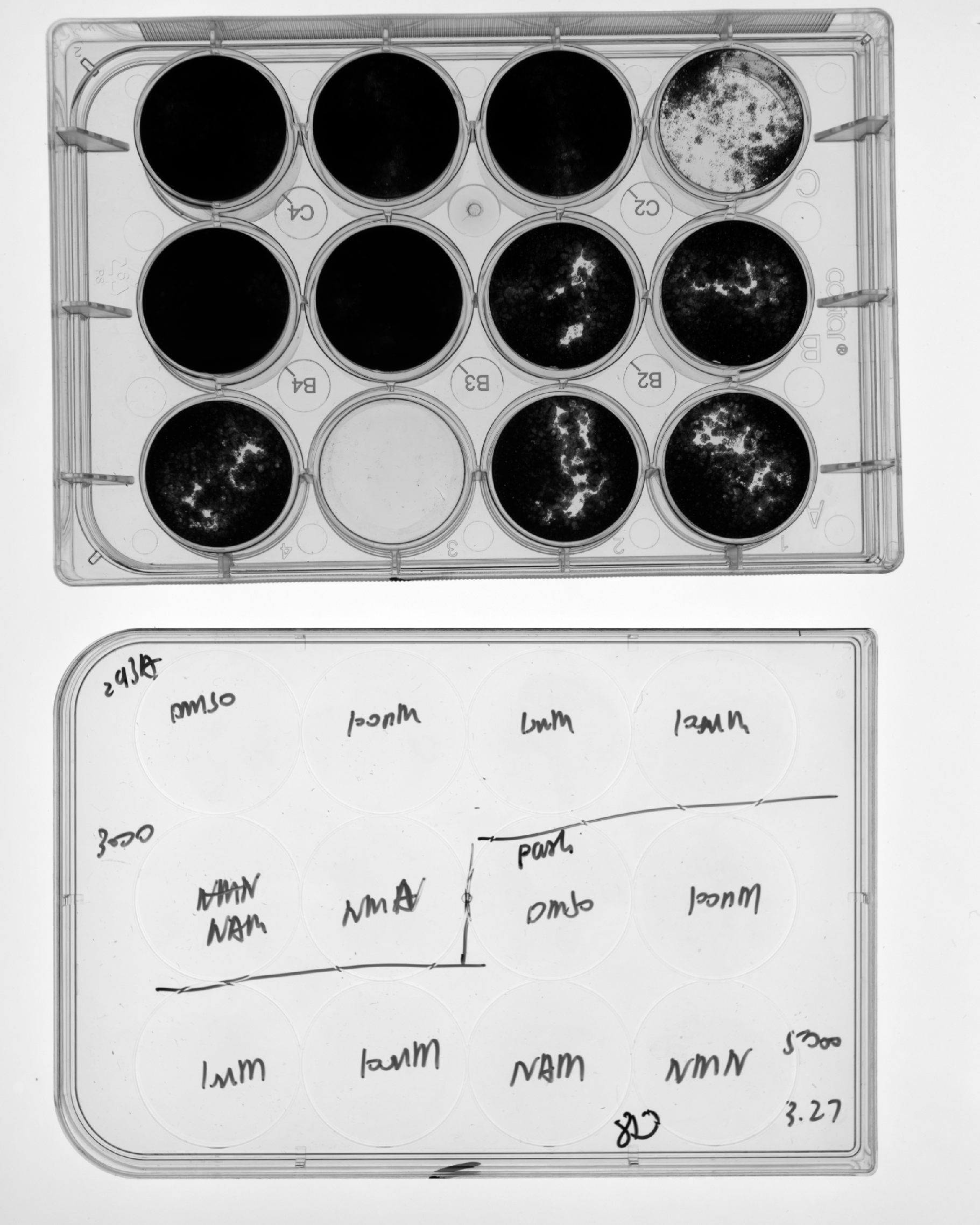

Supplement: Figure 7—source data 1. [file elife-89303-fig7-data1.zip › Figure 7-Source data 1/7C/293A_3.tif]

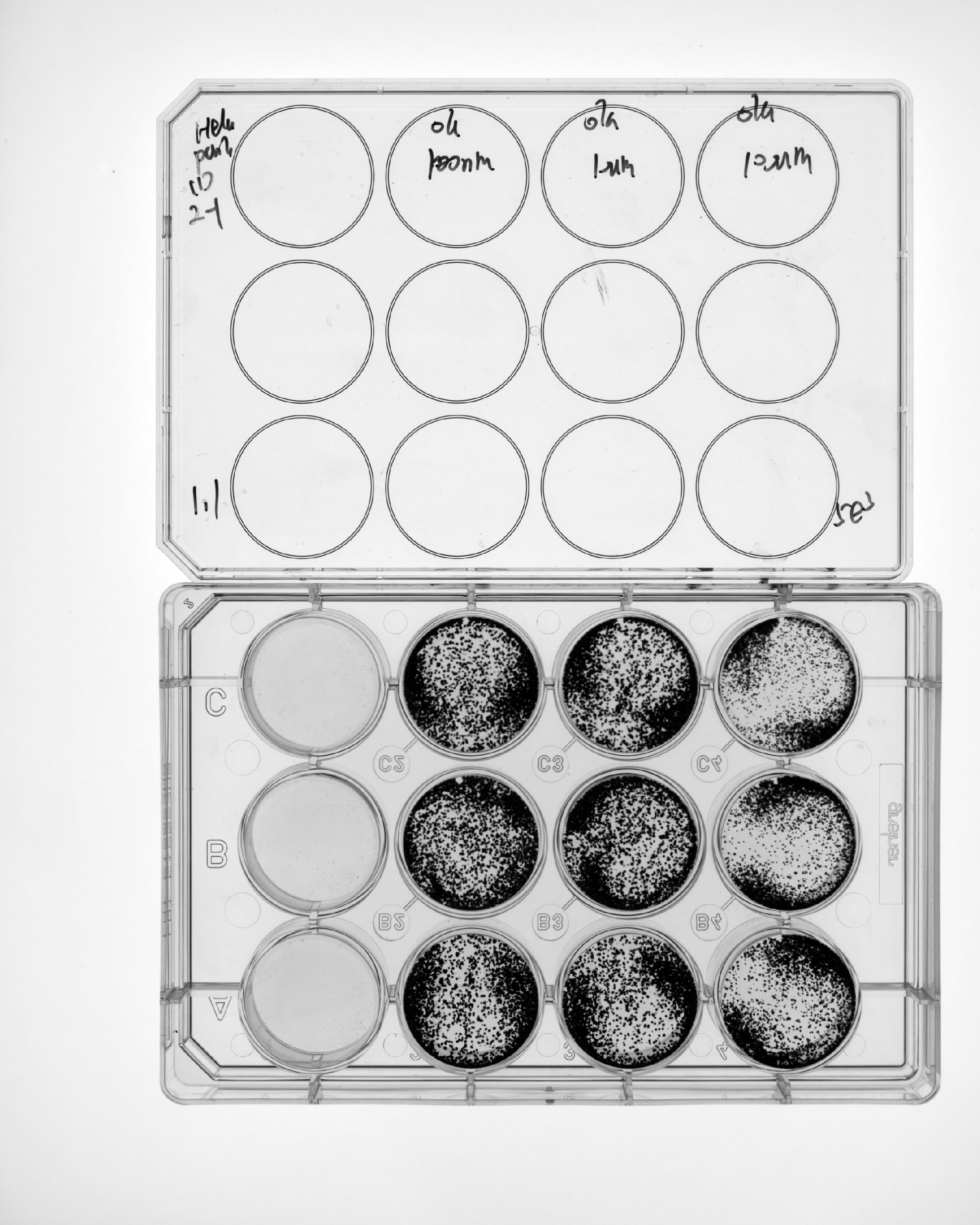

Supplement: Figure 7—source data 1. [file elife-89303-fig7-data1.zip › Figure 7-Source data 1/7C/Hela cKO#3.tif]

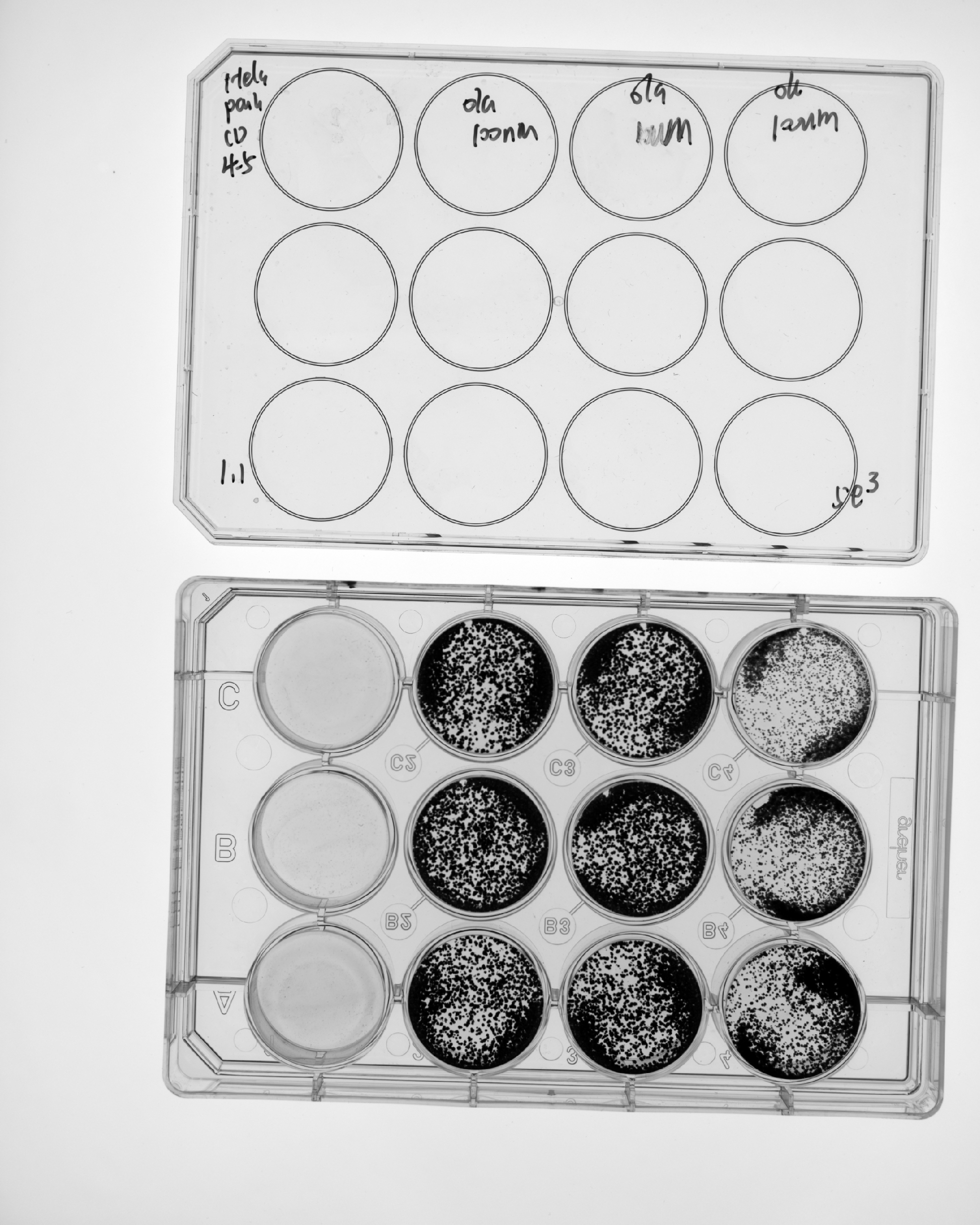

Supplement: Figure 7—source data 1. [file elife-89303-fig7-data1.zip › Figure 7-Source data 1/7C/HeLa cKO#4.tif]

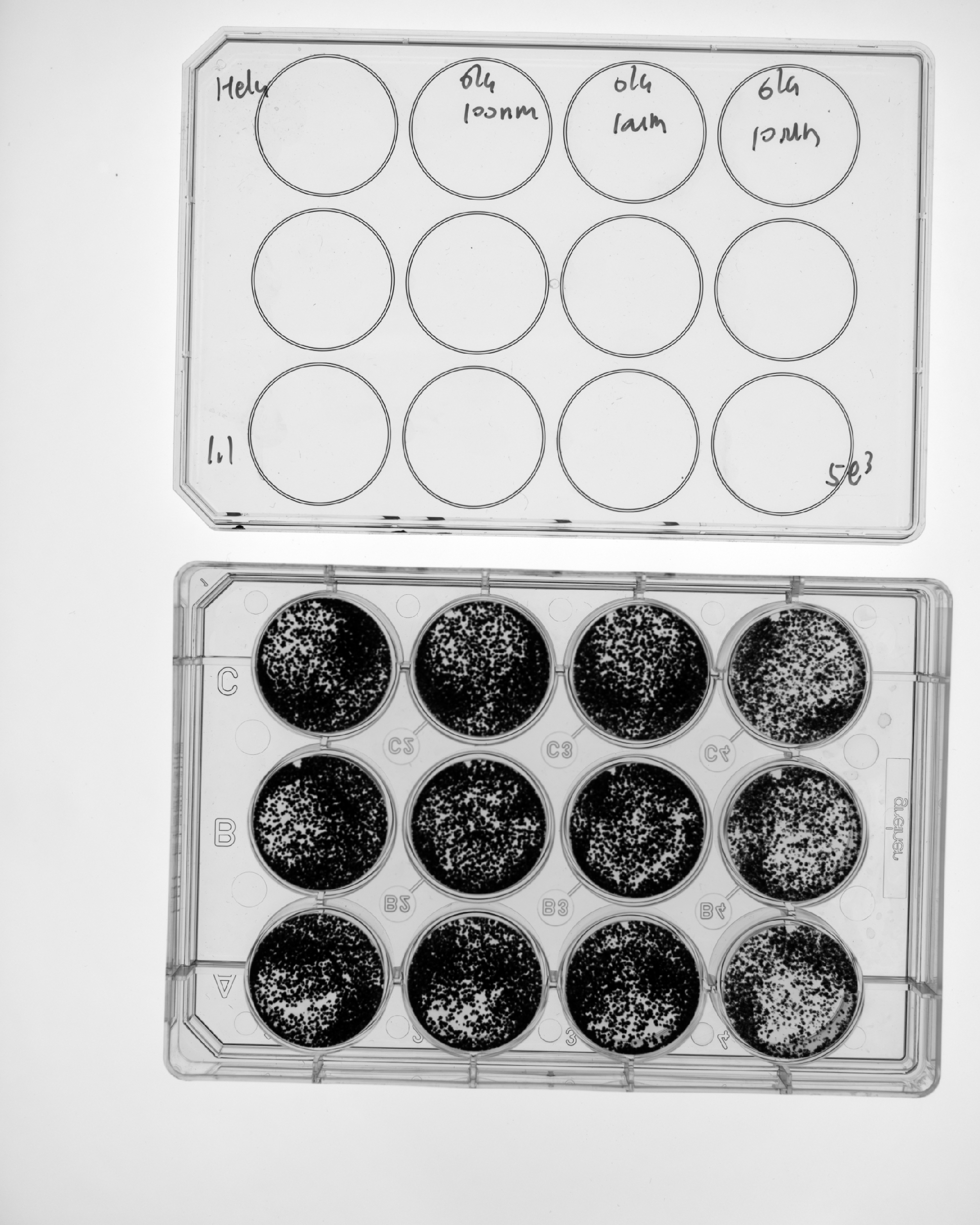

Supplement: Figure 7—source data 1. [file elife-89303-fig7-data1.zip › Figure 7-Source data 1/7C/HeLa.tif]

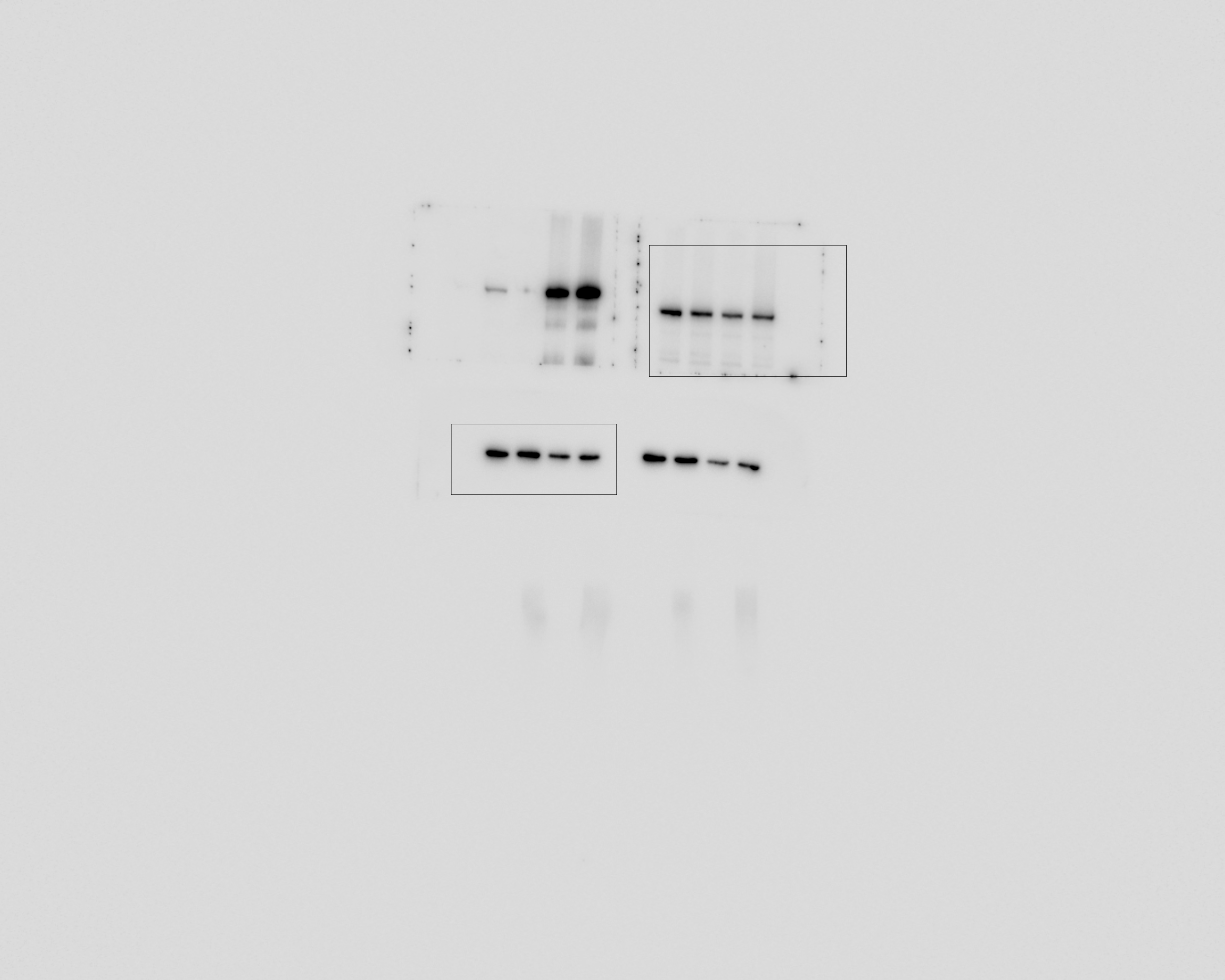

Supplement: Figure 7—source data 1. [file elife-89303-fig7-data1.zip › Figure 7-Source data 1/7D/Actin&PARP1.tif]

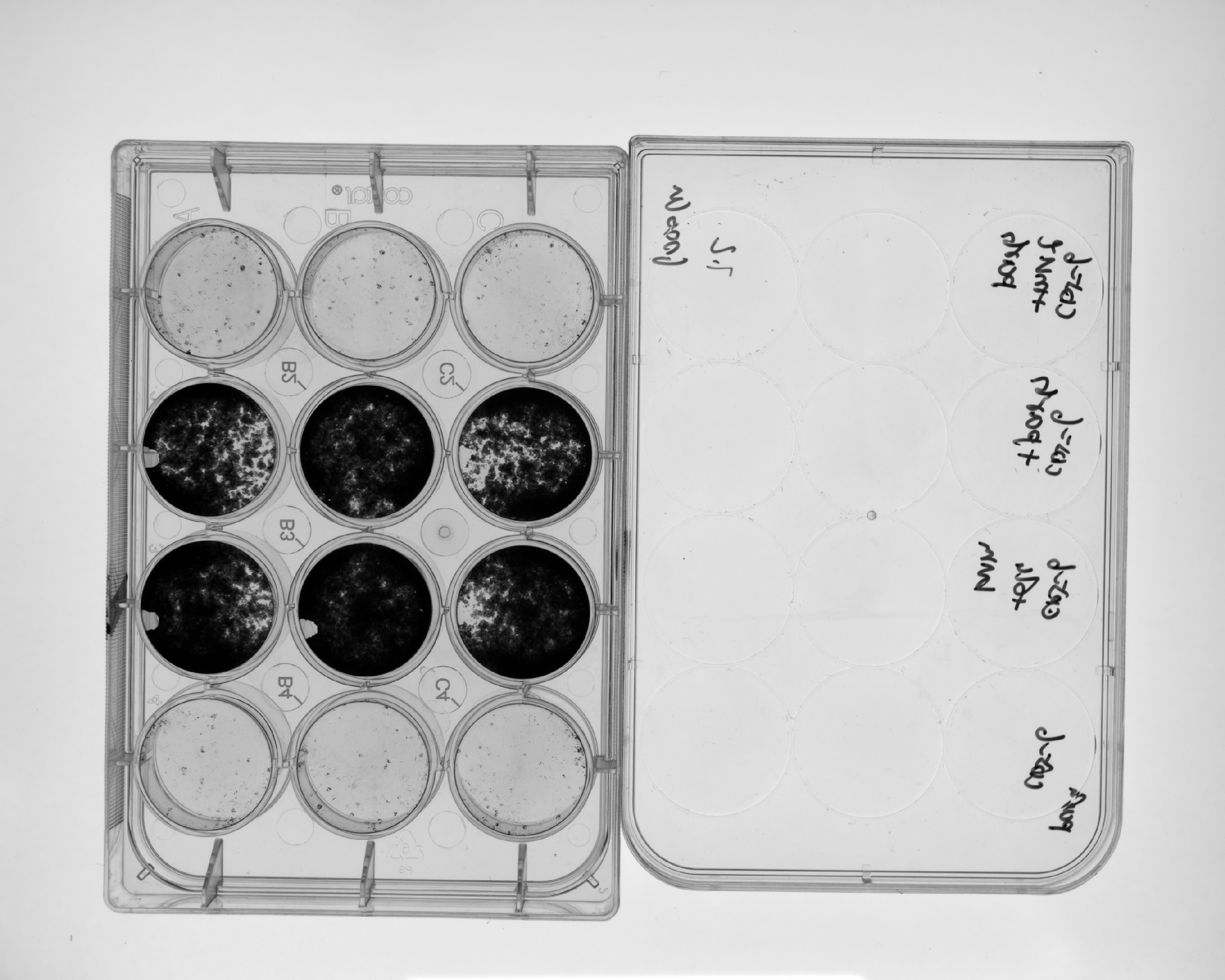

Supplement: Figure 7—source data 1. [file elife-89303-fig7-data1.zip › Figure 7-Source data 1/7D/litong nie 2022-07-10 19h09m50s(Coomassie Blue).tif]

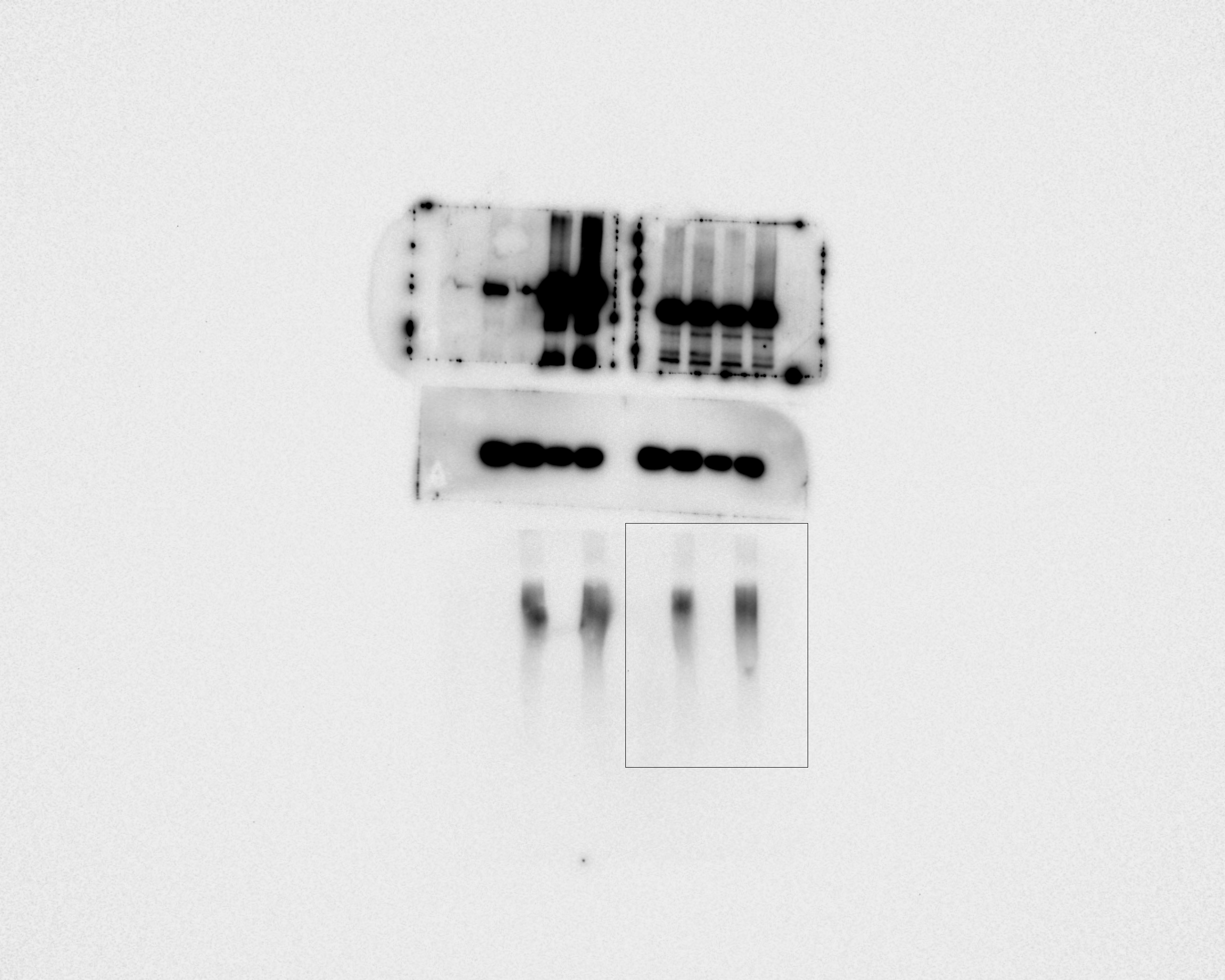

Supplement: Figure 7—source data 1. [file elife-89303-fig7-data1.zip › Figure 7-Source data 1/7D/pADPr.tif]

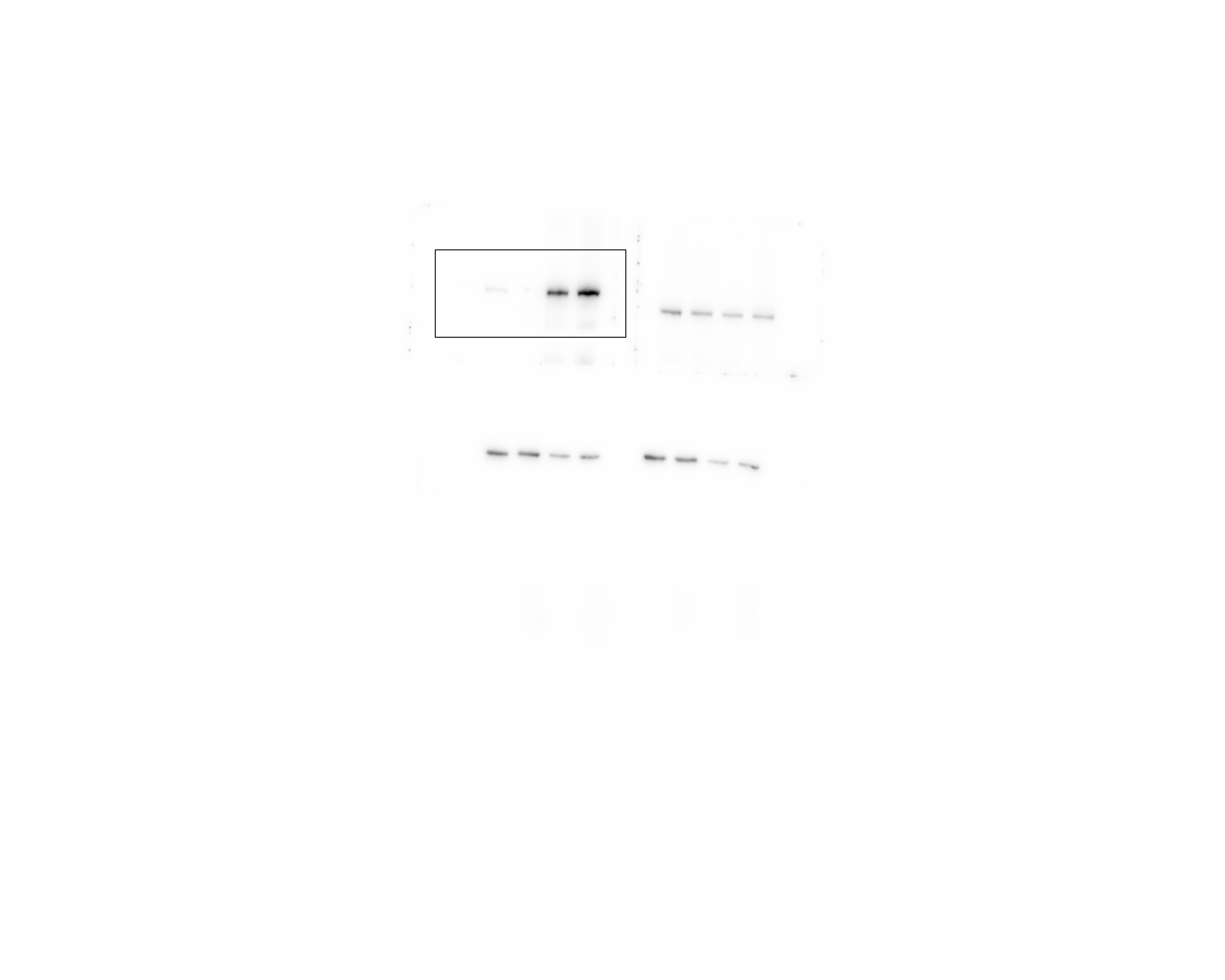

Supplement: Figure 7—source data 1. [file elife-89303-fig7-data1.zip › Figure 7-Source data 1/7D/PARG.tif]

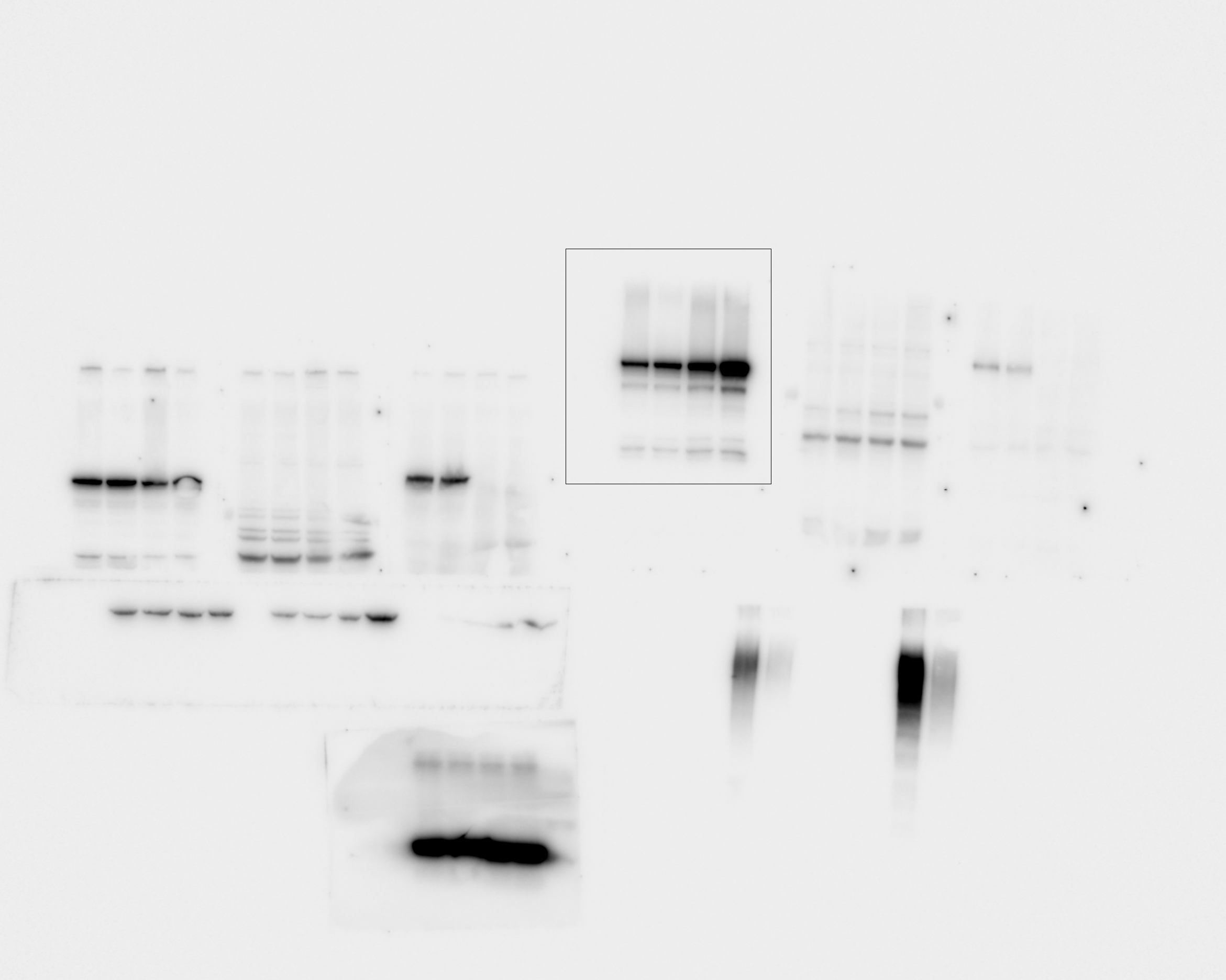

Supplement: Figure 7—source data 1. [file elife-89303-fig7-data1.zip › Figure 7-Source data 1/7G/Chr_PARP1.tif]

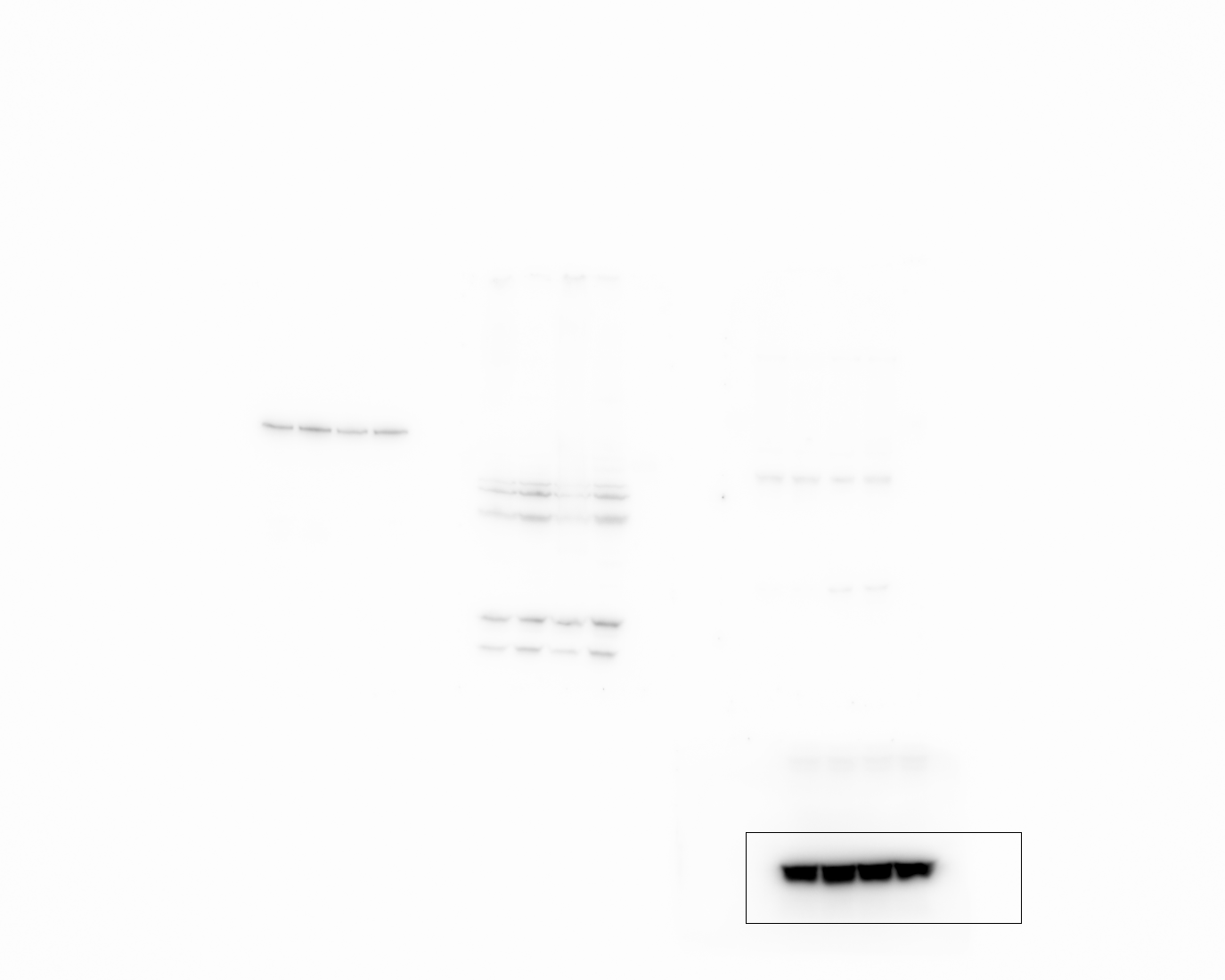

Supplement: Figure 7—source data 1. [file elife-89303-fig7-data1.zip › Figure 7-Source data 1/7G/H3.tif]

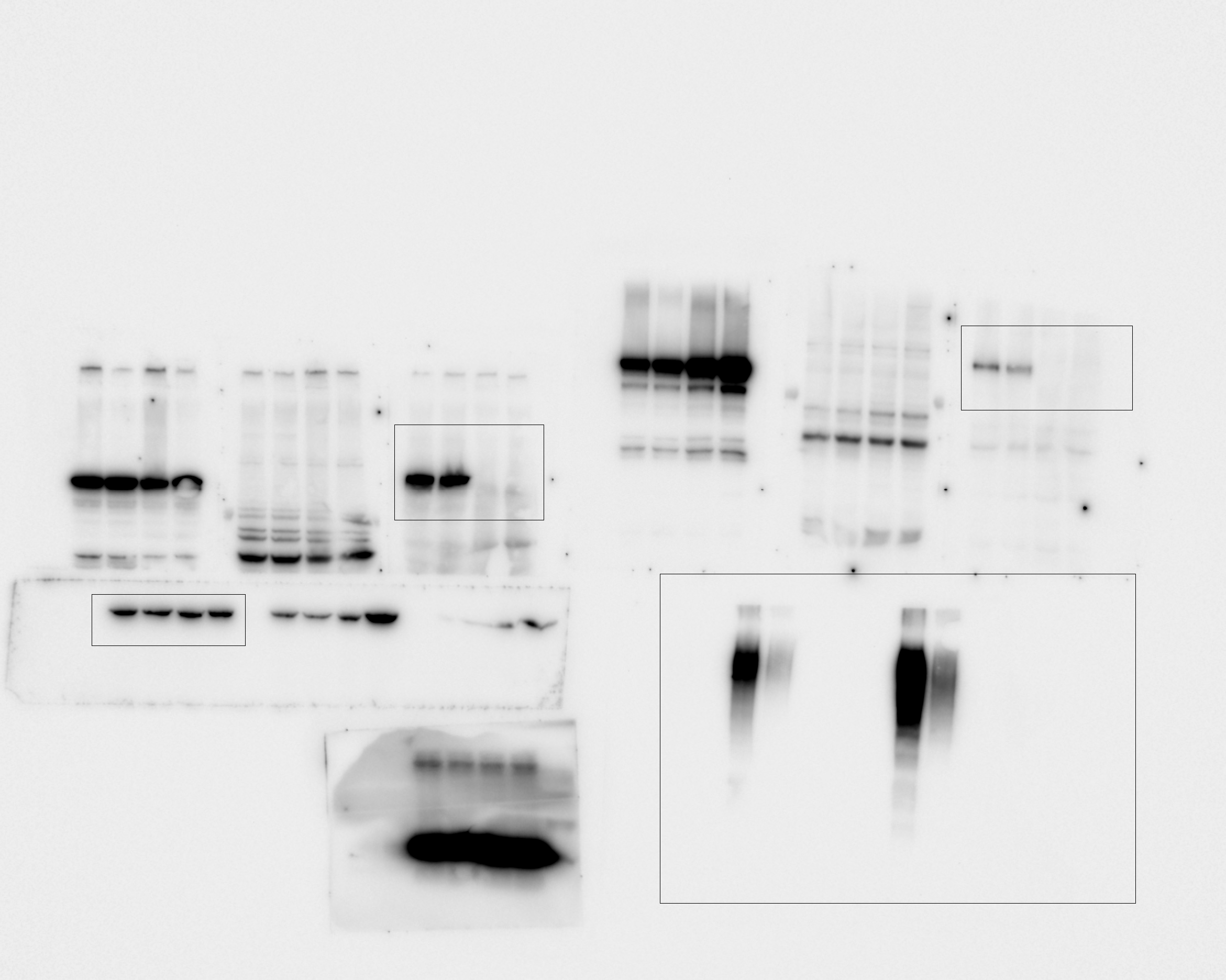

Supplement: Figure 7—source data 1. [file elife-89303-fig7-data1.zip › Figure 7-Source data 1/7G/PARG&pADPr&actin.tif]

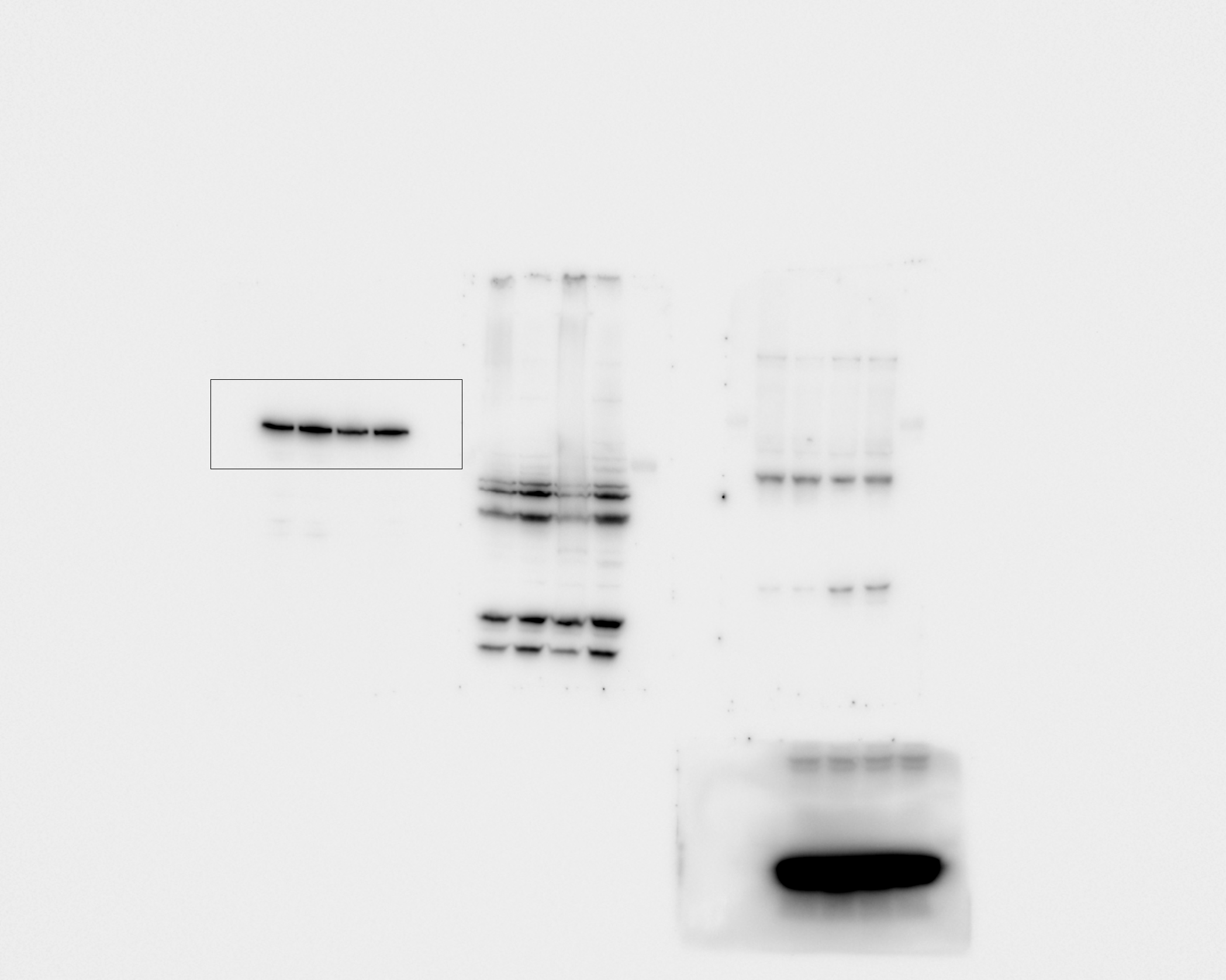

Supplement: Figure 7—source data 1. [file elife-89303-fig7-data1.zip › Figure 7-Source data 1/7G/Sol_PARP1.tif]

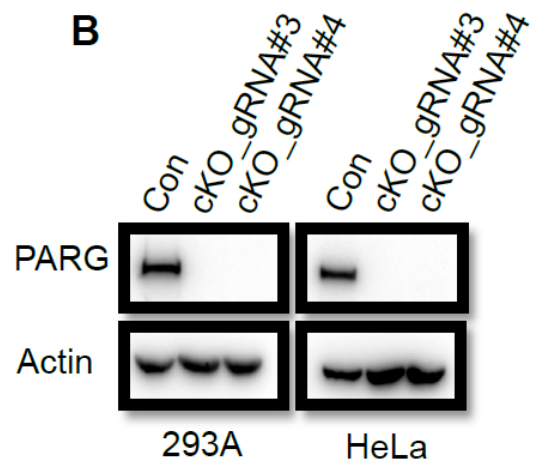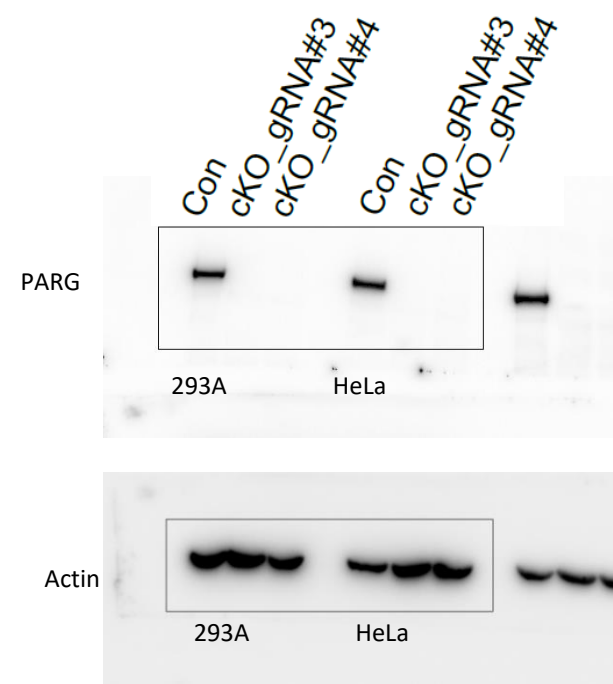

Figure 7

**C**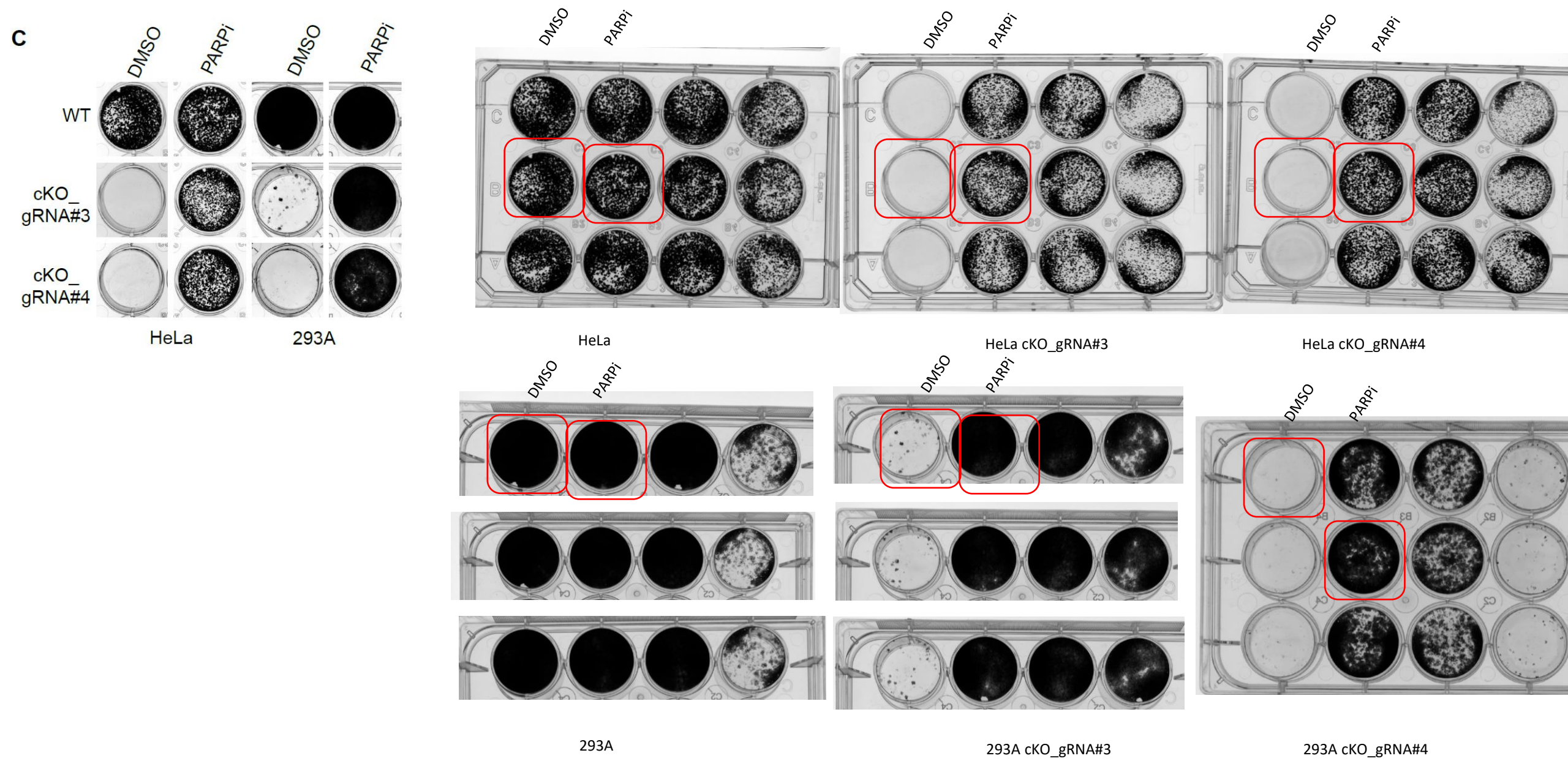

Figure 7

D

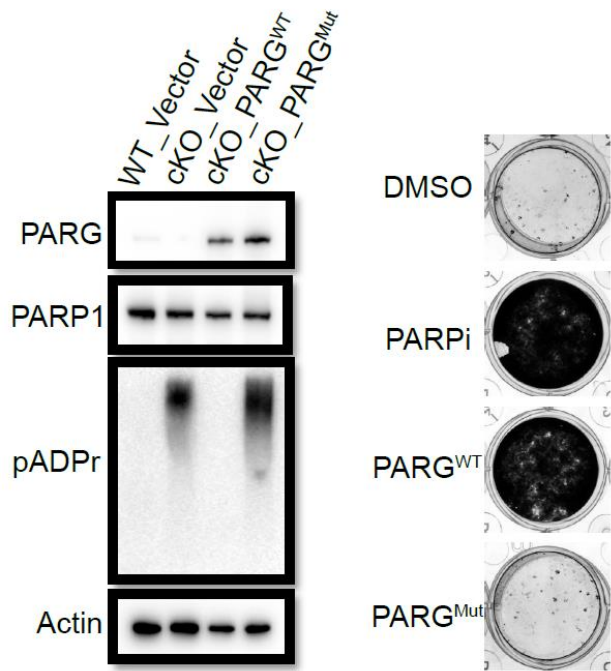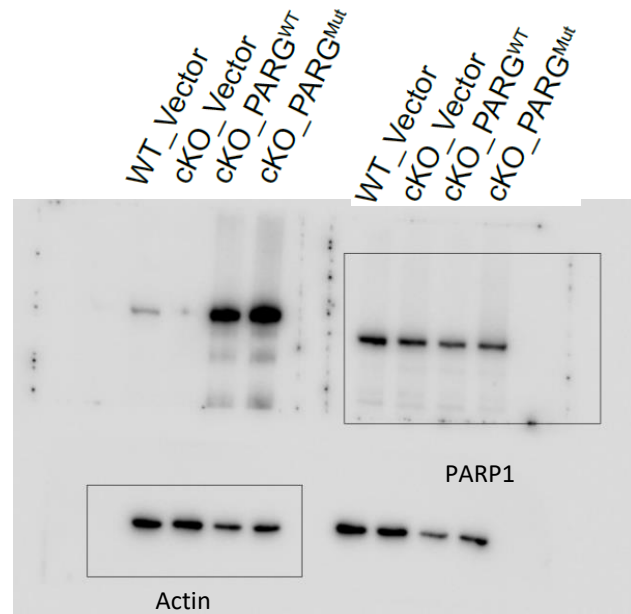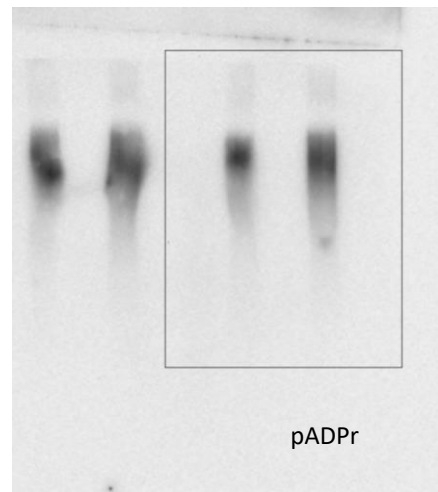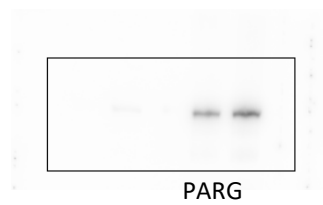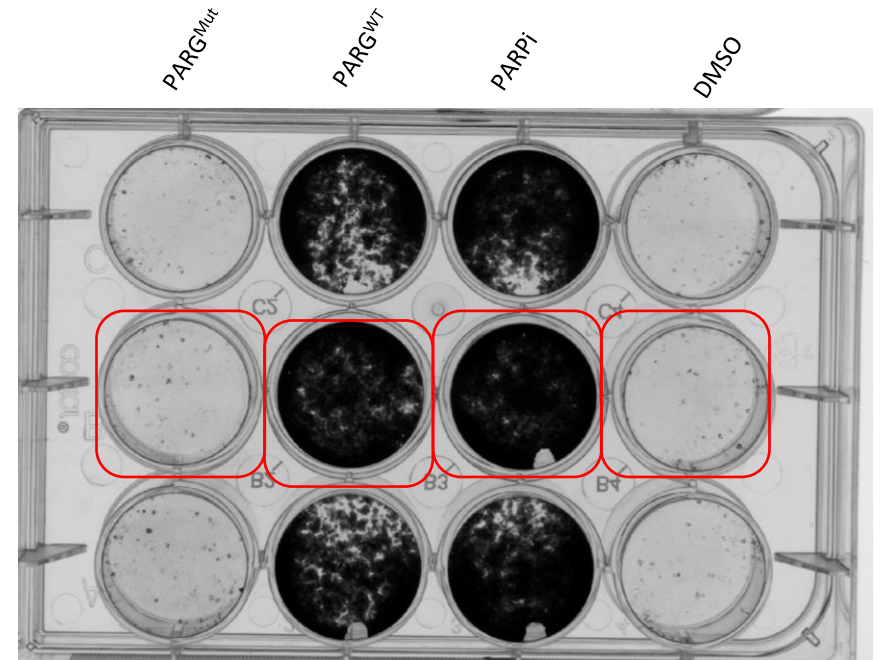

Figure 7

E

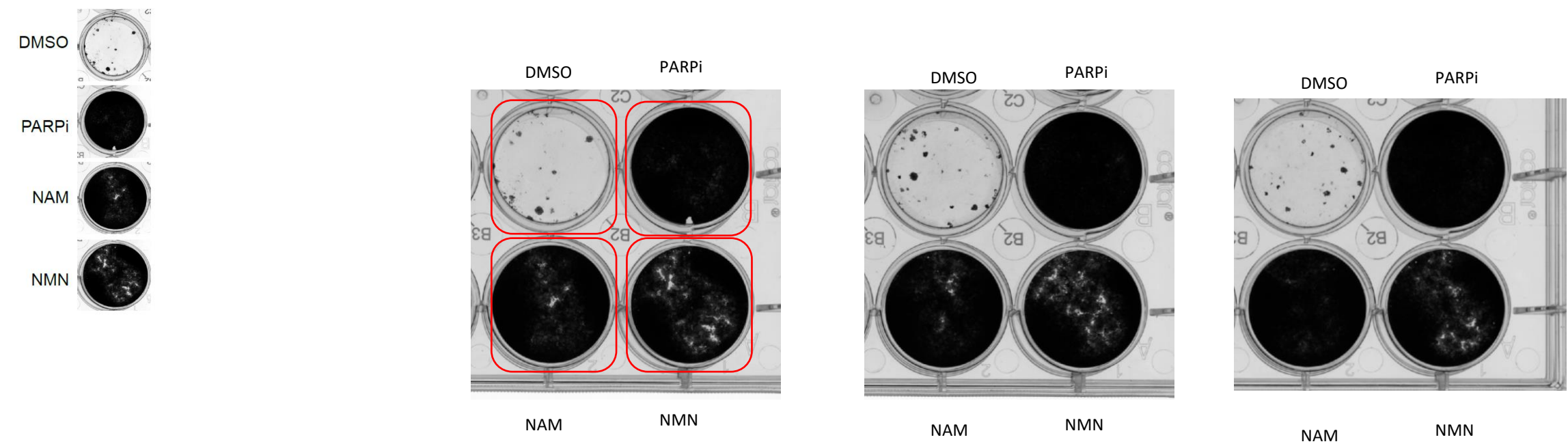

Figure 7

G

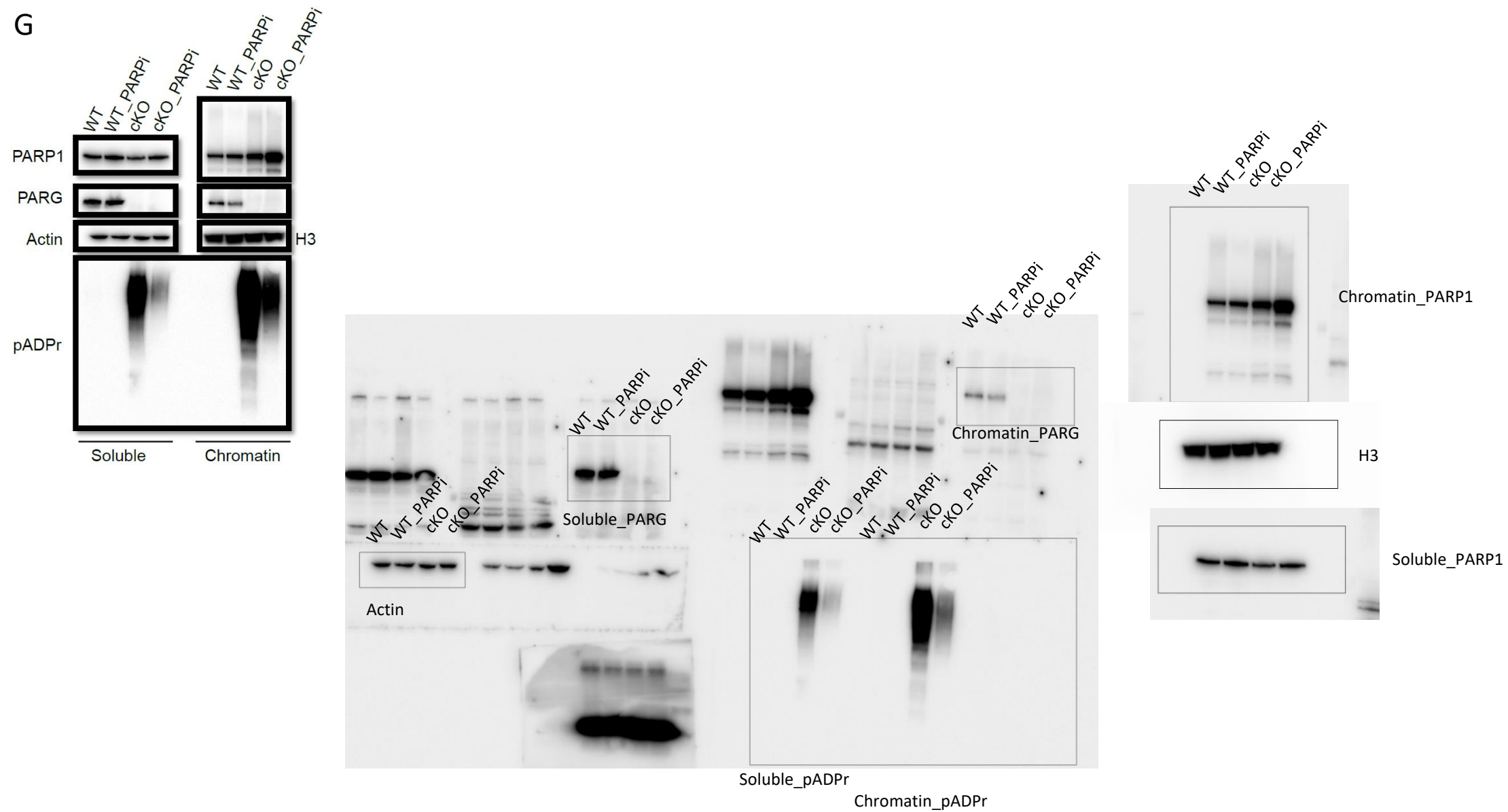

Figure 7

Supplement: Figure 7—source data 2. [file elife-89303-fig7-data2.zip › Figure 7-Source data 2/Figure 7-Source data 2.pdf]

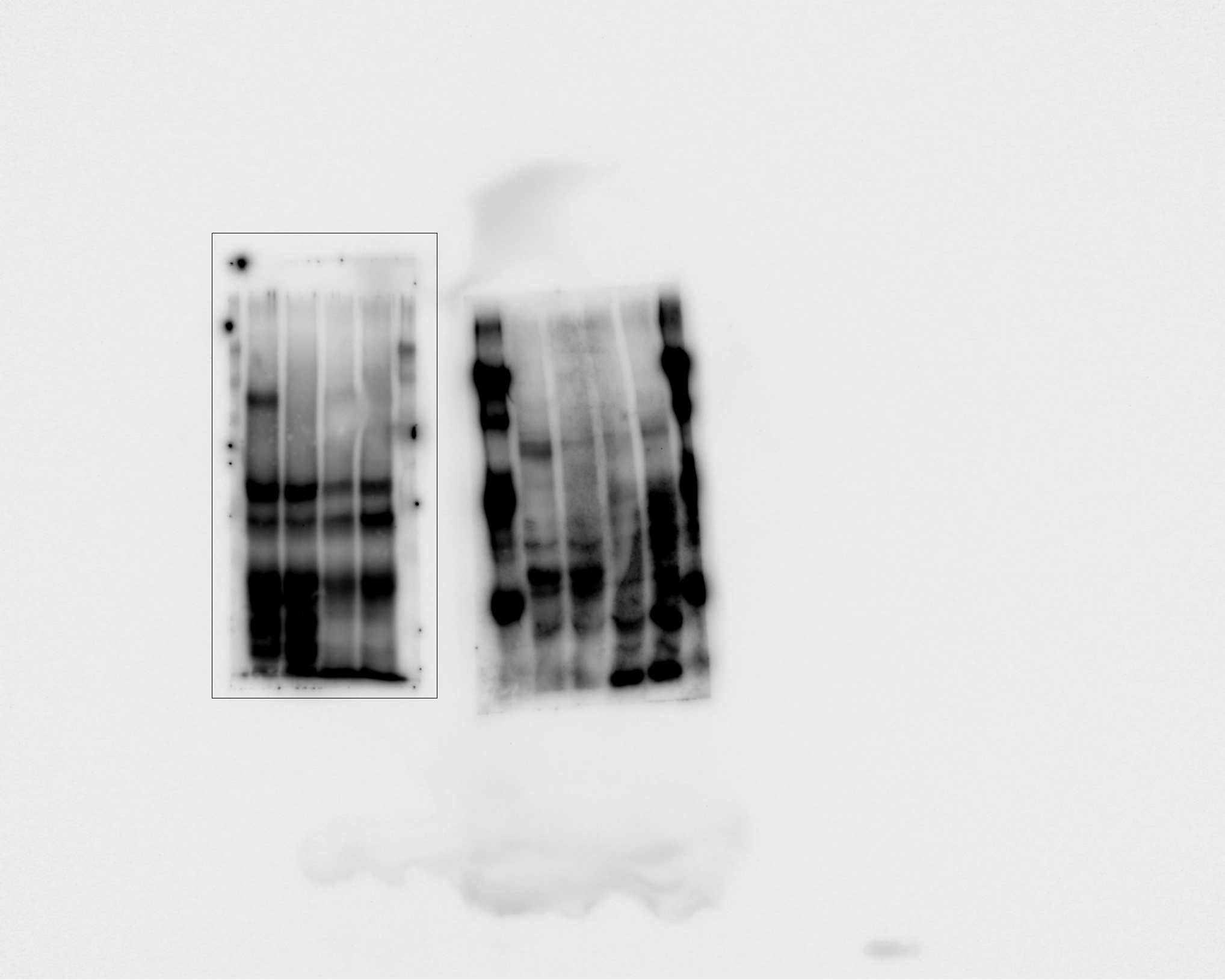

Supplement: Figure 7—figure supplement 2—source data 1. [file elife-89303-fig7-figsupp2-data1.zip › Figure 7-Figure Supplement 2-Source data 1/Antibody#1.tif]

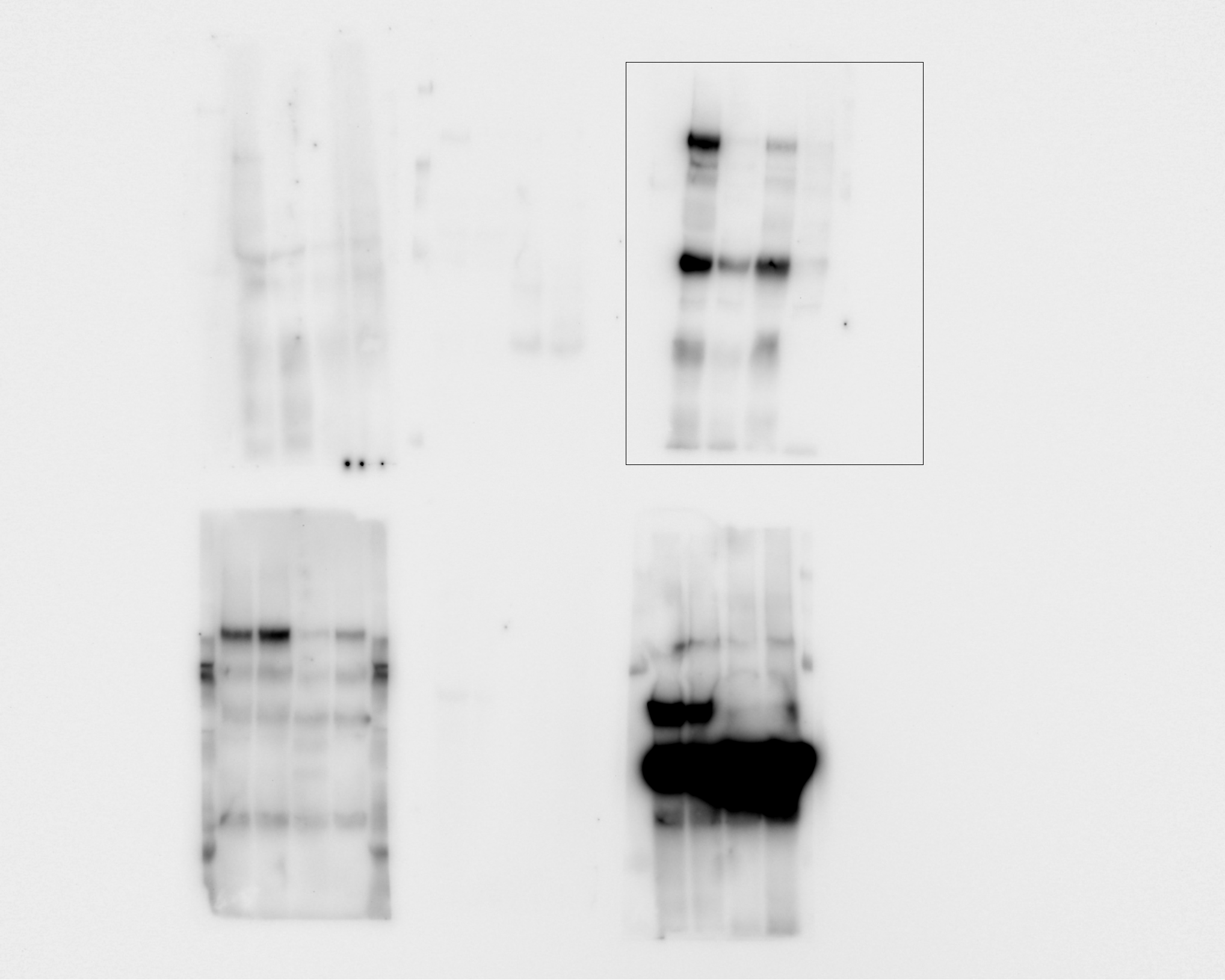

Supplement: Figure 7—figure supplement 2—source data 1. [file elife-89303-fig7-figsupp2-data1.zip › Figure 7-Figure Supplement 2-Source data 1/Antibody#2.tif]

A

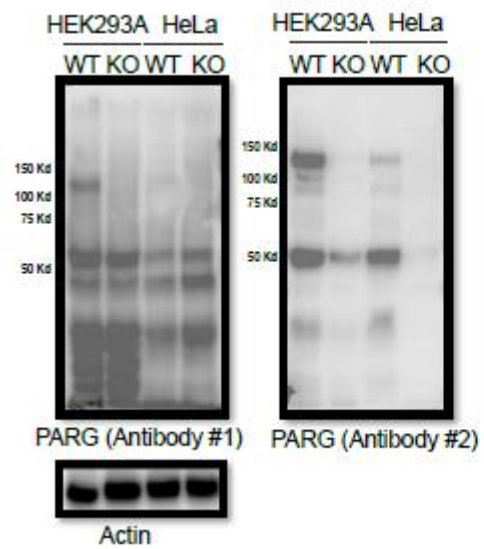

Antibody #1: Anti-AA 677-976  
Antibody #2: Anti-AA 894-976

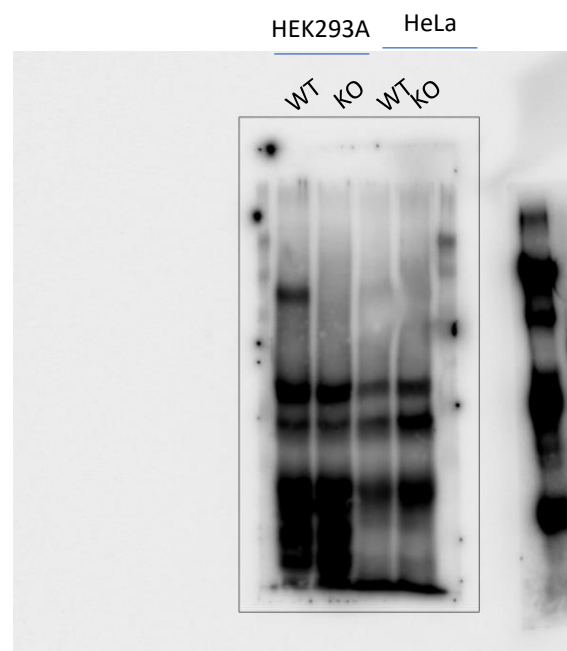

PARG(Antibody #1)

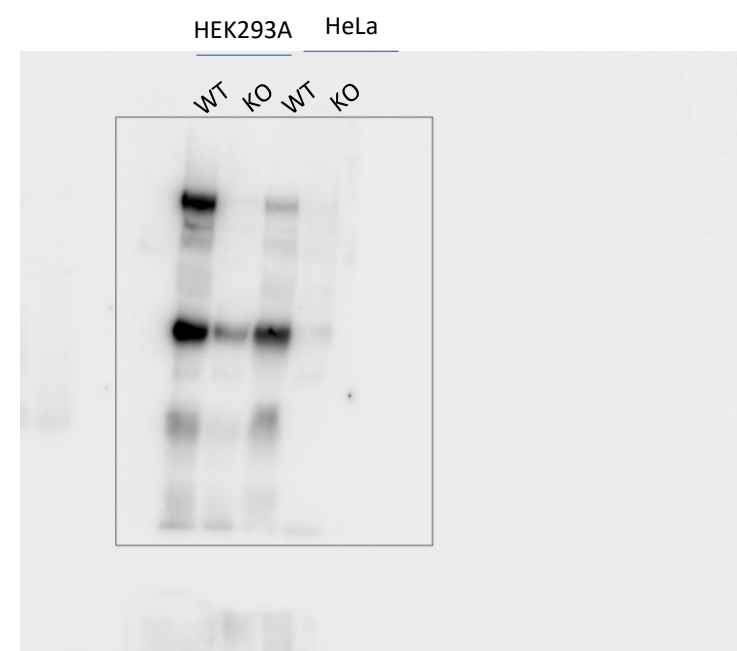

PARG(Antibody #2)

Supplement: Figure 7—figure supplement 2—source data 2. [file elife-89303-fig7-figsupp2-data2.zip › Figure 7-Figure Supplement 2-Source data 2/Figure 7-Figure Supplement 2-Source data 2.pdf]
