# Supplementary material for: DePARylation is critical for S phase progression and cell survival
Source: eLife. 2024 Apr 5;12:RP89303. doi: 10.7554/eLife.89303 (PMC10997334; doi:10.7554/eLife.89303)
Supplement: Figure 6—figure supplement 2—source data 2. [file elife-89303-fig6-figsupp2-data2.zip › Figure 6-Figure Supplement 2-Source data 2/Figure 6-Figure Supplement 2-Source data 2.pdf]

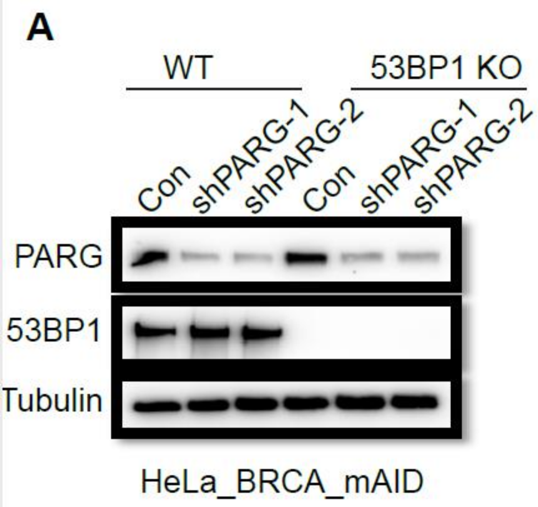

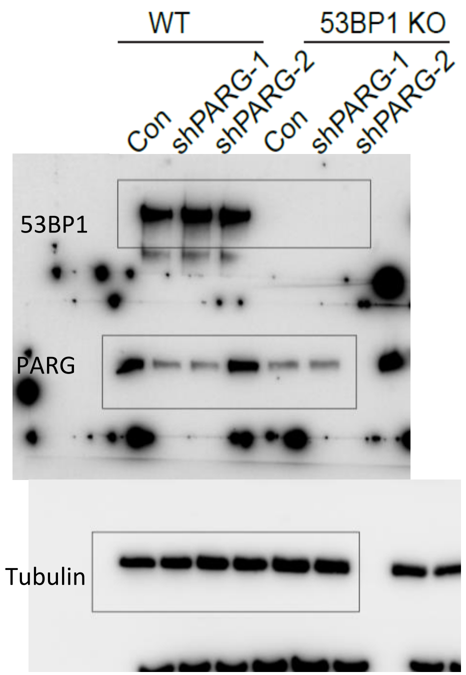

Figure 6-figure supplement 2

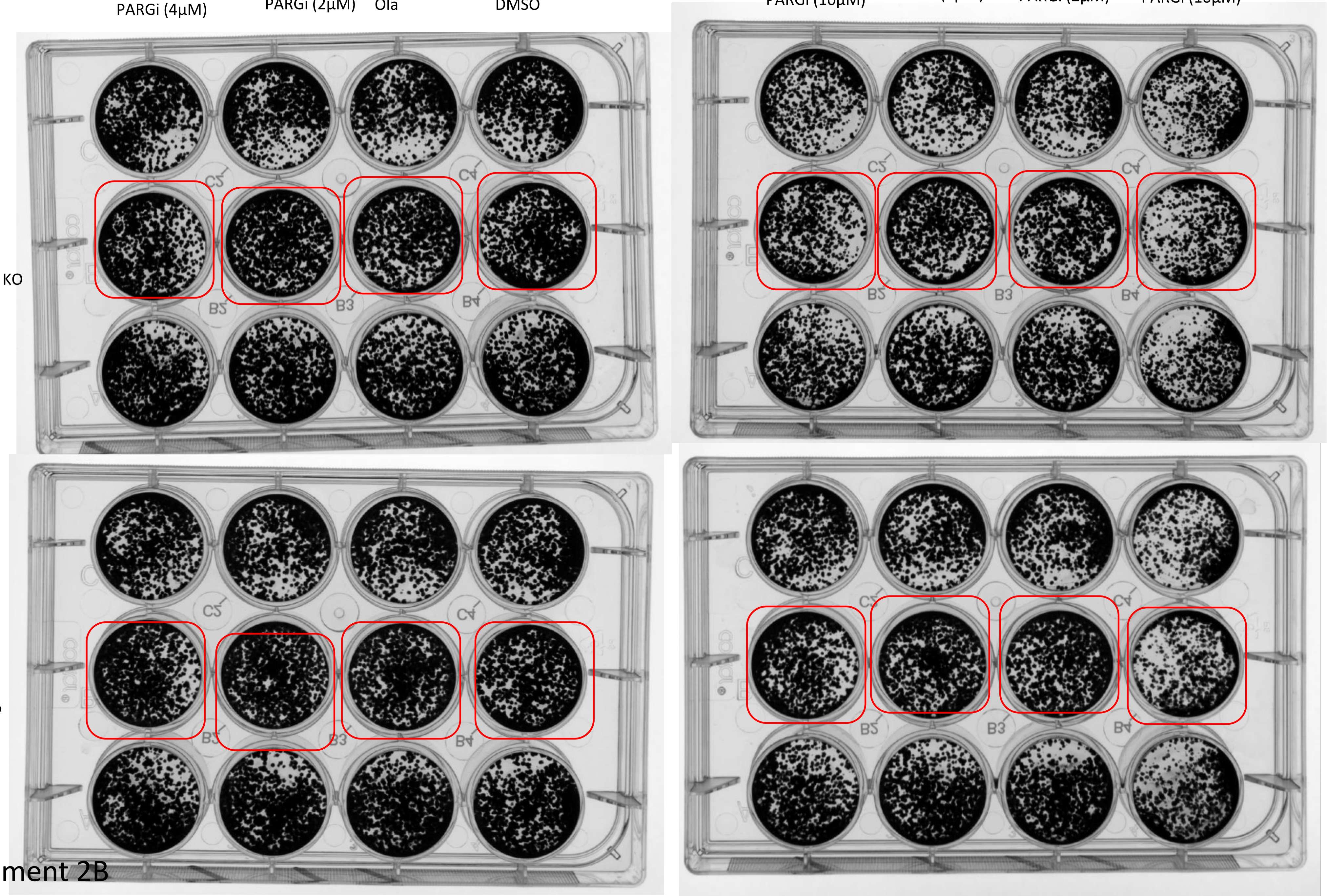

Figure 6-figure supplement 2B

PARGi (4µM) PARGi (2µM) Ola DMSO

Ola+ PARGi (10µM) Ola+ PARGi (4µM) Ola+ PARGi (2µM) PARGi (10µM)

HeLa_BRCA_mAID +IAA&Dox

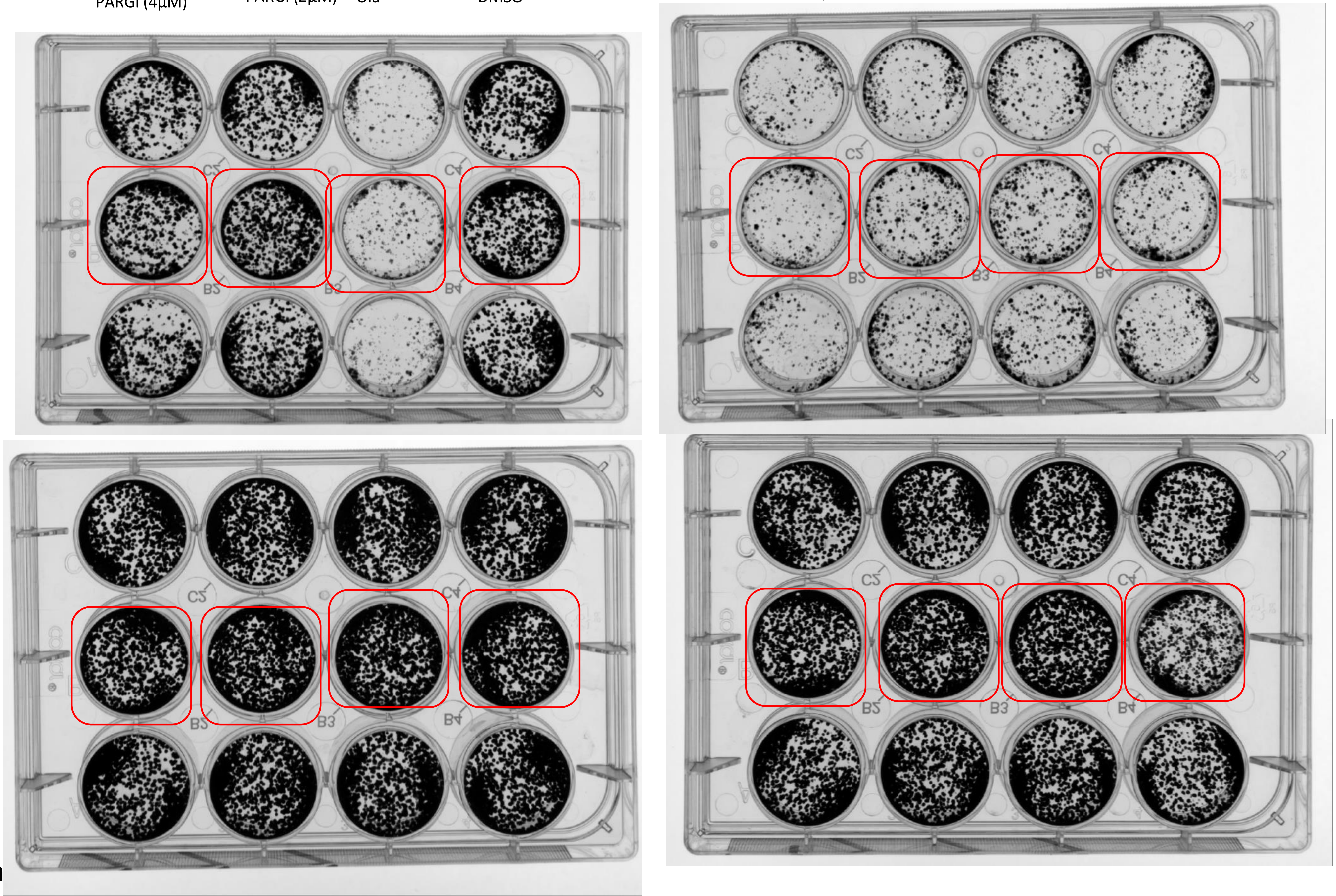

HeLa_BRCA_mAID

Figure 6-figure supplem

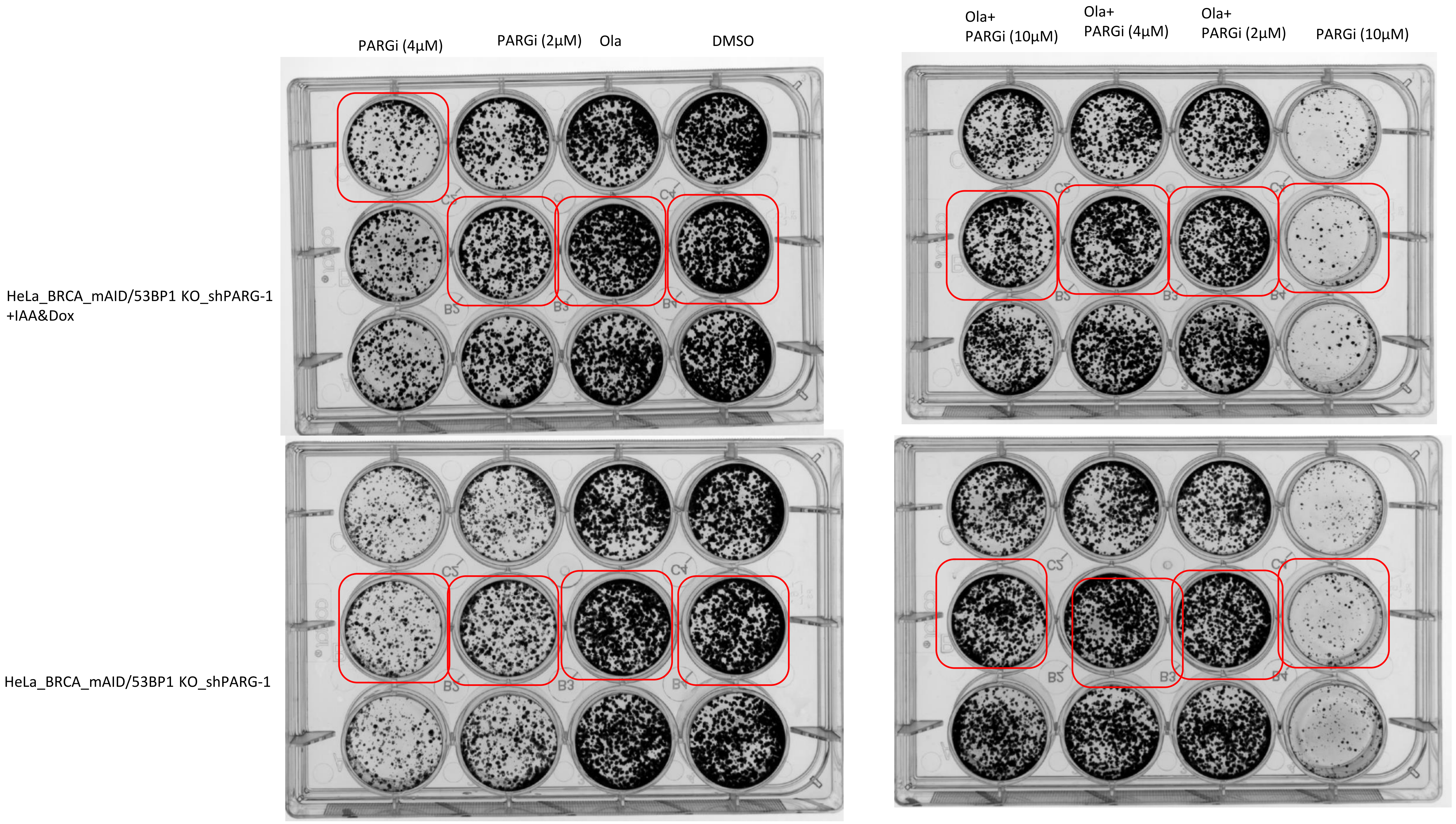

Figure 6-figure supplement 2B

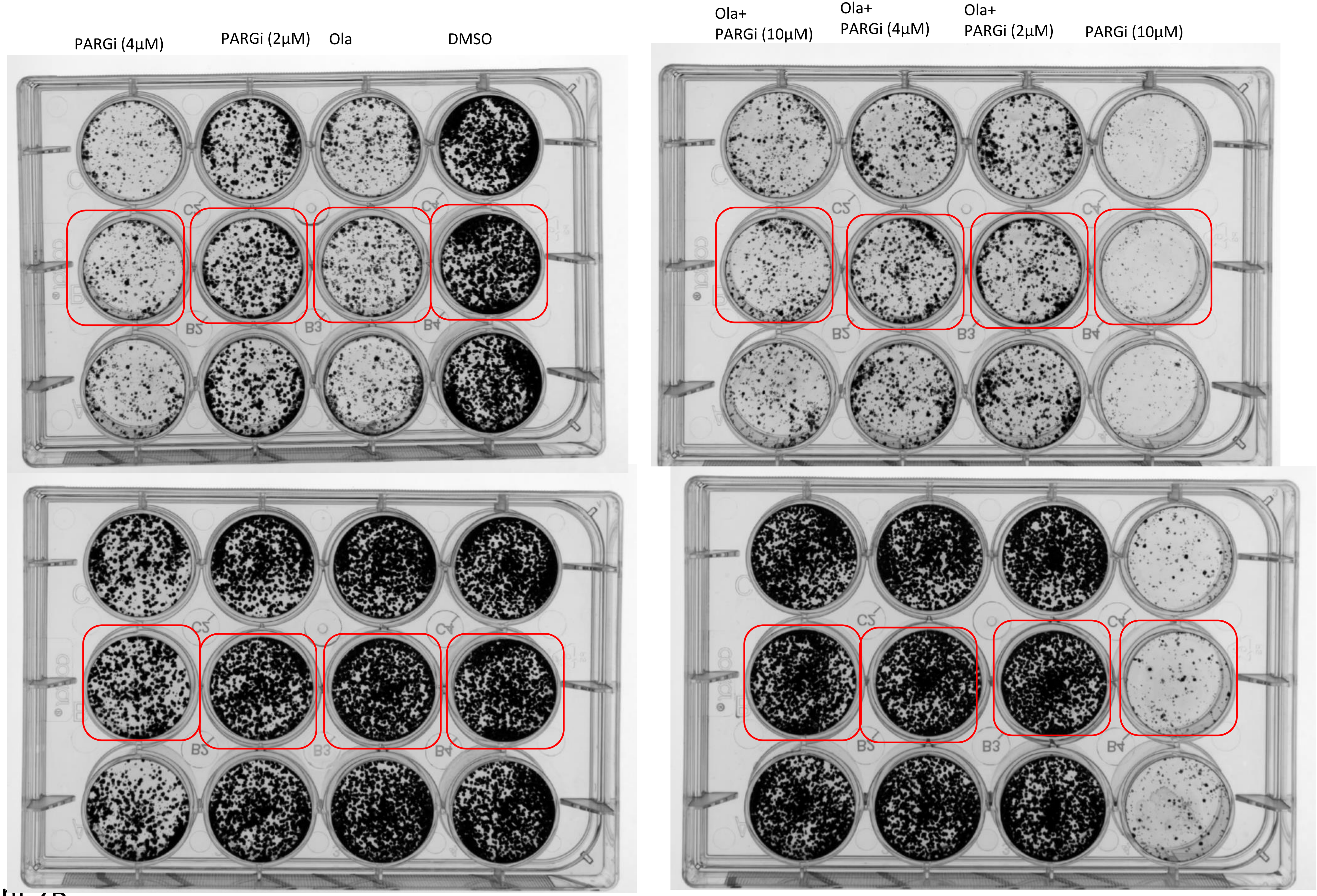

Figure 6-figure supplement 2B

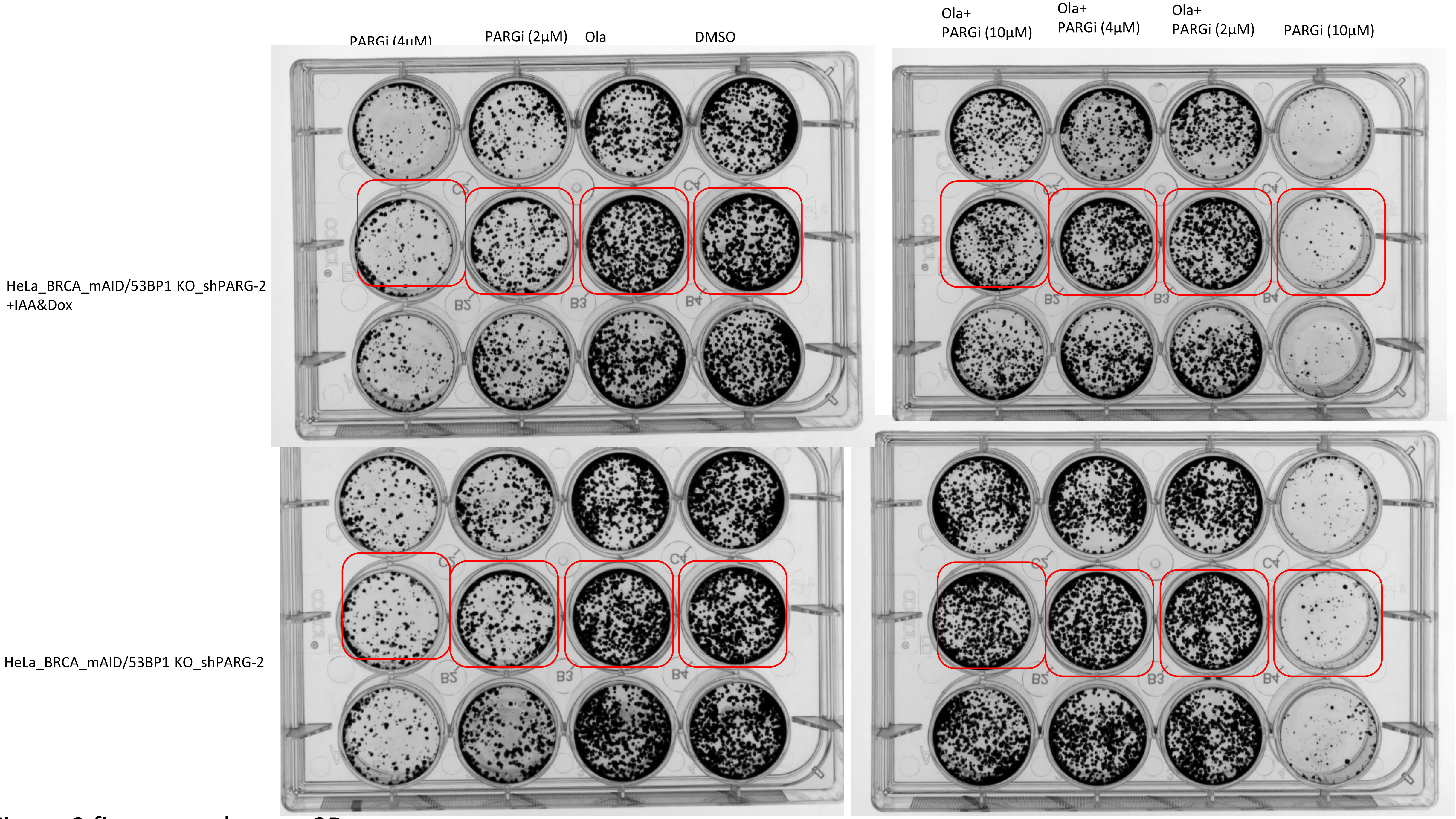

Figure 6-figure supplement 2B

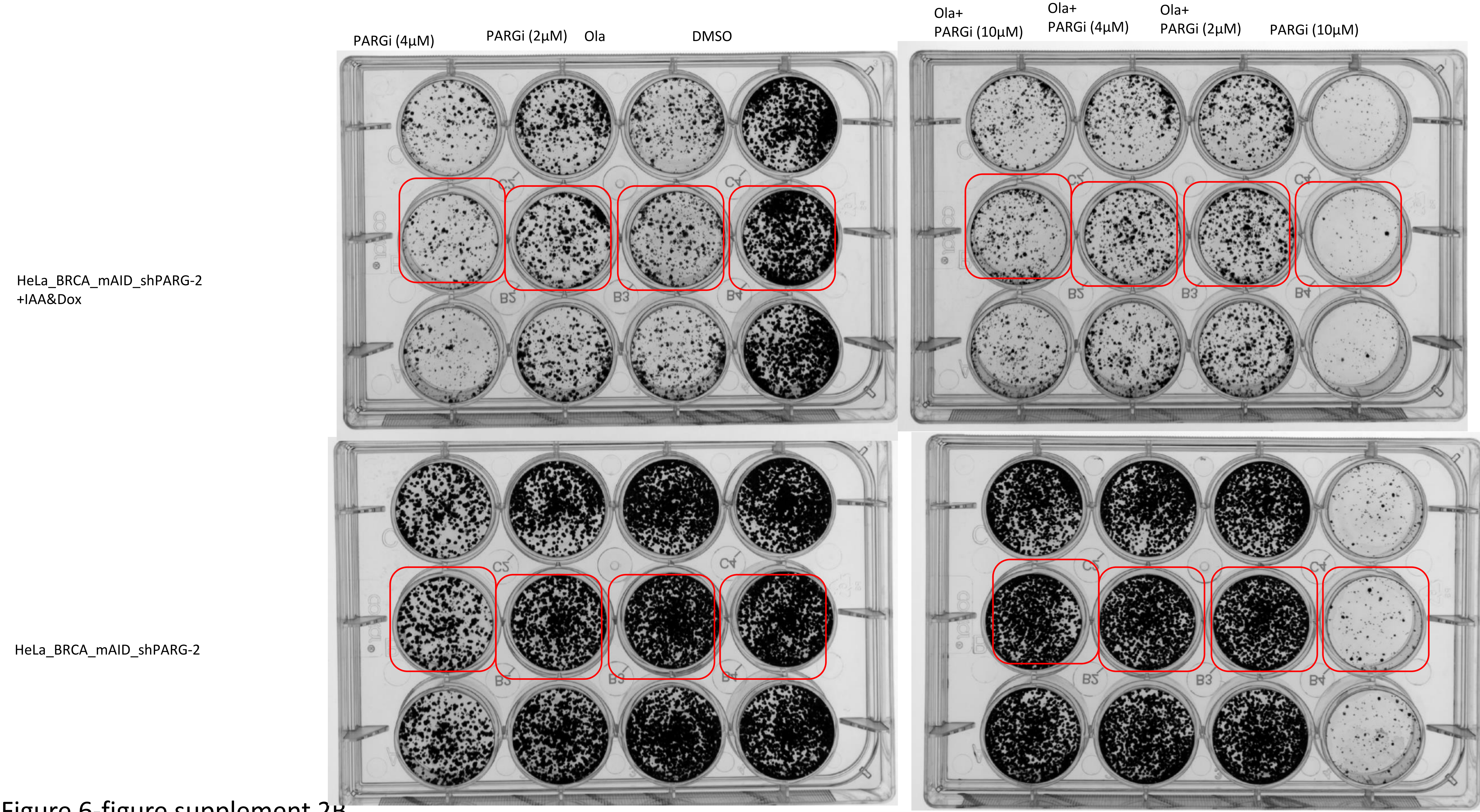

Figure 6-figure supplement 2B
